# Network Properties of Robust Immunity in Plants

**DOI:** 10.1371/journal.pgen.1000772

**Published:** 2009-12-11

**Authors:** Kenichi Tsuda, Masanao Sato, Thomas Stoddard, Jane Glazebrook, Fumiaki Katagiri

**Affiliations:** 1 Department of Plant Biology, Microbial and Plant Genomics Institute, University of Minnesota, St. Paul, Minnesota, United States of America; 2 Department of Life Sciences, Graduate School of Arts and Sciences, The University of Tokyo, Tokyo, Japan; The University of North Carolina at Chapel Hill, United States of America

## Abstract

Two modes of plant immunity against biotrophic pathogens, Effector Triggered Immunity (ETI) and Pattern-Triggered Immunity (PTI), are triggered by recognition of pathogen effectors and Microbe-Associated Molecular Patterns (MAMPs), respectively. Although the jasmonic acid (JA)/ethylene (ET) and salicylic acid (SA) signaling sectors are generally antagonistic and important for immunity against necrotrophic and biotrophic pathogens, respectively, their precise roles and interactions in ETI and PTI have not been clear. We constructed an Arabidopsis *dde2*/*ein2*/*pad4*/*sid2*-quadruple mutant. *DDE2*, *EIN2*, and *SID2* are essential components of the JA, ET, and SA sectors, respectively. The *pad4* mutation affects the SA sector and a poorly characterized sector. Although the ETI triggered by the bacterial effector AvrRpt2 (AvrRpt2-ETI) and the PTI triggered by the bacterial MAMP flg22 (flg22-PTI) were largely intact in plants with mutations in any one of these genes, they were mostly abolished in the quadruple mutant. For the purposes of this study, AvrRpt2-ETI and flg22-PTI were measured as relative growth of *Pseudomonas syringae* bacteria within leaves. Immunity to the necrotrophic fungal pathogen *Alternaria brassicicola* was also severely compromised in the quadruple mutant. Quantitative measurements of the immunity levels in all combinatorial mutants and wild type allowed us to estimate the effects of the wild-type genes and their interactions on the immunity by fitting a mixed general linear model. This signaling allocation analysis showed that, contrary to current ideas, each of the JA, ET, and SA signaling sectors can positively contribute to immunity against both biotrophic and necrotrophic pathogens. The analysis also revealed that while flg22-PTI and AvrRpt2-ETI use a highly overlapping signaling network, the way they use the common network is very different: synergistic relationships among the signaling sectors are evident in PTI, which may amplify the signal; compensatory relationships among the sectors dominate in ETI, explaining the robustness of ETI against genetic and pathogenic perturbations.

## Author Summary

Plants sense molecules originating from pathogens and turn on a battery of immune responses. Activation of immune responses is controlled by a complex network of signaling mechanisms. A traditional approach, knocking out one mechanism at a time, has revealed little about major parts of the signaling network involved in two forms of immunity, Effector-Triggered Immunity (ETI) and Pattern-Triggered Immunity (PTI). ETI and PTI are triggered by different types of pathogen molecules. By simultaneously knocking out four major signaling mechanisms in the network, we demonstrated that a common network comprised of the four signaling mechanisms accounts for most of ETI and PTI triggered by particular molecules. The common network was also important for another form of immunity. We also precisely measured how much each signaling mechanism contributes to ETI and PTI and studied how the signaling mechanisms work together. We found that signaling mechanisms work together synergistically in PTI, which may amplify the signal, while they back up one another in ETI to make the immune signaling highly resistant to pathogen attack (pathogens produce molecules that interfere with immune signaling). Therefore, different forms of plant immunity share the same signaling mechanisms, but they use the same mechanisms in very different ways.

## Introduction

Pattern Triggered Immunity (PTI) and Effector Triggered Immunity (ETI) are forms of plant immunity defined by different modes of pathogen recognition [1]–[3]. In PTI, pattern recognition receptors (PRRs) in the plant plasma membrane recognize molecular structures characteristic of microbes (microbe-associated molecular patterns; MAMPs) [4],[5]. In ETI, a resistance (*R*) gene product, usually inside the plant cell, recognizes a corresponding virulence-promoting effector protein(s) delivered by a pathogen [6]–[9]. Recognition of pathogen attack in PTI and ETI leads to activation of partly overlapping sets of signaling sectors and defense responses [10],[11]. Whereas pathogens well adapted to a particular host plant can prevail over PTI by effector interference with PTI signaling [12]–[15], they typically overcome ETI by evading recognition, not by attacking ETI signaling [16]. In addition, most efforts to obtain mutants defective in ETI yielded only mutations in genes encoding R proteins and proteins required for R protein function. These observations suggest that the ETI signaling network is robust against pathogenic and genetic perturbations. However, the mechanism leading to robust immunity in ETI is yet to be determined.

The salicylic acid (SA) signaling sector is generally important for immunity to biotrophs such as the bacterial pathogen, *Pseudomonas syringae*, while the jasmonic acid (JA) and ethylene (ET) signaling sectors are generally important for immunity to necrotrophs including the fungal pathogen, *Alternaria brassicicola* [17]. The SA and JA/ET sectors are mutually inhibitory in many cases [17]. Exogenously applied SA suppresses the JA sector and promotes susceptibility to *A. brassicicola* [18]. Conversely, *P. syringae* promotes virulence by suppressing the SA sector [19] by producing coronatine [20], a molecular mimic of the active form of JA, JA-isoleucine [21].

In a highly interconnected network, analysis of mutants in which a single signaling sector is blocked provides only a limited view of network structure since phenotypes of single mutants reflect the effects of loss of the disrupted signaling sector as well as loss of interactions with other signaling sectors. As a result, network models derived from such data are incomplete and likely inaccurate. Here we use multiple-mutant analysis to reveal network properties responsible for robust immunity in plants.

## Results

### The signaling network defined by the four genes accounts for ∼80% of AvrRpt2-ETI

To improve understanding of the signaling network in plant innate immunity, we constructed an Arabidopsis *dde2*/*ein2*/*pad4*/*sid2*-quadruple mutant. DDE2, EIN2, and SID2 are essential for JA biosynthesis [22], most of the known ET responses [23], and SA biosynthesis in response to pathogen attack [24], respectively. PAD4 is important for SA accumulation in response to some SA-inducing stimuli [25] and has an SA-independent immune function [26]. Thus, the JA, ET, SA, and PAD4 signaling sectors are all blocked in the quadruple mutant.

**Figure 1:**
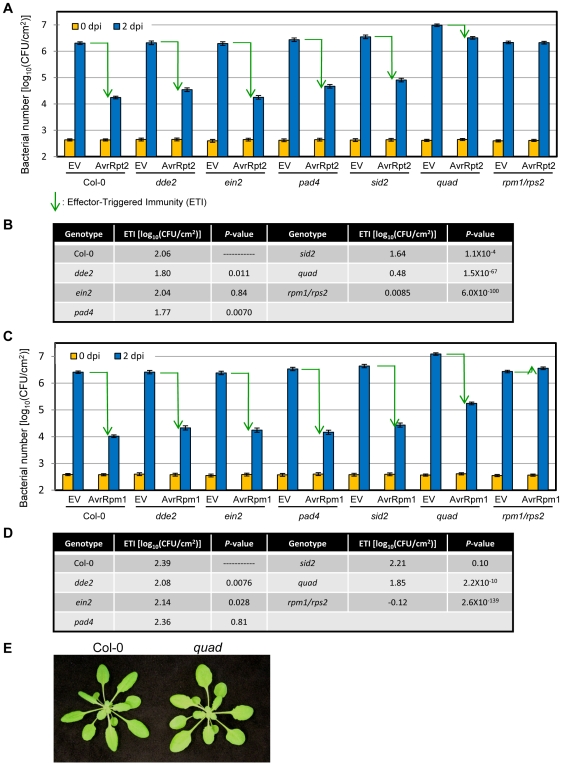
The signaling network defined by the four genes accounts for ∼80% of AvrRpt2-ETI. (A,C) *Pto* DC3000 EV (empty vector), AvrRpt2 (A) or AvrRpm1 (C) (OD_600_ = 0.0001) were infiltrated into leaves of Col-0, *dde2*, *ein2*, *pad4*, *sid2*, *dde2/ein2/pad4/sid2* (quad), and *rpm1/rps2*. The bacterial number was measured at 0 dpi and 2dpi. Data were obtained in four independent experiments (including all genotypes) each with 4 or 12 biological replicates for 0 dpi or 2 dpi, respectively. For Col-0, the quadruple and *rpm1/rps2*, two additional independent experiments each with 4 or 16 biological replicates for 0 dpi or 2 dpi, respectively, were performed and data were integrated with other experiments. Bars represent means and standard errors of data collected in all independent experiments, combined by the mixed linear model. Green arrows indicate the levels of ETI. (B,D) ETI was estimated by subtracting bacterial number at 2 dpi in *Pto* DC3000 (AvrRpt2 or AvrRpm1)-inoculated plants from that in *Pto* DC3000 (EV)-inoculated plants. The ETI level of each mutant was compared with that of Col-0 using a two-tailed *t*-test, to obtain the *P*-values. (E) Four-week old Col-0 and the quadruple mutant.

In the Arabidopsis accession Col-0, the bacterial effector AvrRpt2 is recognized by the R protein, RPS2, resulting in ETI [6],[7]. For the purposes of this study, we defined immunity as the relative level of bacterial growth allowed within leaves. We quantified the level of AvrRpt2-ETI by subtracting log_10_-transformed bacterial number at two days post inoculation (dpi) of a *P. syringae* pv. *tomato* (*Pto*) DC3000 strain expressing AvrRpt2 from that of a *Pto* DC3000 strain carrying the empty vector pLAFR (EV). We observed ∼80% reduction of AvrRpt2-ETI in the quadruple mutant compared to wild-type and reductions of 13%, 1%, 14% or 20% in *dde2*, *ein2*, *pad4* or *sid2* single mutants, respectively (Figure 1A and 1B). Therefore, the effect of the quadruple mutation was larger than the sum of the effects of the four single mutations. We also conducted a similar experiment using a ten-fold lower inoculation dose and observed an indistinguishable effect of the quadruple mutation on AvrRpt2-ETI while the overall bacterial numbers at 2 dpi were ten-fold lower (Figure S1). This observation with a lower dose inoculum indicates that the effect of the quadruple mutation on AvrRpt2-ETI (Figure 1A and 1B) did not result from growth saturation of *Pto* DC3000 EV in the quadruple mutant. Therefore, we conclude that the network defined by the four genes is responsible for 80% of the bacterial growth restriction observed in wild-type plants, which we term AvrRpt2-ETI.

ETI triggered by two other bacterial effectors, AvrRpm1 and AvrPphB, which are recognized by the R proteins RPM1 and RPS5 in Col-0 [8],[9], was reduced by 20% and 50% in the quadruple mutant, respectively (Figure 1C and 1D, and Figure S2). These results indicate that the signaling network defined by the four genes is required to variable extents in the three cases of ETI even though all these effectors originated from *P. syringae* and are recognized by the CC-NB-LRR class of R proteins [27]. In the corresponding *R* gene mutants, ETI was negligible or slightly negative; the latter is likely due to effector virulence functions [28] (Figure 1A–1D and Figure S2). Since the quadruple mutant was morphologically normal, developmental differences are unlikely to explain its immune phenotype (Figure 1E).

ETI is often associated with a hypersensitive response (HR) [29], a form of rapid plant cell death. All three effectors elicited a strong macroscopic HR in wild-type plants. In the quadruple mutant, AvrRpm1-induced HR was similar to the wild type, but HR induced by AvrRpt2 or AvrPphB was undetectable or weak (Figure S3A and S3B). We quantified the strength of the HR triggered by AvrRpt2 by monitoring electrolyte leakage. The quadruple mutant inoculated with *Pto* DC3000 AvrRpt2 clearly displayed less ion leakage than wild-type and was indistinguishable from the quadruple mutant inoculated with *Pto* DC3000 EV (Figure S3C), indicating that the signaling network defined by the four genes controls AvrRpt2-triggered HR. We observed more ion leakage in the quadruple mutant inoculated with *Pto* DC3000 AvrRpm1 than wild-type (Figure S3C). The signaling network defined by the four genes may negatively regulate AvrRpm1-triggered HR. These results indicate that the mechanisms that trigger HR differ between these two cases of ETI.

### The signaling network defined by the four genes accounts for ∼80% of flg22-PTI

**Figure 2:**
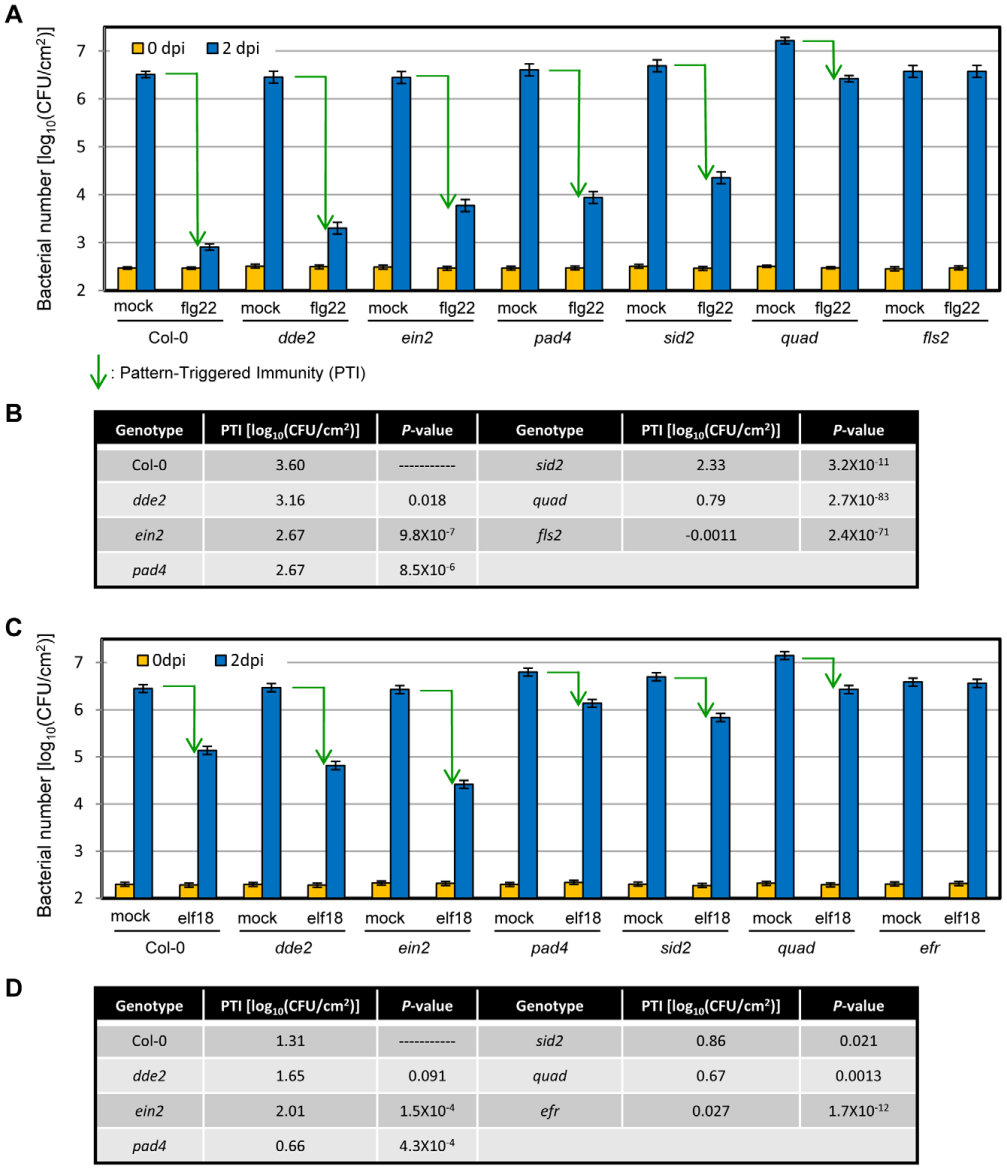
The signaling network defined by the four genes accounts for ∼80% of flg22-PTI. (A,C) *Pto* DC3000 (OD_600_ = 0.0001) was inoculated into the same leaves one day after pretreatment with water (mock), 1 µM flg22 (flg22) (A) or 1 µM elf18 (elf18) (C). The bacterial number was measured at 0 dpi and 2 dpi. Data were obtained in three (A) or four (B) independent experiments each with at least 4 or 12 biological replicates for 0 dpi or 2 dpi, respectively. Bars represent means and standard errors of data collected in all independent experiments, combined by the mixed linear model. Green arrows indicate the levels of PTI. (B,D) PTI was estimated by subtracting bacterial number at 2 dpi in flg22- (B) or elf18- (D) pretreated plants from that in mock-pretreated plants. The PTI level of each mutant was compared with that of Col-0 using a two-tailed *t*-test, to obtain the *P*-values.

MAMP-induced resistance against virulent *Pto* DC3000 is considered to be a good measure of PTI induced by the MAMP [4],[11]. Flg22 is a MAMP derived from bacterial flagellin [30]. As previously reported [4],[11], pretreatment with flg22 induces immunity in plants observable as suppression of bacterial multiplication (Figure 2A). Here we refer to flg22-induced resistance as flg22-PTI. Flg22-PTI was defined as the difference in log_10_-transformed bacterial number of *Pto* DC3000 between water-pretreated (mock) and flg22-pretreated leaves at two dpi. The bacterial growth suppression by flg22 pretreatment may only represent limited aspects of PTI in natural situations. However, we employed this experimental system because it allows quantification of the specific effect of the well-defined MAMP flg22 in respect to the state of no induced resistance in mock-pretreated plants. Consequently, we were able to define the percentage of the flg22-specific effect on bacterial growth that was abolished in particular plant mutants. Previous analysis of single mutants suggested that the JA and ET sectors have no effect on flg22-PTI, while the SA sector has a modest effect [4],[11],[31]. Our results were very similar to these previous observations (Figure 2A and 2B). In contrast, analysis of the quadruple mutant revealed that the signaling network defined by the four genes accounted for ∼80% of flg22-PTI (Figure 2A and 2B). Thus, multiple mutant analysis revealed the fundamental importance of the SA, JA, and ET sectors in flg22-PTI, which was not clear from analysis of single mutants.

We also measured elf18-PTI to *Pto* DC3000. Elf18 is derived from the bacterial elongation factor Tu [32]. Elf18-PTI is weaker than flg22-PTI in the wild-type (Figure 2C and 2D). In the quadruple mutant ∼50% of elf18-PTI was lost (Figure 2D). However, the level of immunity remaining in the quadruple mutant was very similar in flg22- and elf18-PTI (Figure 2B and 2D). These results indicate that the stronger effect of flg22 than elf18 on PTI is entirely dependent on the signaling network defined by the four genes. For both flg22 and elf18, no PTI was detected in the corresponding PRR mutants, *fls2* and *efr*, respectively (Figure 2A–2D), indicating that the PTI observed was completely dependent on specific recognition by the PRRs.

**Figure 3:**
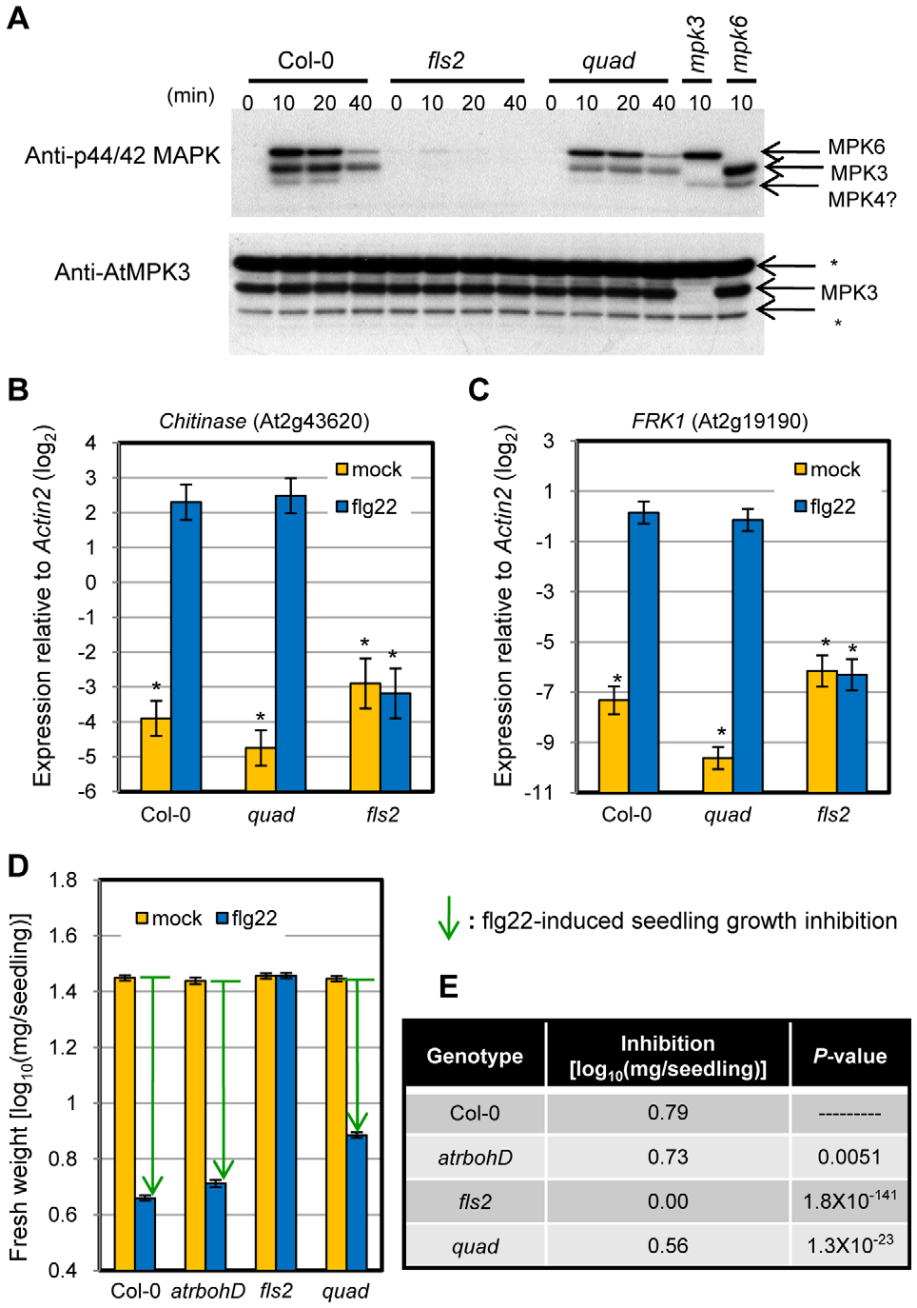
Early signaling events in flg22 response are intact in the quadruple mutant. (A) Eleven-day-old seedlings were treated with 1 µM flg22 and samples were collected 0 to 40 min after treatment as indicated. Activated MAPKs were detected by immunoblotting using anti-p44/42 MAPK antibody. Proteins were also detected with anti-AtMPK3 antibody. Experiments were conducted twice with similar results. Asterisks, non-specific bands. (B,C) Water (mock) or 1 µM flg22 (flg22) were infiltrated into 4-week-old leaves. Expression levels were measured by qRT-PCR. (B) *Chitinase* (At2g43620). (C) *FRK1* (At2g19190). Bars represent means and standard errors of two biological replicates calculated by the mixed linear model. The vertical axis is the log_2_ expression level relative to that of *Actin2* (At2g18780). Asterisks indicate significant differences from flg22-treated Col-0 (*P*<0.01, two-tailed *t*-tests). (D) Five-day-old seedlings were treated with or without 1 µM flg22 for 10 days. Three independent experiments were performed with approximately twenty seedlings per treatment per experiment. Bars represent means and standard errors of data collected in three independent experiments, combined by the mixed linear model. Arrows indicate flg22-induced seedling growth inhibition. Note that the vertical axis is the log_10_-transformed values. (E) Flg22-induced seedling growth inhibition was calculated by subtracting weight in flg22-treated seedlings from that in mock-treated seedlings. Two-tailed *t*-tests were used for *P*-values as in Figure 1B.

Flg22-induced MAP kinase (MPK3/6) activation (Figure 3A) and expression of early flg22-responsive genes such as a *Chitinase* (Figure 3B) and *FRK1* (Figure 3C) were comparable between the quadruple mutant and wild type (Col-0). While these responses may contribute to flg22-PTI, they do not require the signaling network defined by the four genes. Inhibition of seedling growth by flg22 [33] was only slightly reduced in the quadruple mutant (Figure 3D and 3E), indicating that seedling growth inhibition by flg22 is mediated mainly by mechanisms other than the signaling network defined by the four genes.

### Immunity to a necrotrophic fungal pathogen is severely compromised in the quadruple mutant

**Figure 4:**
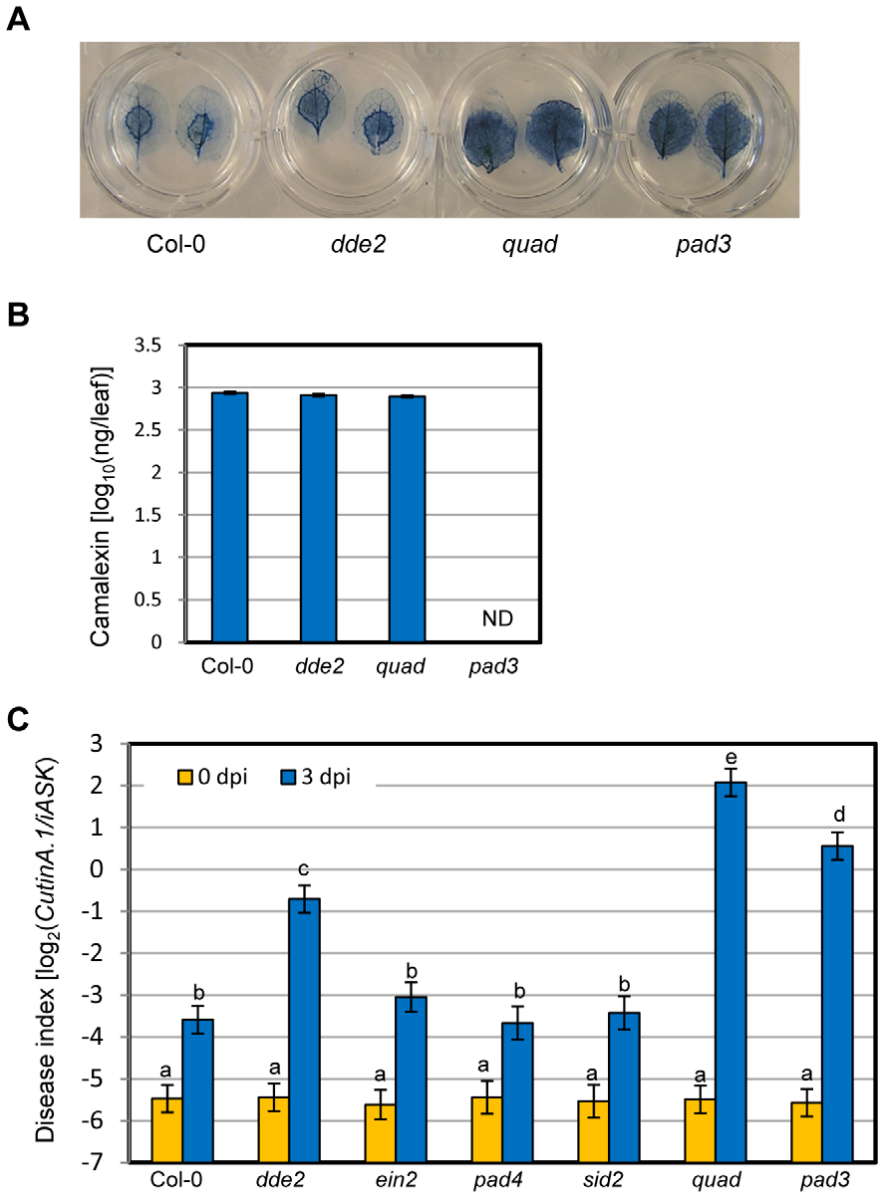
The quadruple mutant was highly susceptible to the necrotrophic pathogen *Alternaria brassicicola*. (A) The damage caused by *A. brassicicola* was visualized by trypan blue staining 3 dpi. (B) Camalexin was extracted from inoculated leaves 3 dpi and quantified. Data were obtained in two independent experiments each with 12 biological replicates per treatment. Camalexin was not detectable (ND) in the *pad3* mutant in which the enzyme for the last biosynthetic step is deficient [58]. Bars represent means and standard errors of data collected in two independent experiments, combined by the mixed linear model. Note that the vertical axis is the log_10_-transformed values. (C) The log_2_ ratio of copy numbers of a fungal gene (*CutinA.1*) and a plant gene (*iASK*) was determined by qPCR and used as the disease index. Each sample consisted of 6 to 8 leaves or 16 to 18 for 0 dpi or 3 dpi, respectively, per genotype per experiment. Data were obtained in six independent experiments (including all genotypes). For Col-0, *dde2*, the quadruple and *pad3*, four additional independent experiments were performed and data were integrated with other experiments. Bars represent means and standard errors of data collected in all independent experiments, combined by the mixed linear model. Different letters indicate significantly different disease index values (a mixed linear model and two-tailed *t*-tests; *P*<0.0001).

Chitin from fungal cell walls is recognized by a LysM receptor kinase and contributes to induction of immunity to the fungal necrotroph *A. brassicicola* [34],[35]. Although the JA sector is important for immunity against this necrotroph [17], the plant immune mechanisms involved are still largely unclear. The quadruple mutant showed greater susceptibility than *dde2* when damaged plant tissue was visualized by trypan blue staining (Figure 4A). The quadruple mutant was also more susceptible than a *pad3* mutant, which is known to be highly susceptible, presumably due to lack of the phytoalexin, camalexin [36] (Figure 4A). In the quadruple mutant, camalexin accumulation was comparable to Col-0 (Figure 4B), indicating that the susceptibility in the quadruple mutant is not due to loss of camalexin. To quantify disease severity, we extracted DNA from infected leaves and determined the ratio of the fungal genome copy number to the plant genome copy number using quantitative PCR. We call the log_2_-transformed ratio value the disease index. We confirmed that disease severity correlates well with the disease index (Figure 4C). The susceptibility in *ein2*, *pad4* and *sid2* single mutants was indistinguishable from Col-0, suggesting that complex interactions among the sectors are involved in the great susceptibility in the quadruple mutant.

### Defense signaling allocation analysis

**Figure 5:**
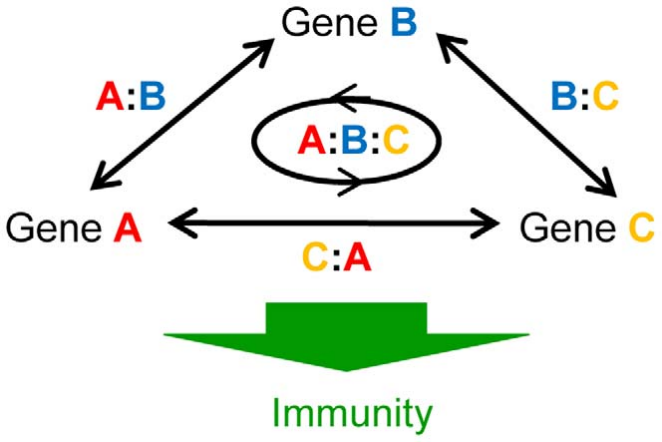
Schematic illustration of the effects of genes and interactions in a signaling network consisting of three signaling sectors. In an interconnected signaling network, not only each gene (signaling sector) but also interactions (interactions among signaling sectors) affect immunity. Colons represent interactions.

Figure 5 illustrates a hypothetical network consisting of three signaling sectors A, B, and C. The output of the network, immunity, is determined by a combination of the effects of each sector A, B, and C and their interactions, A:B, A:C, B:C and A:B:C. If signaling sector A is depleted by mutation *a*, the output loses not only the contribution from sector A but also those from interactions A:B, A:C and A:B:C. Therefore, if the interactions are significant, the sole effect of sector A cannot be estimated based on comparison of the phenotypes of wild type and mutant *a*. To determine the sole effect of sector A, a comparison of the phenotypes of an *a*/*b*/*c* triple mutant in which none of the sectors are functional and a *b*/*c* double mutant, in which only sector A is functional, is required. To expand this concept to the four-sector (gene) situation, we constructed all combinations of double and triple mutants for the four genes, *DDE2*, *EIN2*, *PAD4*, and *SID2*. These double and triple mutants were morphologically normal (data not shown).

**Figure 6:**
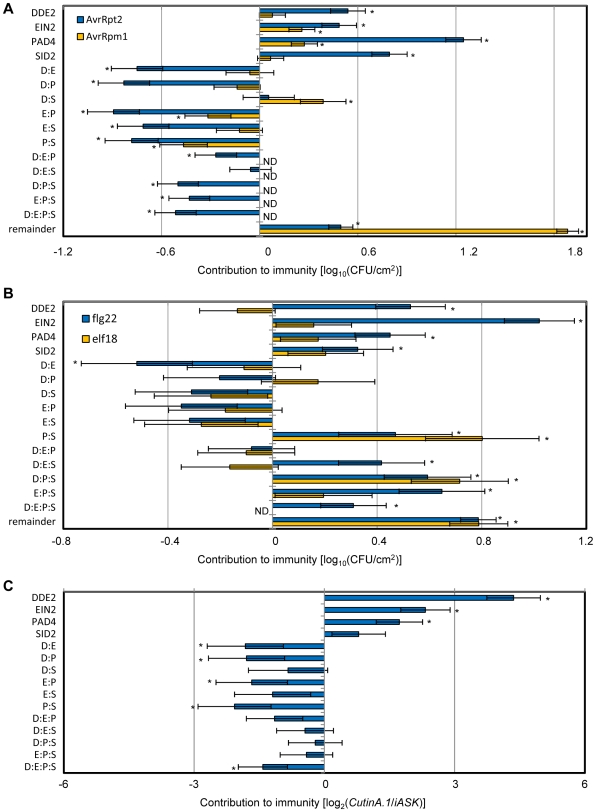
The defense signaling allocations. Positive values represent positive contributions to immunity. (A) The signaling allocations for AvrRpt2- and AvrRpm1-ETI. The 3- and 4-gene interactions for AvrRpm1 were not determined (ND) since the model without the 3- and 4-gene interactions had the lowest Akaike's Information Criterion (AIC). (B) The signaling allocations for flg22- and elf18-PTI. The 4-gene interaction contribution for elf18 was not determined (ND) since the model without the 4-gene interaction had the lowest AIC. (C) The signaling allocations for immunity to *A. brassicicola*. D, *DDE2*; E, *EIN2*; P, *PAD4*; S, *SID2*. Colons indicate interactions. Bars represent means and standard errors determined by the mixed general linear model. Asterisks, significant effects or interactions (*P*<0.05).

We measured levels of AvrRpt2-ETI in all the quadruple, triple, double and single mutants and the wild-type (Figure S4, Table S2, and Table S3). Quantitative measurement with a large number of replicates allowed us to estimate the effects of the wild-type genes (not the effects of the mutations) and of their interactions on immunity (Figure 6A) by fitting the following mixed general linear model:

−log_10_(Bacterial number)∼*a_1_*(*DDE2*) + *a_2_*(*EIN2*) + *a_3_*(*PAD4*) + *a_4_*(*SID2*) + *a_5_*(remainder) + *b_1_*(*DDE2:EIN2*) + *b_2_*(*DDE2:PAD4*) + *b_3_*(*DDE2:SID2*) + *b_4_*(*EIN2:PAD4*) + *b_5_*(*EIN2:SID2*) + *b_6_*(*PAD4:SID2*) + *c_1_*(*DDE2:EIN2:PAD4*) + *c_2_*(*DDE2:EIN2:SID2*) + *c_3_*(*DDE2:PAD4:SID2*) + *c_4_*(*EIN2:PAD4:SID2*) + *d*(*DDE2:EIN2:PAD4:SID2*) + genotype +1|replicate/pot

The values shown in Table 1 were assigned to the variables for the single wild-type gene effects and the 2-, 3-, and 4-wild type gene interactions after inoculation with *Pto* DC3000 AvrRpt2. The remainder represents the remaining immunity in the quadruple mutant. Interactions among the four genes and this remainder of an unknown origin cannot be determined as the remainder cannot be manipulated by the mutations. Note that not all the values assigned are 0 or 1 but that some are fractions (hence, it is a general linear model). The reason for the fractional values is explained in Text S1. When a plant was inoculated with *Pto* DC3000 EV, 0 was assigned to all these variables. In this way, the genotype factor of fixed effects captures the log_10_-bacterial number differences among the genotypes when the plants were inoculated with *Pto* DC3000 EV. The random factors, 1|replicate/pot, indicate that for each independent replicated experiment, plants were grown in multiple pots. The negative of the log_10_-bacterial number was used for the analysis, so obtained positive coefficients indicated positive contributions to immunity. Thus, AvrRpt2-ETI was dissected into effects of single genes *a_1–4_*, two-gene interactions *b_1–6_*, three-gene interactions *c_1–4_*, the four-gene interaction *d*, and the remainder *a_5_*.

**Table 1:** Values assigned to the variables for the single gene effects and interactions.

genotype	gene effect and interaction															
	*DDE2*	*EIN2*	*PAD4*	*SID2*	*D:E*	*D:P*	*D:S*	*E:P*	*E:S*	*P:S*	*D:E:P*	*D:E:S*	*D:P:S*	*E:P:S*	*D:E:P:S*	remainder
wild type	1	1	1	1	1/6	1/6	1/6	1/6	1/6	1/6	1/4	1/4	1/4	1/4	1	1
*dde2*	0	1	1	1	0	0	0	1/3	1/3	1/3	0	0	0	1	0	1
*ein2*	1	0	1	1	0	1/3	1/3	0	0	1/3	0	0	1	0	0	1
*pad4*	1	1	0	1	1/3	0	1/3	0	1/3	0	0	1	0	0	0	1
*sid2*	1	1	1	0	1/3	1/3	0	1/3	0	0	1	0	0	0	0	1
*dde2/ein2*	0	0	1	1	0	0	0	0	0	1	0	0	0	0	0	1
*dde2/pad4*	0	1	0	1	0	0	0	0	1	0	0	0	0	0	0	1
*dde2/sid2*	0	1	1	0	0	0	0	1	0	0	0	0	0	0	0	1
*ein2/pad4*	1	0	0	1	0	0	1	0	0	0	0	0	0	0	0	1
*ein2/sid2*	1	0	1	0	0	1	0	0	0	0	0	0	0	0	0	1
*pad4/sid2*	1	1	0	0	1	0	0	0	0	0	0	0	0	0	0	1
*dde2/ein2/pad4*	0	0	0	1	0	0	0	0	0	0	0	0	0	0	0	1
*dde2/ein2/sid2*	0	0	1	0	0	0	0	0	0	0	0	0	0	0	0	1
*dde2/pad4/sid2*	0	1	0	0	0	0	0	0	0	0	0	0	0	0	0	1
*ein2/pad4/sid2*	1	0	0	0	0	0	0	0	0	0	0	0	0	0	0	1
*dde2/ein2/pad4/sid2*	0	0	0	0	0	0	0	0	0	0	0	0	0	0	0	1

**Figure 7:**
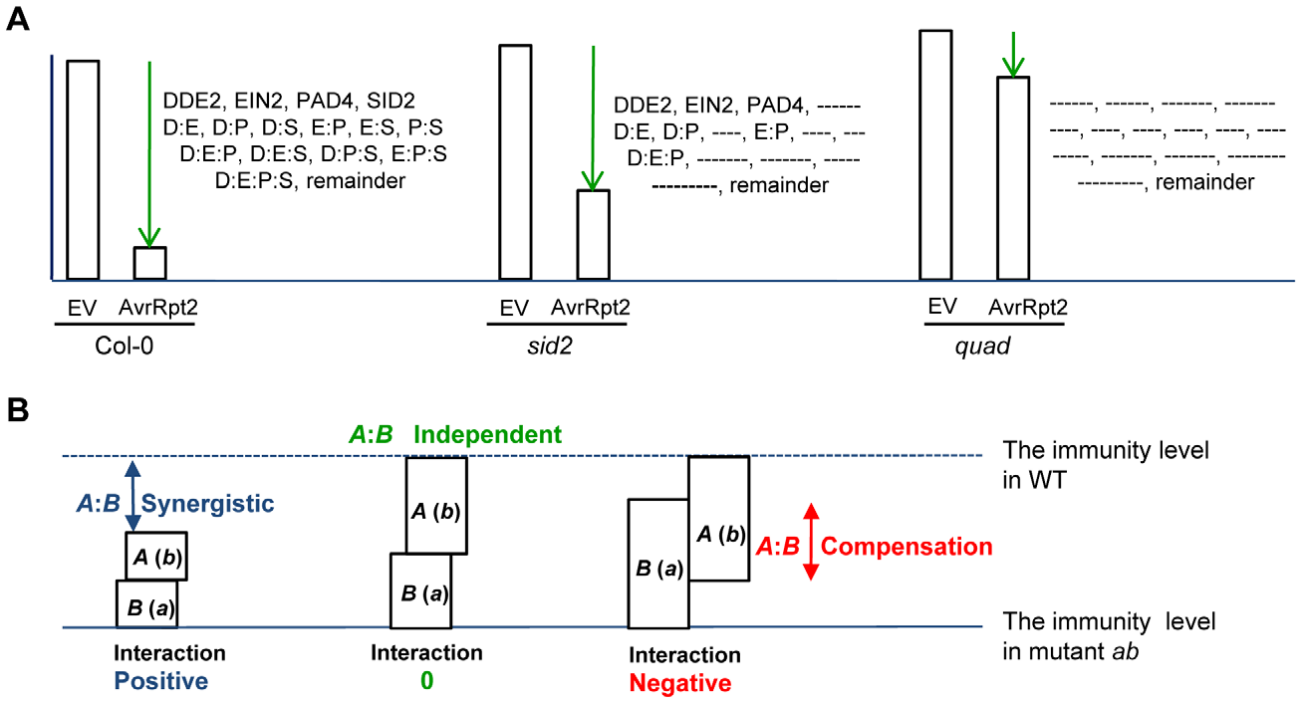
Interpretations of signaling allocation analysis and of the interaction term. (A) In the wild-type, effects of each gene and the remainder and their interactions contribute to AvrRpt2-ETI. In *sid2*, effects of *DDE2*, *EIN2*, *PAD4* and the remainder and *DDE2*:*EIN2*, *DDE2*:*PAD4*, *EIN2*:*PAD4* and *DDE2*:*EIN2*:*PAD4* interactions contribute to AvrRpt2-ETI. In the quadruple mutant, only the remainder effect contributes to AvrRpt2-ETI. (B) Interpretation of an interaction term in a hypothetical network consisting of two sectors A and B. Effect A is the level of immunity in mutant *b*, and effect B in mutant *a*. If the sum of effects A and B is lower than the immunity level in WT, the A:B interaction is positive, indicating that there is a synergistic effect between sectors A and B. If the sum of effects A and B is equal to the immunity level in WT, the A:B interaction is zero, indicating that sectors A and B are independent. If the sum of effects A and B is higher than the immunity level in WT, the A:B interaction is negative, indicating that each of the sectors A and B can compensate for loss of the other.

We call this procedure signaling allocation analysis. In the wild-type, effects of each gene and the remainder and their interactions contribute to AvrRpt2-ETI (Figure 7A). In *sid2*, effects of *DDE2*, *EIN2*, *PAD4* and the remainder and *DDE2*:*EIN2*, *DDE2*:*PAD4*, *EIN2*:*PAD4* and *DDE2*:*EIN2*:*PAD4* interactions contribute to AvrRpt2-ETI (Figure 7A). In the quadruple mutant, only the remainder effect contributes to AvrRpt2-ETI (Figure 7A). In other genotypes, the contributions of the effects of genes and their interactions can be assigned similarly.

Figure 7B illustrates interpretation of an interaction term in a hypothetical network consisting of two sectors A and B. Effect A is the level of immunity in mutant *b*, and effect B in mutant *a*. If the sum of effects A and B is lower than the immunity level in WT, the A:B interaction is positive, meaning that there must be a synergistic effect between sectors A and B. If the sum of effects A and B is equal to the immunity level in WT, the A:B interaction is zero, indicating that sectors A and B are independent. If the sum of effects A and B is higher than the immunity level in WT, the A:B interaction is negative, meaning that each of the sectors A and B can compensate for loss of the other. Note that this compensation in the immunity does not necessarily imply mechanistic similarity, i.e., sectors A and B may regulate completely separate sets of defense components that nevertheless compensate for one another in restricting pathogen growth.

### The robust AvrRpt2-ETI is achieved by compensatory interactions among positively contributing signaling sectors

The signaling allocation for AvrRpt2-ETI indicates that all single gene effects were positive (Figure 6A). In fact, the level of AvrRpt2-ETI in the quadruple mutant was lower than in any of the triple mutants (Figure S4A), meaning that each single signaling sector, including the JA and ET sectors, positively contributed to AvrRpt2-ETI, as defined by the suppression of bacterial growth. These positive contributions to immunity of the JA and ET sectors appear to contradict previous general conclusions based on analyses of single mutants or responses to exogenously applied hormones [17]. The signaling allocation analysis revealed the positive contributions of the JA and ET sectors by dissecting complex interactions among the sectors. Most of the interaction terms were negative (Figure 6A), indicating that the signaling sectors can compensate each other (Figure 7B). For instance, the *PAD4* and *SID2* single gene effects and the *PAD4:SID2* interaction were 1.2, 0.8 and −0.8 (Figure 6A), meaning that PAD4 and SID2 positively contribute to AvrRpt2-ETI and that they can compensate each other's function in AvrRpt2-ETI, suggesting that *PAD4* has a role as a backup in case of loss of the SA (*SID2*) sector. Similarly, the two-gene interactions *DDE2*:*EIN2*, *DDE2*:*PAD4*, *EIN2*:*PAD4* and *EIN2*:*SID2*, were all negative, indicating that most of the signaling sectors can compensate each other in case of losses of sectors in AvrRpt2-ETI. This network compensation among positively contributing signaling sectors should make AvrRpt2-ETI highly resistant to genetic and pathogenic perturbations since when one sector is disrupted, other signaling sectors compensate for its loss.

The signaling allocation analysis was also applied to AvrRpm1-ETI (Figure 6A and Figure S5, Table S4 and Table S5). The overall tendency up to the 2-gene interactions was similar to that in the AvrRpt2-ETI, although the overall contribution of the signaling network defined by the four genes was weaker and the contribution of the remainder variable was higher in the AvrRpm1-ETI than the AvrRpt2-ETI.

### PTI involves synergistic interactions among signaling sectors

For signaling allocation analyses, we measured the levels of MAMP-PTI in all combinatorial mutants (Figure S6 and Figure S7, Tables S6, S7, S8, S9). The signaling allocation analysis of flg22-PTI revealed that all single signaling sectors contribute positively to the immunity and that some interactions, such as the *PAD4*:*SID2* interaction, were positive (Figure 6B). A positive feedback loop comprised of PAD4 and SID2 that amplifies the SA signal [37] likely explains this synergistic interaction. The three-gene interactions *DDE2:PAD4:SID2*, *DDE2:EIN2:SID2* and *DDE2:PAD4:SID2* and the four-gene interaction were also positive (Figure 6B), indicating that there are synergistic interactions between the SA and JA/ET sectors. There are some differences in the signaling allocation between flg22- and elf18-PTI. For example, most of the EIN2-related interactions in elf18-PTI were negative (Figure 6B) and single *EIN2* gene effect was much smaller than that in flg22-PTI, which explains the observation that elf18-PTI in *ein2* plants is stronger than that in wild-type plants but not in the case of flg22-PTI (Figure S6B and Figure S7B). However, overall the signaling allocation of elf18-PTI is similar to that of flg22-PTI although the overall effect size is smaller with elf18 (Figure 6B). These results indicate that unlike ETI, PTI depends heavily on synergistic interactions among the signaling sectors.

### All the signaling sectors can contribute positively to immunity to a necrotrophic fungal pathogen

For a signaling allocation analysis, we measured the disease indexes for *A. brassicicola* infection with all the combinatorial mutants (Figure S8 and Table S10). Since the disease index for no immunity cannot be determined, the remainder term and the genotype factor were eliminated from the signaling allocation model. All the single gene effects were positive although the contribution from *DDE2* was clearly the largest (Figure 6C). All gene interactions were negative (Figure 6C), indicating all signaling sectors can compensate each other. The results of the signaling allocation analysis imply that while the JA sector is the primary signaling sector for immunity to *A. brassicicola*, the ET, SA and PAD4 sectors contribute to robust immunity. For example, although the reduction of immunity in the *dde2* single mutant was 2.9 disease index units (Figure 4C), it was smaller than the *DDE2* single gene effect of 4.3 units (Figure 6C), indicating that other sectors partly compensate the JA sector.

## Discussion

We quantified immunity triggered by MAMPs and effectors by relative growth of *Pto* DC3000 strains within leaves in the Arabidopsis quadruple mutant *dde2/ein2/pad4/sid2*, all the combinatorial mutants and wild-type plants. We have termed the measured restriction of bacterial growth due to a MAMP or an effector MAMP-PTI and effector-ETI, respectively. A striking revelation is that the same network defined by the four genes accounted for ∼80% of both flg22-PTI and AvrRpt2-ETI (Figure 1A and Figure 2A), indicating that the signaling machinery is likely highly conserved between PTI and ETI. If the notion that PTI evolved before ETI [1]–[3] is correct, this observation may suggest that while acquisition of a new class of recognition mechanism (i.e., R proteins) was necessary for evolution of ETI, ETI adapted the rest of the defense machinery mostly from the already existing PTI. However, use of the overlapping signaling network in PTI and ETI is very different as interactions among the signaling sectors are very different (Figure 6A and 6B). Note that the general signaling allocation patterns within the network are characteristic for different types of immunity: strong positive interactions of *PAD4*:*SID2* and *DDE2*:*PAD4*:*SID2* stand out in flg22- and elf18-PTI (Figure 6B); most gene interactions are negative in AvrRpt2- and AvrRpm1-ETI (Figure 6A). In all cases, the immunity was measured by growth suppression of *Pto* DC3000-derived strains. These differences between immunity types suggest that while immunity is enhanced by synergistic sector interactions in PTI, robust immunity to perturbations of signaling sectors is achieved by compensation functions among the sectors in ETI. In other words, PTI can be substantially impaired by a pathogen effector that disrupts one of the synergistically interacting sectors, while ETI is robust against such disruption. It will be interesting to investigate whether or not the bacterial effectors that suppress PTI signaling [12]–[15] are able to overcome ETI.

While overall signaling allocation patterns were similar between AvrRpt2-ETI and AvrRpm1-ETI, levels of the dependency on the signaling network defined by the four genes were very different, ∼80% and ∼20%, respectively. The difference in the dependency on the signaling network may be explained by a difference in the effectiveness of early immune responses. In flg22-PTI, the signaling network defined by the four genes did not have substantial roles in induction of early responses (Figure 3A and 3B). By extending this observation, we speculate that also in ETI the signaling network defined by the four genes mainly controls late immune responses, In AvrRpm1-ETI, early immune responses may be more effective, and consequently the contribution from late immune responses controlled by the signaling network may be less important. In contrast, early immune responses may not contribute as much to AvrRpt2-ETI, and consequently the contribution from the signaling network may be more important. Consistent with this hypothesis, it is well documented that HR induced by AvrRpm1 is more rapid than that induced by AvrRpt2 [38]. The contribution of early immune responses to AvrPphB-ETI may be somewhere between those in AvrRpm1-ETI and AvrRpt2-ETI, as the level of dependency of AvrPphB-ETI on the network (∼50%) was intermediate.

**Figure 8:**
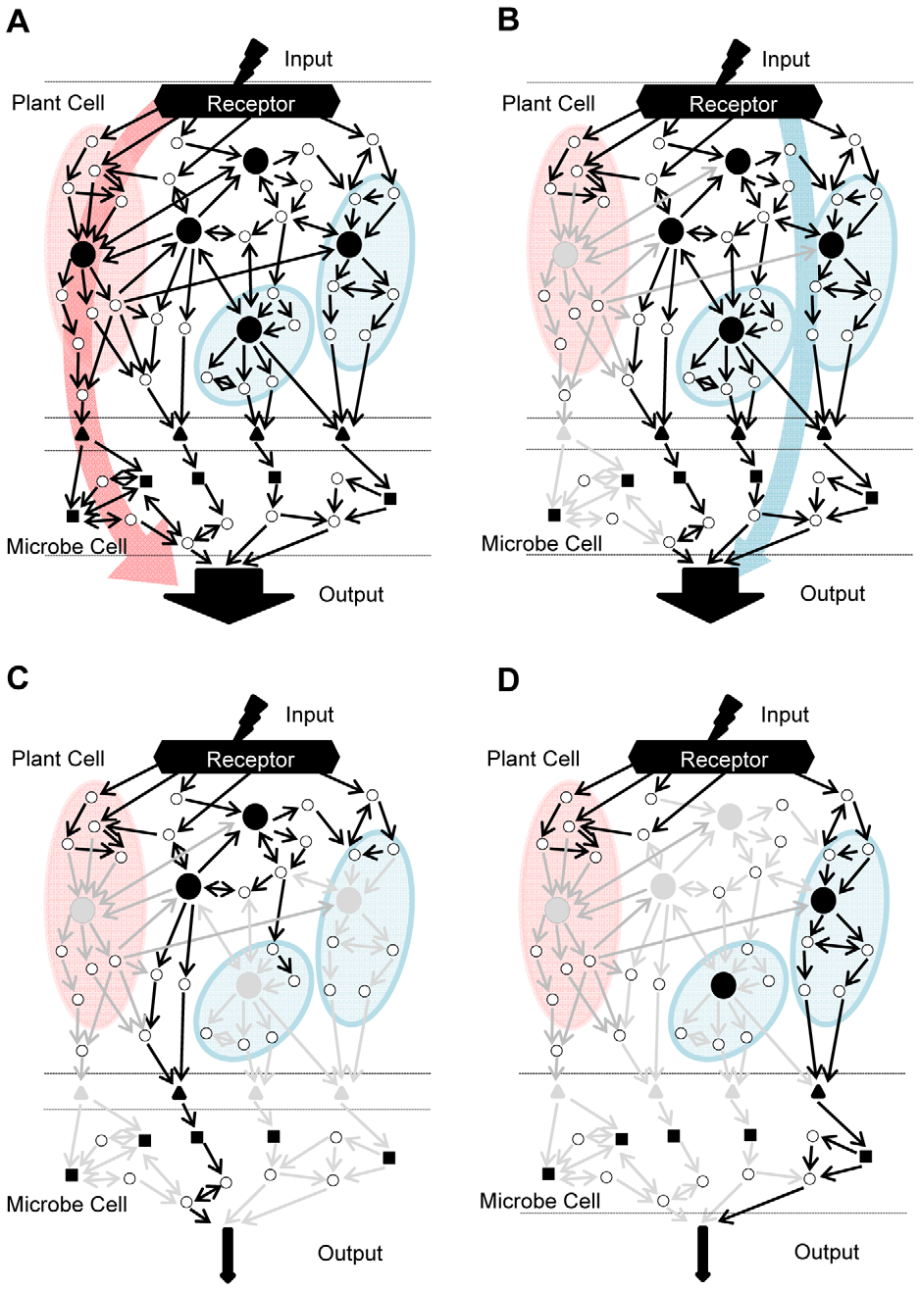
Robust plant immunity is achieved by network compensation (Conceptual diagrams). (A) Many genes (signaling network components, circles) are highly interconnected in the network. The network is not perfectly democratic because there is a primary signal flow, shown by a red arrow, when the network is intact. (B) None of the single sector disruptions have much effect on the output (restriction of pathogen growth), because the signal flow is rerouted, as shown by the blue arrow. (C) Disruption of a sufficient number of sectors results in loss of most output. (D) There could be different combinations of sector disruptions that result in similar levels of output reduction. Input, MAMPs or effectors; Output, pathogen growth inhibition; Large black circles, major hubs in signaling sectors; Triangles, plant defense components directly affecting pathogens; Grey arrows, circles and triangles mean disrupted connections, genes, and defense components, respectively.

In considering a network to explain plant immunity, it is necessary to include a pathogen (Figure 8A): The input of the network is MAMPs or effectors; this input is fed into the complex signaling network in the plant; multiple outputs from the signaling network result in induction of a battery of plant defense components; the plant defense components attack a variety of targets in the pathogen; and the effects on the targets are combined to influence the growth of the pathogen. The network output is restriction of pathogen growth. In this way, robust immunity is understood as the network in which blockages of a small number of sectors (i.e., parts of the network) on the plant side do not have much effect on the output. When each sector influences many other sectors in a network (i.e., it is highly interconnected) and there is no particular specialization among the sectors, the network is called a democratic network [39] because each sector has a similar level of importance. An extreme analogy of a democratic network is percolation through a mesh of water paths: many sectors need to be blocked before the output of water is significantly reduced. On the other hand, networks comprised of signaling sectors that lack interconnections are called autocratic networks [39]. A network in which specific pathways control specific functions is a typical autocratic network. Autocratic networks can be studied as individual signaling sectors in isolation from the others, and are easily studied using reductionist approaches to biological processes. However, biological networks often have architectures somewhere between the democratic and autocratic. We think that the plant ETI network has a democratic property to some extent: when one or two network sectors are blocked, the signal flows through some other sectors can compensate for the loss of the blocked sector(s). As a result, the level of the output, restriction of pathogen growth, does not change much (Figure 8B). However, there is a clear difference between the ETI network and a perfectly democratic network. The SA and JA/ET sectors are mutually inhibitory in many cases [17]. Such mutually inhibitory regulatory relationships between signaling sectors result in a steady state in which a dominant, primary sector suppresses the activities of the others (Figure 8A). Other sectors get heavily used only when the primary sector is blocked (Figure 8B). Thus, other sectors effectively buffer blockages in the primary sector. As both the SA and JA/ET sectors positively contribute to immunity (Figure 6A), the loss of signaling flow through the SA sector is compensated by rerouting signal through the JA/ET sectors. Such compensation between positively contributing sectors explains the robustness of AvrRpt2-ETI as suggested previously [40]. Disruptions of multiple signaling sectors are required to compromise AvrRpt2-ETI (Figure 8C). Since there are clear differences in importance among signaling sectors, the ETI network is not really a democratic network although it has some democratic properties.

General conclusions based on analyses of single mutants and responses induced by exogenous application of hormones are that the SA sector is inhibitory to immunity to necrotrophs and that the JA/ET sectors are inhibitory to immunity to biotrophs [17]. Our observations show that the SA, JA, and ET sectors can contribute positively to immunity to both biotrophs and necrotrophs as single sectors. Mutual inhibition between the SA and JA/ET sectors and differences in the levels of contribution can reconcile the apparent contradiction between the general conclusions and our observations. An important point is that the general conclusions were made without data from plants blocked in all three sectors. In immunity to a biotroph, for example, immunity mediated by the JA/ET sectors is not as strong as that mediated by the primary sector, SA, although the JA/ET sectors can still contribute positively to immunity. When the JA sector is activated by exogenous JA or coronatine from pathogens (a JA-Ile mimic), the JA sector seems to have negative effects on immunity to biotrophs. This is because the backup immunity mediated by the JA sector is not as strong as that mediated by the SA sector, which is inhibited by the strongly activated JA sector.

Another important aspect of a highly interconnected network with some democratic properties is that the concepts of upstream, downstream, and parallel signal flows, which can be clearly defined in an autocratic network, become somewhat obscure. Figure 8C and 8D illustrate this situation: two different combinations of triple mutations result in loss of most resistance, but the relationships among these sectors cannot be clearly defined as upstream, downstream, or parallel. In addition to the signaling sectors we explored in this study, other signaling sectors including the MAP kinases [41] and other hormones such as abscisic acid, auxins and gibberellins [42]–[44], are involved in plant immunity. It is conceivable that different combinations of simultaneous sector disruptions among all these sectors could also lead to loss of most immunity in AvrRpt2-ETI. This view correlates with a property of the extreme analogy of percolation: as long as a sufficient number of sectors are blocked, the water flow becomes nearly zero when the blocked sectors are randomly chosen. It will be necessary to expand this multiple sector disruption approach to a larger network including other signaling sectors in order to elucidate properties of the larger plant immune network.

We took advantage of the model plant Arabidopsis in this study since mutants deficient in defense responses were available. Given that the defense-related genes such as *DDE2*, *EIN2*, *PAD4* and *SID2* which are used in this study are highly conserved among different plant species (data not shown), immune network properties we observed with Arabidopsis may be conserved in other plant species. We observed similar signaling allocations in the network defined by the four genes in two cases of ETI or two cases of PTI although the network defined by the four genes is used to variable extents in each case of ETI or PTI (Figure 1A–1D and Figure 2A–2D). Therefore the network properties we revealed may be common features in ETI and PTI. Further investigation will be required to determine whether these network properties generally apply to various cases of ETI and PTI, including those involving different species of pathogens and hosts.

Network compensation among positively contributing signaling sectors can explain the robustness of plant immunity, particularly AvrRpt2-ETI. We have demonstrated that quantitative analysis of all combinations of multiple mutations that together deplete almost all of the network function provides a powerful approach for elucidation of quantitative relationships among highly interconnected signaling sectors. Similar approaches should benefit analysis of other complex biological networks.

## Materials and Methods

### Plant materials and growth conditions

*Arabidopsis thaliana* accession Col-0 was the background of all mutants used in this study. Arabidopsis *atrbohD* [45], *dde2-2* [46], *ein2-1* [23], *efr-2* [5], *fls2* (SAIL_691C4) [4], *mpk3* (SALK_151594) [47], *mpk6-2* [48], *npr1-1* [49], *pad3-1* [50], *pad4-1* [25], *pmr4-1* [51], *rpm1-3/rps2 101C* [52], *rps5* (SALK_015294) [53] and *sid2-2* [24] were previously described. We generated the double (*dde2-2/ein2-1*, *dde2-2/pad4-1*, *dde2-2/sid2-2*, *ein2-1/pad4-1*, *ein2-1/sid2-2* and *pad4-1/sid2-2*), the triple (*dde2-2/ein2-1/pad4-1*, *dde2-2/ein2-1/sid2-2*, *dde2-2/pad4-1/sid2-2* and *ein2-1/pad4-1/sid2-2*) and the quadruple (*dde2-2/ein2-1/pad4-1/sid2-2*) mutants by standard genetic crosses, tracking the mutations by PCR product length difference or cleaved amplified polymorphic sequence (CAPS) markers. Primers and restriction enzymes used for screening of the mutants are listed in Table S1. The mutants containing *dde2-2* required spraying with MeJA for seed production due to loss of JA biosynthesis [46]. The *ein2-1* and *pad4-1* mutations create premature Stop codons [23],[25]. The *sid2-2* mutation is a deletion [24]. Therefore, mutations used for constructing the quadruple *dde2/ein2/pad4/sid2* mutant are considered null. Arabidopsis plants were grown in a controlled environment at 22°C with a 12 h photoperiod and 75% relative humidity.

### Bacterial growth assay

Bacterial growth assays were performed as described previously [11]. In brief, bacterial suspensions were infiltrated into leaves of 4 to 5 week-old plants using a needleless syringe. Log_10_-transformed colony-forming units (cfu) per cm^2^ leaf surface area were calculated and the models described in Materials and Methods were fit to the data.

### Chemicals

Flg22 and elf18 peptides were purchased from EZBiolab Inc (Westfield, IN, USA). Indicated concentrations of flg22 and elf18 solutions were applied to leaves of 4 to 5 week-old plants by infiltration using a needleless syringe one day before infiltration with bacteria.

### Program used for modeling and statistical analysis

The lme function in the nlme package in the R environment was used.

### Bacterial strains and preparation of inocula

*Pto* DC3000 was grown overnight at room temperature in King's B medium supplemented with 25 µg/ml of rifampicin. *Pto* DC3000 strains carrying the empty vector (pLAFR), AvrRpt2, AvrRpm1 or AvrPphB were grown overnight at room temperature in King's B medium supplemented with 25 µg/ml of rifampicin and 10 µg/ml of tetracycline. The bacteria were harvested by centrifugation, washed, and diluted to the desired density with water.

### Mixed linear models and statistical analysis for bacterial growth assay

The following models were fit to the log_10_-transformed bacterial number data: *S_gtrp_* = *GT_gt_*+*R/P_rp_*+*ε_gtrp_* (Figure 1A–1D, Figures S1, S2, S4, S5); *S_gtrfp_* = *GT_gt_*+*R/F/P_rfp_*+*ε_gtrfp_* (Figure 2A–2D, Figures S6, S7), where *S*, log_10_-transformed bacterial number; *GT*, genotype:treatment interaction, and random factors: *R*, replicate; *F*, flat; *P*, pot; and *ε*, residual. The mean estimates of the genotype:treatment interaction were used as the modeled log_10_-transformed bacterial number. The modeled log_10_-transformed bacterial number values were compared using two-tailed *t*-tests. ETI and PTI values were compared using two-tailed *t*-tests. For the *t*-tests, the standard errors appropriate for each comparison were calculated using the variance and covariance values obtained from the model fitting.

### MAP kinase assay

MAP kinase assays were performed as described previously [54]. Arabidopsis seedlings were grown for 11 days on a medium solidified with 0.8% agar that contained 0.5×MS salts with Gamborg's vitamins (M0404; Sigma) and 1% (w/v) sucrose and then transferred to 12-well plates (six seedlings per well) in which each well contained 3 ml of liquid medium containing 0.5×MS salts with Gamborg's vitamins and 1% (w/v) sucrose with 1 µM flg22 peptide. After 0 to 40 min as indicated, the seedlings were frozen in liquid nitrogen. The frozen seedlings were ground in liquid nitrogen and homogenized in 100 µl of extraction buffer (100 mM HEPES, pH 7.5, 5 mM EDTA, 5 mM EGTA, 2 mM dithiothreitol, 10 mM Na_3_VO_4_, 10 mM NaF, 50 mM ß-glycerolphosphate, 1 mM phenylmethylsulfonyl fluoride, 1 tablet/10 ml extraction buffer of proteinase inhibitor cocktail (11 836 153 001; Roche Applied Science, Indianapolis, IN, USA) and phosphatase inhibitor cocktail (04 906 845 001; Roche Applied Science), 10% glycerol, 1% (w/v) polyvinylpolypyrrolidone). After centrifugation at 13,000 rpm for 30 min at 4°C, supernatants were frozen and stored at −20°C. The protein concentration was determined using a Bradford assay (BIO-RAD, Hercules, CA, USA) with IgG as a standard. Twenty micrograms of protein was separated in an 8% polyacrylamide gel. Immunoblot analysis was performed using anti-phospho-p44/42 MAPK (1∶2000, Cell Signaling Technology, Danvers, MA, USA) and anti-AtMPK3 (1∶2000, Sigma) as primary antibody, and peroxidase-conjugated goat anti-rabbit IgG (1∶15,000, A 6154; Sigma).

### Quantitative RT–PCR analysis

Water (mock) or 1 µM flg22 (flg22) were infiltrated into 4-week-old Col-0, *dde2/ein2/pad4/sid2* or *fls2* plants. Six leaves from 3 plants per sample were collected 3 hpi, frozen in liquid nitrogen and stored at −80°C. Total RNA isolation and real-time PCR analysis was carried out as described previously [11]. Two independent experiments (biological replicates) were performed. The following model was fit to the Ct value data using the lme function in the nlme package in the R environment: *C_tgytr_* = *GYT_gyt_*+*R_r_*+*ε_gytr_*, where *GYT*, gene:genotype:treatment interaction, and random factors; *R*, replicate; *ε*, residual. The mean estimate of the gene:genotype:treatment interaction was used as the modeled Ct value. The relative log_2_ expression values were obtained by subtracting the Ct value of the genes from the Ct value of the *Actin2* gene and compared for each gene using two-tailed *t*-tests. For the *t*-tests, the standard error appropriate for each comparison was calculated using the variance and covariance values obtained from the model fitting.

### Seedling growth inhibition

Flg22-induced seedling growth inhibition assays were performed as described previously [55]. Approximately twenty Arabidopsis seedlings per treatment were grown on a medium solidified with 0.8% agar that contained 0.5×MS salts with Gamborg's vitamins and 1% (w/v) sucrose for 5 days and then transferred to 24-well plates (one seedling per well) in which each well contained 800 µl of liquid medium containing 0.5×MS salts with Gamborg's vitamins and 1% (w/v) sucrose with and without 1 µM flg22 peptide. Seedling fresh weight was recorded 10 days later. From this data, the log_10_-transformed seedling weights were calculated. The following model was fit to the log_10_-transformed seedling weight data using the lme function in the nlme package in the R environment: *S_gtrfp_* = *GT_gt_*+*R/P_rp_*+*ε_gtrp_*, where *S*, log_10_-transformed seedling weight; *GT*, genotype:treatment interaction, and random factors; *R*, replicate and *P*, plate, *ε*, residual. The mean estimate of the genotype:treatment interaction was used as the modeled log_10_-transformed seedling weight. Flg22-induced seedling growth inhibition values were obtained by subtracting the value of seedling weight in flg22-treated samples from the value of seedling weight in mock-treated samples and compared using two-tailed *t*-tests. For the *t*-tests, the standard error appropriate for each comparison was calculated using the variance and covariance values obtained from the model fitting.

### Fungal material and preparation of inocula

*Alternaria brassicicola* strain ATCC96836 was grown on potato dextrose agar (BD, Franklin Lakes, NJ, USA) for 10 days at room temperature with a 12 h photoperiod. Subsequently, the spores were washed from the surface of the plate with 0.02% Tween-20, and hyphae were removed from the suspension by filtering through four layers of cotton cheesecloth. Concentration of spores was determined using a hemocytometer and adjusted to 1×10^5^ spores/ml with 0.02% Tween-20.

### Fungal disease assay

Three-week-old plants were inoculated by placing a 10 µl droplet of spore suspension (1×10^5^ spores/ml) onto the leaf surface. Inoculated plants were kept at 100% RH at 22°C with a 12 h photoperiod for 3 days. DNA was isolated and the relative amount of *A. brassicicola* (*CutinA.1*) DNA to plant (*iASK*) DNA was determined by qPCR as described previously [56]. qPCR analysis was carried out using an ABI7500 Real Time PCR system (Applied Biosystems, Foster city, CA, USA) and the SYBR Green JumpStart Taq ReadyMix (Sigma, Saint Louis, MO, USA). Disease index [log_2_(*CutinA.1/iASK*)] values were obtained by subtracting the Ct value of the *CutinA.1* from the Ct value of the *iASK*. The following model was fit to the disease index data: *S_gytr_* = *GT_gt_*+*R_r_*+*ε_gytr_*, where *S*, disease index; GT, genotype:time interaction, and random factors: *R*, replicate; *ε*, residual. The mean estimate of the genotype:time interaction was used as the modeled disease index value. The disease index values were compared using two-tailed *t*-tests. For the *t*-tests, the standard error appropriate for each comparison was calculated using the variance and covariance values obtained from the model fitting.

### Trypan blue staining

Inoculated leaves were boiled in 2 ml of a staining solution (14 ml of 95% ethanol, 2 ml of water-saturated phenol, 2 ml of Glycerol, 2 ml of lactic acid, 1 ml of water and 0.02 g of trypan blue) and incubated overnight at room temperature. Then samples were destained in destaining solution (2.5 g/ml of chloral hydrate) for 2 days.

### Camalexin assay

Plants were inoculated by placing one droplet of 10 µl of fungal spore suspension onto the leaf surface. Inoculated plants were kept at 100% RH at 22°C with a 12 h photoperiod for 3 days. Each sample consisted of one leaf. Two independent experiments were conducted with 12 replicates per sample per experiment. Camalexin was extracted from Arabidopsis leaves infected with *A. brassicicola* and quantified as described previously [57]. From this data, the log_10_-transformed camalexin amount (ng per leaf) was calculated. The following model was fit to the log_10_-transformed camalexin data using the lme function in the nlme package in the R environment: *S_gp_* = *G_g_*+*R_r_*+*ε_gr_*, where *S*, log_10_-transformed camalexin amount; *G*, genotype, and random factors; *R*, replicate; *ε*, residual. The mean estimate of the genotype was used as the modeled log_10_-transformed camalexin. The modeled log_10_-transformed camalexin values were compared using two-tailed *t*-tests. For the *t*-tests, the standard error appropriate for each comparison was calculated using the variance and covariance values obtained from the model fitting.

### Defense signaling allocation analysis

We estimated the effect of each wild-type gene and the interactions among the wild-type genes, which we call the defense signaling allocation, by fitting mixed general linear models.

For signaling allocation analysis of flg22 or elf18-PTI:

−log_10_(Bacterial number)∼*a_1_*(*DDE2*) + *a_2_*(*EIN2*) + *a_3_*(*PAD4*) + *a_4_*(*SID2*) + *a_5_*(remainder) + *b_1_*(*DDE2:EIN2*) + *b_2_*(*DDE2:PAD4*) + *b_3_*(*DDE2:SID2*) + *b_4_*(*EIN2:PAD4*) + *b_5_*(*EIN2:SID2*) + *b_6_*(*PAD4:SID2*) + *c_1_*(*DDE2:EIN2:PAD4*) + *c_2_*(*DDE2:EIN2:SID2*) + *c_3_*(*DDE2:PAD4:SID2*) + *c_4_*(*EIN2:PAD4:SID2*) + *d*(*DDE2:EIN2:PAD4:SID2*) + genotype+1|replicate/flat/pot

The values shown in Table 1 were assigned to the variables for the single wild-type gene effects and the 2-, 3-, and 4-wild type gene interactions according to the genotype of the plant when the plant was pretreated with a MAMP. The remainder variable represents the remaining immunity in the quadruple mutant. When plants were mock-pretreated, 0 was assigned to all these variables. In this way, the genotype factor captures the log_10_-bacterial number differences among the genotypes when the plants were mock-pretreated. The random factors 1|replicate/flat/pot indicate that for each independent replicated experiment, plants were grown in multiple flats each of which is divided into multiple pots. The negative of the log_10_-bacterial number was used for the response, so obtained positive coefficients indicated positive contributions to immunity. Thus, PTI was dissected into effects of single genes *a_1–4_*, two-gene interactions *b_1–6_*, three-gene interactions *c_1–4_*, the four-gene interaction *d*, and the remaining immunity *a_5_*.

For signaling allocation analysis of immunity against *A. brassicicola*:

From the model design for PTI, the remainder term was removed because the null immunity value was unknown and the genotype factor term was removed because mock treatment in each genotype cannot be defined.

−log_2_(*CutinA.1/iASK*)∼*a_1_*(*DDE2*) + *a_2_*(*EIN2*) + *a_3_*(*PAD4*) + *a_4_*(*SID2*) + ;*b_1_*(*DDE2:EIN2*) + *b_2_*(*DDE2:PAD4*) + *b_3_*(*DDE2:SID2*) + *b_4_*(*EIN2:PAD4*) + *b_5_*(*EIN2:SID2*) + *b_6_*(*PAD4:SID2*) + *c_1_*(*DDE2:EIN2:PAD4*) + *c_2_*(*DDE2:EIN2:SID2*) + *c_3_*(*DDE2:PAD4:SID2*) + *c_4_*(*EIN2:PAD4:SID2*) + *d*(*DDE2:EIN2:PAD4:SID2*) +1|replicate

The values shown in Table 1 were assigned to the variables for the single wild-type gene effects and the 2-, 3-, and 4-wild type gene interactions according to the genotype of the plant, except that no remainder variable was in this model.

Additional Materials and Methods are provided in Text S2.

## Supporting Information

Figure S1 Comparable AvrRpt2-ETI using a ten times lower dose of *Pto* DC3000 derivatives. (A) *Pto* DC3000 EV or *Pto* DC3000 AvrRpt2 (OD_600_ = 0.0001 or OD_600_ = 0.00001) were infiltrated into plants. The bacterial number was measured at 2 dpi. Data were obtained in two independent experiments each with 16 biological replicates. Bars represent means and standard errors determined by a mixed linear model. (B) AvrRpt2-ETI was calculated by subtracting bacterial number in *Pto* DC3000 AvrRpt2-inoculated plants from that in *Pto* DC3000 EV-inoculated plants. Bars represent means and standard errors determined by a mixed linear model.
(0.07 MB PDF)

Figure S2 AvrPphB-ETI in the quadruple mutant. (A) *Pto* DC3000 EV or *Pto* DC3000 AvrPphB (OD_600_ = 0.0001) were infiltrated into plants. Bacterial number was measured at 0 dpi and 2 dpi. Data were obtained in two independent experiments each with 4 or 16 biological replicates for 0 dpi or 2 dpi, respectively. Bars represent means and standard error determined by a mixed linear model. Arrows indicate AvrPphB-ETI. (B) AvrPphB-ETI was calculated by subtracting bacterial number in *Pto* DC3000 (AvrPphB)-inoculated plants from that in *Pto* DC3000 (EV)-inoculated plants. Two-tailed t-tests were used for *P*-values.
(0.09 MB PDF)

Figure S3 The hypersensitive response (HR) triggered by AvrRpt2 was compromised in the quadruple mutant. (A) *Pto* DC3000 EV, *Pto* DC3000 AvrRpm1, *Pto* DC3000 AvrRpt2 or *Pto* DC3000 AvrPphB (OD_600_ = 0.05) were infiltrated into the left halves of the leaves of either Col-0 or the quadruple mutant. Representative leaves at 24 hpi are shown. (B) The number of leaves that showed a macroscopic HR/the total number of leaves infiltrated. (C) Electrolyte leakage measurements following inoculation with *Pto* DC3000 EV, *Pto* DC3000 AvrRpm1 or *Pto* DC3000 AvrRpt2 (OD_600_ = 0.1). Each sample consisted of four leaf discs from two leaves. Two independent experiments were performed with three biological replicates per treatment per experiment. The data from two experiments were combined, and means and standard errors were calculated.
(0.19 MB PDF)

Figure S4 AvrRpt2-ETI in all single, double, triple and the quadruple mutants. (A) *Pto* DC3000 EV or *Pto* DC3000 AvrRpt2 (OD_600_ = 0.0001) were infiltrated into leaves of the indicated genotypes. The bacterial number was measured at 0 dpi and 2 dpi. Data were obtained in at least four independent experiments each with at least 4 or 8 biological replicates for 0 dpi or 2 dpi, respectively. Bars represent means and standard errors determined by a mixed linear model. For *P*-values in all the pairwise comparisons, see Table S2 (two-tailed t-tests). (B) AvrRpt2-ETI was calculated by subtracting bacterial number in *Pto* DC3000 AvrRpt2 -inoculated plants from that in *Pto* DC3000 EV -inoculated plants. Bars represent means and standard errors determined by a mixed linear model. For *P*-values in all the pairwise comparisons, see Table S3 (two-tailed t-tests).
(0.09 MB PDF)

Figure S5 AvrRpm1-ETI in all single, double, triple and quadruple mutants. (A) *Pto* DC3000 EV or *Pto* DC3000 AvrRpm1 (OD_600_ = 0.0001) were infiltrated into plants. Bacterial number was measured at 0 dpi and 2dpi. Data were obtained in at least four independent experiments each with at least 4 or 8 biological replicates for 0 dpi or 2 dpi, respectively. Bars represent means and standard errors determined by a mixed linear model. For *P*-values in all the pairwise comparisons, see Table S4 (two-tailed *t*-tests). (B) AvrRpm1-ETI was calculated by subtracting bacterial number in *Pto* DC3000 (AvrRpm1)-inoculated plants from that in *Pto* DC3000 (EV)-inoculated plants. Bars represent means and standard errors determined by a mixed linear model. For *P*-values in all the pairwise comparisons, see Table S5 (two-tailed *t*-tests).
(0.09 MB PDF)

Figure S6 Flg22-PTI to *P. syringae* in all single, double, triple and quadruple mutants. (A) *Pto* DC3000 (OD_600_ = 0.0001) was infiltrated into plants one day after treatment with water (mock) or 1 mM flg22 (flg22). The bacterial number (cfu/cm^2^) was measured at 0 dpi and 2 dpi. Data were obtained in at least three independent experiments each with at least 4 or 12 biological replicates for 0 dpi or 2 dpi, respectively. Bars represent means and standard errors determined by a mixed linear model. For the *P*-values in all the pairwise comparisons, see Table S6 (two-tailed *t*-tests). (B) Flg22-PTI was calculated by subtracting bacterial number in flg22-treated plants from that in mock-treated plants. Bars represent means and standard errors determined by a mixed linear model. For *P*-values in all the pairwise comparisons, see Table S7 (two-tailed *t*-tests).
(0.09 MB PDF)

Figure S7 Elf18-PTI to *P. syringae* in all single, double, triple and quadruple mutants. (A) *Pto* DC3000 (OD_600_ = 0.0001) was infiltrated into plants one day after treatment with water (mock) or 1 mM elf18 (elf18). The bacterial number (cfu/cm^2^) was measured at 0 dpi and 2 dpi. Data were obtained in four independent experiments each with 4 or 8 biological replicates for 0 dpi or 2 dpi, respectively. Bars represent means and standard errors determined by a mixed linear model. For *P*-values in all the pairwise comparisons, see Table S8 (two-tailed *t*-tests). (B) Elf18-PTI was calculated by subtracting bacterial number in elf18-treated plants from that in mock-treated plants. Bars represent means and standard errors determined by a mixed linear model. For *P*-values in all the pairwise comparisons, see Table S9 (two-tailed *t*-tests).
(0.09 MB PDF)

Figure S8 Immunity against *A. brassicicola* in all single, double, triple and the quadruple mutants. DNA was extracted from inoculated leaves 0 dpi and 3 dpi. The log_2_ ratio of copy number between a fungal gene (*CutinA.1*) and a plant gene (*iASK*) was determined by qPCR and used as the disease index. Each sample consists of 6 to 8 leaves or 16 to 18 for 0 dpi or 3 dpi, respectively. Data were obtained in at least five independent experiments. Bars represent means and standard errors determined by a mixed linear model. For *P*-values in all the pairwise comparisons, see Table S10 (two-tailed *t*-tests).
(0.08 MB PDF)

Table S1 Primers and restriction enzymes used for genotyping.
(0.03 MB DOC)

Table S2 *P*-values for all comparisons in Figure S4A.
(0.02 MB PDF)

Table S3 *P*-values for all comparisons in Figure S4B.
(0.01 MB PDF)

Table S4 *P*-values for all comparisons in Figure S5A.
(0.02 MB PDF)

Table S5 *P*-values for all comparisons in Figure S5B.
(0.01 MB PDF)

Table S6 *P*-values for all comparisons in Figure S6A.
(0.02 MB PDF)

Table S7 *P*-values for all comparisons in Figure S6B.
(0.01 MB PDF)

Table S8 *P*-values for all comparisons in Figure S7A.
(0.02 MB PDF)

Table S9 *P*-values for all comparisons in Figure S7B.
(0.01 MB PDF)

Table S10 *P*-values for all comparisons in Figure S8.
(0.02 MB PDF)

Text S1 Information for Table 1.
(0.04 MB DOC)

Text S2 Supporting materials and methods.
(0.04 MB DOC)

## References

[1] Jones JD, Dangl JL (2006). The plant immune system.. Nature.

[2] Chisholm ST, Coaker G, Day B, Staskawicz BJ (2006). Host-microbe interactions: Shaping the evolution of the plant immune response.. Cell.

[3] Abramovitch RB, Anderson JC, Martin GB (2006). Bacterial elicitation and evasion of plant innate immunity.. Nat Rev Mol Cell Biol.

[4] Zipfel C, Robatzek S, Navarro L, Oakeley EJ, Jones JD (2004). Bacterial disease resistance in *Arabidopsis* through flagellin perception.. Nature.

[5] Zipfel C, Kunze G, Chinchilla D, Caniard A, Jones JD (2006). Perception of the bacterial PAMP EF-tu by the receptor EFR restricts *Agrobacterium*-mediated transformation.. Cell.

[6] Mindrinos M, Katagiri F, Yu GL, Ausubel FM (1994). The A. thaliana disease resistance gene *RPS2* encodes a protein containing a nucleotide-binding site and leucine-rich repeats.. Cell.

[7] Bent AF, Kunkel BN, Dahlbeck D, Brown KL, Schmidt R (1994). *RPS2* of *Arabidopsis thaliana*: A leucine-rich repeat class of plant disease resistance genes.. Science.

[8] Grant MR, Godiard L, Straube E, Ashfield T, Lewald J (1995). Structure of the *Arabidopsis RPM1* gene enabling dual specificity disease resistance.. Science.

[9] Shao F, Golstein C, Ade J, Stoutemyer M, Dixon JE (2003). Cleavage of *Arabidopsis* PBS1 by a bacterial type III effector.. Science.

[10] Tao Y, Xie Z, Chen W, Glazebrook J, Chang HS (2003). Quantitative nature of arabidopsis responses during compatible and incompatible interactions with the bacterial pathogen *Pseudomonas syringae*.. Plant Cell.

[11] Tsuda K, Sato M, Glazebrook J, Cohen JD, Katagiri F (2008). Interplay between MAMP-triggered and SA-mediated defense responses.. Plant J.

[12] Navarro L, Jay F, Nomura K, He SY, Voinnet O (2008). Suppression of the microRNA pathway by bacterial effector proteins.. Science.

[13] Zhang J, Shao F, Li Y, Cui H, Chen L (2007). A *Pseudomonas syringae* effector inactivates MAPKs to suppress PAMP-induced immunity in plants.. Cell Host Microbe.

[14] Fu ZQ, Guo M, Jeong BR, Tian F, Elthon TE (2007). A type III effector ADP-ribosylates RNA-binding proteins and quells plant immunity.. Nature.

[15] Nomura K, Debroy S, Lee YH, Pumplin N, Jones J (2006). A bacterial virulence protein suppresses host innate immunity to cause plant disease.. Science.

[16] Rosebrock TR, Zeng L, Brady JJ, Abramovitch RB, Xiao F (2007). A bacterial E3 ubiquitin ligase targets a host protein kinase to disrupt plant immunity.. Nature.

[17] Glazebrook J (2005). Contrasting mechanisms of defense against biotrophic and necrotrophic pathogens.. Annu Rev Phytopathol.

[18] Spoel SH, Johnson JS, Dong X (2007). Regulation of tradeoffs between plant defenses against pathogens with different lifestyles.. Proc Natl Acad Sci U S A.

[19] Brooks DM, Bender CL, Kunkel BN (2005). The *Pseudomonas syringae* phytotoxin coronatine promotes virulence by overcoming salicylic acid-dependent defences in *Arabidopsis thaliana*.. Mol Plant Pathol.

[20] Bender CL, Malvick DK, Mitchell RE (1989). Plasmid-mediated production of the phytotoxin coronatine in *Pseudomonas syringae* pv. *tomato*.. J Bacteriol.

[21] Thines B, Katsir L, Melotto M, Niu Y, Mandaokar A (2007). JAZ repressor proteins are targets of the SCF(COI1) complex during jasmonate signalling.. Nature.

[22] Park JH, Halitschke R, Kim HB, Baldwin IT, Feldmann KA (2002). A knock-out mutation in allene oxide synthase results in male sterility and defective wound signal transduction in *Arabidopsis* due to a block in jasmonic acid biosynthesis.. Plant J.

[23] Alonso JM, Hirayama T, Roman G, Nourizadeh S, Ecker JR (1999). EIN2, a bifunctional transducer of ethylene and stress responses in *Arabidopsis*.. Science.

[24] Wildermuth MC, Dewdney J, Wu G, Ausubel FM (2001). Isochorismate synthase is required to synthesize salicylic acid for plant defence.. Nature.

[25] Jirage D, Tootle TL, Reuber TL, Frost LN, Feys BJ (1999). *Arabidopsis thaliana* PAD4 encodes a lipase-like gene that is important for salicylic acid signaling.. Proc Natl Acad Sci U S A.

[26] Glazebrook J, Chen W, Estes B, Chang HS, Nawrath C (2003). Topology of the network integrating salicylate and jasmonate signal transduction derived from global expression phenotyping.. Plant J.

[27] Belkhadir Y, Subramaniam R, Dangl JL (2004). Plant disease resistance protein signaling: NBS-LRR proteins and their partners.. Curr Opin Plant Biol.

[28] Kim MG, da Cunha L, McFall AJ, Belkhadir Y, DebRoy S (2005). Two *Pseudomonas syringae* type III effectors inhibit RIN4-regulated basal defense in *Arabidopsis*.. Cell.

[29] Lamb CJ, Lawton MA, Dron M, Dixon RA (1989). Signals and transduction mechanisms for activation of plant defenses against microbial attack.. Cell.

[30] Gomez-Gomez L, Felix G, Boller T (1999). A single locus determines sensitivity to bacterial flagellin in *Arabidopsis thaliana*.. Plant J.

[31] Tsuda K, Glazebrook J, Katagiri F (2008). The interplay between MAMP and SA signaling.. Plant Signal Behav.

[32] Kunze G, Zipfel C, Robatzek S, Niehaus K, Boller T (2004). The N terminus of bacterial elongation factor tu elicits innate immunity in Arabidopsis plants.. Plant Cell.

[33] Chinchilla D, Zipfel C, Robatzek S, Kemmerling B, Nurnberger T (2007). A flagellin-induced complex of the receptor FLS2 and BAK1 initiates plant defence.. Nature.

[34] Miya A, Albert P, Shinya T, Desaki Y, Ichimura K (2007). CERK1, a LysM receptor kinase, is essential for chitin elicitor signaling in *Arabidopsis*.. Proc Natl Acad Sci U S A.

[35] Wan J, Zhang XC, Neece D, Ramonell KM, Clough S (2008). A LysM receptor-like kinase plays a critical role in chitin signaling and fungal resistance in *Arabidopsis*.. Plant Cell.

[36] Thomma BP, Eggermont K, Penninckx IA, Mauch-Mani B, Vogelsang R (1998). Separate jasmonate-dependent and salicylate-dependent defense-response pathways in arabidopsis are essential for resistance to distinct microbial pathogens.. Proc Natl Acad Sci U S A.

[37] Shah J (2003). The salicylic acid loop in plant defense.. Curr Opin Plant Biol.

[38] Ritter C, Dangl JL (1996). Interference between two specific pathogen recognition events mediated by distinct plant disease resistance genes.. Plant Cell.

[39] Bar-Yam Y, Harmon D, de Bivort B (2009). Systems biology. attractors and democratic dynamics.. Science.

[40] McDowell JM, Cuzick A, Can C, Beynon J, Dangl JL (2000). Downy mildew (*Peronospora parasitica*) resistance genes in Arabidopsis vary in functional requirements for *NDR1*, *EDS1*, *NPR1* and salicylic acid accumulation.. Plant J 2000.

[41] Asai T, Tena G, Plotnikova J, Willmann MR, Chiu WL (2002). MAP kinase signalling cascade in *Arabidopsis* innate immunity.. Nature.

[42] Navarro L, Bari R, Achard P, Lison P, Nemri A (2008). DELLAs control plant immune responses by modulating the balance of jasmonic acid and salicylic acid signaling.. Curr Biol.

[43] Adie BA, Perez-Perez J, Perez-Perez MM, Godoy M, Sanchez-Serrano JJ (2007). ABA is an essential signal for plant resistance to pathogens affecting JA biosynthesis and the activation of defenses in *Arabidopsis*.. Plant Cell.

[44] Navarro L, Dunoyer P, Jay F, Arnold B, Dharmasiri N (2006). A plant miRNA contributes to antibacterial resistance by repressing auxin signaling.. Science.

[45] Torres MA, Dangl JL, Jones JD (2002). *Arabidopsis* gp91phox homologues *AtrbohD* and *AtrbohF* are required for accumulation of reactive oxygen intermediates in the plant defense response.. Proc Natl Acad Sci U S A.

[46] von Malek B, van der Graaff E, Schneitz K, Keller B (2002). The *Arabidopsis* male-sterile mutant *dde2-2* is defective in the *ALLENE OXIDE SYNTHASE* gene encoding one of the key enzymes of the jasmonic acid biosynthesis pathway.. Planta.

[47] Wang H, Ngwenyama N, Liu Y, Walker JC, Zhang S (2007). Stomatal development and patterning are regulated by environmentally responsive mitogen-activated protein kinases in *Arabidopsis*.. Plant Cell.

[48] Liu Y, Zhang S (2004). Phosphorylation of 1-aminocyclopropane-1-carboxylic acid synthase by MPK6, a stress-responsive mitogen-activated protein kinase, induces ethylene biosynthesis in Arabidopsis.. Plant Cell.

[49] Cao H, Glazebrook J, Clarke JD, Volko S, Dong X (1997). The Arabidopsis *NPR1* gene that controls systemic acquired resistance encodes a novel protein containing ankyrin repeats.. Cell.

[50] Zhou N, Tootle TL, Tsui F, Klessig DF, Glazebrook J (1998). *PAD4* functions upstream from salicylic acid to control defense responses in arabidopsis.. Plant Cell.

[51] Nishimura MT, Stein M, Hou BH, Vogel JP, Edwards H (2003). Loss of a callose synthase results in salicylic acid-dependent disease resistance.. Science.

[52] Mackey D, Belkhadir Y, Alonso JM, Ecker JR, Dangl JL (2003). *Arabidopsis* RIN4 is a target of the type III virulence effector AvrRpt2 and modulates RPS2-mediated resistance.. Cell.

[53] Qi Y, Katagiri F (2009). Purification of low-abundance Arabidopsis plasma-membrane protein complexes and identification of candidate components.. Plant J.

[54] Lee JS, Ellis BE (2007). *Arabidopsis* MAPK phosphatase 2 (MKP2) positively regulates oxidative stress tolerance and inactivates the MPK3 and MPK6 MAPKs.. J Biol Chem.

[55] Suarez-Rodriguez MC, Adams-Phillips L, Liu Y, Wang H, Su SH (2007). MEKK1 is required for flg22-induced MPK4 activation in Arabidopsis plants.. Plant Physiol.

[56] Gachon C, Saindrenan P (2004). Real-time PCR monitoring of fungal development in *Arabidopsis thaliana* infected by *Alternaria brassicicola* and *Botrytis cinerea*.. Plant Physiol Biochem.

[57] Glazebrook J, Ausubel FM (1994). Isolation of phytoalexin-deficient mutants of *Arabidopsis thaliana* and characterization of their interactions with bacterial pathogens.. Proc Natl Acad Sci U S A.

[58] Schuhegger R, Nafisi M, Mansourova M, Petersen BL, Olsen CE (2006). CYP71B15 (PAD3) catalyzes the final step in camalexin biosynthesis.. Plant Physiol.

