# Supplementary figures and images for: Network Properties of Robust Immunity in Plants
Source: PLoS Genet. 2009 Dec 11;5(12):e1000772. doi: 10.1371/journal.pgen.1000772 (PMC2782137; doi:10.1371/journal.pgen.1000772)

Supporting Information Figure S1

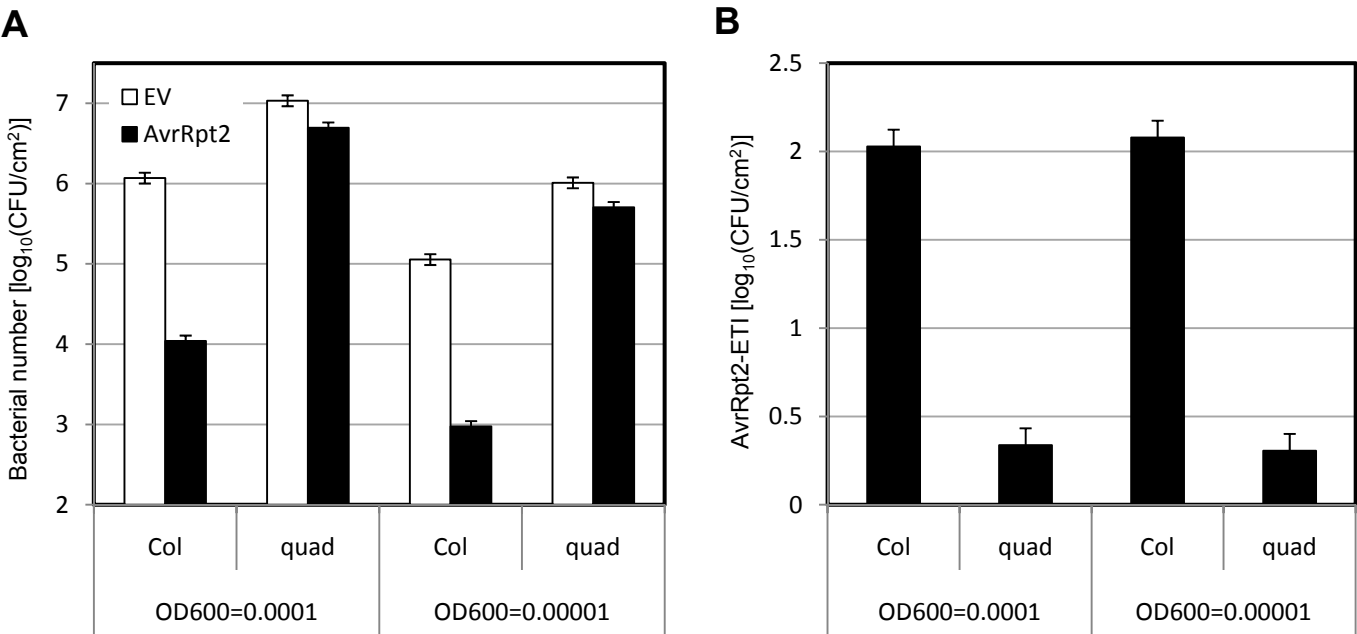

Supplement: Figure S1 — Comparable AvrRpt2-ETI using a ten times lower dose of Pto DC3000 derivatives. (A) Pto DC3000 EV or Pto DC3000 AvrRpt2 (OD600 = 0.0001 or OD600 = 0.00001) were infiltrated into plants. The bacterial number was measured at 2 dpi. Data were obtained in two independent experiments each with 16 biological replicates. Bars represent means and standard errors determined by a mixed linear model. (B) AvrRpt2-ETI was calculated by subtracting bacterial number in Pto DC3000 AvrRpt2-inoculated plants from that in Pto DC3000 EV-inoculated plants. Bars represent means and standard errors determined by a mixed linear model. (0.07 MB PDF) [file pgen.1000772.s001.pdf]

Supporting Information Figure S3

A

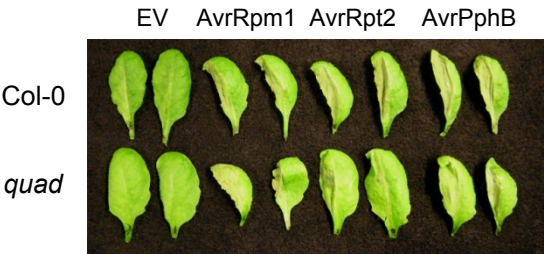

B

|         | Col-0 | quad  |
|---------|-------|-------|
| EV      | 0/15  | 0/16  |
| AvrRpm1 | 15/15 | 15/15 |
| AvrRpt2 | 16/16 | 1/16  |
| AvrPphB | 15/15 | 9/15  |

C

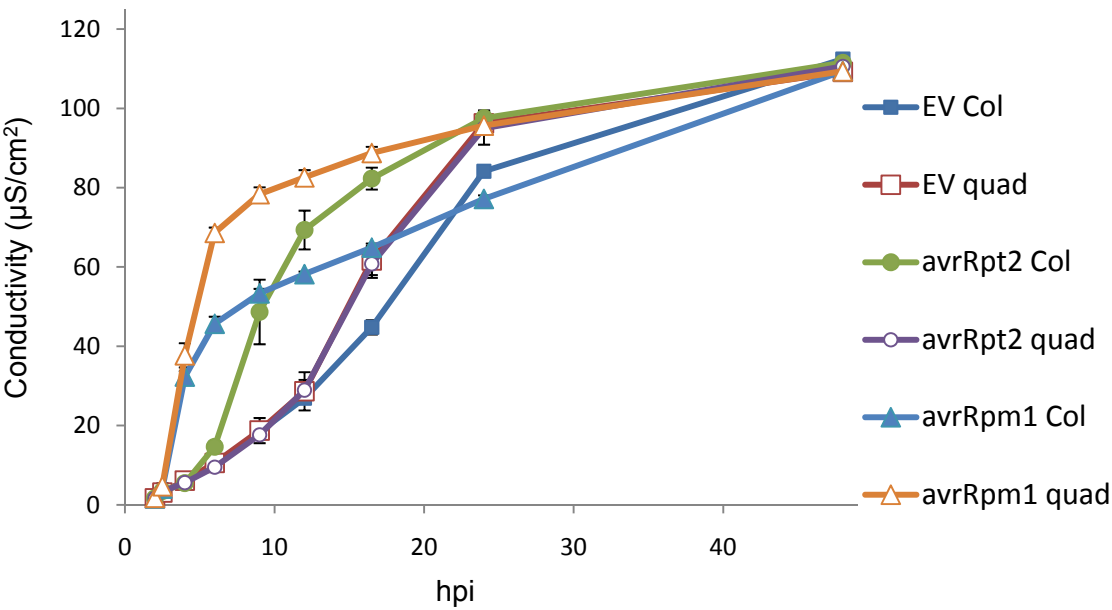

Supplement: Figure S3 — The hypersensitive response (HR) triggered by AvrRpt2 was compromised in the quadruple mutant. (A) Pto DC3000 EV, Pto DC3000 AvrRpm1, Pto DC3000 AvrRpt2 or Pto DC3000 AvrPphB (OD600 = 0.05) were infiltrated into the left halves of the leaves of either Col-0 or the quadruple mutant. Representative leaves at 24 hpi are shown. (B) The number of leaves that showed a macroscopic HR/the total number of leaves infiltrated. (C) Electrolyte leakage measurements following inoculation with Pto DC3000 EV, Pto DC3000 AvrRpm1 or Pto DC3000 AvrRpt2 (OD600 = 0.1). Each sample consisted of four leaf discs from two leaves. Two independent experiments were performed with three biological replicates per treatment per experiment. The data from two experiments were combined, and means and standard errors were calculated. (0.19 MB PDF) [file pgen.1000772.s003.pdf]

Supporting Information Figure S4

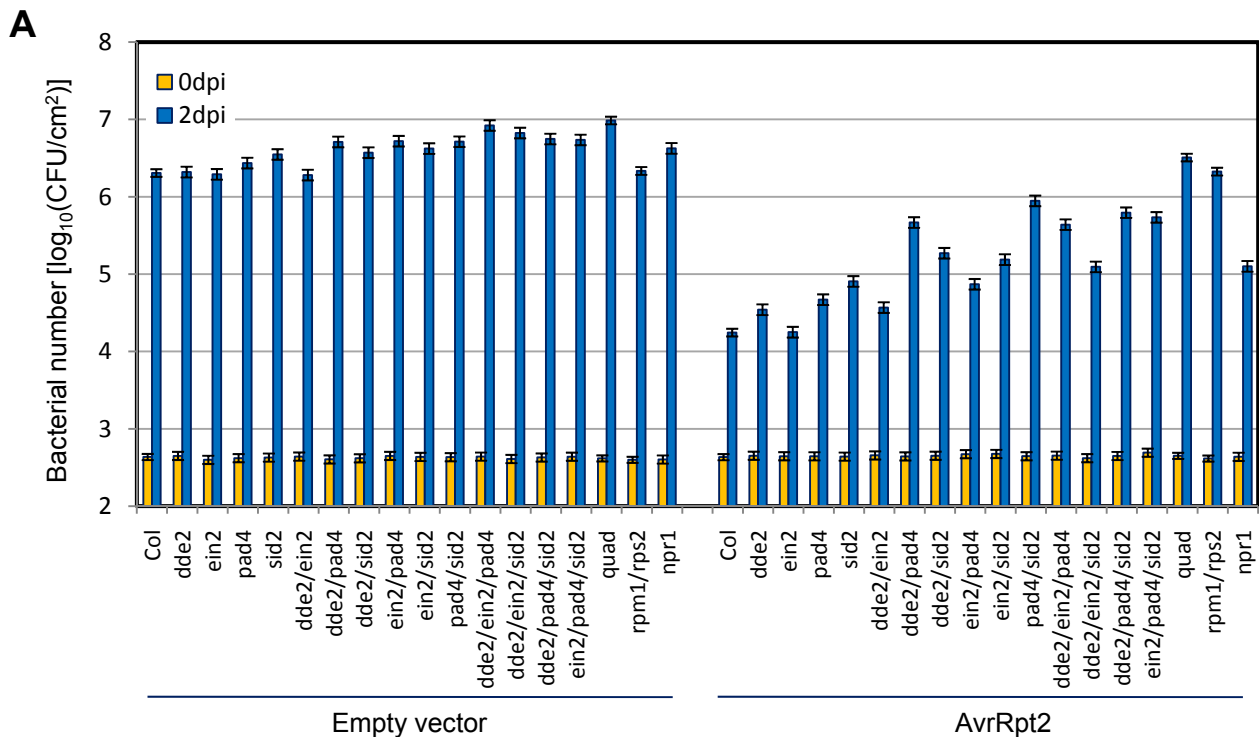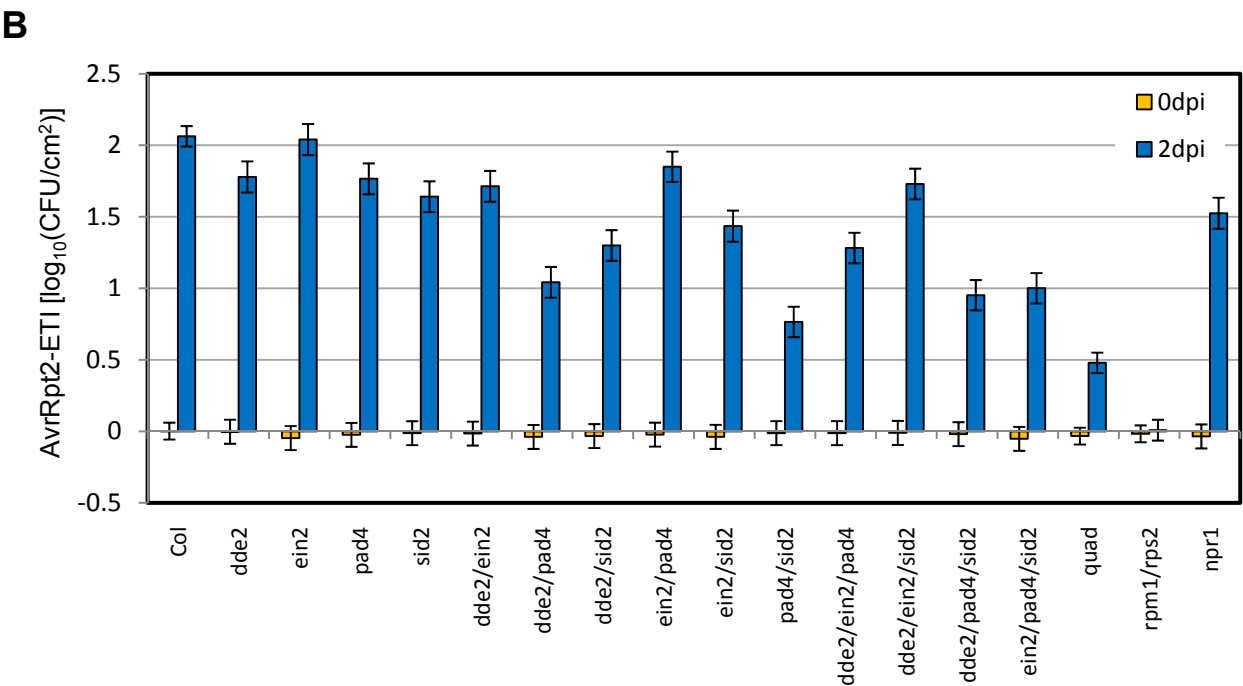

Supplement: Figure S4 — AvrRpt2-ETI in all single, double, triple and the quadruple mutants. (A) Pto DC3000 EV or Pto DC3000 AvrRpt2 (OD600 = 0.0001) were infiltrated into leaves of the indicated genotypes. The bacterial number was measured at 0 dpi and 2 dpi. Data were obtained in at least four independent experiments each with at least 4 or 8 biological replicates for 0 dpi or 2 dpi, respectively. Bars represent means and standard errors determined by a mixed linear model. For P-values in all the pairwise comparisons, see Table S2 (two-tailed t-tests). (B) AvrRpt2-ETI was calculated by subtracting bacterial number in Pto DC3000 AvrRpt2 -inoculated plants from that in Pto DC3000 EV -inoculated plants. Bars represent means and standard errors determined by a mixed linear model. For P-values in all the pairwise comparisons, see Table S3 (two-tailed t-tests). (0.09 MB PDF) [file pgen.1000772.s004.pdf]

Supporting Information Figure S5

A

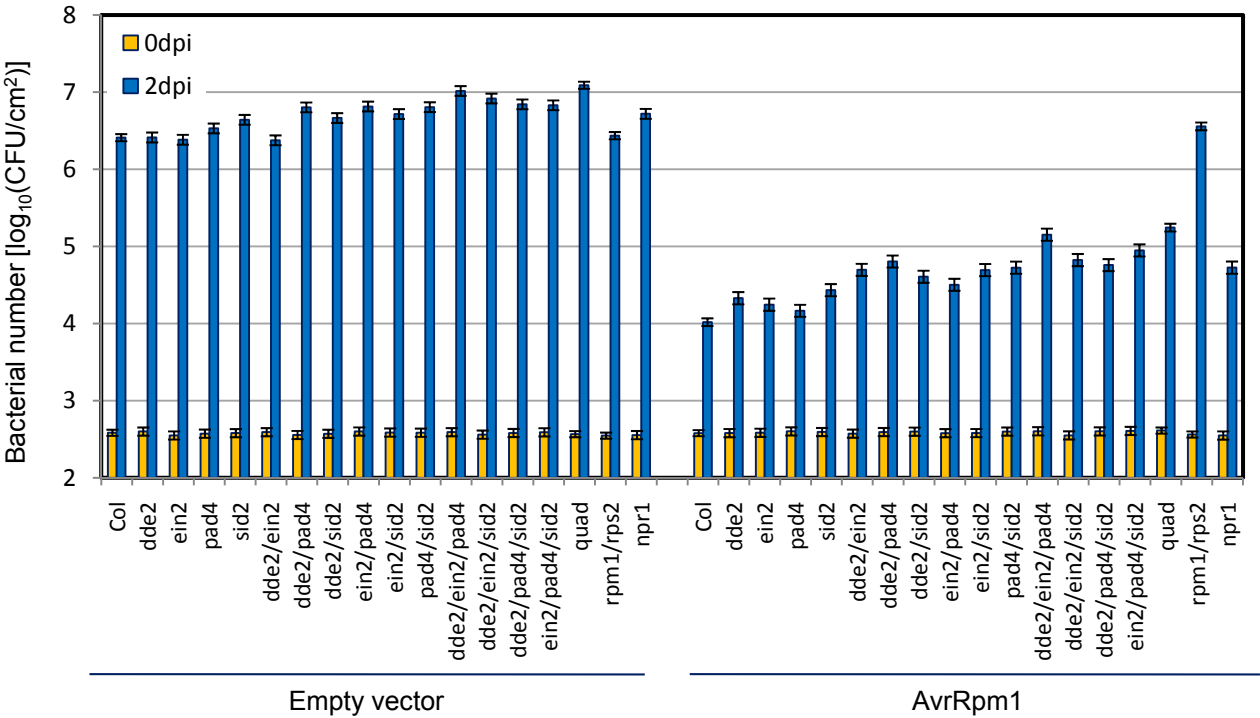

B

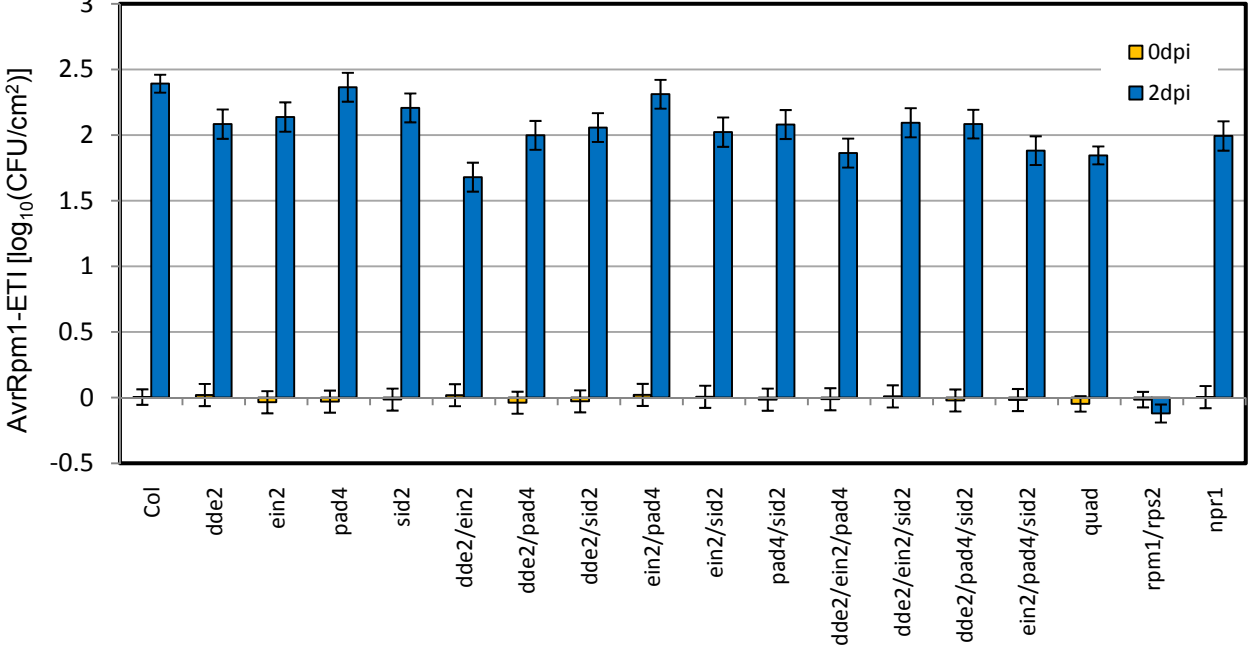

Supplement: Figure S5 — AvrRpm1-ETI in all single, double, triple and quadruple mutants. (A) Pto DC3000 EV or Pto DC3000 AvrRpm1 (OD600 = 0.0001) were infiltrated into plants. Bacterial number was measured at 0 dpi and 2dpi. Data were obtained in at least four independent experiments each with at least 4 or 8 biological replicates for 0 dpi or 2 dpi, respectively. Bars represent means and standard errors determined by a mixed linear model. For P-values in all the pairwise comparisons, see Table S4 (two-tailed t-tests). (B) AvrRpm1-ETI was calculated by subtracting bacterial number in Pto DC3000 (AvrRpm1)-inoculated plants from that in Pto DC3000 (EV)-inoculated plants. Bars represent means and standard errors determined by a mixed linear model. For P-values in all the pairwise comparisons, see Table S5 (two-tailed t-tests). (0.09 MB PDF) [file pgen.1000772.s005.pdf]

Supporting Information Figure S6

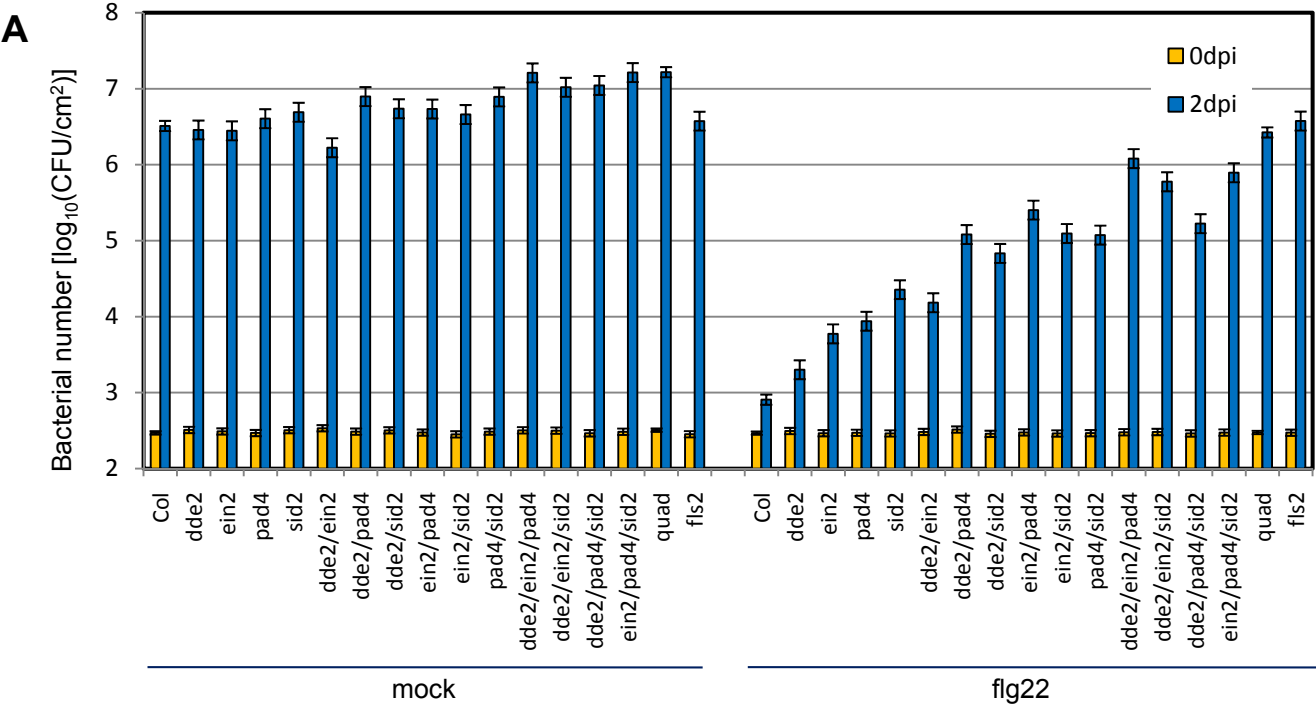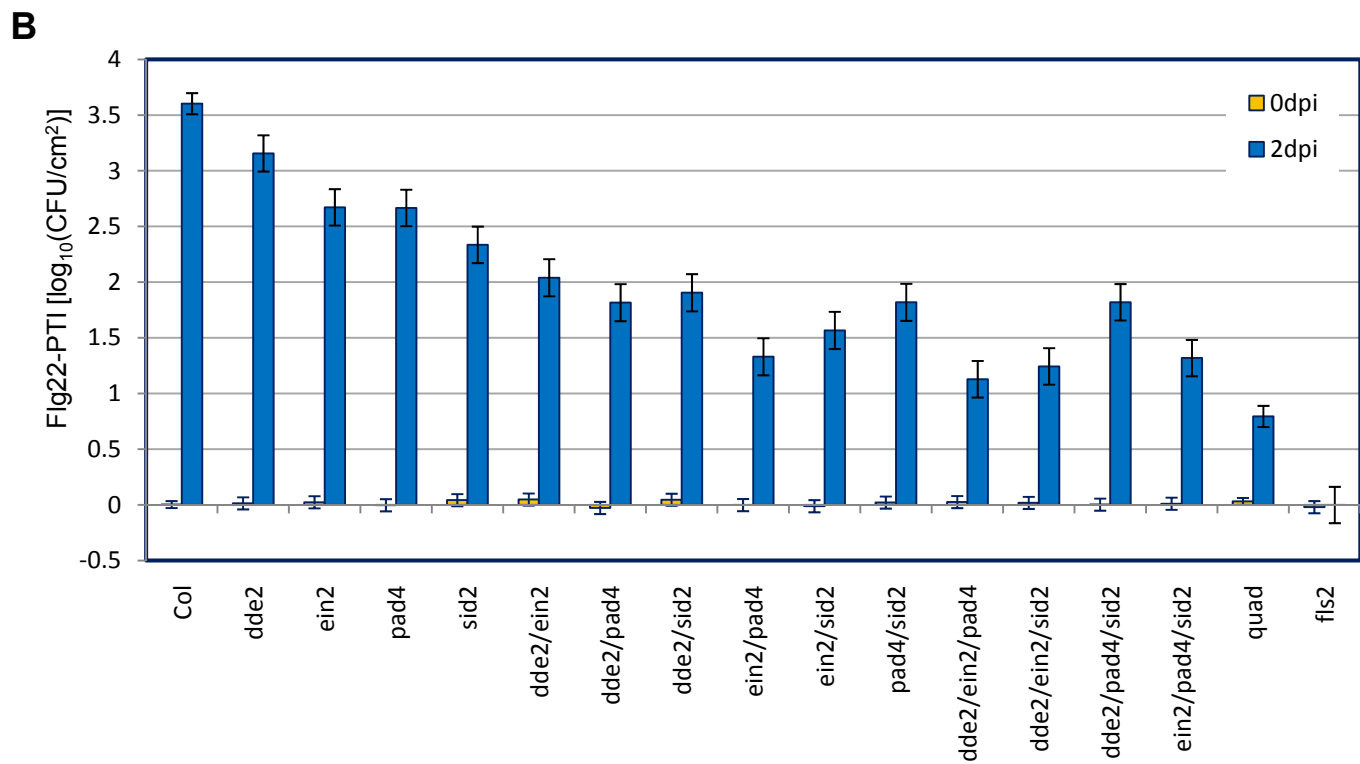

Supplement: Figure S6 — Flg22-PTI to P. syringae in all single, double, triple and quadruple mutants. (A) Pto DC3000 (OD600 = 0.0001) was infiltrated into plants one day after treatment with water (mock) or 1 mM flg22 (flg22). The bacterial number (cfu/cm2) was measured at 0 dpi and 2 dpi. Data were obtained in at least three independent experiments each with at least 4 or 12 biological replicates for 0 dpi or 2 dpi, respectively. Bars represent means and standard errors determined by a mixed linear model. For the P-values in all the pairwise comparisons, see Table S6 (two-tailed t-tests). (B) Flg22-PTI was calculated by subtracting bacterial number in flg22-treated plants from that in mock-treated plants. Bars represent means and standard errors determined by a mixed linear model. For P-values in all the pairwise comparisons, see Table S7 (two-tailed t-tests). (0.09 MB PDF) [file pgen.1000772.s006.pdf]

Supporting Information Figure S7

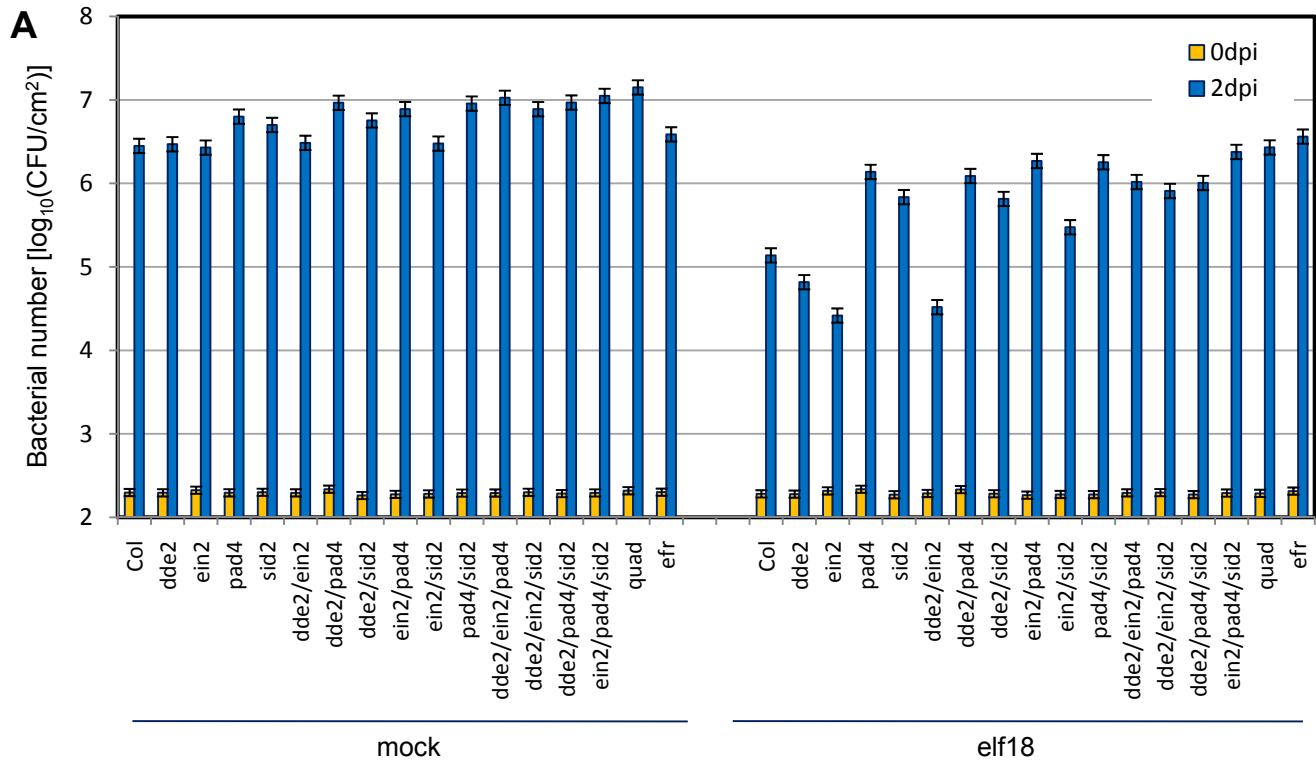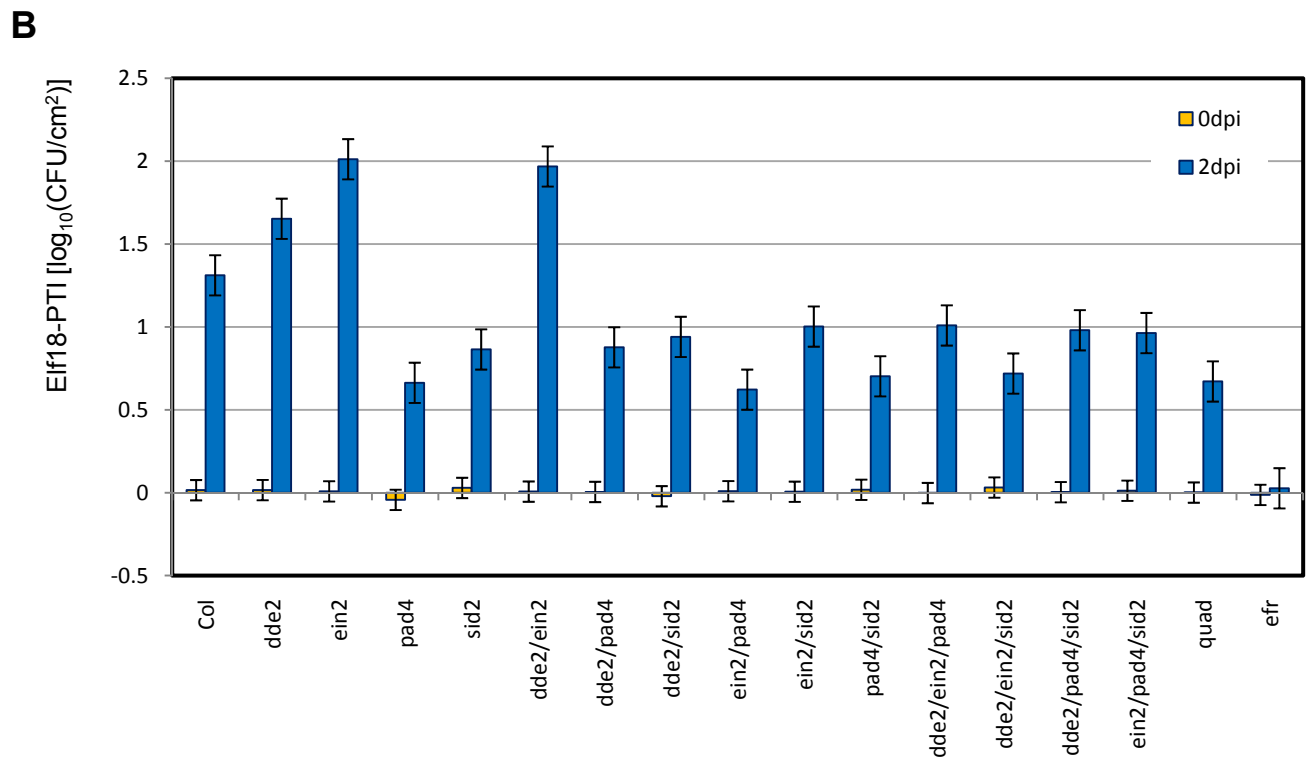

Supplement: Figure S7 — Elf18-PTI to P. syringae in all single, double, triple and quadruple mutants. (A) Pto DC3000 (OD600 = 0.0001) was infiltrated into plants one day after treatment with water (mock) or 1 mM elf18 (elf18). The bacterial number (cfu/cm2) was measured at 0 dpi and 2 dpi. Data were obtained in four independent experiments each with 4 or 8 biological replicates for 0 dpi or 2 dpi, respectively. Bars represent means and standard errors determined by a mixed linear model. For P-values in all the pairwise comparisons, see Table S8 (two-tailed t-tests). (B) Elf18-PTI was calculated by subtracting bacterial number in elf18-treated plants from that in mock-treated plants. Bars represent means and standard errors determined by a mixed linear model. For P-values in all the pairwise comparisons, see Table S9 (two-tailed t-tests). (0.09 MB PDF) [file pgen.1000772.s007.pdf]

Supporting Information Figure S8

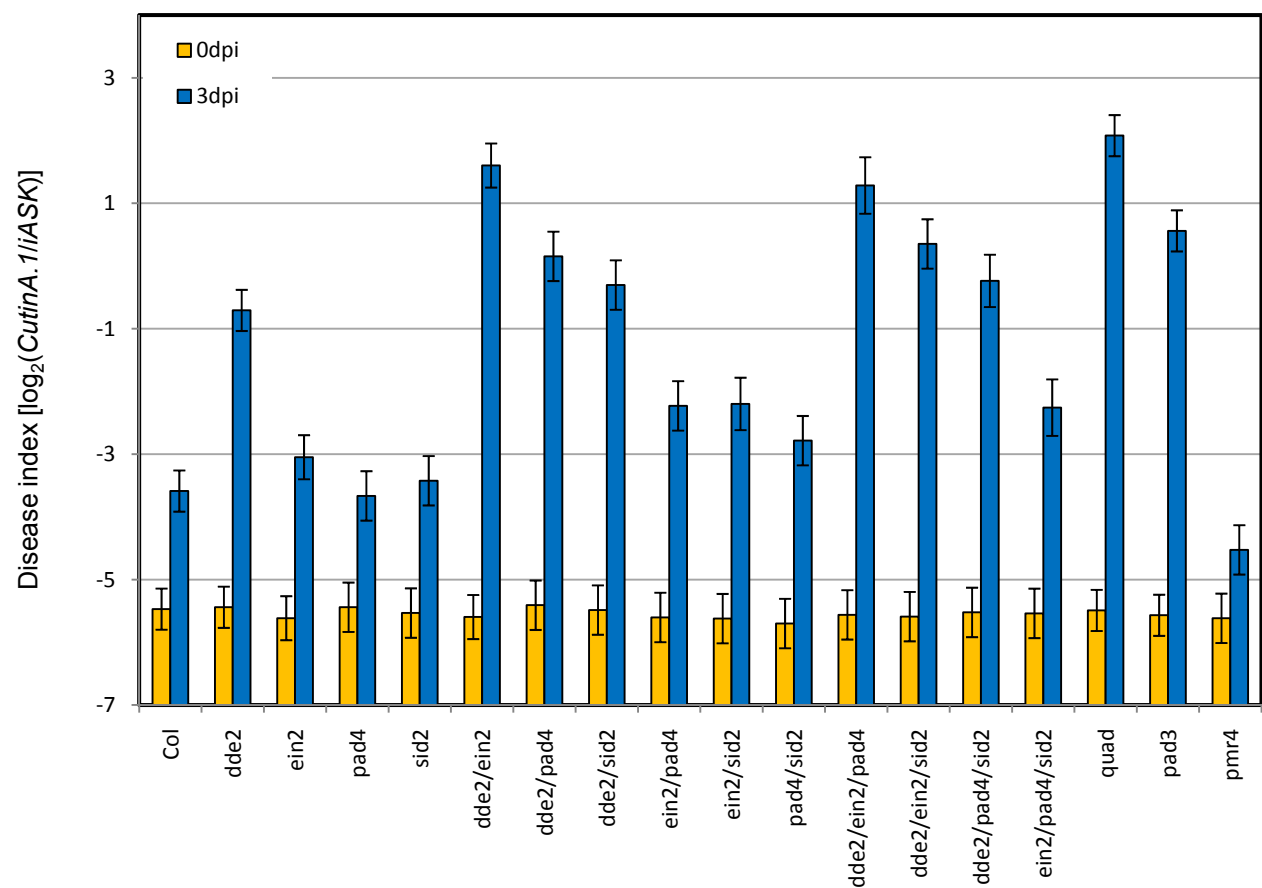

Supplement: Figure S8 — Immunity against A. brassicicola in all single, double, triple and the quadruple mutants. DNA was extracted from inoculated leaves 0 dpi and 3 dpi. The log2 ratio of copy number between a fungal gene (CutinA.1) and a plant gene (iASK) was determined by qPCR and used as the disease index. Each sample consists of 6 to 8 leaves or 16 to 18 for 0 dpi or 3 dpi, respectively. Data were obtained in at least five independent experiments. Bars represent means and standard errors determined by a mixed linear model. For P-values in all the pairwise comparisons, see Table S10 (two-tailed t-tests). (0.08 MB PDF) [file pgen.1000772.s008.pdf]
