# Supplementary material for: Network Properties of Robust Immunity in Plants
Source: PLoS Genet. 2009 Dec 11;5(12):e1000772. doi: 10.1371/journal.pgen.1000772 (PMC2782137; doi:10.1371/journal.pgen.1000772)
Supplement: Figure S2 — AvrPphB-ETI in the quadruple mutant. (A) Pto DC3000 EV or Pto DC3000 AvrPphB (OD600 = 0.0001) were infiltrated into plants. Bacterial number was measured at 0 dpi and 2 dpi. Data were obtained in two independent experiments each with 4 or 16 biological replicates for 0 dpi or 2 dpi, respectively. Bars represent means and standard error determined by a mixed linear model. Arrows indicate AvrPphB-ETI. (B) AvrPphB-ETI was calculated by subtracting bacterial number in Pto DC3000 (AvrPphB)-inoculated plants from that in Pto DC3000 (EV)-inoculated plants. Two-tailed t-tests were used for P-values. (0.09 MB PDF) [file pgen.1000772.s002.pdf]

Supporting Information Figure S2

A

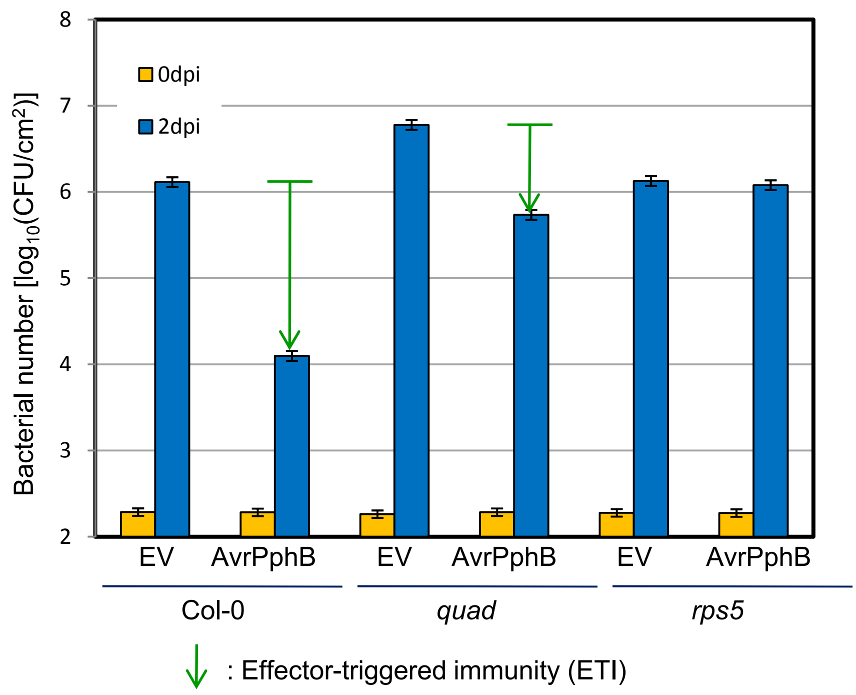

B

**Genotype**	**ETI [$\log_{10}$(CFU/cm^2)]**	***P*-value**
Col-0	2.02	---------
quad	1.04	< $1X10^{-13}$
rps5	0.05	< $1X10^{-36}$
