# Supplementary material for: Network Properties of Robust Immunity in Plants
Source: PLoS Genet. 2009 Dec 11;5(12):e1000772. doi: 10.1371/journal.pgen.1000772 (PMC2782137; doi:10.1371/journal.pgen.1000772)
Supplement: Table S1 — Primers and restriction enzymes used for genotyping. (0.03 MB DOC) [file pgen.1000772.s009.doc]

**Table S1** Primers and restriction enzymes used for genotyping.

|  | Primer (5' to 3') | |  |
| --- | --- | --- | --- |
| mutation | Forward | Reverse | PCR product size |
| *dde2-2* | GACACGAACCGGATCCAAAG | GCCGAAATCCGCTTTCCCTTTA | 498 bp |
| *ein2-1* | TGCGAGTAACAGAGCGGAAG | TACATCAGAGTCTTCTTTAAGACTAC | 310 bp |
| *pad4-1* | GCGATGCATCAGAAGAG | TTAGCCCAAAAGCAAGTATC | 391 bp |
| *sid2-2* | TTCTTCATGCAGGGGAGGAG | AAGCAAAATGTTTGAGTCAGCA | 879 bp (WT) 581 bp (*sid2-2*) |
|  | CAACCACCTGGTGCACCAGC |  |  |

| mutation | Restriction Enzyme | fragments in WT (bp) | fragments in mutant (bp) |
| --- | --- | --- | --- |
| *dde2-2* | *Bst*UI | 498 bp | 248 and 250 bp |
| *ein2-1* | *Afl*II | 310 bp | 32 and 278 bp |
| *pad4-1* | *Bsm*F1 | 110 and 281 bp | 391 bp |
