# Supplementary material for: Network Properties of Robust Immunity in Plants
Source: PLoS Genet. 2009 Dec 11;5(12):e1000772. doi: 10.1371/journal.pgen.1000772 (PMC2782137; doi:10.1371/journal.pgen.1000772)
Supplement: Table S2 — P-values for all comparisons in Figure S4A. (0.02 MB PDF) [file pgen.1000772.s010.pdf]

Table S2

Comparisons	0dpi	2dpi
_Col:_pLAFR:dde2:_pLAFR	0.81716	0.87445
_Col:_pLAFR:dde2/ein2:_pLAFR	0.92914	0.73592
_Col:_pLAFR:dde2/ein2/pad4:_pLAFR	0.9358	2.6E-15
_Col:_pLAFR:dde2/ein2/pad4/sid2:_pLAFR	0.70944	1.5E-27
_Col:_pLAFR:dde2/ein2/sid2:_pLAFR	0.66073	2.2E-11
_Col:_pLAFR:dde2/pad4:_pLAFR	0.59702	2.8E-07
_Col:_pLAFR:dde2/pad4/sid2:_pLAFR	0.91461	7.6E-09
_Col:_pLAFR:dde2/sid2:_pLAFR	0.77394	0.00068
_Col:_pLAFR:ein2:_pLAFR	0.53048	0.82773
_Col:_pLAFR:ein2/pad4:_pLAFR	0.81474	5.4E-08
_Col:_pLAFR:ein2/pad4/sid2:_pLAFR	0.95249	1.6E-08
_Col:_pLAFR:ein2/sid2:_pLAFR	0.9871	6E-05
_Col:_pLAFR:npr1:_pLAFR	0.5802	5.2E-05
_Col:_pLAFR:pad4:_pLAFR	0.79679	0.09775
_Col:_pLAFR:pad4/sid2:_pLAFR	0.96908	1.6E-07
_Col:_pLAFR:rpm1/rps2:_pLAFR	0.45471	0.67225
_Col:_pLAFR:sid2:_pLAFR	0.89139	0.00197
_Col:_pLAFR:_Col:avrRpt2	0.97259	3E-145
_Col:_pLAFR:dde2:avrRpt2	0.82661	4.4E-73
_Col:_pLAFR:dde2/ein2:avrRpt2	0.77659	5.9E-72
_Col:_pLAFR:dde2/ein2/pad4:avrRpt2	0.81484	3.5E-13
_Col:_pLAFR:dde2/ein2/pad4/sid2:avrRpt2	0.80537	0.0052
_Col:_pLAFR:dde2/ein2/sid2:avrRpt2	0.83387	3.2E-38
_Col:_pLAFR:dde2/pad4:avrRpt2	0.91781	4.5E-12
_Col:_pLAFR:dde2/pad4/sid2:avrRpt2	0.86012	1.9E-08
_Col:_pLAFR:dde2/sid2:avrRpt2	0.82853	1.4E-28
_Col:_pLAFR:ein2:avrRpt2	0.89407	4.5E-95
_Col:_pLAFR:ein2/pad4:avrRpt2	0.61631	2.2E-52
_Col:_pLAFR:ein2/pad4/sid2:avrRpt2	0.44352	3.6E-10
_Col:_pLAFR:ein2/sid2:avrRpt2	0.59013	2.8E-32
_Col:_pLAFR:npr1:avrRpt2	0.97556	5.5E-37
_Col:_pLAFR:pad4:avrRpt2	0.89949	1E-64
_Col:_pLAFR:pad4/sid2:avrRpt2	0.88958	7.8E-05
_Col:_pLAFR:rpm1/rps2:avrRpt2	0.73319	0.80021
_Col:_pLAFR:sid2:avrRpt2	0.95164	3.1E-49
dde2:_pLAFR:dde2/ein2:_pLAFR	0.89432	0.64551
dde2:_pLAFR:dde2/ein2/pad4:_pLAFR	0.88829	1.5E-12
dde2:_pLAFR:dde2/ein2/pad4/sid2:_pLAFR	0.5913	7.8E-17
dde2:_pLAFR:dde2/ein2/sid2:_pLAFR	0.52914	2E-09
dde2:_pLAFR:dde2/pad4:_pLAFR	0.48156	3.2E-06
dde2:_pLAFR:dde2/pad4/sid2:_pLAFR	0.75564	8.4E-07
dde2:_pLAFR:dde2/sid2:_pLAFR	0.63415	0.00298
dde2:_pLAFR:ein2:_pLAFR	0.42014	0.71816
dde2:_pLAFR:ein2/pad4:_pLAFR	0.99955	3.3E-06
dde2:_pLAFR:ein2/pad4/sid2:_pLAFR	0.87307	1.3E-06
dde2:_pLAFR:ein2/sid2:_pLAFR	0.83994	0.00021
dde2:_pLAFR:npr1:_pLAFR	0.4614	0.00019
dde2:_pLAFR:pad4:_pLAFR	0.65064	0.16136
dde2:_pLAFR:pad4/sid2:_pLAFR	0.8009	3.6E-06
dde2:_pLAFR:rpm1/rps2:_pLAFR	0.39043	0.85429
dde2:_pLAFR:sid2:_pLAFR	0.73533	0.00707
dde2:_pLAFR:_Col:avrRpt2	0.82879	1.8E-96
dde2:_pLAFR:dde2:avrRpt2	0.97885	2.7E-55
dde2:_pLAFR:dde2/ein2:avrRpt2	0.93474	3.1E-54
dde2:_pLAFR:dde2/ein2/pad4:avrRpt2	0.96862	3.7E-10
dde2:_pLAFR:dde2/ein2/pad4/sid2:avrRpt2	0.99095	0.04203
dde2:_pLAFR:dde2/ein2/sid2:avrRpt2	0.73072	1.1E-28
dde2:_pLAFR:dde2/pad4:avrRpt2	0.94091	2.2E-09
dde2:_pLAFR:dde2/pad4/sid2:avrRpt2	0.99105	1.2E-06
dde2:_pLAFR:dde2/sid2:avrRpt2	0.98115	1.4E-21
dde2:_pLAFR:ein2:avrRpt2	0.96192	2.9E-72

dde2:_pLAFR:ein2/pad4:avrRpt2	0.78743	3.6E-39
dde2:_pLAFR:ein2/pad4/sid2:avrRpt2	0.61792	6.2E-08
dde2:_pLAFR:ein2/sid2:avrRpt2	0.762	1.9E-24
dde2:_pLAFR:npr1:avrRpt2	0.89152	5.7E-28
dde2:_pLAFR:pad4:avrRpt2	0.9568	1E-48
dde2:_pLAFR:pad4/sid2:avrRpt2	0.96565	0.00056
dde2:_pLAFR:rpm1/rps2:avrRpt2	0.64205	0.94972
dde2:_pLAFR:sid2:avrRpt2	0.9116	5.4E-37
dde2/ein2:_pLAFR:dde2/ein2/pad4:_pLAFR	0.9947	1.4E-13
dde2/ein2:_pLAFR:dde2/ein2/pad4/sid2:_pLAI	0.69005	3E-19
dde2/ein2:_pLAFR:dde2/ein2/sid2:_pLAFR	0.6251	2.4E-10
dde2/ein2:_pLAFR:dde2/pad4:_pLAFR	0.56789	3E-07
dde2/ein2:_pLAFR:dde2/pad4/sid2:_pLAFR	0.85596	4.1E-08
dde2/ein2:_pLAFR:dde2/sid2:_pLAFR	0.72757	0.00047
dde2/ein2:_pLAFR:ein2:_pLAFR	0.50712	0.9151
dde2/ein2:_pLAFR:ein2/pad4:_pLAFR	0.89253	1.9E-07
dde2/ein2:_pLAFR:ein2/pad4/sid2:_pLAFR	0.97825	6.8E-08
dde2/ein2:_pLAFR:ein2/sid2:_pLAFR	0.9469	4.4E-05
dde2/ein2:_pLAFR:npr1:_pLAFR	0.55215	3.8E-05
dde2/ein2:_pLAFR:pad4:_pLAFR	0.7489	0.06251
dde2/ein2:_pLAFR:pad4/sid2:_pLAFR	0.90685	5.7E-07
dde2/ein2:_pLAFR:rpm1/rps2:_pLAFR	0.4775	0.50388
dde2/ein2:_pLAFR:sid2:_pLAFR	0.83468	0.0013
dde2/ein2:_pLAFR:_Col:avrRpt2	0.92045	1.5E-94
dde2/ein2:_pLAFR:dde2:avrRpt2	0.89839	1.2E-53
dde2/ein2:_pLAFR:dde2/ein2:avrRpt2	0.85446	1.4E-52
dde2/ein2:_pLAFR:dde2/ein2/pad4:avrRpt2	0.88814	2.7E-09
dde2/ein2:_pLAFR:dde2/ein2/pad4/sid2:avrRp	0.89775	0.01371
dde2/ein2:_pLAFR:dde2/ein2/sid2:avrRpt2	0.80735	2.5E-27
dde2/ein2:_pLAFR:dde2/pad4:avrRpt2	0.9784	1.5E-08
dde2/ein2:_pLAFR:dde2/pad4/sid2:avrRpt2	0.92812	5.8E-06
dde2/ein2:_pLAFR:dde2/sid2:avrRpt2	0.90041	2.3E-20
dde2/ein2:_pLAFR:ein2:avrRpt2	0.95752	1.6E-70
dde2/ein2:_pLAFR:ein2/pad4:avrRpt2	0.71018	1.2E-37
dde2/ein2:_pLAFR:ein2/pad4/sid2:avrRpt2	0.54767	3.7E-07
dde2/ein2:_pLAFR:ein2/sid2:avrRpt2	0.68573	3.6E-23
dde2/ein2:_pLAFR:npr1:avrRpt2	0.97163	1.3E-26
dde2/ein2:_pLAFR:pad4:avrRpt2	0.96245	4.2E-47
dde2/ein2:_pLAFR:pad4/sid2:avrRpt2	0.95369	0.00186
dde2/ein2:_pLAFR:rpm1/rps2:avrRpt2	0.72686	0.63111
dde2/ein2:_pLAFR:sid2:avrRpt2	0.99216	1.7E-35
dde2/ein2/pad4:_pLAFR:dde2/ein2/pad4/sid2:	0.69704	0.39843
dde2/ein2/pad4:_pLAFR:dde2/ein2/sid2:_pLAI	0.6275	0.23516
dde2/ein2/pad4:_pLAFR:dde2/pad4:_pLAFR	0.57026	0.0117
dde2/ein2/pad4:_pLAFR:dde2/pad4/sid2:_pLA	0.86216	0.03614
dde2/ein2/pad4:_pLAFR:dde2/sid2:_pLAFR	0.73436	3.8E-05
dde2/ein2/pad4:_pLAFR:ein2:_pLAFR	0.50853	1.2E-13
dde2/ein2/pad4:_pLAFR:ein2/pad4:_pLAFR	0.88919	0.01588
dde2/ein2/pad4:_pLAFR:ein2/pad4/sid2:_pLAI	0.98393	0.02624
dde2/ein2/pad4:_pLAFR:ein2/sid2:_pLAFR	0.9519	0.00041
dde2/ein2/pad4:_pLAFR:npr1:_pLAFR	0.55394	0.00046
dde2/ein2/pad4:_pLAFR:pad4:_pLAFR	0.75262	1E-08
dde2/ein2/pad4:_pLAFR:pad4/sid2:_pLAFR	0.91052	0.01039
dde2/ein2/pad4:_pLAFR:rpm1/rps2:_pLAFR	0.47686	4E-14
dde2/ein2/pad4:_pLAFR:sid2:_pLAFR	0.841	1.1E-05
dde2/ein2/pad4:_pLAFR:_Col:avrRpt2	0.92524	1E-150
dde2/ein2/pad4:_pLAFR:dde2:avrRpt2	0.89449	7.2E-94
dde2/ein2/pad4:_pLAFR:dde2/ein2:avrRpt2	0.85064	8.5E-93
dde2/ein2/pad4:_pLAFR:dde2/ein2/pad4:avrR	0.88425	6.6E-32
dde2/ein2/pad4:_pLAFR:dde2/ein2/pad4/sid2:	0.89327	5.5E-06
dde2/ein2/pad4:_pLAFR:dde2/ein2/sid2:avrRp	0.8115	4E-60
dde2/ein2/pad4:_pLAFR:dde2/pad4:avrRpt2	0.97437	2.4E-30
dde2/ein2/pad4:_pLAFR:dde2/pad4/sid2:avrR	0.92416	2.1E-25

dde2/ein2/pad4:_pLAFR:dde2/sid2:avrRpt2	0.8965	6E-50
dde2/ein2/pad4:_pLAFR:ein2:avrRpt2	0.95353	6E-114
dde2/ein2/pad4:_pLAFR:ein2/pad4:avrRpt2	0.70672	3.1E-74
dde2/ein2/pad4:_pLAFR:ein2/pad4/sid2:avrRp	0.54475	7.4E-28
dde2/ein2/pad4:_pLAFR:ein2/sid2:avrRpt2	0.68235	7.6E-54
dde2/ein2/pad4:_pLAFR:npr1:avrRpt2	0.97571	8.8E-59
dde2/ein2/pad4:_pLAFR:pad4:avrRpt2	0.95844	5E-86
dde2/ein2/pad4:_pLAFR:pad4/sid2:avrRpt2	0.9497	1.8E-19
dde2/ein2/pad4:_pLAFR:rpm1/rps2:avrRpt2	0.73168	8.9E-11
dde2/ein2/pad4:_pLAFR:sid2:avrRpt2	0.99623	3.7E-71
dde2/ein2/pad4/sid2:_pLAFR:dde2/ein2/sid2:_	0.89661	0.03533
dde2/ein2/pad4/sid2:_pLAFR:dde2/pad4:_pLA	0.82566	0.00036
dde2/ein2/pad4/sid2:_pLAFR:dde2/pad4/sid2:_	0.83819	0.00149
dde2/ein2/pad4/sid2:_pLAFR:dde2/sid2:_pLAF	0.98123	7.4E-08
dde2/ein2/pad4/sid2:_pLAFR:ein2:_pLAFR	0.75086	3.5E-18
dde2/ein2/pad4/sid2:_pLAFR:ein2/pad4:_pLAF	0.58681	0.00043
dde2/ein2/pad4/sid2:_pLAFR:ein2/pad4/sid2:_	0.71169	0.00092
dde2/ein2/pad4/sid2:_pLAFR:ein2/sid2:_pLAF	0.74669	4.7E-06
dde2/ein2/pad4/sid2:_pLAFR:npr1:_pLAFR	0.80751	5.5E-06
dde2/ein2/pad4/sid2:_pLAFR:pad4:_pLAFR	0.95859	2E-12
dde2/ein2/pad4/sid2:_pLAFR:pad4/sid2:_pLAF	0.78744	0.00035
dde2/ein2/pad4/sid2:_pLAFR:rpm1/rps2:_pLA	0.70458	2.6E-24
dde2/ein2/pad4/sid2:_pLAFR:sid2:_pLAFR	0.86126	1.3E-08
dde2/ein2/pad4/sid2:_pLAFR:_Col:avrRpt2	0.78284	4E-224
dde2/ein2/pad4/sid2:_pLAFR:dde2:avrRpt2	0.63801	5E-127
dde2/ein2/pad4/sid2:_pLAFR:dde2/ein2:avrRp	0.59196	4E-126
dde2/ein2/pad4/sid2:_pLAFR:dde2/ein2/pad4:	0.62696	1.9E-46
dde2/ein2/pad4/sid2:_pLAFR:dde2/ein2/pad4/	0.57774	2.9E-11
dde2/ein2/pad4/sid2:_pLAFR:dde2/ein2/sid2:a	0.96686	2.7E-84
dde2/ein2/pad4/sid2:_pLAFR:dde2/pad4:avrR	0.72226	4.3E-44
dde2/ein2/pad4/sid2:_pLAFR:dde2/pad4/sid2:	0.6681	1.6E-37
dde2/ein2/pad4/sid2:_pLAFR:dde2/sid2:avrRp	0.63899	1.1E-70
dde2/ein2/pad4/sid2:_pLAFR:ein2:avrRpt2	0.70056	4E-152
dde2/ein2/pad4/sid2:_pLAFR:ein2/pad4:avrRp	0.45127	5E-103
dde2/ein2/pad4/sid2:_pLAFR:ein2/pad4/sid2:a	0.30848	6.6E-41
dde2/ein2/pad4/sid2:_pLAFR:ein2/sid2:avrRpt	0.42949	1.8E-75
dde2/ein2/pad4/sid2:_pLAFR:npr1:avrRpt2	0.77792	5.9E-82
dde2/ein2/pad4/sid2:_pLAFR:pad4:avrRpt2	0.70502	2E-117
dde2/ein2/pad4/sid2:_pLAFR:pad4/sid2:avrRp	0.69606	4.2E-29
dde2/ein2/pad4/sid2:_pLAFR:rpm1/rps2:avrRp	0.97496	1.5E-19
dde2/ein2/pad4/sid2:_pLAFR:sid2:avrRpt2	0.75417	1.5E-98
dde2/ein2/sid2:_pLAFR:dde2/pad4:_pLAFR	0.93465	0.17715
dde2/ein2/sid2:_pLAFR:dde2/pad4/sid2:_pLAF	0.75921	0.36074
dde2/ein2/sid2:_pLAFR:dde2/sid2:_pLAFR	0.88723	0.0032
dde2/ein2/sid2:_pLAFR:ein2:_pLAFR	0.85949	2.3E-10
dde2/ein2/sid2:_pLAFR:ein2/pad4:_pLAFR	0.53396	0.20791
dde2/ein2/sid2:_pLAFR:ein2/pad4/sid2:_pLAF	0.64428	0.28535
dde2/ein2/sid2:_pLAFR:ein2/sid2:_pLAFR	0.66905	0.01646
dde2/ein2/sid2:_pLAFR:npr1:_pLAFR	0.91454	0.01789
dde2/ein2/sid2:_pLAFR:pad4:_pLAFR	0.86574	5.5E-06
dde2/ein2/sid2:_pLAFR:pad4/sid2:_pLAFR	0.70874	0.17586
dde2/ein2/sid2:_pLAFR:rpm1/rps2:_pLAFR	0.85284	3.6E-10
dde2/ein2/sid2:_pLAFR:sid2:_pLAFR	0.77972	0.00129
dde2/ein2/sid2:_pLAFR:_Col:avrRpt2	0.74157	3E-141
dde2/ein2/sid2:_pLAFR:dde2:avrRpt2	0.61813	4.7E-87
dde2/ein2/sid2:_pLAFR:dde2/ein2:avrRpt2	0.57894	5.7E-86
dde2/ein2/sid2:_pLAFR:dde2/ein2/pad4:avrRp	0.60875	1.6E-27
dde2/ein2/sid2:_pLAFR:dde2/ein2/pad4/sid2:a	0.57716	0.00053
dde2/ein2/sid2:_pLAFR:dde2/ein2/sid2:avrRpt	0.89825	2.6E-54
dde2/ein2/sid2:_pLAFR:dde2/pad4:avrRpt2	0.68986	4.2E-26
dde2/ein2/sid2:_pLAFR:dde2/pad4/sid2:avrRp	0.64386	1.7E-21
dde2/ein2/sid2:_pLAFR:dde2/sid2:avrRpt2	0.61909	1.4E-44
dde2/ein2/sid2:_pLAFR:ein2:avrRpt2	0.67137	1E-106

dde2/ein2/sid2:_pLAFR:ein2/pad4:avrRpt2	0.45774	7E-68
dde2/ein2/sid2:_pLAFR:ein2/pad4/sid2:avrRpt	0.33131	9.3E-24
dde2/ein2/sid2:_pLAFR:ein2/sid2:avrRpt2	0.43854	2.4E-48
dde2/ein2/sid2:_pLAFR:npr1:avrRpt2	0.73712	4.5E-53
dde2/ein2/sid2:_pLAFR:pad4:avrRpt2	0.67521	2.3E-79
dde2/ein2/sid2:_pLAFR:pad4/sid2:avrRpt2	0.66757	4.6E-16
dde2/ein2/sid2:_pLAFR:rpm1/rps2:avrRpt2	0.93554	6.1E-08
dde2/ein2/sid2:_pLAFR:sid2:avrRpt2	0.71695	6E-65
dde2/pad4:_pLAFR:dde2/pad4/sid2:_pLAFR	0.69436	0.65507
dde2/pad4:_pLAFR:dde2/sid2:_pLAFR	0.82111	0.09253
dde2/pad4:_pLAFR:ein2:_pLAFR	0.92596	5.5E-07
dde2/pad4:_pLAFR:ein2/pad4:_pLAFR	0.48105	0.9096
dde2/pad4:_pLAFR:ein2/pad4/sid2:_pLAFR	0.58629	0.76352
dde2/pad4:_pLAFR:ein2/sid2:_pLAFR	0.61402	0.30202
dde2/pad4:_pLAFR:npr1:_pLAFR	0.98082	0.3167
dde2/pad4:_pLAFR:pad4:_pLAFR	0.80157	0.00104
dde2/pad4:_pLAFR:pad4/sid2:_pLAFR	0.64839	0.97061
dde2/pad4:_pLAFR:rpm1/rps2:_pLAFR	0.92281	1.6E-06
dde2/pad4:_pLAFR:sid2:_pLAFR	0.71454	0.04937
dde2/pad4:_pLAFR:_Col:avrRpt2	0.68689	5E-130
dde2/pad4:_pLAFR:dde2:avrRpt2	0.57407	4.2E-79
dde2/pad4:_pLAFR:dde2/ein2:avrRpt2	0.53606	5.1E-78
dde2/pad4:_pLAFR:dde2/ein2/pad4:avrRpt2	0.56494	9.6E-23
dde2/pad4:_pLAFR:dde2/ein2/pad4/sid2:avrRp	0.52766	0.02786
dde2/pad4:_pLAFR:dde2/ein2/sid2:avrRpt2	0.84887	1.1E-47
dde2/pad4:_pLAFR:dde2/pad4:avrRpt2	0.6437	1.8E-21
dde2/pad4:_pLAFR:dde2/pad4/sid2:avrRpt2	0.59889	2.9E-17
dde2/pad4:_pLAFR:dde2/sid2:avrRpt2	0.57486	1.5E-38
dde2/pad4:_pLAFR:ein2:avrRpt2	0.62579	3.6E-98
dde2/pad4:_pLAFR:ein2/pad4:avrRpt2	0.41976	1.4E-60
dde2/pad4:_pLAFR:ein2/pad4/sid2:avrRpt2	0.30008	2.6E-19
dde2/pad4:_pLAFR:ein2/sid2:avrRpt2	0.40155	4E-42
dde2/pad4:_pLAFR:npr1:avrRpt2	0.69001	1.4E-46
dde2/pad4:_pLAFR:pad4:avrRpt2	0.62942	1.2E-71
dde2/pad4:_pLAFR:pad4/sid2:avrRpt2	0.62204	1.8E-12
dde2/pad4:_pLAFR:rpm1/rps2:avrRpt2	0.87815	3.3E-05
dde2/pad4:_pLAFR:sid2:avrRpt2	0.67012	8.1E-58
dde2/pad4/sid2:_pLAFR:dde2/sid2:_pLAFR	0.86671	0.03487
dde2/pad4/sid2:_pLAFR:ein2:_pLAFR	0.63109	1.4E-07
dde2/pad4/sid2:_pLAFR:ein2/pad4:_pLAFR	0.75214	0.73357
dde2/pad4/sid2:_pLAFR:ein2/pad4/sid2:_pLAF	0.87728	0.8831
dde2/pad4/sid2:_pLAFR:ein2/sid2:_pLAFR	0.91002	0.15219
dde2/pad4/sid2:_pLAFR:npr1:_pLAFR	0.68048	0.16088
dde2/pad4/sid2:_pLAFR:pad4:_pLAFR	0.88834	0.00024
dde2/pad4/sid2:_pLAFR:pad4/sid2:_pLAFR	0.95004	0.67811
dde2/pad4/sid2:_pLAFR:rpm1/rps2:_pLAFR	0.60399	1.3E-07
dde2/pad4/sid2:_pLAFR:sid2:_pLAFR	0.97818	0.0169
dde2/pad4/sid2:_pLAFR:_Col:avrRpt2	0.95287	5E-136
dde2/pad4/sid2:_pLAFR:dde2:avrRpt2	0.79087	9.9E-83
dde2/pad4/sid2:_pLAFR:dde2/ein2:avrRpt2	0.748	1.2E-81
dde2/pad4/sid2:_pLAFR:dde2/ein2/pad4:avrRp	0.78077	1.3E-24
dde2/pad4/sid2:_pLAFR:dde2/ein2/pad4/sid2:a	0.77349	0.00822
dde2/pad4/sid2:_pLAFR:dde2/ein2/sid2:avrRp	0.91502	1.7E-50
dde2/pad4/sid2:_pLAFR:dde2/pad4:avrRpt2	0.86909	2.9E-23
dde2/pad4/sid2:_pLAFR:dde2/pad4/sid2:avrRp	0.81956	7.4E-19
dde2/pad4/sid2:_pLAFR:dde2/sid2:avrRpt2	0.7925	4.8E-41
dde2/pad4/sid2:_pLAFR:ein2:avrRpt2	0.84877	3E-102
dde2/pad4/sid2:_pLAFR:ein2/pad4:avrRpt2	0.61033	8.6E-64
dde2/pad4/sid2:_pLAFR:ein2/pad4/sid2:avrRp	0.45967	5.2E-21
dde2/pad4/sid2:_pLAFR:ein2/sid2:avrRpt2	0.58758	1E-44
dde2/pad4/sid2:_pLAFR:npr1:avrRpt2	0.91893	2.5E-49
dde2/pad4/sid2:_pLAFR:pad4:avrRpt2	0.85336	3.9E-75
dde2/pad4/sid2:_pLAFR:pad4/sid2:avrRpt2	0.84488	8.7E-14

dde2/pad4/sid2:_pLAFR:rpm1/rps2:avrRpt2	0.84871	3.9E-06
dde2/pad4/sid2:_pLAFR:sid2:avrRpt2	0.89816	6.3E-61
dde2/sid2:_pLAFR:ein2:_pLAFR	0.75236	0.00091
dde2/sid2:_pLAFR:ein2/pad4:_pLAFR	0.62936	0.07839
dde2/sid2:_pLAFR:ein2/pad4/sid2:_pLAFR	0.74802	0.05131
dde2/sid2:_pLAFR:ein2/sid2:_pLAFR	0.78123	0.53065
dde2/sid2:_pLAFR:npr1:_pLAFR	0.80482	0.51086
dde2/sid2:_pLAFR:pad4:_pLAFR	0.9789	0.10439
dde2/sid2:_pLAFR:pad4/sid2:_pLAFR	0.81936	0.09562
dde2/sid2:_pLAFR:rpm1/rps2:_pLAFR	0.73427	0.00261
dde2/sid2:_pLAFR:sid2:_pLAFR	0.88827	0.7746
dde2/sid2:_pLAFR:_Col:avrRpt2	0.8373	1E-118
dde2/sid2:_pLAFR:dde2:avrRpt2	0.69548	1.2E-70
dde2/sid2:_pLAFR:dde2/ein2:avrRpt2	0.65428	1.5E-69
dde2/sid2:_pLAFR:dde2/ein2/pad4:avrRpt2	0.68569	8.9E-18
dde2/sid2:_pLAFR:dde2/ein2/pad4/sid2:avrRp	0.66423	0.49177
dde2/sid2:_pLAFR:dde2/ein2/sid2:avrRpt2	0.98438	1.1E-40
dde2/sid2:_pLAFR:dde2/pad4:avrRpt2	0.77076	1.1E-16
dde2/sid2:_pLAFR:dde2/pad4/sid2:avrRpt2	0.72276	5.9E-13
dde2/sid2:_pLAFR:dde2/sid2:avrRpt2	0.69673	3.6E-32
dde2/sid2:_pLAFR:ein2:avrRpt2	0.75128	4.1E-89
dde2/sid2:_pLAFR:ein2/pad4:avrRpt2	0.52471	8.2E-53
dde2/sid2:_pLAFR:ein2/pad4/sid2:avrRpt2	0.38659	9.6E-15
dde2/sid2:_pLAFR:ein2/sid2:avrRpt2	0.50379	1.6E-35
dde2/sid2:_pLAFR:npr1:avrRpt2	0.81961	1.1E-39
dde2/sid2:_pLAFR:pad4:avrRpt2	0.75549	1.9E-63
dde2/sid2:_pLAFR:pad4/sid2:avrRpt2	0.7474	7E-09
dde2/sid2:_pLAFR:rpm1/rps2:avrRpt2	0.96443	0.00778
dde2/sid2:_pLAFR:sid2:avrRpt2	0.79898	3.1E-50
ein2:_pLAFR:ein2/pad4:_pLAFR	0.42568	6E-07
ein2:_pLAFR:ein2/pad4/sid2:_pLAFR	0.5245	2.3E-07
ein2:_pLAFR:ein2/sid2:_pLAFR	0.54559	4.8E-05
ein2:_pLAFR:npr1:_pLAFR	0.94442	4.2E-05
ein2:_pLAFR:pad4:_pLAFR	0.73086	0.0794
ein2:_pLAFR:pad4/sid2:_pLAFR	0.58268	6.4E-07
ein2:_pLAFR:rpm1/rps2:_pLAFR	0.99684	0.57536
ein2:_pLAFR:sid2:_pLAFR	0.65028	0.00235
ein2:_pLAFR:_Col:avrRpt2	0.62888	3.7E-94
ein2:_pLAFR:dde2:avrRpt2	0.52792	1.1E-53
ein2:_pLAFR:dde2/ein2:avrRpt2	0.49151	1.3E-52
ein2:_pLAFR:dde2/ein2/pad4:avrRpt2	0.51913	2E-09
ein2:_pLAFR:dde2/ein2/pad4/sid2:avrRpt2	0.47705	0.01867
ein2:_pLAFR:dde2/ein2/sid2:avrRpt2	0.79438	1.8E-27
ein2:_pLAFR:dde2/pad4:avrRpt2	0.59467	1.1E-08
ein2:_pLAFR:dde2/pad4/sid2:avrRpt2	0.55156	4.4E-06
ein2:_pLAFR:dde2/sid2:avrRpt2	0.52853	1.6E-20
ein2:_pLAFR:ein2:avrRpt2	0.57755	1.8E-70
ein2:_pLAFR:ein2/pad4:avrRpt2	0.38132	9.6E-38
ein2:_pLAFR:ein2/pad4/sid2:avrRpt2	0.26947	2.7E-07
ein2:_pLAFR:ein2/sid2:avrRpt2	0.36427	2.6E-23
ein2:_pLAFR:npr1:avrRpt2	0.63951	9.3E-27
ein2:_pLAFR:pad4:avrRpt2	0.58093	3.6E-47
ein2:_pLAFR:pad4/sid2:avrRpt2	0.57389	0.00148
ein2:_pLAFR:rpm1/rps2:avrRpt2	0.81471	0.70317
ein2:_pLAFR:sid2:avrRpt2	0.62012	1.3E-35
ein2/pad4:_pLAFR:ein2/pad4/sid2:_pLAFR	0.87101	0.84527
ein2/pad4:_pLAFR:ein2/sid2:_pLAFR	0.84148	0.26345
ein2/pad4:_pLAFR:npr1:_pLAFR	0.46671	0.27649
ein2/pad4:_pLAFR:pad4:_pLAFR	0.65011	0.00092
ein2/pad4:_pLAFR:pad4/sid2:_pLAFR	0.80282	0.93783
ein2/pad4:_pLAFR:rpm1/rps2:_pLAFR	0.3932	1E-06
ein2/pad4:_pLAFR:sid2:_pLAFR	0.7316	0.04164
ein2/pad4:_pLAFR:_Col:avrRpt2	0.82787	4E-133

ein2/pad4:_pLAFR:dde2:avrRpt2	0.97915	1E-80
ein2/pad4:_pLAFR:dde2/ein2:avrRpt2	0.93493	1.2E-79
ein2/pad4:_pLAFR:dde2/ein2/pad4:avrRpt2	0.9689	2E-23
ein2/pad4:_pLAFR:dde2/ein2/pad4/sid2:avrRp	0.99132	0.01988
ein2/pad4:_pLAFR:dde2/ein2/sid2:avrRpt2	0.7298	7.6E-49
ein2/pad4:_pLAFR:dde2/pad4:avrRpt2	0.94043	4E-22
ein2/pad4:_pLAFR:dde2/pad4/sid2:avrRpt2	0.99068	7.9E-18
ein2/pad4:_pLAFR:dde2/sid2:avrRpt2	0.98145	1.5E-39
ein2/pad4:_pLAFR:ein2:avrRpt2	0.96148	5E-100
ein2/pad4:_pLAFR:ein2/pad4:avrRpt2	0.78728	6E-62
ein2/pad4:_pLAFR:ein2/pad4/sid2:avrRpt2	0.61741	6.4E-20
ein2/pad4:_pLAFR:ein2/sid2:avrRpt2	0.7618	3.6E-43
ein2/pad4:_pLAFR:npr1:avrRpt2	0.89094	1.1E-47
ein2/pad4:_pLAFR:pad4:avrRpt2	0.95636	3.5E-73
ein2/pad4:_pLAFR:pad4/sid2:avrRpt2	0.96523	6.4E-13
ein2/pad4:_pLAFR:rpm1/rps2:avrRpt2	0.64066	1.7E-05
ein2/pad4:_pLAFR:sid2:avrRpt2	0.91105	3.9E-59
ein2/pad4/sid2:_pLAFR:ein2/sid2:_pLAFR	0.96836	0.19203
ein2/pad4/sid2:_pLAFR:npr1:_pLAFR	0.57028	0.20246
ein2/pad4/sid2:_pLAFR:pad4:_pLAFR	0.76941	0.00047
ein2/pad4/sid2:_pLAFR:pad4/sid2:_pLAFR	0.9281	0.78902
ein2/pad4/sid2:_pLAFR:rpm1/rps2:_pLAFR	0.49564	3.7E-07
ein2/pad4/sid2:_pLAFR:sid2:_pLAFR	0.8559	0.02603
ein2/pad4/sid2:_pLAFR:_Col:avrRpt2	0.93931	2E-134
ein2/pad4/sid2:_pLAFR:dde2:avrRpt2	0.88219	9.6E-82
ein2/pad4/sid2:_pLAFR:dde2/ein2:avrRpt2	0.83837	1.2E-80
ein2/pad4/sid2:_pLAFR:dde2/ein2/pad4:avrRp	0.87195	4.7E-24
ein2/pad4/sid2:_pLAFR:dde2/ein2/pad4/sid2:a	0.879	0.01228
ein2/pad4/sid2:_pLAFR:dde2/ein2/sid2:avrRpt	0.82326	1E-49
ein2/pad4/sid2:_pLAFR:dde2/pad4:avrRpt2	0.96202	9.9E-23
ein2/pad4/sid2:_pLAFR:dde2/pad4/sid2:avrRp	0.9118	2.2E-18
ein2/pad4/sid2:_pLAFR:dde2/sid2:avrRpt2	0.88416	2.5E-40
ein2/pad4/sid2:_pLAFR:ein2:avrRpt2	0.9412	4E-101
ein2/pad4/sid2:_pLAFR:ein2/pad4:avrRpt2	0.69495	6.7E-63
ein2/pad4/sid2:_pLAFR:ein2/pad4/sid2:avrRpt	0.53408	1.7E-20
ein2/pad4/sid2:_pLAFR:ein2/sid2:avrRpt2	0.67073	5.6E-44
ein2/pad4/sid2:_pLAFR:npr1:avrRpt2	0.98798	1.5E-48
ein2/pad4/sid2:_pLAFR:pad4:avrRpt2	0.94608	3.5E-74
ein2/pad4/sid2:_pLAFR:pad4/sid2:avrRpt2	0.93736	2.2E-13
ein2/pad4/sid2:_pLAFR:rpm1/rps2:avrRpt2	0.74473	7.6E-06
ein2/pad4/sid2:_pLAFR:sid2:avrRpt2	0.99144	4.6E-60
ein2/sid2:_pLAFR:npr1:_pLAFR	0.59285	0.97491
ein2/sid2:_pLAFR:pad4:_pLAFR	0.80001	0.02498
ein2/sid2:_pLAFR:pad4/sid2:_pLAFR	0.95901	0.29142
ein2/sid2:_pLAFR:rpm1/rps2:_pLAFR	0.5203	0.00023
ein2/sid2:_pLAFR:sid2:_pLAFR	0.88878	0.3657
ein2/sid2:_pLAFR:_Col:avrRpt2	0.96726	3E-121
ein2/sid2:_pLAFR:dde2:avrRpt2	0.85874	4.6E-73
ein2/sid2:_pLAFR:dde2/ein2:avrRpt2	0.81522	5.6E-72
ein2/sid2:_pLAFR:dde2/ein2/pad4:avrRpt2	0.84855	2.2E-19
ein2/sid2:_pLAFR:dde2/ein2/pad4/sid2:avrRpt	0.85195	0.21008
ein2/sid2:_pLAFR:dde2/ein2/sid2:avrRpt2	0.84718	8.1E-43
ein2/sid2:_pLAFR:dde2/pad4:avrRpt2	0.93806	3.1E-18
ein2/sid2:_pLAFR:dde2/pad4/sid2:avrRpt2	0.88807	2.3E-14
ein2/sid2:_pLAFR:dde2/sid2:avrRpt2	0.86062	3.6E-34
ein2/sid2:_pLAFR:ein2:avrRpt2	0.91739	1.3E-91
ein2/sid2:_pLAFR:ein2/pad4:avrRpt2	0.67344	4.1E-55
ein2/sid2:_pLAFR:ein2/pad4/sid2:avrRpt2	0.51535	3.1E-16
ein2/sid2:_pLAFR:ein2/sid2:avrRpt2	0.64962	1.4E-37
ein2/sid2:_pLAFR:npr1:avrRpt2	0.98796	8.2E-42
ein2/sid2:_pLAFR:pad4:avrRpt2	0.92219	7.9E-66
ein2/sid2:_pLAFR:pad4/sid2:avrRpt2	0.91354	4.5E-10
ein2/sid2:_pLAFR:rpm1/rps2:avrRpt2	0.772	0.00135

ein2/sid2:_pLAFR:sid2:avrRpt2	0.96735	1.7E-52
npr1:_pLAFR:pad4:_pLAFR	0.78324	0.02306
npr1:_pLAFR:pad4/sid2:_pLAFR	0.63109	0.30556
npr1:_pLAFR:rpm1/rps2:_pLAFR	0.94368	0.0002
npr1:_pLAFR:sid2:_pLAFR	0.70026	0.34977
npr1:_pLAFR:_Col:avrRpt2	0.67241	2E-121
npr1:_pLAFR:dde2:avrRpt2	0.56263	3.2E-73
npr1:_pLAFR:dde2/ein2:avrRpt2	0.52507	3.9E-72
npr1:_pLAFR:dde2/ein2/pad4:avrRpt2	0.5536	1.8E-19
npr1:_pLAFR:dde2/ein2/pad4/sid2:avrRpt2	0.5153	0.20013
npr1:_pLAFR:dde2/ein2/sid2:avrRpt2	0.83496	6E-43
npr1:_pLAFR:dde2/pad4:avrRpt2	0.63144	2.5E-18
npr1:_pLAFR:dde2/pad4/sid2:avrRpt2	0.58712	1.9E-14
npr1:_pLAFR:dde2/sid2:avrRpt2	0.56337	2.7E-34
npr1:_pLAFR:ein2:avrRpt2	0.61376	9.1E-92
npr1:_pLAFR:ein2/pad4:avrRpt2	0.41043	2.9E-55
npr1:_pLAFR:ein2/pad4/sid2:avrRpt2	0.2928	2.6E-16
npr1:_pLAFR:ein2/sid2:avrRpt2	0.39253	1.1E-37
npr1:_pLAFR:npr1:avrRpt2	0.67732	6.1E-42
npr1:_pLAFR:pad4:avrRpt2	0.61732	5.6E-66
npr1:_pLAFR:pad4/sid2:avrRpt2	0.61004	3.9E-10
npr1:_pLAFR:rpm1/rps2:avrRpt2	0.86193	0.00123
npr1:_pLAFR:sid2:avrRpt2	0.65759	1.2E-52
pad4:_pLAFR:pad4/sid2:_pLAFR	0.83877	0.00108
pad4:_pLAFR:rpm1/rps2:_pLAFR	0.71186	0.18927
pad4:_pLAFR:sid2:_pLAFR	0.90986	0.18005
pad4:_pLAFR:_Col:avrRpt2	0.85561	4E-107
pad4:_pLAFR:dde2:avrRpt2	0.71054	1.8E-62
pad4:_pLAFR:dde2/ein2:avrRpt2	0.66907	2.1E-61
pad4:_pLAFR:dde2/ein2/pad4:avrRpt2	0.7007	1.8E-13
pad4:_pLAFR:dde2/ein2/pad4/sid2:avrRpt2	0.68147	0.43767
pad4:_pLAFR:dde2/ein2/sid2:avrRpt2	0.99965	3.8E-34
pad4:_pLAFR:dde2/pad4:avrRpt2	0.78631	1.5E-12
pad4:_pLAFR:dde2/pad4/sid2:avrRpt2	0.73805	2.5E-09
pad4:_pLAFR:dde2/sid2:avrRpt2	0.71186	2.3E-26
pad4:_pLAFR:ein2:avrRpt2	0.76669	3.2E-80
pad4:_pLAFR:ein2/pad4:avrRpt2	0.53818	1.8E-45
pad4:_pLAFR:ein2/pad4/sid2:avrRpt2	0.39805	7.4E-11
pad4:_pLAFR:ein2/sid2:avrRpt2	0.51696	1.8E-29
pad4:_pLAFR:npr1:avrRpt2	0.83533	2.7E-33
pad4:_pLAFR:pad4:avrRpt2	0.77096	1.4E-55
pad4:_pLAFR:pad4/sid2:avrRpt2	0.76281	5.4E-06
pad4:_pLAFR:rpm1/rps2:avrRpt2	0.94603	0.2288
pad4:_pLAFR:sid2:avrRpt2	0.81468	4.3E-43
pad4/sid2:_pLAFR:rpm1/rps2:_pLAFR	0.55481	1E-06
pad4/sid2:_pLAFR:sid2:_pLAFR	0.92856	0.05228
pad4/sid2:_pLAFR:_Col:avrRpt2	0.99696	4E-132
pad4/sid2:_pLAFR:dde2:avrRpt2	0.82821	4.2E-80
pad4/sid2:_pLAFR:dde2/ein2:avrRpt2	0.78495	5.1E-79
pad4/sid2:_pLAFR:dde2/ein2/pad4:avrRpt2	0.81805	4.1E-23
pad4/sid2:_pLAFR:dde2/ein2/pad4/sid2:avrRp	0.81665	0.02433
pad4/sid2:_pLAFR:dde2/ein2/sid2:avrRpt2	0.87752	2.3E-48
pad4/sid2:_pLAFR:dde2/pad4:avrRpt2	0.90709	8E-22
pad4/sid2:_pLAFR:dde2/pad4/sid2:avrRpt2	0.85728	1.5E-17
pad4/sid2:_pLAFR:dde2/sid2:avrRpt2	0.82998	4E-39
pad4/sid2:_pLAFR:ein2:avrRpt2	0.88656	2E-99
pad4/sid2:_pLAFR:ein2/pad4:avrRpt2	0.64493	2.1E-61
pad4/sid2:_pLAFR:ein2/pad4/sid2:avrRpt2	0.4901	1.2E-19
pad4/sid2:_pLAFR:ein2/sid2:avrRpt2	0.62158	1E-42
pad4/sid2:_pLAFR:npr1:avrRpt2	0.95699	3.1E-47
pad4/sid2:_pLAFR:pad4:avrRpt2	0.89127	1.4E-72
pad4/sid2:_pLAFR:pad4/sid2:avrRpt2	0.88269	1.1E-12
pad4/sid2:_pLAFR:rpm1/rps2:avrRpt2	0.80622	2.4E-05

pad4/sid2:_pLAFR:sid2:avrRpt2	0.9363	1.3E-58
rpm1/rps2:_pLAFR:sid2:_pLAFR	0.62451	0.00666
rpm1/rps2:_pLAFR:_Col:avrRpt2	0.55429	1E-146
rpm1/rps2:_pLAFR:dde2:avrRpt2	0.46756	1.7E-74
rpm1/rps2:_pLAFR:dde2/ein2:avrRpt2	0.42797	2.3E-73
rpm1/rps2:_pLAFR:dde2/ein2/pad4:avrRpt2	0.45787	5.1E-14
rpm1/rps2:_pLAFR:dde2/ein2/pad4/sid2:avrRp	0.3832	0.01619
rpm1/rps2:_pLAFR:dde2/ein2/sid2:avrRpt2	0.76563	1.9E-39
rpm1/rps2:_pLAFR:dde2/pad4:avrRpt2	0.54025	7.1E-13
rpm1/rps2:_pLAFR:dde2/pad4/sid2:avrRpt2	0.49277	3.8E-09
rpm1/rps2:_pLAFR:dde2/sid2:avrRpt2	0.4677	1E-29
rpm1/rps2:_pLAFR:ein2:avrRpt2	0.52173	1.6E-96
rpm1/rps2:_pLAFR:ein2/pad4:avrRpt2	0.31268	1E-53
rpm1/rps2:_pLAFR:ein2/pad4/sid2:avrRpt2	0.20259	6.5E-11
rpm1/rps2:_pLAFR:ein2/sid2:avrRpt2	0.29579	1.9E-33
rpm1/rps2:_pLAFR:npr1:avrRpt2	0.59039	3.4E-38
rpm1/rps2:_pLAFR:pad4:avrRpt2	0.5251	4.2E-66
rpm1/rps2:_pLAFR:pad4/sid2:avrRpt2	0.51754	2.4E-05
rpm1/rps2:_pLAFR:rpm1/rps2:avrRpt2	0.77592	0.90643
rpm1/rps2:_pLAFR:sid2:avrRpt2	0.56836	1.5E-50
sid2:_pLAFR:_Col:avrRpt2	0.9339	1E-116
sid2:_pLAFR:dde2:avrRpt2	0.77507	3.2E-69
sid2:_pLAFR:dde2/ein2:avrRpt2	0.73243	3.9E-68
sid2:_pLAFR:dde2/ein2/pad4:avrRpt2	0.76501	5.5E-17
sid2:_pLAFR:dde2/ein2/pad4/sid2:avrRpt2	0.75531	0.66597
sid2:_pLAFR:dde2/ein2/sid2:avrRpt2	0.93136	1.6E-39
sid2:_pLAFR:dde2/pad4:avrRpt2	0.85289	6.4E-16
sid2:_pLAFR:dde2/pad4/sid2:avrRpt2	0.80356	2.8E-12
sid2:_pLAFR:dde2/sid2:avrRpt2	0.77663	3.9E-31
sid2:_pLAFR:ein2:avrRpt2	0.83268	1.4E-87
sid2:_pLAFR:ein2/pad4:avrRpt2	0.59596	1.6E-51
sid2:_pLAFR:ein2/pad4/sid2:avrRpt2	0.44726	5E-14
sid2:_pLAFR:ein2/sid2:avrRpt2	0.57349	1.9E-34
sid2:_pLAFR:npr1:avrRpt2	0.90262	1.5E-38
sid2:_pLAFR:pad4:avrRpt2	0.83722	4.6E-62
sid2:_pLAFR:pad4/sid2:avrRpt2	0.82879	2.5E-08
sid2:_pLAFR:rpm1/rps2:avrRpt2	0.86741	0.01606
sid2:_pLAFR:sid2:avrRpt2	0.88185	5.6E-49
_Col:avrRpt2:dde2:avrRpt2	0.76187	0.00017
_Col:avrRpt2:dde2/ein2:avrRpt2	0.69988	2.8E-05
_Col:avrRpt2:dde2/ein2/pad4:avrRpt2	0.74877	1.3E-66
_Col:avrRpt2:dde2/ein2/pad4/sid2:avrRpt2	0.73575	1E-212
_Col:avrRpt2:dde2/ein2/sid2:avrRpt2	0.82337	1.7E-27
_Col:avrRpt2:dde2/pad4:avrRpt2	0.87219	2.4E-67
_Col:avrRpt2:dde2/pad4/sid2:avrRpt2	0.80152	2.4E-82
_Col:avrRpt2:dde2/sid2:avrRpt2	0.76337	4.8E-38
_Col:avrRpt2:ein2:avrRpt2	0.84335	0.94431
_Col:avrRpt2:ein2/pad4:avrRpt2	0.513	2.5E-16
_Col:avrRpt2:ein2/pad4/sid2:avrRpt2	0.32613	1.8E-77
_Col:avrRpt2:ein2/sid2:avrRpt2	0.48695	8E-32
_Col:avrRpt2:npr1:avrRpt2	0.94276	1.1E-26
_Col:avrRpt2:pad4:avrRpt2	0.84992	5.4E-08
_Col:avrRpt2:pad4/sid2:avrRpt2	0.83854	4E-94
_Col:avrRpt2:rpm1/rps2:avrRpt2	0.71411	2E-178
_Col:avrRpt2:sid2:avrRpt2	0.91312	2.9E-17
dde2:avrRpt2:dde2/ein2:avrRpt2	0.94207	0.74239
dde2:avrRpt2:dde2/ein2/pad4:avrRpt2	0.98648	9E-37
dde2:avrRpt2:dde2/ein2/pad4/sid2:avrRpt2	0.98117	2E-114
dde2:avrRpt2:dde2/ein2/sid2:avrRpt2	0.62114	5E-11
dde2:avrRpt2:dde2/pad4:avrRpt2	0.8947	2.1E-39
dde2:avrRpt2:dde2/pad4/sid2:avrRpt2	0.96066	8.8E-45
dde2:avrRpt2:dde2/sid2:avrRpt2	0.99692	1.2E-17
dde2:avrRpt2:ein2:avrRpt2	0.92112	0.00039

dde2:avrRpt2:ein2/pad4:avrRpt2	0.74949	0.00013
dde2:avrRpt2:ein2/pad4/sid2:avrRpt2	0.53506	1.1E-41
dde2:avrRpt2:ein2/sid2:avrRpt2	0.71251	4.4E-15
dde2:avrRpt2:npr1:avrRpt2	0.82806	1E-11
dde2:avrRpt2:pad4:avrRpt2	0.91548	0.11896
dde2:avrRpt2:pad4/sid2:avrRpt2	0.92657	2.9E-57
dde2:avrRpt2:rpm1/rps2:avrRpt2	0.54082	8E-99
dde2:avrRpt2:sid2:avrRpt2	0.85754	1.5E-05
dde2/ein2:avrRpt2:dde2/ein2/pad4:avrRpt2	0.95571	4.3E-34
dde2/ein2:avrRpt2:dde2/ein2/pad4/sid2:avrRp	0.9182	2E-115
dde2/ein2:avrRpt2:dde2/ein2/sid2:avrRpt2	0.57504	8.2E-10
dde2/ein2:avrRpt2:dde2/pad4:avrRpt2	0.83745	9.4E-38
dde2/ein2:avrRpt2:dde2/pad4/sid2:avrRpt2	0.90201	7.7E-45
dde2/ein2:avrRpt2:dde2/sid2:avrRpt2	0.93856	3.9E-17
dde2/ein2:avrRpt2:ein2:avrRpt2	0.86466	0.00014
dde2/ein2:avrRpt2:ein2/pad4:avrRpt2	0.80274	0.00033
dde2/ein2:avrRpt2:ein2/pad4/sid2:avrRpt2	0.57913	1.2E-41
dde2/ein2:avrRpt2:ein2/sid2:avrRpt2	0.7709	1.8E-13
dde2/ein2:avrRpt2:npr1:avrRpt2	0.77402	2.3E-10
dde2/ein2:avrRpt2:pad4:avrRpt2	0.858	0.21818
dde2/ein2:avrRpt2:pad4/sid2:avrRpt2	0.87026	1.1E-53
dde2/ein2:avrRpt2:rpm1/rps2:avrRpt2	0.49286	5.5E-95
dde2/ein2:avrRpt2:sid2:avrRpt2	0.79873	4.3E-05
dde2/ein2/pad4:avrRpt2:dde2/ein2/pad4/sid2:a	0.96659	1.3E-28
dde2/ein2/pad4:avrRpt2:dde2/ein2/sid2:avrRp	0.61127	5.2E-11
dde2/ein2/pad4:avrRpt2:dde2/pad4:avrRpt2	0.88068	0.74477
dde2/ein2/pad4:avrRpt2:dde2/pad4/sid2:avrRp	0.94702	0.06313
dde2/ein2/pad4:avrRpt2:dde2/sid2:avrRpt2	0.98347	1.5E-05
dde2/ein2/pad4:avrRpt2:ein2:avrRpt2	0.90814	4.7E-56
dde2/ein2/pad4:avrRpt2:ein2/pad4:avrRpt2	0.76351	1.7E-19
dde2/ein2/pad4:avrRpt2:ein2/pad4/sid2:avrRp	0.54834	0.26165
dde2/ein2/pad4:avrRpt2:ein2/sid2:avrRpt2	0.7268	1E-07
dde2/ein2/pad4:avrRpt2:npr1:avrRpt2	0.81587	2.3E-10
dde2/ein2/pad4:avrRpt2:pad4:avrRpt2	0.90155	2.3E-29
dde2/ein2/pad4:avrRpt2:pad4/sid2:avrRpt2	0.91261	0.00018
dde2/ein2/pad4:avrRpt2:rpm1/rps2:avrRpt2	0.52674	1.5E-18
dde2/ein2/pad4:avrRpt2:sid2:avrRpt2	0.84348	1.7E-17
dde2/ein2/pad4/sid2:avrRpt2:dde2/ein2/sid2:a	0.61701	1.1E-67
dde2/ein2/pad4/sid2:avrRpt2:dde2/pad4:avrRp	0.90477	2.3E-26
dde2/ein2/pad4/sid2:avrRpt2:dde2/pad4/sid2:a	0.97597	1.1E-20
dde2/ein2/pad4/sid2:avrRpt2:dde2/sid2:avrRp	0.98425	1E-53
dde2/ein2/pad4/sid2:avrRpt2:ein2:avrRpt2	0.93519	3E-143
dde2/ein2/pad4/sid2:avrRpt2:ein2/pad4:avrRp	0.71118	3.3E-90
dde2/ein2/pad4/sid2:avrRpt2:ein2/pad4/sid2:a	0.48595	1.2E-23
dde2/ein2/pad4/sid2:avrRpt2:ein2/sid2:avrRpt	0.67884	2.2E-57
dde2/ein2/pad4/sid2:avrRpt2:npr1:avrRpt2	0.83637	2.7E-64
dde2/ein2/pad4/sid2:avrRpt2:pad4:avrRpt2	0.92734	2E-105
dde2/ein2/pad4/sid2:avrRpt2:pad4/sid2:avrRp	0.9402	4E-13
dde2/ein2/pad4/sid2:avrRpt2:rpm1/rps2:avrRp	0.48175	0.00394
dde2/ein2/pad4/sid2:avrRpt2:sid2:avrRpt2	0.86271	1.4E-84
dde2/ein2/sid2:avrRpt2:dde2/pad4:avrRpt2	0.7218	2.3E-11
dde2/ein2/sid2:avrRpt2:dde2/pad4/sid2:avrRp	0.66302	2.1E-16
dde2/ein2/sid2:avrRpt2:dde2/sid2:avrRpt2	0.63039	0.04035
dde2/ein2/sid2:avrRpt2:ein2:avrRpt2	0.69266	3.2E-23
dde2/ein2/sid2:avrRpt2:ein2/pad4:avrRpt2	0.41961	0.00707
dde2/ein2/sid2:avrRpt2:ein2/pad4/sid2:avrRpt	0.26799	3.1E-14
dde2/ein2/sid2:avrRpt2:ein2/sid2:avrRpt2	0.38845	0.26134
dde2/ein2/sid2:avrRpt2:npr1:avrRpt2	0.78171	0.93731
dde2/ein2/sid2:avrRpt2:pad4:avrRpt2	0.70225	7E-07
dde2/ein2/sid2:avrRpt2:pad4/sid2:avrRpt2	0.68975	3E-24
dde2/ein2/sid2:avrRpt2:rpm1/rps2:avrRpt2	0.93412	1.9E-52
dde2/ein2/sid2:avrRpt2:sid2:avrRpt2	0.75992	0.02876
dde2/pad4:avrRpt2:dde2/pad4/sid2:avrRpt2	0.93369	0.13074

dde2/pad4:avrRpt2:dde2/sid2:avrRpt2	0.89716	1.7E-06
dde2/pad4:avrRpt2:ein2:avrRpt2	0.97246	1.9E-59
dde2/pad4:avrRpt2:ein2/pad4:avrRpt2	0.65123	2.5E-20
dde2/pad4:avrRpt2:ein2/pad4/sid2:avrRpt2	0.45128	0.43134
dde2/pad4:avrRpt2:ein2/sid2:avrRpt2	0.61958	1.1E-08
dde2/pad4:avrRpt2:npr1:avrRpt2	0.93444	1.5E-11
dde2/pad4:avrRpt2:pad4:avrRpt2	0.97905	1E-31
dde2/pad4:avrRpt2:pad4/sid2:avrRpt2	0.9673	0.00092
dde2/pad4:avrRpt2:rpm1/rps2:avrRpt2	0.6389	6.9E-17
dde2/pad4:avrRpt2:sid2:avrRpt2	0.96118	9.5E-20
dde2/pad4/sid2:avrRpt2:dde2/sid2:avrRpt2	0.96305	4.7E-10
dde2/pad4/sid2:avrRpt2:ein2:avrRpt2	0.96183	5.9E-65
dde2/pad4/sid2:avrRpt2:ein2/pad4:avrRpt2	0.71031	4E-28
dde2/pad4/sid2:avrRpt2:ein2/pad4/sid2:avrRpt	0.50003	0.46165
dde2/pad4/sid2:avrRpt2:ein2/sid2:avrRpt2	0.68111	3.3E-12
dde2/pad4/sid2:avrRpt2:npr1:avrRpt2	0.86986	1.9E-15
dde2/pad4/sid2:avrRpt2:pad4:avrRpt2	0.9547	2.9E-38
dde2/pad4/sid2:avrRpt2:pad4/sid2:avrRpt2	0.96656	0.06858
dde2/pad4/sid2:avrRpt2:rpm1/rps2:avrRpt2	0.57773	1.5E-11
dde2/pad4/sid2:avrRpt2:sid2:avrRpt2	0.89446	1.8E-25
dde2/sid2:avrRpt2:ein2:avrRpt2	0.92565	3E-32
dde2/sid2:avrRpt2:ein2/pad4:avrRpt2	0.74488	2.1E-06
dde2/sid2:avrRpt2:ein2/pad4/sid2:avrRpt2	0.52973	4.5E-08
dde2/sid2:avrRpt2:ein2/sid2:avrRpt2	0.71474	0.32899
dde2/sid2:avrRpt2:npr1:avrRpt2	0.83422	0.04426
dde2/sid2:avrRpt2:pad4:avrRpt2	0.91808	5.6E-13
dde2/sid2:avrRpt2:pad4/sid2:avrRpt2	0.93015	3.8E-15
dde2/sid2:avrRpt2:rpm1/rps2:avrRpt2	0.54442	4.7E-39
dde2/sid2:avrRpt2:sid2:avrRpt2	0.85796	8.9E-06
ein2:avrRpt2:ein2/pad4:avrRpt2	0.67663	7.2E-13
ein2:avrRpt2:ein2/pad4/sid2:avrRpt2	0.47276	1.4E-61
ein2:avrRpt2:ein2/sid2:avrRpt2	0.64016	2.7E-29
ein2:avrRpt2:npr1:avrRpt2	0.90593	1.5E-24
ein2:avrRpt2:pad4:avrRpt2	0.99339	5E-07
ein2:avrRpt2:pad4/sid2:avrRpt2	0.995	1.1E-79
ein2:avrRpt2:rpm1/rps2:avrRpt2	0.6131	5E-127
ein2:avrRpt2:sid2:avrRpt2	0.93438	1.3E-14
ein2/pad4:avrRpt2:ein2/pad4/sid2:avrRpt2	0.76042	2.7E-25
ein2/pad4:avrRpt2:ein2/sid2:avrRpt2	0.96447	0.0002
ein2/pad4:avrRpt2:npr1:avrRpt2	0.59354	0.00682
ein2/pad4:avrRpt2:pad4:avrRpt2	0.67024	0.01977
ein2/pad4:avrRpt2:pad4/sid2:avrRpt2	0.68281	9.9E-36
ein2/pad4:avrRpt2:rpm1/rps2:avrRpt2	0.34157	2.6E-69
ein2/pad4:avrRpt2:sid2:avrRpt2	0.61477	0.6604
ein2/pad4/sid2:avrRpt2:ein2/sid2:avrRpt2	0.79753	2.3E-10
ein2/pad4/sid2:avrRpt2:npr1:avrRpt2	0.40397	2.2E-13
ein2/pad4/sid2:avrRpt2:pad4:avrRpt2	0.46718	3.9E-34
ein2/pad4/sid2:avrRpt2:pad4/sid2:avrRpt2	0.47897	0.01151
ein2/pad4/sid2:avrRpt2:rpm1/rps2:avrRpt2	0.20246	8.5E-14
ein2/pad4/sid2:avrRpt2:sid2:avrRpt2	0.42014	4.3E-22
ein2/sid2:avrRpt2:npr1:avrRpt2	0.55815	0.28503
ein2/sid2:avrRpt2:pad4:avrRpt2	0.6382	6.6E-10
ein2/sid2:avrRpt2:pad4/sid2:avrRpt2	0.64662	7.5E-19
ein2/sid2:avrRpt2:rpm1/rps2:avrRpt2	0.31464	9.1E-45
ein2/sid2:avrRpt2:sid2:avrRpt2	0.58845	0.00084
npr1:avrRpt2:pad4:avrRpt2	0.9136	2.7E-07
npr1:avrRpt2:pad4/sid2:avrRpt2	0.90151	6.4E-23
npr1:avrRpt2:rpm1/rps2:avrRpt2	0.70453	3.9E-51
npr1:avrRpt2:sid2:avrRpt2	0.97316	0.02108
pad4:avrRpt2:pad4/sid2:avrRpt2	0.98835	2.7E-48
pad4:avrRpt2:rpm1/rps2:avrRpt2	0.6187	1.2E-87
pad4:avrRpt2:sid2:avrRpt2	0.94016	0.00433
pad4/sid2:avrRpt2:rpm1/rps2:avrRpt2	0.60655	9.9E-07

pad4/sid2:avrRpt2:sid2:avrRpt2	0.92903	8.1E-33
rpm1/rps2:avrRpt2:sid2:avrRpt2	0.6781	2.2E-66
