# Supplementary material for: Network Properties of Robust Immunity in Plants
Source: PLoS Genet. 2009 Dec 11;5(12):e1000772. doi: 10.1371/journal.pgen.1000772 (PMC2782137; doi:10.1371/journal.pgen.1000772)
Supplement: Table S3 — P-values for all comparisons in Figure S4B. (0.01 MB PDF) [file pgen.1000772.s011.pdf]

Table S3

Comparisons	0dpi	2dpi
_Col:dde2	0.9594276	0.0105172
_Col:dde2/ein2	0.8336475	0.0013266
_Col:dde2/ein2/pad4	0.8651961	9.966E-13
_Col:dde2/ein2/pad4/sid2	0.615615	1.481E-67
_Col:dde2/ein2/sid2	0.8785727	0.0021128
_Col:dde2/pad4	0.6253373	5.508E-20
_Col:dde2/pad4/sid2	0.7995464	1.702E-24
_Col:dde2/sid2	0.6769981	4.05E-12
_Col:ein2	0.5591145	0.8384655
_Col:ein2/pad4	0.7661419	0.0453311
_Col:ein2/pad4/sid2	0.5134951	8.498E-23
_Col:ein2/sid2	0.6305638	1.724E-08
_Col:npr1	0.6580825	1.322E-06
_Col:pad4	0.7517494	0.0069957
_Col:pad4/sid2	0.8636386	1.545E-31
_Col:rpm1/rps2	0.7873665	6.07E-100
_Col:sid2	0.8619023	0.0001146
dde2:dde2/ein2	0.8844603	0.5770567
dde2:dde2/ein2/pad4	0.9113755	3.357E-05
dde2:dde2/ein2/pad4/sid2	0.7161689	4.267E-30
dde2:dde2/ein2/sid2	0.9238573	0.6771112
dde2:dde2/pad4	0.6860887	5.142E-10
dde2:dde2/pad4/sid2	0.8530756	1.85E-11
dde2:dde2/sid2	0.7385318	6.106E-05
dde2:ein2	0.6169447	0.0240307
dde2:ein2/pad4	0.8216707	0.5588764
dde2:ein2/pad4/sid2	0.5812297	1.648E-10
dde2:ein2/sid2	0.6866823	0.0029572
dde2:npr1	0.7133429	0.0280611
dde2:pad4	0.8062861	0.9106014
dde2:pad4/sid2	0.9099071	4.92E-17
dde2:rpm1/rps2	0.8608615	1.843E-53
dde2:sid2	0.9107319	0.2461775
dde2/ein2:dde2/ein2/pad4	0.9724148	0.0003976
dde2/ein2:dde2/ein2/pad4/sid2	0.8339074	2.293E-28
dde2/ein2:dde2/ein2/sid2	0.9593694	0.8905245
dde2/ein2:dde2/pad4	0.795737	1.416E-08
dde2/ein2:dde2/pad4/sid2	0.9670618	2.438E-10
dde2/ein2:dde2/sid2	0.8478695	0.0004066
dde2/ein2:ein2	0.7273729	0.0056797
dde2/ein2:ein2/pad4	0.935359	0.2484914
dde2/ein2:ein2/pad4/sid2	0.6807013	2.131E-09
dde2/ein2:ein2/sid2	0.8002704	0.0185378
dde2/ein2:npr1	0.8278556	0.1109225
dde2/ein2:pad4	0.920472	0.6549273
dde2/ein2:pad4/sid2	0.9738738	1.087E-14
dde2/ein2:rpm1/rps2	0.9861098	3.19E-49
dde2/ein2:sid2	0.9738843	0.5345964
dde2/ein2/pad4:dde2/ein2/pad4/sid2	0.805635	1.737E-13
dde2/ein2/pad4:dde2/ein2/sid2	0.9870039	0.0001301
dde2/ein2/pad4:dde2/pad4	0.7677448	0.0440751
dde2/ein2/pad4:dde2/pad4/sid2	0.9395936	0.005209
dde2/ein2/pad4:dde2/sid2	0.8217414	0.8841026
dde2/ein2/pad4:ein2	0.6995075	2.909E-10
dde2/ein2/pad4:ein2/pad4	0.909052	2.022E-06
dde2/ein2/pad4:ein2/pad4/sid2	0.660862	0.0180507
dde2/ein2/pad4:ein2/sid2	0.7720348	0.2010678
dde2/ein2/pad4:npr1	0.79955	0.0422742
dde2/ein2/pad4:pad4	0.8922931	5.247E-05
dde2/ein2/pad4:pad4/sid2	0.9985136	8.287E-06
dde2/ein2/pad4:rpm1/rps2	0.9557036	2.04E-30

dde2/ein2/pad4:sid2	0.9982047	0.0029551
dde2/ein2/pad4/sid2:dde2/ein2/sid2	0.7931882	2.617E-29
dde2/ein2/pad4/sid2:dde2/pad4	0.9431845	3.058E-07
dde2/ein2/pad4/sid2:dde2/pad4/sid2	0.8682058	9.178E-06
dde2/ein2/pad4/sid2:dde2/sid2	0.9978601	7.066E-14
dde2/ein2/pad4/sid2:ein2	0.8671524	6.755E-42
dde2/ein2/pad4/sid2:ein2/pad4	0.9023563	1.075E-35
dde2/ein2/pad4/sid2:ein2/pad4/sid2	0.8166798	1.221E-06
dde2/ein2/pad4/sid2:ein2/sid2	0.9485808	2.57E-17
dde2/ein2/pad4/sid2:npr1	0.9790308	2.468E-20
dde2/ein2/pad4/sid2:pad4	0.9192719	1.392E-30
dde2/ein2/pad4/sid2:pad4/sid2	0.8071736	0.0081495
dde2/ein2/pad4/sid2:rpm1/rps2	0.8185063	1.486E-07
dde2/ein2/pad4/sid2:sid2	0.8055175	8.633E-26
dde2/ein2/sid2:dde2/pad4	0.7567536	1.292E-08
dde2/ein2/sid2:dde2/pad4/sid2	0.9271752	9.328E-11
dde2/ein2/sid2:dde2/sid2	0.8103203	0.0004141
dde2/ein2/sid2:ein2	0.6857393	0.0087852
dde2/ein2/sid2:ein2/pad4	0.8957531	0.3095731
dde2/ein2/sid2:ein2/pad4/sid2	0.6474852	8.367E-10
dde2/ein2/sid2:ein2/sid2	0.7582293	0.0127937
dde2/ein2/sid2:npr1	0.785767	0.0835164
dde2/ein2/sid2:pad4	0.8801469	0.7645081
dde2/ein2/sid2:pad4/sid2	0.9855264	2.981E-16
dde2/ein2/sid2:rpm1/rps2	0.9419411	1.331E-51
dde2/ein2/sid2:sid2	0.9853862	0.4637826
dde2/pad4:dde2/pad4/sid2	0.8266176	0.4514205
dde2/pad4:dde2/sid2	0.9453839	0.0272908
dde2/pad4:ein2	0.9281899	5.691E-17
dde2/pad4:ein2/pad4	0.8581718	2.79E-11
dde2/pad4:ein2/pad4/sid2	0.8823886	0.7310434
dde2/pad4:ein2/sid2	0.9955411	0.0008718
dde2/pad4:npr1	0.9671663	4.366E-05
dde2/pad4:pad4	0.8735558	9.331E-10
dde2/pad4:pad4/sid2	0.7691596	0.0203991
dde2/pad4:rpm1/rps2	0.7920106	1.917E-20
dde2/pad4:sid2	0.7694755	3.249E-07
dde2/pad4/sid2:dde2/sid2	0.8796328	0.0033275
dde2/pad4/sid2:ein2	0.7598008	1.496E-18
dde2/pad4/sid2:ein2/pad4	0.9686233	2.548E-14
dde2/pad4/sid2:ein2/pad4/sid2	0.7130153	0.6769809
dde2/pad4/sid2:ein2/sid2	0.8331249	8.067E-05
dde2/pad4/sid2:npr1	0.8607918	2.985E-06
dde2/pad4/sid2:pad4	0.9528636	1.404E-11
dde2/pad4/sid2:pad4/sid2	0.9410572	0.1138083
dde2/pad4/sid2:rpm1/rps2	0.9785066	2.419E-17
dde2/pad4/sid2:sid2	0.9406384	7.067E-09
dde2/sid2:ein2	0.8751376	6.849E-10
dde2/sid2:ein2/pad4	0.9114481	4.349E-06
dde2/sid2:ein2/pad4/sid2	0.8279861	0.0122288
dde2/sid2:ein2/sid2	0.9504993	0.2569998
dde2/sid2:npr1	0.9786604	0.059129
dde2/sid2:pad4	0.9271498	6.809E-05
dde2/sid2:pad4/sid2	0.8231725	9.69E-06
dde2/sid2:rpm1/rps2	0.8501559	3.262E-30
dde2/sid2:sid2	0.8212811	0.0032269
ein2:ein2/pad4	0.7882747	0.1157171
ein2:ein2/pad4/sid2	0.9539692	2.076E-17
ein2:ein2/sid2	0.9228386	1.875E-07
ein2:npr1	0.8943051	8.912E-06
ein2:pad4	0.8032888	0.0200241
ein2:pad4/sid2	0.7008793	1.084E-25
ein2:rpm1/rps2	0.7181526	3.102E-68

ein2:sid2	0.7048289	0.0008278
ein2/pad4:ein2/pad4/sid2	0.7410812	3.32E-13
ein2/pad4:ein2/sid2	0.8627148	0.0006214
ein2/pad4:npr1	0.890736	0.007322
ein2/pad4:pad4	0.9843095	0.485837
ein2/pad4:pad4/sid2	0.9105025	2.46E-19
ein2/pad4:rpm1/rps2	0.9450289	4.597E-57
ein2/pad4:sid2	0.9097413	0.0799035
ein2/pad4/sid2:ein2/sid2	0.8781129	0.0003396
ein2/pad4/sid2:npr1	0.8501649	1.554E-05
ein2/pad4/sid2:pad4	0.7588697	2.765E-10
ein2/pad4/sid2:pad4/sid2	0.662188	0.0480858
ein2/pad4/sid2:rpm1/rps2	0.6739276	9.618E-19
ein2/pad4/sid2:sid2	0.6586207	9.264E-08
ein2/sid2:npr1	0.9712817	0.4364085
ein2/sid2:pad4	0.8780917	0.0050454
ein2/sid2:pad4/sid2	0.7734519	2.426E-08
ein2/sid2:rpm1/rps2	0.7970849	3.074E-36
ein2/sid2:sid2	0.7765862	0.0845364
npr1:pad4	0.9062136	0.0410717
npr1:pad4/sid2	0.800981	2.641E-10
npr1:rpm1/rps2	0.8270118	1.797E-40
npr1:sid2	0.8037912	0.3315222
pad4:pad4/sid2	0.8937574	1.091E-16
pad4:rpm1/rps2	0.92756	3.151E-53
pad4:sid2	0.8940842	0.283908
pad4/sid2:rpm1/rps2	0.9573025	4.933E-12
pad4/sid2:sid2	0.9996724	5.75E-13
rpm1/rps2:sid2	0.9579614	3.979E-46
