# Supplementary material for: Network Properties of Robust Immunity in Plants
Source: PLoS Genet. 2009 Dec 11;5(12):e1000772. doi: 10.1371/journal.pgen.1000772 (PMC2782137; doi:10.1371/journal.pgen.1000772)
Supplement: Table S4 — P-values for all comparisons in Figure S5A. (0.02 MB PDF) [file pgen.1000772.s012.pdf]

Table S4

Comparisons	0dpi	2dpi
_Col:_pLAFR:dde2:_pLAFR	0.787052	0.973065
_Col:_pLAFR:dde2/ein2:_pLAFR	0.891557	0.629199
_Col:_pLAFR:dde2/ein2/pad4:_pLAFR	0.895035	3.63E-17
_Col:_pLAFR:dde2/ein2/pad4/sid2:_pLAFR	0.716954	1.16E-31
_Col:_pLAFR:dde2/ein2/sid2:_pLAFR	0.69226	1.1E-12
_Col:_pLAFR:dde2/pad4:_pLAFR	0.633413	5.3E-08
_Col:_pLAFR:dde2/pad4/sid2:_pLAFR	0.962171	7.92E-10
_Col:_pLAFR:dde2/sid2:_pLAFR	0.82066	0.000359
_Col:_pLAFR:ein2:_pLAFR	0.559893	0.707984
_Col:_pLAFR:ein2/pad4:_pLAFR	0.778533	7.37E-09
_Col:_pLAFR:ein2/pad4/sid2:_pLAFR	0.914717	1.89E-09
_Col:_pLAFR:ein2/sid2:_pLAFR	0.954975	2.43E-05
_Col:_pLAFR:npr1:_pLAFR	0.610555	2.08E-05
_Col:_pLAFR:pad4:_pLAFR	0.84627	0.094863
_Col:_pLAFR:pad4/sid2:_pLAFR	0.989285	2.84E-08
_Col:_pLAFR:rpm1/rps2:_pLAFR	0.459644	0.65813
_Col:_pLAFR:sid2:_pLAFR	0.938912	0.001188
_Col:_pLAFR:_Col:avrRpm1	0.937271	5.9E-187
_Col:_pLAFR:dde2:avrRpm1	0.956711	1.86E-84
_Col:_pLAFR:dde2/ein2:avrRpm1	0.878103	3.82E-62
_Col:_pLAFR:dde2/ein2/pad4:avrRpm1	0.78661	1.71E-35
_Col:_pLAFR:dde2/ein2/pad4/sid2:avrRpm1	0.62427	5.2E-59
_Col:_pLAFR:dde2/ein2/sid2:avrRpm1	0.644279	6.44E-53
_Col:_pLAFR:dde2/pad4:avrRpm1	0.894376	1.27E-55
_Col:_pLAFR:dde2/pad4/sid2:avrRpm1	0.801686	1.25E-58
_Col:_pLAFR:dde2/sid2:avrRpm1	0.841276	3.07E-68
_Col:_pLAFR:ein2:avrRpm1	0.99633	3.3E-90
_Col:_pLAFR:ein2/pad4:avrRpm1	0.947652	1.05E-74
_Col:_pLAFR:ein2/pad4/sid2:avrRpm1	0.741748	5.29E-47
_Col:_pLAFR:ein2/sid2:avrRpm1	0.961464	1.04E-60
_Col:_pLAFR:npr1:avrRpm1	0.631688	9.39E-59
_Col:_pLAFR:pad4:avrRpm1	0.799589	1.84E-98
_Col:_pLAFR:pad4/sid2:avrRpm1	0.828359	1.46E-59
_Col:_pLAFR:rpm1/rps2:avrRpm1	0.70998	0.032793
_Col:_pLAFR:sid2:avrRpm1	0.889945	8.16E-80
dde2:_pLAFR:dde2/ein2:_pLAFR	0.899917	0.633157
dde2:_pLAFR:dde2/ein2/pad4:_pLAFR	0.897486	1.84E-14
dde2:_pLAFR:dde2/ein2/pad4/sid2:_pLAFR	0.571311	5.99E-20
dde2:_pLAFR:dde2/ein2/sid2:_pLAFR	0.530514	7.98E-11
dde2:_pLAFR:dde2/pad4:_pLAFR	0.489226	4.21E-07
dde2:_pLAFR:dde2/pad4/sid2:_pLAFR	0.770485	7.84E-08
dde2:_pLAFR:dde2/sid2:_pLAFR	0.649062	0.001182
dde2:_pLAFR:ein2:_pLAFR	0.421691	0.695227
dde2:_pLAFR:ein2/pad4:_pLAFR	0.994615	3.87E-07
dde2:_pLAFR:ein2/pad4/sid2:_pLAFR	0.878786	1.33E-07
dde2:_pLAFR:ein2/sid2:_pLAFR	0.840465	5.84E-05
dde2:_pLAFR:npr1:_pLAFR	0.462898	5.05E-05
dde2:_pLAFR:pad4:_pLAFR	0.667239	0.126049
dde2:_pLAFR:pad4/sid2:_pLAFR	0.81031	4.9E-07
dde2:_pLAFR:rpm1/rps2:_pLAFR	0.3722	0.745899
dde2:_pLAFR:sid2:_pLAFR	0.750218	0.003246
dde2:_pLAFR:_Col:avrRpm1	0.776088	9E-131
dde2:_pLAFR:dde2:avrRpm1	0.812191	2.41E-68
dde2:_pLAFR:dde2/ein2:avrRpm1	0.746205	1.1E-49
dde2:_pLAFR:dde2/ein2/pad4:avrRpm1	0.9658	2.34E-28
dde2:_pLAFR:dde2/ein2/pad4/sid2:avrRpm1	0.860202	1.02E-38
dde2:_pLAFR:dde2/ein2/sid2:avrRpm1	0.555418	1.7E-42
dde2:_pLAFR:dde2/pad4:avrRpm1	0.938876	2.24E-44
dde2:_pLAFR:dde2/pad4/sid2:avrRpm1	0.979829	9.25E-47
dde2:_pLAFR:dde2/sid2:avrRpm1	0.985121	1.5E-54
dde2:_pLAFR:ein2:avrRpm1	0.845653	4.09E-73

dde2:_pLAFR:ein2/pad4:avrRpm1	0.80419	6.46E-60
dde2:_pLAFR:ein2/pad4/sid2:avrRpm1	0.925701	1.83E-37
dde2:_pLAFR:ein2/sid2:avrRpm1	0.816193	7.85E-49
dde2:_pLAFR:npr1:avrRpm1	0.545252	3.08E-47
dde2:_pLAFR:pad4:avrRpm1	0.977764	1.89E-79
dde2:_pLAFR:pad4/sid2:avrRpm1	0.997052	9.52E-48
dde2:_pLAFR:rpm1/rps2:avrRpm1	0.601712	0.098195
dde2:_pLAFR:sid2:avrRpm1	0.942556	5.33E-64
dde2/ein2:_pLAFR:dde2/ein2/pad4:_pLAFR	0.998451	1.37E-15
dde2/ein2:_pLAFR:dde2/ein2/pad4/sid2:_pLAFR	0.661855	1.14E-22
dde2/ein2:_pLAFR:dde2/ein2/sid2:_pLAFR	0.623032	7.61E-12
dde2/ein2:_pLAFR:dde2/pad4:_pLAFR	0.571359	3.12E-08
dde2/ein2:_pLAFR:dde2/pad4/sid2:_pLAFR	0.865246	2.64E-09
dde2/ein2:_pLAFR:dde2/sid2:_pLAFR	0.737065	0.00015
dde2/ein2:_pLAFR:ein2:_pLAFR	0.505999	0.925518
dde2/ein2:_pLAFR:ein2/pad4:_pLAFR	0.892884	1.57E-08
dde2/ein2:_pLAFR:ein2/pad4/sid2:_pLAFR	0.97832	4.73E-09
dde2/ein2:_pLAFR:ein2/sid2:_pLAFR	0.942335	1.03E-05
dde2/ein2:_pLAFR:npr1:_pLAFR	0.550673	8.85E-06
dde2/ein2:_pLAFR:pad4:_pLAFR	0.760946	0.04453
dde2/ein2:_pLAFR:pad4/sid2:_pLAFR	0.911254	6.42E-08
dde2/ein2:_pLAFR:rpm1/rps2:_pLAFR	0.453393	0.408422
dde2/ein2:_pLAFR:sid2:_pLAFR	0.84402	0.000491
dde2/ein2:_pLAFR:_Col:avrRpm1	0.861614	6.6E-129
dde2/ein2:_pLAFR:dde2:avrRpm1	0.886875	1.07E-66
dde2/ein2:_pLAFR:dde2/ein2:avrRpm1	0.819421	3.88E-48
dde2/ein2:_pLAFR:dde2/ein2/pad4:avrRpm1	0.889303	4.51E-27
dde2/ein2:_pLAFR:dde2/ein2/pad4/sid2:avrRpm1	0.773253	5.5E-37
dde2/ein2:_pLAFR:dde2/ein2/sid2:avrRpm1	0.620318	4.99E-41
dde2/ein2:_pLAFR:dde2/pad4:avrRpm1	0.984491	7.1E-43
dde2/ein2:_pLAFR:dde2/pad4/sid2:avrRpm1	0.903063	3.09E-45
dde2/ein2:_pLAFR:dde2/sid2:avrRpm1	0.938042	5.8E-53
dde2/ein2:_pLAFR:ein2:avrRpm1	0.920981	1.89E-71
dde2/ein2:_pLAFR:ein2/pad4:avrRpm1	0.878919	2.67E-58
dde2/ein2:_pLAFR:ein2/pad4/sid2:avrRpm1	0.849422	4.83E-36
dde2/ein2:_pLAFR:ein2/sid2:avrRpm1	0.89096	2.64E-47
dde2/ein2:_pLAFR:npr1:avrRpm1	0.609577	1E-45
dde2/ein2:_pLAFR:pad4:avrRpm1	0.901075	9.47E-78
dde2/ein2:_pLAFR:pad4/sid2:avrRpm1	0.926317	3.18E-46
dde2/ein2:_pLAFR:rpm1/rps2:avrRpm1	0.67968	0.036809
dde2/ein2:_pLAFR:sid2:avrRpm1	0.980721	2.33E-62
dde2/ein2/pad4:_pLAFR:dde2/ein2/pad4/sid2:_pLAFR	0.665706	0.292616
dde2/ein2/pad4:_pLAFR:dde2/ein2/sid2:_pLAFR	0.621386	0.198588
dde2/ein2/pad4:_pLAFR:dde2/pad4:_pLAFR	0.570031	0.006248
dde2/ein2/pad4:_pLAFR:dde2/pad4/sid2:_pLAFR	0.867941	0.024282
dde2/ein2/pad4:_pLAFR:dde2/sid2:_pLAFR	0.740737	8.44E-06
dde2/ein2/pad4:_pLAFR:ein2:_pLAFR	0.503543	1E-15
dde2/ein2/pad4:_pLAFR:ein2/pad4:_pLAFR	0.89372	0.009507
dde2/ein2/pad4:_pLAFR:ein2/pad4/sid2:_pLAFR	0.980352	0.016935
dde2/ein2/pad4:_pLAFR:ein2/sid2:_pLAFR	0.9434	0.000125
dde2/ein2/pad4:_pLAFR:npr1:_pLAFR	0.548506	0.000143
dde2/ein2/pad4:_pLAFR:pad4:_pLAFR	0.76084	5.52E-10
dde2/ein2/pad4:_pLAFR:pad4/sid2:_pLAFR	0.910815	0.005432
dde2/ein2/pad4:_pLAFR:rpm1/rps2:_pLAFR	0.448101	7.19E-16
dde2/ein2/pad4:_pLAFR:sid2:_pLAFR	0.846886	2.05E-06
dde2/ein2/pad4:_pLAFR:_Col:avrRpm1	0.863248	5.6E-187
dde2/ein2/pad4:_pLAFR:dde2:avrRpm1	0.888219	4E-106
dde2/ein2/pad4:_pLAFR:dde2/ein2:avrRpm1	0.820849	8.3E-85
dde2/ein2/pad4:_pLAFR:dde2/ein2/pad4:avrRpm1	0.888288	1.14E-57
dde2/ein2/pad4:_pLAFR:dde2/ein2/pad4/sid2:avrRpm1	0.772381	4.51E-82
dde2/ein2/pad4:_pLAFR:dde2/ein2/sid2:avrRpm1	0.62189	1.3E-75
dde2/ein2/pad4:_pLAFR:dde2/pad4:avrRpm1	0.983321	2.06E-78
dde2/ein2/pad4:_pLAFR:dde2/pad4/sid2:avrRpm1	0.902022	2.25E-81

dde2/ein2/pad4:_pLAFR:dde2/sid2:avrRpm1	0.936944	1.03E-90
dde2/ein2/pad4:_pLAFR:ein2:avrRpm1	0.922281	1.6E-111
dde2/ein2/pad4:_pLAFR:ein2/pad4:avrRpm1	0.880277	6.66E-97
dde2/ein2/pad4:_pLAFR:ein2/pad4/sid2:avrRpm1	0.848473	9.77E-70
dde2/ein2/pad4:_pLAFR:ein2/sid2:avrRpm1	0.892299	2.44E-83
dde2/ein2/pad4:_pLAFR:npr1:avrRpm1	0.611153	2.05E-81
dde2/ein2/pad4:_pLAFR:pad4:avrRpm1	0.900039	3.9E-119
dde2/ein2/pad4:_pLAFR:pad4/sid2:avrRpm1	0.925242	3.01E-82
dde2/ein2/pad4:_pLAFR:rpm1/rps2:avrRpm1	0.681557	1E-07
dde2/ein2/pad4:_pLAFR:sid2:avrRpm1	0.979557	9.8E-102
dde2/ein2/pad4/sid2:_pLAFR:dde2/ein2/sid2:_pLAFR	0.923663	0.015684
dde2/ein2/pad4/sid2:_pLAFR:dde2/pad4:_pLAFR	0.859386	6.28E-05
dde2/ein2/pad4/sid2:_pLAFR:dde2/pad4/sid2:_pLAFR	0.797941	0.000371
dde2/ein2/pad4/sid2:_pLAFR:dde2/sid2:_pLAFR	0.93947	2.68E-09
dde2/ein2/pad4/sid2:_pLAFR:ein2:_pLAFR	0.777948	1.59E-21
dde2/ein2/pad4/sid2:_pLAFR:ein2/pad4:_pLAFR	0.560987	8.54E-05
dde2/ein2/pad4/sid2:_pLAFR:ein2/pad4/sid2:_pLAFR	0.683065	0.000213
dde2/ein2/pad4/sid2:_pLAFR:ein2/sid2:_pLAFR	0.72351	3.35E-07
dde2/ein2/pad4/sid2:_pLAFR:npr1:_pLAFR	0.834698	4.02E-07
dde2/ein2/pad4/sid2:_pLAFR:pad4:_pLAFR	0.914661	1.01E-14
dde2/ein2/pad4/sid2:_pLAFR:pad4/sid2:_pLAFR	0.754208	5.96E-05
dde2/ein2/pad4/sid2:_pLAFR:rpm1/rps2:_pLAFR	0.702516	4.22E-28
dde2/ein2/pad4/sid2:_pLAFR:sid2:_pLAFR	0.820676	3.55E-10
dde2/ein2/pad4/sid2:_pLAFR:_Col:avrRpm1	0.823333	4.1E-262
dde2/ein2/pad4/sid2:_pLAFR:dde2:avrRpm1	0.849008	1.3E-133
dde2/ein2/pad4/sid2:_pLAFR:dde2/ein2:avrRpm1	0.92652	4.5E-109
dde2/ein2/pad4/sid2:_pLAFR:dde2/ein2/pad4:avrRpm1	0.605878	8.47E-76
dde2/ein2/pad4/sid2:_pLAFR:dde2/ein2/pad4/sid2:avrRpr	0.428212	4.2E-127
dde2/ein2/pad4/sid2:_pLAFR:dde2/ein2/sid2:avrRpm1	0.827962	2.23E-97
dde2/ein2/pad4/sid2:_pLAFR:dde2/pad4:avrRpm1	0.705017	1.9E-101
dde2/ein2/pad4/sid2:_pLAFR:dde2/pad4/sid2:avrRpm1	0.618985	4.1E-105
dde2/ein2/pad4/sid2:_pLAFR:dde2/sid2:avrRpm1	0.655327	2.6E-116
dde2/ein2/pad4/sid2:_pLAFR:ein2:avrRpm1	0.810262	6.4E-140
dde2/ein2/pad4/sid2:_pLAFR:ein2/pad4:avrRpm1	0.857162	1.7E-123
dde2/ein2/pad4/sid2:_pLAFR:ein2/pad4/sid2:avrRpm1	0.565089	7.37E-91
dde2/ein2/pad4/sid2:_pLAFR:ein2/sid2:avrRpm1	0.844334	1.2E-106
dde2/ein2/pad4/sid2:_pLAFR:npr1:avrRpm1	0.814242	2.4E-104
dde2/ein2/pad4/sid2:_pLAFR:pad4:avrRpm1	0.617289	1E-149
dde2/ein2/pad4/sid2:_pLAFR:pad4/sid2:avrRpm1	0.644048	1.2E-105
dde2/ein2/pad4/sid2:_pLAFR:rpm1/rps2:avrRpm1	0.943787	1.34E-14
dde2/ein2/pad4/sid2:_pLAFR:sid2:avrRpm1	0.70063	2.1E-129
dde2/ein2/sid2:_pLAFR:dde2/pad4:_pLAFR	0.941301	0.142541
dde2/ein2/sid2:_pLAFR:dde2/pad4/sid2:_pLAFR	0.748279	0.330148
dde2/ein2/sid2:_pLAFR:dde2/sid2:_pLAFR	0.874803	0.001422
dde2/ein2/sid2:_pLAFR:ein2:_pLAFR	0.859957	6.26E-12
dde2/ein2/sid2:_pLAFR:ein2/pad4:_pLAFR	0.532627	0.178544
dde2/ein2/sid2:_pLAFR:ein2/pad4/sid2:_pLAFR	0.642056	0.255159
dde2/ein2/sid2:_pLAFR:ein2/sid2:_pLAFR	0.670094	0.009027
dde2/ein2/sid2:_pLAFR:npr1:_pLAFR	0.914828	0.009943
dde2/ein2/sid2:_pLAFR:pad4:_pLAFR	0.850789	8.36E-07
dde2/ein2/sid2:_pLAFR:pad4/sid2:_pLAFR	0.70192	0.140853
dde2/ein2/sid2:_pLAFR:rpm1/rps2:_pLAFR	0.824126	2.35E-11
dde2/ein2/sid2:_pLAFR:sid2:_pLAFR	0.76853	0.000494
dde2/ein2/sid2:_pLAFR:_Col:avrRpm1	0.795241	1.4E-177
dde2/ein2/sid2:_pLAFR:dde2:avrRpm1	0.816159	1.2E-99
dde2/ein2/sid2:_pLAFR:dde2/ein2:avrRpm1	0.882488	1.19E-78
dde2/ein2/sid2:_pLAFR:dde2/ein2/pad4:avrRpm1	0.607546	2.52E-52
dde2/ein2/sid2:_pLAFR:dde2/ein2/pad4/sid2:avrRpm1	0.471444	3.42E-74
dde2/ein2/sid2:_pLAFR:dde2/ein2/sid2:avrRpm1	0.904738	9.47E-70
dde2/ein2/sid2:_pLAFR:dde2/pad4:avrRpm1	0.692942	2.12E-72
dde2/ein2/sid2:_pLAFR:dde2/pad4/sid2:avrRpm1	0.619035	2.78E-75
dde2/ein2/sid2:_pLAFR:dde2/sid2:avrRpm1	0.65033	2.05E-84
dde2/ein2/sid2:_pLAFR:ein2:avrRpm1	0.782994	6E-105

dde2/ein2/sid2:_pLAFR:ein2/pad4:avrRpm1	0.823114	1.69E-90
dde2/ein2/sid2:_pLAFR:ein2/pad4/sid2:avrRpm1	0.572323	5.72E-64
dde2/ein2/sid2:_pLAFR:ein2/sid2:avrRpm1	0.812158	2.74E-77
dde2/ein2/sid2:_pLAFR:npr1:avrRpm1	0.892676	2.08E-75
dde2/ein2/sid2:_pLAFR:pad4:avrRpm1	0.61751	2.2E-112
dde2/ein2/sid2:_pLAFR:pad4/sid2:avrRpm1	0.640479	3.45E-76
dde2/ein2/sid2:_pLAFR:rpm1/rps2:avrRpm1	0.982803	2.78E-05
dde2/ein2/sid2:_pLAFR:sid2:avrRpm1	0.689221	3.15E-95
dde2/pad4:_pLAFR:dde2/pad4/sid2:_pLAFR	0.688894	0.613894
dde2/pad4:_pLAFR:dde2/sid2:_pLAFR	0.814725	0.069743
dde2/pad4:_pLAFR:ein2:_pLAFR	0.920192	5.41E-08
dde2/pad4:_pLAFR:ein2/pad4:_pLAFR	0.484738	0.884297
dde2/pad4:_pLAFR:ein2/pad4/sid2:_pLAFR	0.589696	0.727056
dde2/pad4:_pLAFR:ein2/sid2:_pLAFR	0.621775	0.259512
dde2/pad4:_pLAFR:npr1:_pLAFR	0.974682	0.273927
dde2/pad4:_pLAFR:pad4:_pLAFR	0.793196	0.000378
dde2/pad4:_pLAFR:pad4/sid2:_pLAFR	0.647764	0.97034
dde2/pad4:_pLAFR:rpm1/rps2:_pLAFR	0.886498	3.56E-07
dde2/pad4:_pLAFR:sid2:_pLAFR	0.708917	0.033937
dde2/pad4:_pLAFR:_Col:avrRpm1	0.744655	3.7E-166
dde2/pad4:_pLAFR:dde2:avrRpm1	0.77228	5.29E-92
dde2/pad4:_pLAFR:dde2/ein2:avrRpm1	0.837863	1.76E-71
dde2/pad4:_pLAFR:dde2/ein2/pad4:avrRpm1	0.567706	3.17E-46
dde2/pad4:_pLAFR:dde2/ein2/pad4/sid2:avrRpm1	0.430538	4.42E-65
dde2/pad4:_pLAFR:dde2/ein2/sid2:avrRpm1	0.949068	5.81E-63
dde2/pad4:_pLAFR:dde2/pad4:avrRpm1	0.65094	1.99E-65
dde2/pad4:_pLAFR:dde2/pad4/sid2:avrRpm1	0.578771	3.35E-68
dde2/pad4:_pLAFR:dde2/sid2:avrRpm1	0.60926	4.73E-77
dde2/pad4:_pLAFR:ein2:avrRpm1	0.739544	3.35E-97
dde2/pad4:_pLAFR:ein2/pad4:avrRpm1	0.779025	5.43E-83
dde2/pad4:_pLAFR:ein2/pad4/sid2:avrRpm1	0.53346	2.55E-57
dde2/pad4:_pLAFR:ein2/sid2:avrRpm1	0.768326	3.02E-70
dde2/pad4:_pLAFR:npr1:avrRpm1	0.936902	1.99E-68
dde2/pad4:_pLAFR:pad4:avrRpm1	0.577325	2.2E-104
dde2/pad4:_pLAFR:pad4/sid2:avrRpm1	0.599763	3.86E-69
dde2/pad4:_pLAFR:rpm1/rps2:avrRpm1	0.930925	0.004384
dde2/pad4:_pLAFR:sid2:avrRpm1	0.647258	1.4E-87
dde2/pad4/sid2:_pLAFR:dde2/sid2:_pLAFR	0.86715	0.021563
dde2/pad4/sid2:_pLAFR:ein2:_pLAFR	0.621899	9.75E-09
dde2/pad4/sid2:_pLAFR:ein2/pad4:_pLAFR	0.761613	0.714092
dde2/pad4/sid2:_pLAFR:ein2/pad4/sid2:_pLAFR	0.886508	0.87567
dde2/pad4/sid2:_pLAFR:ein2/sid2:_pLAFR	0.924185	0.113747
dde2/pad4/sid2:_pLAFR:npr1:_pLAFR	0.670595	0.121315
dde2/pad4/sid2:_pLAFR:pad4:_pLAFR	0.89129	6.39E-05
dde2/pad4/sid2:_pLAFR:pad4/sid2:_pLAFR	0.955306	0.636177
dde2/pad4/sid2:_pLAFR:rpm1/rps2:_pLAFR	0.567431	1.84E-08
dde2/pad4/sid2:_pLAFR:sid2:_pLAFR	0.978257	0.009301
dde2/pad4/sid2:_pLAFR:_Col:avrRpm1	0.979618	1.5E-172
dde2/pad4/sid2:_pLAFR:dde2:avrRpm1	0.988986	1.82E-95
dde2/pad4/sid2:_pLAFR:dde2/ein2:avrRpm1	0.920698	1.25E-74
dde2/pad4/sid2:_pLAFR:dde2/ein2/pad4:avrRpm1	0.788596	9.29E-49
dde2/pad4/sid2:_pLAFR:dde2/ein2/pad4/sid2:avrRpm1	0.661643	2.55E-69
dde2/pad4/sid2:_pLAFR:dde2/ein2/sid2:avrRpm1	0.713337	6.96E-66
dde2/pad4/sid2:_pLAFR:dde2/pad4:avrRpm1	0.881961	1.87E-68
dde2/pad4/sid2:_pLAFR:dde2/pad4/sid2:avrRpm1	0.801765	2.7E-71
dde2/pad4/sid2:_pLAFR:dde2/sid2:avrRpm1	0.836045	2.53E-80
dde2/pad4/sid2:_pLAFR:ein2:avrRpm1	0.976621	9.6E-101
dde2/pad4/sid2:_pLAFR:ein2/pad4:avrRpm1	0.981239	2.33E-86
dde2/pad4/sid2:_pLAFR:ein2/pad4/sid2:avrRpm1	0.749688	3.71E-60
dde2/pad4/sid2:_pLAFR:ein2/sid2:avrRpm1	0.993116	2.54E-73
dde2/pad4/sid2:_pLAFR:npr1:avrRpm1	0.70196	1.83E-71
dde2/pad4/sid2:_pLAFR:pad4:avrRpm1	0.799914	4.3E-108
dde2/pad4/sid2:_pLAFR:pad4/sid2:avrRpm1	0.824797	3.24E-72

dde2/pad4/sid2:_pLAFR:rpm1/rps2:avrRpm1	0.7913	0.000839
dde2/pad4/sid2:_pLAFR:sid2:avrRpm1	0.878137	4.83E-91
dde2/sid2:_pLAFR:ein2:_pLAFR	0.741517	0.000294
dde2/sid2:_pLAFR:ein2/pad4:_pLAFR	0.638798	0.054641
dde2/sid2:_pLAFR:ein2/pad4/sid2:_pLAFR	0.757495	0.03345
dde2/sid2:_pLAFR:ein2/sid2:_pLAFR	0.796006	0.507883
dde2/sid2:_pLAFR:npr1:_pLAFR	0.793322	0.486897
dde2/sid2:_pLAFR:pad4:_pLAFR	0.976563	0.076251
dde2/sid2:_pLAFR:pad4/sid2:_pLAFR	0.825356	0.072587
dde2/sid2:_pLAFR:rpm1/rps2:_pLAFR	0.693877	0.001531
dde2/sid2:_pLAFR:sid2:_pLAFR	0.888639	0.755926
dde2/sid2:_pLAFR:_Col:avrRpm1	0.904249	1.7E-154
dde2/sid2:_pLAFR:dde2:avrRpm1	0.910783	9.79E-84
dde2/sid2:_pLAFR:dde2/ein2:avrRpm1	0.978719	1E-63
dde2/sid2:_pLAFR:dde2/ein2/pad4:avrRpm1	0.69347	1.17E-39
dde2/sid2:_pLAFR:dde2/ein2/pad4/sid2:avrRpm1	0.559558	1.43E-55
dde2/sid2:_pLAFR:dde2/ein2/sid2:avrRpm1	0.809135	1.3E-55
dde2/sid2:_pLAFR:dde2/pad4:avrRpm1	0.783524	6.89E-58
dde2/sid2:_pLAFR:dde2/pad4/sid2:avrRpm1	0.705867	1.51E-60
dde2/sid2:_pLAFR:dde2/sid2:avrRpm1	0.7389	4.34E-69
dde2/sid2:_pLAFR:ein2:avrRpm1	0.876695	8.11E-89
dde2/sid2:_pLAFR:ein2/pad4:avrRpm1	0.91819	7.19E-75
dde2/sid2:_pLAFR:ein2/pad4/sid2:avrRpm1	0.656141	3.86E-50
dde2/sid2:_pLAFR:ein2/sid2:avrRpm1	0.906679	1.28E-62
dde2/sid2:_pLAFR:npr1:avrRpm1	0.7973	7.26E-61
dde2/sid2:_pLAFR:pad4:avrRpm1	0.704174	9.51E-96
dde2/sid2:_pLAFR:pad4/sid2:avrRpm1	0.72829	1.64E-61
dde2/sid2:_pLAFR:rpm1/rps2:avrRpm1	0.905362	0.208301
dde2/sid2:_pLAFR:sid2:avrRpm1	0.779714	2.59E-79
ein2:_pLAFR:ein2/pad4:_pLAFR	0.425178	5.21E-08
ein2:_pLAFR:ein2/pad4/sid2:_pLAFR	0.523241	1.67E-08
ein2:_pLAFR:ein2/sid2:_pLAFR	0.54693	1.05E-05
ein2:_pLAFR:npr1:_pLAFR	0.944606	8.97E-06
ein2:_pLAFR:pad4:_pLAFR	0.717416	0.05566
ein2:_pLAFR:pad4/sid2:_pLAFR	0.576977	6.53E-08
ein2:_pLAFR:rpm1/rps2:_pLAFR	0.973136	0.463265
ein2:_pLAFR:sid2:_pLAFR	0.640818	0.000896
ein2:_pLAFR:_Col:avrRpm1	0.680007	2.9E-128
ein2:_pLAFR:dde2:avrRpm1	0.715356	1.1E-66
ein2:_pLAFR:dde2/ein2:avrRpm1	0.779172	3.48E-48
ein2:_pLAFR:dde2/ein2/pad4:avrRpm1	0.518347	3.53E-27
ein2:_pLAFR:dde2/ein2/pad4/sid2:avrRpm1	0.382658	5.47E-37
ein2:_pLAFR:dde2/ein2/sid2:avrRpm1	0.990006	4.21E-41
ein2:_pLAFR:dde2/pad4:avrRpm1	0.597891	6.13E-43
ein2:_pLAFR:dde2/pad4/sid2:avrRpm1	0.52877	2.72E-45
ein2:_pLAFR:dde2/sid2:avrRpm1	0.557888	5.41E-53
ein2:_pLAFR:ein2:avrRpm1	0.68357	2.03E-71
ein2:_pLAFR:ein2/pad4:avrRpm1	0.721755	2.6E-58
ein2:_pLAFR:ein2/pad4/sid2:avrRpm1	0.48572	3.98E-36
ein2:_pLAFR:ein2/sid2:avrRpm1	0.711512	2.33E-47
ein2:_pLAFR:npr1:avrRpm1	0.997834	8.74E-46
ein2:_pLAFR:pad4:avrRpm1	0.527434	1.09E-77
ein2:_pLAFR:pad4/sid2:avrRpm1	0.548935	2.8E-46
ein2:_pLAFR:rpm1/rps2:avrRpm1	0.861535	0.046325
ein2:_pLAFR:sid2:avrRpm1	0.594309	2.36E-62
ein2/pad4:_pLAFR:ein2/pad4/sid2:_pLAFR	0.87144	0.832231
ein2/pad4:_pLAFR:ein2/sid2:_pLAFR	0.837691	0.213546
ein2/pad4:_pLAFR:npr1:_pLAFR	0.465901	0.225803
ein2/pad4:_pLAFR:pad4:_pLAFR	0.662146	0.000299
ein2/pad4:_pLAFR:pad4/sid2:_pLAFR	0.807922	0.912425
ein2/pad4:_pLAFR:rpm1/rps2:_pLAFR	0.372208	1.88E-07
ein2/pad4:_pLAFR:sid2:_pLAFR	0.741088	0.026258
ein2/pad4:_pLAFR:_Col:avrRpm1	0.770645	1.4E-169

ein2/pad4:_pLAFR:dde2:avrRpm1	0.807655	1.48E-93
ein2/pad4:_pLAFR:dde2/ein2:avrRpm1	0.741571	7.6E-73
ein2/pad4:_pLAFR:dde2/ein2/pad4:avrRpm1	0.969814	2.96E-47
ein2/pad4:_pLAFR:dde2/ein2/pad4/sid2:avrRpm1	0.864341	4.99E-67
ein2/pad4:_pLAFR:dde2/ein2/sid2:avrRpm1	0.550812	3.37E-64
ein2/pad4:_pLAFR:dde2/pad4:avrRpm1	0.934583	1.01E-66
ein2/pad4:_pLAFR:dde2/pad4/sid2:avrRpm1	0.983898	1.56E-69
ein2/pad4:_pLAFR:dde2/sid2:avrRpm1	0.980948	1.74E-78
ein2/pad4:_pLAFR:ein2:avrRpm1	0.841173	8.4E-99
ein2/pad4:_pLAFR:ein2/pad4:avrRpm1	0.799626	1.75E-84
ein2/pad4:_pLAFR:ein2/pad4/sid2:avrRpm1	0.929589	1.65E-58
ein2/pad4:_pLAFR:ein2/sid2:avrRpm1	0.811663	1.43E-71
ein2/pad4:_pLAFR:npr1:avrRpm1	0.540658	9.93E-70
ein2/pad4:_pLAFR:pad4:avrRpm1	0.981822	4.4E-106
ein2/pad4:_pLAFR:pad4/sid2:avrRpm1	0.992925	1.83E-70
ein2/pad4:_pLAFR:rpm1/rps2:avrRpm1	0.596117	0.002617
ein2/pad4:_pLAFR:sid2:avrRpm1	0.938268	3.96E-89
ein2/pad4/sid2:_pLAFR:ein2/sid2:_pLAFR	0.963635	0.148089
ein2/pad4/sid2:_pLAFR:npr1:_pLAFR	0.56866	0.157453
ein2/pad4/sid2:_pLAFR:pad4:_pLAFR	0.781432	0.000135
ein2/pad4/sid2:_pLAFR:pad4/sid2:_pLAFR	0.93235	0.752082
ein2/pad4/sid2:_pLAFR:rpm1/rps2:_pLAFR	0.470915	5.69E-08
ein2/pad4/sid2:_pLAFR:sid2:_pLAFR	0.865184	0.015219
ein2/pad4/sid2:_pLAFR:_Col:avrRpm1	0.88024	5.4E-171
ein2/pad4/sid2:_pLAFR:dde2:avrRpm1	0.903027	1.5E-94
ein2/pad4/sid2:_pLAFR:dde2/ein2:avrRpm1	0.835379	8.71E-74
ein2/pad4/sid2:_pLAFR:dde2/ein2/pad4:avrRpm1	0.873167	4.63E-48
ein2/pad4/sid2:_pLAFR:dde2/ein2/pad4/sid2:avrRpm1	0.755202	3.31E-68
ein2/pad4/sid2:_pLAFR:dde2/ein2/sid2:avrRpm1	0.634798	4.3E-65
ein2/pad4/sid2:_pLAFR:dde2/pad4:avrRpm1	0.968151	1.23E-67
ein2/pad4/sid2:_pLAFR:dde2/pad4/sid2:avrRpm1	0.886844	1.83E-70
ein2/pad4/sid2:_pLAFR:dde2/sid2:avrRpm1	0.921744	1.89E-79
ein2/pad4/sid2:_pLAFR:ein2:avrRpm1	0.937211	8.2E-100
ein2/pad4/sid2:_pLAFR:ein2/pad4:avrRpm1	0.895097	1.83E-85
ein2/pad4/sid2:_pLAFR:ein2/pad4/sid2:avrRpm1	0.833404	2.19E-59
ein2/pad4/sid2:_pLAFR:ein2/sid2:avrRpm1	0.907124	1.71E-72
ein2/pad4/sid2:_pLAFR:npr1:avrRpm1	0.623948	1.2E-70
ein2/pad4/sid2:_pLAFR:pad4:avrRpm1	0.884877	4E-107
ein2/pad4/sid2:_pLAFR:pad4/sid2:avrRpm1	0.910085	2.18E-71
ein2/pad4/sid2:_pLAFR:rpm1/rps2:avrRpm1	0.697079	0.001398
ein2/pad4/sid2:_pLAFR:sid2:avrRpm1	0.96437	3.98E-90
ein2/sid2:_pLAFR:npr1:_pLAFR	0.594085	0.972764
ein2/sid2:_pLAFR:pad4:_pLAFR	0.81681	0.015339
ein2/sid2:_pLAFR:pad4/sid2:_pLAFR	0.967935	0.250162
ein2/sid2:_pLAFR:rpm1/rps2:_pLAFR	0.498124	0.000105
ein2/sid2:_pLAFR:sid2:_pLAFR	0.903074	0.33525
ein2/sid2:_pLAFR:_Col:avrRpm1	0.912493	5.6E-157
ein2/sid2:_pLAFR:dde2:avrRpm1	0.930858	3.92E-86
ein2/sid2:_pLAFR:dde2/ein2:avrRpm1	0.863139	4.76E-66
ein2/sid2:_pLAFR:dde2/ein2/pad4:avrRpm1	0.846229	1.02E-41
ein2/sid2:_pLAFR:dde2/ein2/pad4/sid2:avrRpm1	0.725797	3.03E-58
ein2/sid2:_pLAFR:dde2/ein2/sid2:avrRpm1	0.660741	7.45E-58
ein2/sid2:_pLAFR:dde2/pad4:avrRpm1	0.940506	3.61E-60
ein2/sid2:_pLAFR:dde2/pad4/sid2:avrRpm1	0.859718	7.52E-63
ein2/sid2:_pLAFR:dde2/sid2:avrRpm1	0.89435	1.9E-71
ein2/sid2:_pLAFR:ein2:avrRpm1	0.96504	3.16E-91
ein2/sid2:_pLAFR:ein2/pad4:avrRpm1	0.923003	2.97E-77
ein2/sid2:_pLAFR:ein2/pad4/sid2:avrRpm1	0.806819	2.42E-52
ein2/sid2:_pLAFR:ein2/sid2:avrRpm1	0.934957	6.47E-65
ein2/sid2:_pLAFR:npr1:avrRpm1	0.649739	3.78E-63
ein2/sid2:_pLAFR:pad4:avrRpm1	0.857792	3.47E-98
ein2/sid2:_pLAFR:pad4/sid2:avrRpm1	0.882845	8.28E-64
ein2/sid2:_pLAFR:rpm1/rps2:avrRpm1	0.728154	0.06607

ein2/sid2:_pLAFR:sid2:avrRpm1	0.936719	1.02E-81
npr1:_pLAFR:pad4:_pLAFR	0.769161	0.013985
npr1:_pLAFR:pad4/sid2:_pLAFR	0.624938	0.264033
npr1:_pLAFR:rpm1/rps2:_pLAFR	0.914046	9.07E-05
npr1:_pLAFR:sid2:_pLAFR	0.690097	0.318911
npr1:_pLAFR:_Col:avrRpm1	0.724668	3.4E-157
npr1:_pLAFR:dde2:avrRpm1	0.754571	2.74E-86
npr1:_pLAFR:dde2/ein2:avrRpm1	0.819501	3.41E-66
npr1:_pLAFR:dde2/ein2/pad4:avrRpm1	0.552651	7.72E-42
npr1:_pLAFR:dde2/ein2/pad4/sid2:avrRpm1	0.416326	2.01E-58
npr1:_pLAFR:dde2/ein2/sid2:avrRpm1	0.968461	5.43E-58
npr1:_pLAFR:dde2/pad4:avrRpm1	0.634622	2.61E-60
npr1:_pLAFR:dde2/pad4/sid2:avrRpm1	0.563505	5.41E-63
npr1:_pLAFR:dde2/sid2:avrRpm1	0.593525	1.35E-71
npr1:_pLAFR:ein2:avrRpm1	0.72218	2.19E-91
npr1:_pLAFR:ein2/pad4:avrRpm1	0.761198	2.09E-77
npr1:_pLAFR:ein2/pad4/sid2:avrRpm1	0.518955	1.77E-52
npr1:_pLAFR:ein2/sid2:avrRpm1	0.750657	4.66E-65
npr1:_pLAFR:npr1:avrRpm1	0.956315	2.73E-63
npr1:_pLAFR:pad4:avrRpm1	0.562094	2.38E-98
npr1:_pLAFR:pad4/sid2:avrRpm1	0.584208	5.96E-64
npr1:_pLAFR:rpm1/rps2:avrRpm1	0.909063	0.061836
npr1:_pLAFR:sid2:avrRpm1	0.630978	7.13E-82
pad4:_pLAFR:pad4/sid2:_pLAFR	0.846801	0.000397
pad4:_pLAFR:rpm1/rps2:_pLAFR	0.669094	0.189461
pad4:_pLAFR:sid2:_pLAFR	0.912711	0.142816
pad4:_pLAFR:_Col:avrRpm1	0.924811	2.7E-142
pad4:_pLAFR:dde2:avrRpm1	0.928522	1.41E-75
pad4:_pLAFR:dde2/ein2:avrRpm1	0.996539	3.3E-56
pad4:_pLAFR:dde2/ein2/pad4:avrRpm1	0.71025	1.69E-33
pad4:_pLAFR:dde2/ein2/pad4/sid2:avrRpm1	0.577526	1.86E-46
pad4:_pLAFR:dde2/ein2/sid2:avrRpm1	0.792112	1.48E-48
pad4:_pLAFR:dde2/pad4:avrRpm1	0.800913	1.26E-50
pad4:_pLAFR:dde2/pad4/sid2:avrRpm1	0.722787	3.76E-53
pad4:_pLAFR:dde2/sid2:avrRpm1	0.756049	2.51E-61
pad4:_pLAFR:ein2:avrRpm1	0.894371	1.64E-80
pad4:_pLAFR:ein2/pad4:avrRpm1	0.935992	6.54E-67
pad4:_pLAFR:ein2/pad4/sid2:avrRpm1	0.672632	2.72E-43
pad4:_pLAFR:ein2/sid2:avrRpm1	0.924413	3.12E-55
pad4:_pLAFR:npr1:avrRpm1	0.780356	1.48E-53
pad4:_pLAFR:pad4:avrRpm1	0.721065	3.76E-87
pad4:_pLAFR:pad4/sid2:avrRpm1	0.745323	3.93E-54
pad4:_pLAFR:rpm1/rps2:avrRpm1	0.885109	0.760021
pad4:_pLAFR:sid2:avrRpm1	0.797099	3.52E-71
pad4/sid2:_pLAFR:rpm1/rps2:_pLAFR	0.523335	2.12E-07
pad4/sid2:_pLAFR:sid2:_pLAFR	0.933958	0.036317
pad4/sid2:_pLAFR:_Col:avrRpm1	0.940268	2.4E-168
pad4/sid2:_pLAFR:dde2:avrRpm1	0.954939	7.35E-93
pad4/sid2:_pLAFR:dde2/ein2:avrRpm1	0.886957	3.29E-72
pad4/sid2:_pLAFR:dde2/ein2/pad4:avrRpm1	0.822258	9.74E-47
pad4/sid2:_pLAFR:dde2/ein2/pad4/sid2:avrRpm1	0.69905	3.52E-66
pad4/sid2:_pLAFR:dde2/ein2/sid2:avrRpm1	0.68243	1.32E-63
pad4/sid2:_pLAFR:dde2/pad4:avrRpm1	0.916197	4.15E-66
pad4/sid2:_pLAFR:dde2/pad4/sid2:avrRpm1	0.83562	6.58E-69
pad4/sid2:_pLAFR:dde2/sid2:avrRpm1	0.87012	7.95E-78
pad4/sid2:_pLAFR:ein2:avrRpm1	0.989224	4.29E-98
pad4/sid2:_pLAFR:ein2/pad4:avrRpm1	0.947126	8.33E-84
pad4/sid2:_pLAFR:ein2/pad4/sid2:avrRpm1	0.783039	6.21E-58
pad4/sid2:_pLAFR:ein2/sid2:avrRpm1	0.959053	6E-71
pad4/sid2:_pLAFR:npr1:avrRpm1	0.671269	4.09E-69
pad4/sid2:_pLAFR:pad4:avrRpm1	0.833724	2.4E-105
pad4/sid2:_pLAFR:pad4/sid2:avrRpm1	0.858717	7.68E-70
pad4/sid2:_pLAFR:rpm1/rps2:avrRpm1	0.75419	0.003674

pad4/sid2:_pLAFR:sid2:avrRpm1	0.912393	1.96E-88
rpm1/rps2:_pLAFR:sid2:_pLAFR	0.587265	0.004428
rpm1/rps2:_pLAFR:_Col:avrRpm1	0.587808	3.6E-188
rpm1/rps2:_pLAFR:dde2:avrRpm1	0.653469	8.34E-86
rpm1/rps2:_pLAFR:dde2/ein2:avrRpm1	0.724864	1.9E-63
rpm1/rps2:_pLAFR:dde2/ein2/pad4:avrRpm1	0.438636	1.3E-36
rpm1/rps2:_pLAFR:dde2/ein2/pad4/sid2:avrRpm1	0.267105	1.21E-60
rpm1/rps2:_pLAFR:dde2/ein2/sid2:avrRpm1	0.966672	3.66E-54
rpm1/rps2:_pLAFR:dde2/pad4:avrRpm1	0.523522	6.73E-57
rpm1/rps2:_pLAFR:dde2/pad4/sid2:avrRpm1	0.449187	6.37E-60
rpm1/rps2:_pLAFR:dde2/sid2:avrRpm1	0.480221	1.44E-69
rpm1/rps2:_pLAFR:ein2:avrRpm1	0.618079	1.46E-91
rpm1/rps2:_pLAFR:ein2/pad4:avrRpm1	0.660226	4.76E-76
rpm1/rps2:_pLAFR:ein2/pad4/sid2:avrRpm1	0.404143	3.17E-48
rpm1/rps2:_pLAFR:ein2/sid2:avrRpm1	0.649176	5.42E-62
rpm1/rps2:_pLAFR:npr1:avrRpm1	0.980725	4.99E-60
rpm1/rps2:_pLAFR:pad4:avrRpm1	0.44794	7.8E-100
rpm1/rps2:_pLAFR:pad4/sid2:avrRpm1	0.47111	7.57E-61
rpm1/rps2:_pLAFR:rpm1/rps2:avrRpm1	0.803721	0.080461
rpm1/rps2:_pLAFR:sid2:avrRpm1	0.519478	3.59E-81
sid2:_pLAFR:_Col:avrRpm1	0.998574	1.9E-152
sid2:_pLAFR:dde2:avrRpm1	0.994654	2.51E-82
sid2:_pLAFR:dde2/ein2:avrRpm1	0.937041	2.04E-62
sid2:_pLAFR:dde2/ein2/pad4:avrRpm1	0.772837	1.43E-38
sid2:_pLAFR:dde2/ein2/pad4/sid2:avrRpm1	0.644504	5.66E-54
sid2:_pLAFR:dde2/ein2/sid2:avrRpm1	0.728683	2.22E-54
sid2:_pLAFR:dde2/pad4:avrRpm1	0.865755	1.28E-56
sid2:_pLAFR:dde2/pad4/sid2:avrRpm1	0.785891	2.94E-59
sid2:_pLAFR:dde2/sid2:avrRpm1	0.820002	9.67E-68
sid2:_pLAFR:ein2:avrRpm1	0.960273	2.19E-87
sid2:_pLAFR:ein2/pad4:avrRpm1	0.997646	1.72E-73
sid2:_pLAFR:ein2/pad4/sid2:avrRpm1	0.734149	6.13E-49
sid2:_pLAFR:ein2/sid2:avrRpm1	0.990524	2.48E-61
sid2:_pLAFR:npr1:avrRpm1	0.71722	1.36E-59
sid2:_pLAFR:pad4:avrRpm1	0.784065	2.86E-94
sid2:_pLAFR:pad4/sid2:avrRpm1	0.808848	3.17E-60
sid2:_pLAFR:rpm1/rps2:avrRpm1	0.809647	0.323729
sid2:_pLAFR:sid2:avrRpm1	0.861929	6.59E-78
_Col:avrRpm1:dde2:avrRpm1	0.990721	0.000598
_Col:avrRpm1:dde2/ein2:avrRpm1	0.91206	1.73E-14
_Col:avrRpm1:dde2/ein2/pad4:avrRpm1	0.683652	4.58E-34
_Col:avrRpm1:dde2/ein2/pad4/sid2:avrRpm1	0.494978	1.55E-71
_Col:avrRpm1:dde2/ein2/sid2:avrRpm1	0.626105	1.65E-18
_Col:avrRpm1:dde2/pad4:avrRpm1	0.809661	2.71E-18
_Col:avrRpm1:dde2/pad4/sid2:avrRpm1	0.698353	1.03E-16
_Col:avrRpm1:dde2/sid2:avrRpm1	0.745195	3.13E-11
_Col:avrRpm1:ein2:avrRpm1	0.942239	0.012012
_Col:avrRpm1:ein2/pad4:avrRpm1	0.998403	3.51E-08
_Col:avrRpm1:ein2/pad4/sid2:avrRpm1	0.626619	2.14E-25
_Col:avrRpm1:ein2/sid2:avrRpm1	0.984894	1.36E-13
_Col:avrRpm1:npr1:avrRpm1	0.610895	9.82E-15
_Col:avrRpm1:pad4:avrRpm1	0.697233	0.097445
_Col:avrRpm1:pad4/sid2:avrRpm1	0.732469	1.14E-14
_Col:avrRpm1:rpm1/rps2:avrRpm1	0.726732	1.2E-216
_Col:avrRpm1:sid2:avrRpm1	0.803426	2.49E-06
dde2:avrRpm1:dde2/ein2:avrRpm1	0.911	0.0003
dde2:avrRpm1:dde2/ein2/pad4:avrRpm1	0.710283	2.15E-16
dde2:avrRpm1:dde2/ein2/pad4/sid2:avrRpm1	0.583161	8.62E-23
dde2:avrRpm1:dde2/ein2/sid2:avrRpm1	0.638407	4.2E-07
dde2:avrRpm1:dde2/pad4:avrRpm1	0.832185	2.59E-06
dde2:avrRpm1:dde2/pad4/sid2:avrRpm1	0.731999	3.43E-05
dde2:avrRpm1:dde2/sid2:avrRpm1	0.775298	0.007115
dde2:avrRpm1:ein2:avrRpm1	0.954306	0.390176

dde2:avrRpm1:ein2/pad4:avrRpm1	0.989938	0.086216
dde2:avrRpm1:ein2/pad4/sid2:avrRpm1	0.664199	1.31E-09
dde2:avrRpm1:ein2/sid2:avrRpm1	0.99451	0.000182
dde2:avrRpm1:npr1:avrRpm1	0.623952	4.74E-05
dde2:avrRpm1:pad4:avrRpm1	0.727149	0.105032
dde2:avrRpm1:pad4/sid2:avrRpm1	0.756652	6.53E-05
dde2:avrRpm1:rpm1/rps2:avrRpm1	0.760353	2.6E-112
dde2:avrRpm1:sid2:avrRpm1	0.828884	0.308472
dde2/ein2:avrRpm1:dde2/ein2/pad4:avrRpm1	0.633444	9.48E-06
dde2/ein2:avrRpm1:dde2/ein2/pad4/sid2:avrRpm1	0.49783	7.22E-10
dde2/ein2:avrRpm1:dde2/ein2/sid2:avrRpm1	0.725802	0.209841
dde2/ein2:avrRpm1:dde2/pad4:avrRpm1	0.745952	0.280349
dde2/ein2:avrRpm1:dde2/pad4/sid2:avrRpm1	0.644911	0.529777
dde2/ein2:avrRpm1:dde2/sid2:avrRpm1	0.687025	0.368506
dde2/ein2:avrRpm1:ein2:avrRpm1	0.866454	9.37E-06
dde2/ein2:avrRpm1:ein2/pad4:avrRpm1	0.919735	0.04688
dde2/ein2:avrRpm1:ein2/pad4/sid2:avrRpm1	0.579123	0.009603
dde2/ein2:avrRpm1:ein2/sid2:avrRpm1	0.905633	0.980618
dde2/ein2:avrRpm1:npr1:avrRpm1	0.710842	0.771158
dde2/ein2:avrRpm1:pad4:avrRpm1	0.644753	1.46E-07
dde2/ein2:avrRpm1:pad4/sid2:avrRpm1	0.677243	0.779723
dde2/ein2:avrRpm1:rpm1/rps2:avrRpm1	0.855479	3.84E-82
dde2/ein2:avrRpm1:sid2:avrRpm1	0.739556	0.008108
dde2/ein2/pad4:avrRpm1:dde2/ein2/pad4/sid2:avrRpm1	0.876852	0.307364
dde2/ein2/pad4:avrRpm1:dde2/ein2/sid2:avrRpm1	0.402188	0.000903
dde2/ein2/pad4:avrRpm1:dde2/pad4:avrRpm1	0.874473	0.000457
dde2/ein2/pad4:avrRpm1:dde2/pad4/sid2:avrRpm1	0.981524	0.000107
dde2/ein2/pad4:avrRpm1:dde2/sid2:avrRpm1	0.935742	9.01E-08
dde2/ein2/pad4:avrRpm1:ein2:avrRpm1	0.753061	1.86E-19
dde2/ein2/pad4:avrRpm1:ein2/pad4:avrRpm1	0.705138	3.36E-10
dde2/ein2/pad4:avrRpm1:ein2/pad4/sid2:avrRpm1	0.947808	0.046983
dde2/ein2/pad4:avrRpm1:ein2/sid2:avrRpm1	0.715375	3.89E-06
dde2/ein2/pad4:avrRpm1:npr1:avrRpm1	0.390957	1.72E-05
dde2/ein2/pad4:avrRpm1:pad4:avrRpm1	0.984115	1.38E-22
dde2/ein2/pad4:avrRpm1:pad4/sid2:avrRpm1	0.950515	1.2E-05
dde2/ein2/pad4:avrRpm1:rpm1/rps2:avrRpm1	0.478157	1.16E-52
dde2/ein2/pad4:avrRpm1:sid2:avrRpm1	0.880385	2.3E-12
dde2/ein2/pad4/sid2:avrRpm1:dde2/ein2/sid2:avrRpm1	0.296411	4.48E-06
dde2/ein2/pad4/sid2:avrRpm1:dde2/pad4:avrRpm1	0.744103	7.06E-07
dde2/ein2/pad4/sid2:avrRpm1:dde2/pad4/sid2:avrRpm1	0.855283	3.12E-08
dde2/ein2/pad4/sid2:avrRpm1:dde2/sid2:avrRpm1	0.806272	5.06E-13
dde2/ein2/pad4/sid2:avrRpm1:ein2:avrRpm1	0.625313	1.19E-26
dde2/ein2/pad4/sid2:avrRpm1:ein2/pad4:avrRpm1	0.568572	1.19E-16
dde2/ein2/pad4/sid2:avrRpm1:ein2/pad4/sid2:avrRpm1	0.932688	0.000826
dde2/ein2/pad4/sid2:avrRpm1:ein2/sid2:avrRpm1	0.588152	2.19E-09
dde2/ein2/pad4/sid2:avrRpm1:npr1:avrRpm1	0.286613	1.7E-08
dde2/ein2/pad4/sid2:avrRpm1:pad4:avrRpm1	0.859048	1.43E-32
dde2/ein2/pad4/sid2:avrRpm1:pad4/sid2:avrRpm1	0.825186	9.69E-09
dde2/ein2/pad4/sid2:avrRpm1:rpm1/rps2:avrRpm1	0.303363	6.55E-79
dde2/ein2/pad4/sid2:avrRpm1:sid2:avrRpm1	0.747718	6.86E-20
dde2/ein2/sid2:avrRpm1:dde2/pad4:avrRpm1	0.499822	0.852342
dde2/ein2/sid2:avrRpm1:dde2/pad4/sid2:avrRpm1	0.423032	0.529208
dde2/ein2/sid2:avrRpm1:dde2/sid2:avrRpm1	0.456751	0.036648
dde2/ein2/sid2:avrRpm1:ein2:avrRpm1	0.598053	3.56E-09
dde2/ein2/sid2:avrRpm1:ein2/pad4:avrRpm1	0.652816	0.0016
dde2/ein2/sid2:avrRpm1:ein2/pad4/sid2:avrRpm1	0.370115	0.217603
dde2/ein2/sid2:avrRpm1:ein2/sid2:avrRpm1	0.633501	0.182692
dde2/ein2/sid2:avrRpm1:npr1:avrRpm1	0.983774	0.31526
dde2/ein2/sid2:avrRpm1:pad4:avrRpm1	0.416979	9.54E-11
dde2/ein2/sid2:avrRpm1:pad4/sid2:avrRpm1	0.437641	0.319469
dde2/ein2/sid2:avrRpm1:rpm1/rps2:avrRpm1	0.8436	5.11E-74
dde2/ein2/sid2:avrRpm1:sid2:avrRpm1	0.499711	0.000172
dde2/pad4:avrRpm1:dde2/pad4/sid2:avrRpm1	0.892912	0.640187

dde2/pad4:avrRpm1:dde2/sid2:avrRpm1	0.938895	0.046312
dde2/pad4:avrRpm1:ein2:avrRpm1	0.876467	3.45E-08
dde2/pad4:avrRpm1:ein2/pad4:avrRpm1	0.822246	0.002692
dde2/pad4:avrRpm1:ein2/pad4/sid2:avrRpm1	0.823622	0.152268
dde2/pad4:avrRpm1:ein2/sid2:avrRpm1	0.837477	0.271381
dde2/pad4:avrRpm1:npr1:avrRpm1	0.487186	0.433609
dde2/pad4:avrRpm1:pad4:avrRpm1	0.890826	2.23E-10
dde2/pad4:avrRpm1:pad4/sid2:avrRpm1	0.923221	0.420603
dde2/pad4:avrRpm1:rpm1/rps2:avrRpm1	0.591804	6.87E-77
dde2/pad4:avrRpm1:sid2:avrRpm1	0.995059	0.000188
dde2/pad4/sid2:avrRpm1:dde2/sid2:avrRpm1	0.953436	0.120435
dde2/pad4/sid2:avrRpm1:ein2:avrRpm1	0.774481	7.74E-07
dde2/pad4/sid2:avrRpm1:ein2/pad4:avrRpm1	0.71822	0.009859
dde2/pad4/sid2:avrRpm1:ein2/pad4/sid2:avrRpm1	0.928427	0.054822
dde2/pad4/sid2:avrRpm1:ein2/sid2:avrRpm1	0.73706	0.531827
dde2/pad4/sid2:avrRpm1:npr1:avrRpm1	0.41164	0.751958
dde2/pad4/sid2:avrRpm1:pad4:avrRpm1	0.997248	2.7E-09
dde2/pad4/sid2:avrRpm1:pad4/sid2:avrRpm1	0.969755	0.741409
dde2/pad4/sid2:avrRpm1:rpm1/rps2:avrRpm1	0.497857	9.24E-79
dde2/pad4/sid2:avrRpm1:sid2:avrRpm1	0.897057	0.000875
dde2/sid2:avrRpm1:ein2:avrRpm1	0.81848	0.000473
dde2/sid2:avrRpm1:ein2/pad4:avrRpm1	0.761979	0.29089
dde2/sid2:avrRpm1:ein2/pad4/sid2:avrRpm1	0.882509	0.000579
dde2/sid2:avrRpm1:ein2/sid2:avrRpm1	0.780449	0.402662
dde2/sid2:avrRpm1:npr1:avrRpm1	0.444838	0.25184
dde2/sid2:avrRpm1:pad4:avrRpm1	0.95103	8.68E-06
dde2/sid2:avrRpm1:pad4/sid2:avrRpm1	0.984403	0.246002
dde2/sid2:avrRpm1:rpm1/rps2:avrRpm1	0.538113	3.35E-90
dde2/sid2:avrRpm1:sid2:avrRpm1	0.943407	0.075004
ein2:avrRpm1:ein2/pad4:avrRpm1	0.944989	0.011155
ein2:avrRpm1:ein2/pad4/sid2:avrRpm1	0.705616	6.42E-12
ein2:avrRpm1:ein2/sid2:avrRpm1	0.959788	4.37E-06
ein2:avrRpm1:npr1:avrRpm1	0.584015	8.79E-07
ein2:avrRpm1:pad4:avrRpm1	0.769922	0.428137
ein2:avrRpm1:pad4/sid2:avrRpm1	0.800263	1.38E-06
ein2:avrRpm1:rpm1/rps2:avrRpm1	0.71418	3.9E-119
ein2:avrRpm1:sid2:avrRpm1	0.872741	0.067813
ein2/pad4:avrRpm1:ein2/pad4/sid2:avrRpm1	0.649869	5.05E-06
ein2/pad4:avrRpm1:ein2/sid2:avrRpm1	0.984536	0.059794
ein2/pad4:avrRpm1:npr1:avrRpm1	0.638455	0.028044
ein2/pad4:avrRpm1:pad4:avrRpm1	0.717535	0.000797
ein2/pad4:avrRpm1:pad4/sid2:avrRpm1	0.75069	0.030535
ein2/pad4:avrRpm1:rpm1/rps2:avrRpm1	0.772623	7.05E-97
ein2/pad4:avrRpm1:sid2:avrRpm1	0.81621	0.486791
ein2/pad4/sid2:avrRpm1:ein2/sid2:avrRpm1	0.669122	0.012115
ein2/pad4/sid2:avrRpm1:npr1:avrRpm1	0.359539	0.028186
ein2/pad4/sid2:avrRpm1:pad4:avrRpm1	0.931646	1.3E-14
ein2/pad4/sid2:avrRpm1:pad4/sid2:avrRpm1	0.899624	0.029512
ein2/pad4/sid2:avrRpm1:rpm1/rps2:avrRpm1	0.44132	5.04E-64
ein2/pad4/sid2:avrRpm1:sid2:avrRpm1	0.827229	2.3E-07
ein2/sid2:avrRpm1:npr1:avrRpm1	0.619094	0.742508
ein2/sid2:avrRpm1:pad4:avrRpm1	0.732244	1.84E-07
ein2/sid2:avrRpm1:pad4/sid2:avrRpm1	0.761851	0.752746
ein2/sid2:avrRpm1:rpm1/rps2:avrRpm1	0.754759	1.2E-83
ein2/sid2:avrRpm1:sid2:avrRpm1	0.834125	0.012056
npr1:avrRpm1:pad4:avrRpm1	0.405585	3.28E-08
npr1:avrRpm1:pad4/sid2:avrRpm1	0.425828	0.993586
npr1:avrRpm1:rpm1/rps2:avrRpm1	0.826629	3.05E-81
npr1:avrRpm1:sid2:avrRpm1	0.487187	0.004827
pad4:avrRpm1:pad4/sid2:avrRpm1	0.966743	1.93E-08
pad4:avrRpm1:rpm1/rps2:avrRpm1	0.493232	5.1E-128
pad4:avrRpm1:sid2:avrRpm1	0.895081	0.00671
pad4/sid2:avrRpm1:rpm1/rps2:avrRpm1	0.520501	2.8E-83

pad4/sid2:avrRpm1:sid2:avrRpm1	0.928757	0.004175
rpm1/rps2:avrRpm1:sid2:avrRpm1	0.589167	8.5E-104
