# Supplementary material for: Network Properties of Robust Immunity in Plants
Source: PLoS Genet. 2009 Dec 11;5(12):e1000772. doi: 10.1371/journal.pgen.1000772 (PMC2782137; doi:10.1371/journal.pgen.1000772)
Supplement: Table S5 — P-values for all comparisons in Figure S5B. (0.01 MB PDF) [file pgen.1000772.s013.pdf]

Table S5

Comparisons	0dpi	2dpi
_Col:dde2	0.58653156	0.00758268
_Col:dde2/ein2	0.85681127	3.0105E-10
_Col:dde2/ein2/pad4	0.82521738	4.26E-06
_Col:dde2/ein2/pad4/sid2	0.45709124	2.24E-10
_Col:dde2/ein2/sid2	0.96374686	0.00928142
_Col:dde2/pad4	0.3432099	5.57E-04
_Col:dde2/pad4/sid2	0.75698618	5.97E-03
_Col:dde2/sid2	0.69504521	3.09E-03
_Col:ein2	0.62952827	0.02761238
_Col:ein2/pad4	0.8365844	0.46937407
_Col:ein2/pad4/sid2	0.79245549	4.98E-06
_Col:ein2/sid2	0.99414347	1.38E-03
_Col:npr1	0.98442482	0.00055702
_Col:pad4	0.66589981	0.81047075
_Col:pad4/sid2	0.79627925	6.67E-03
_Col:rpm1/rps2	0.76552734	2.58E-139
_Col:sid2	0.81654907	0.10211313
dde2:dde2/ein2	0.73461421	0.00152461
dde2:dde2/ein2/pad4	0.4742365	0.07966451
dde2:dde2/ein2/pad4/sid2	0.25038907	4.09E-02
dde2:dde2/ein2/sid2	0.63838575	0.93201815
dde2:dde2/pad4	0.16841082	5.00E-01
dde2:dde2/pad4/sid2	0.43582013	1.00E+00
dde2:dde2/sid2	0.39305225	0.83959827
dde2:ein2	0.33343795	0.66029771
dde2:ein2/pad4	0.75240131	0.07626369
dde2:ein2/pad4/sid2	0.45765033	0.11580868
dde2:ein2/sid2	0.61294419	0.61864864
dde2:npr1	0.59528736	0.46140384
dde2:pad4	0.36705557	0.02673298
dde2:pad4/sid2	0.45289195	9.81E-01
dde2:rpm1/rps2	0.42427534	6.54E-73
dde2:sid2	0.47827516	0.33868836
dde2/ein2:dde2/ein2/pad4	0.71383239	0.15660979
dde2/ein2:dde2/ein2/pad4/sid2	0.42426387	1.43E-01
dde2/ein2:dde2/ein2/sid2	0.90269168	0.00124112
dde2/ein2:dde2/pad4	0.29856834	0.01177357
dde2/ein2:dde2/pad4/sid2	0.65119508	1.35E-03
dde2/ein2:dde2/sid2	0.59719496	0.0024885
dde2/ein2:ein2	0.54145902	0.00032826
dde2/ein2:ein2/pad4	0.98087685	4.0586E-07
dde2/ein2:ein2/pad4/sid2	0.68066125	0.10334326
dde2/ein2:ein2/sid2	0.87483018	0.00711888
dde2/ein2:npr1	0.85528406	0.01384444
dde2/ein2:pad4	0.57326905	7.0139E-08
dde2/ein2:pad4/sid2	0.68832877	1.99E-03
dde2/ein2:rpm1/rps2	0.67024503	1.91E-50
dde2/ein2:sid2	0.70357512	2.4834E-05
dde2/ein2/pad4:dde2/ein2/pad4/sid2	0.69443771	0.877352
dde2/ein2/pad4:dde2/ein2/sid2	0.80278475	0.06397754
dde2/ein2/pad4:dde2/pad4	0.49967499	0.28297928
dde2/ein2/pad4:dde2/pad4/sid2	0.93777938	0.08312368
dde2/ein2/pad4:dde2/sid2	0.87789063	0.12934305
dde2/ein2/pad4:ein2	0.80739339	2.92E-02
dde2/ein2/pad4:ein2/pad4	0.69649254	5.04E-04
dde2/ein2/pad4:ein2/pad4/sid2	0.97268081	0.88453875
dde2/ein2/pad4:ein2/sid2	0.83045622	0.20529911
dde2/ein2/pad4:npr1	0.85008922	0.3014771
dde2/ein2/pad4:pad4	0.84573248	7.0602E-05
dde2/ein2/pad4:pad4/sid2	0.97181644	0.07695951
dde2/ein2/pad4:rpm1/rps2	0.97792454	2.35E-62

dde2/ein2/pad4:sid2	0.99427818	0.00730455
dde2/ein2/pad4/sid2:dde2/ein2/sid2	0.51244237	3.12E-02
dde2/ein2/pad4/sid2:dde2/pad4	0.73925438	0.17731827
dde2/ein2/pad4/sid2:dde2/pad4/sid2	0.75407032	0.03226693
dde2/ein2/pad4/sid2:dde2/sid2	0.81789349	5.86E-02
dde2/ein2/pad4/sid2:ein2	0.89585305	1.21E-02
dde2/ein2/pad4/sid2:ein2/pad4	0.40955381	3.53E-05
dde2/ein2/pad4/sid2:ein2/pad4/sid2	0.71987164	0.74672502
dde2/ein2/pad4/sid2:ein2/sid2	0.53709282	1.29E-01
dde2/ein2/pad4/sid2:npr1	0.55481919	2.05E-01
dde2/ein2/pad4/sid2:pad4	0.85227864	4.89E-06
dde2/ein2/pad4/sid2:pad4/sid2	0.72232858	0.03906491
dde2/ein2/pad4/sid2:rpm1/rps2	0.66022183	7.15E-95
dde2/ein2/pad4/sid2:sid2	0.69569719	1.27E-03
dde2/ein2/sid2:dde2/pad4	0.35943269	4.51E-01
dde2/ein2/sid2:dde2/pad4/sid2	0.74719124	9.35E-01
dde2/ein2/sid2:dde2/sid2	0.69118915	0.77766737
dde2/ein2/sid2:ein2	0.61872213	0.72599865
dde2/ein2/sid2:ein2/pad4	0.88409112	0.08812001
dde2/ein2/sid2:ein2/pad4/sid2	0.77819375	0.0950114
dde2/ein2/sid2:ein2/sid2	0.97133523	0.56271786
dde2/ein2/sid2:npr1	0.951152	0.41477085
dde2/ein2/sid2:pad4	0.65964535	0.03432805
dde2/ein2/sid2:pad4/sid2	0.77581766	9.14E-01
dde2/ein2/sid2:rpm1/rps2	0.76664909	6.69E-74
dde2/ein2/sid2:sid2	0.80096834	0.38486606
dde2/pad4:dde2/pad4/sid2	0.55104526	0.49696519
dde2/pad4:dde2/sid2	0.60315013	0.63422267
dde2/pad4:ein2	0.66892087	2.71E-01
dde2/pad4:ein2/pad4	0.28776367	1.40E-02
dde2/pad4:ein2/pad4/sid2	0.52533836	0.36084251
dde2/pad4:ein2/sid2	0.37822139	0.84782171
dde2/pad4:npr1	0.39182186	0.96808131
dde2/pad4:pad4	0.63307021	3.66E-03
dde2/pad4:pad4/sid2	0.52217348	0.51143127
dde2/pad4:rpm1/rps2	0.48234189	3.82E-69
dde2/pad4:sid2	0.50428548	9.38E-02
dde2/pad4/sid2:dde2/sid2	0.93861071	0.83310939
dde2/pad4/sid2:ein2	0.87147723	6.78E-01
dde2/pad4/sid2:ein2/pad4	0.63417303	6.81E-02
dde2/pad4/sid2:ein2/pad4/sid2	0.96489278	0.10563979
dde2/pad4/sid2:ein2/sid2	0.77380752	0.63870605
dde2/pad4/sid2:npr1	0.79273081	4.87E-01
dde2/pad4/sid2:pad4	0.90788577	2.58E-02
dde2/pad4/sid2:pad4/sid2	0.96539218	0.98168718
dde2/pad4/sid2:rpm1/rps2	0.95424264	5.21E-73
dde2/pad4/sid2:sid2	0.94241651	3.19E-01
dde2/sid2:ein2	0.93077459	0.53481497
dde2/sid2:ein2/pad4	0.58081527	0.04369332
dde2/sid2:ein2/pad4/sid2	0.90417999	0.16228998
dde2/sid2:ein2/sid2	0.71712935	0.78611426
dde2/sid2:npr1	0.73561328	0.61803144
dde2/sid2:pad4	0.96861533	0.01398962
dde2/sid2:pad4/sid2	0.90529787	0.85621032
dde2/sid2:rpm1/rps2	0.88909831	2.63E-71
dde2/sid2:sid2	0.88135826	0.22331741
ein2:ein2/pad4	0.5260053	0.17630425
ein2:ein2/pad4/sid2	0.83617423	4.63E-02
ein2:ein2/sid2	0.64426535	0.34868495
ein2:npr1	0.66252768	0.23967227
ein2:pad4	0.96109615	0.07351069
ein2:pad4/sid2	0.83463557	6.50E-01
ein2:rpm1/rps2	0.81389293	5.47E-76

ein2:sid2	0.81650776	0.59016667
ein2/pad4:ein2/pad4/sid2	0.66318296	4.93E-04
ein2/pad4:ein2/sid2	0.85632919	0.02453497
ein2/pad4:npr1	0.83686673	0.01325893
ein2/pad4:pad4	0.55733248	0.67612661
ein2/pad4:pad4/sid2	0.67122418	7.30E-02
ein2/pad4:rpm1/rps2	0.65185538	4.37E-85
ein2/pad4:sid2	0.6860212	0.40820722
ein2/pad4/sid2:ein2/sid2	0.80536295	0.27303751
ein2/pad4/sid2:npr1	0.82465703	0.38574621
ein2/pad4/sid2:pad4	0.87409638	0.00015705
ein2/pad4/sid2:pad4/sid2	0.99986196	0.12179696
ein2/pad4/sid2:rpm1/rps2	0.99200619	5.23E-61
ein2/pad4/sid2:sid2	0.97797206	0.00971073
ein2/sid2:npr1	0.97979447	0.8112418
ein2/sid2:pad4	0.68540809	0.00699692
ein2/sid2:pad4/sid2	0.80324755	0.64311212
ein2/sid2:rpm1/rps2	0.79614065	1.79E-69
ein2/sid2:sid2	0.8280956	0.15268413
npr1:pad4	0.70379142	0.00341387
npr1:pad4/sid2	0.82272993	4.86E-01
npr1:rpm1/rps2	0.8171065	7.67E-68
npr1:sid2	0.84734176	0.09750206
pad4:pad4/sid2	0.87324467	2.42E-02
pad4:rpm1/rps2	0.85487856	1.36E-90
pad4:sid2	0.85140837	0.20784582
pad4/sid2:rpm1/rps2	0.9917172	1.34E-74
pad4/sid2:sid2	0.97805932	3.23E-01
rpm1/rps2:sid2	0.98437034	7.28E-80
