# Supplementary material for: Network Properties of Robust Immunity in Plants
Source: PLoS Genet. 2009 Dec 11;5(12):e1000772. doi: 10.1371/journal.pgen.1000772 (PMC2782137; doi:10.1371/journal.pgen.1000772)
Supplement: Table S6 — P-values for all comparisons in Figure S6A. (0.02 MB PDF) [file pgen.1000772.s014.pdf]

Table S6

Comparisons	0dpi	2dpi
_Col:_mock:dde2:_mock	0.411	0.708
_Col:_mock:dde2/ein2:_mock	0.194	0.045
_Col:_mock:dde2/ein2/pad4:_mock	0.473	7E-07
_Col:_mock:dde2/ein2/pad4/sid2:_mock	0.247	2E-13
_Col:_mock:dde2/ein2/sid2:_mock	0.527	3E-04
_Col:_mock:dde2/pad4:_mock	0.726	0.007
_Col:_mock:dde2/pad4/sid2:_mock	0.919	2E-04
_Col:_mock:dde2/sid2:_mock	0.472	0.113
_Col:_mock:ein2:_mock	0.681	0.647
_Col:_mock:ein2/pad4:_mock	0.92	0.118
_Col:_mock:ein2/pad4/sid2:_mock	0.755	6E-07
_Col:_mock:ein2/sid2:_mock	0.684	0.291
_Col:_mock:fls2:_mock	0.704	0.655
_Col:_mock:pad4:_mock	0.97	0.497
_Col:_mock:pad4/sid2:_mock	0.715	0.007
_Col:_mock:sid2:_mock	0.447	0.2
_Col:_mock:_Col:flg22	0.932	5E-209
_Col:_mock:dde2:flg22	0.597	4E-96
_Col:_mock:dde2/ein2:flg22	0.784	3E-54
_Col:_mock:dde2/ein2/pad4:flg22	0.853	0.002
_Col:_mock:dde2/ein2/pad4/sid2:flg22	0.863	0.368
_Col:_mock:dde2/ein2/sid2:flg22	0.799	2E-07
_Col:_mock:dde2/pad4:flg22	0.349	1E-22
_Col:_mock:dde2/pad4/sid2:flg22	0.882	2E-19
_Col:_mock:dde2/sid2:flg22	0.795	2E-30
_Col:_mock:ein2:flg22	0.934	2E-73
_Col:_mock:ein2/pad4:flg22	0.891	2E-14
_Col:_mock:ein2/pad4/sid2:flg22	0.926	1E-05
_Col:_mock:ein2/sid2:flg22	0.874	3E-22
_Col:_mock:fls2:flg22	0.956	0.648
_Col:_mock:pad4:flg22	0.975	1E-65
_Col:_mock:pad4/sid2:flg22	0.936	7E-23
_Col:_mock:sid2:flg22	0.885	6E-49
dde2:_mock:dde2/ein2:_mock	0.711	0.198
dde2:_mock:dde2/ein2/pad4:_mock	0.936	3E-05
dde2:_mock:dde2/ein2/pad4/sid2:_mock	0.962	8E-08
dde2:_mock:dde2/ein2/sid2:_mock	0.884	0.002
dde2:_mock:dde2/pad4:_mock	0.715	0.016
dde2:_mock:dde2/pad4/sid2:_mock	0.474	0.001
dde2:_mock:dde2/sid2:_mock	0.937	0.124
dde2:_mock:ein2:_mock	0.723	0.943
dde2:_mock:ein2/pad4:_mock	0.575	0.13
dde2:_mock:ein2/pad4/sid2:_mock	0.693	3E-05
dde2:_mock:ein2/sid2:_mock	0.34	0.263
dde2:_mock:fls2:_mock	0.299	0.478
dde2:_mock:pad4:_mock	0.458	0.363
dde2:_mock:pad4/sid2:_mock	0.723	0.017
dde2:_mock:sid2:_mock	0.958	0.155
dde2:_mock:_Col:flg22	0.38	2E-113
dde2:_mock:dde2:flg22	0.8	3E-73
dde2:_mock:dde2/ein2:flg22	0.671	8E-34
dde2:_mock:dde2/ein2/pad4:flg22	0.622	0.036
dde2:_mock:dde2/ein2/pad4/sid2:flg22	0.48	0.815
dde2:_mock:dde2/ein2/sid2:flg22	0.66	2E-04
dde2:_mock:dde2/pad4:flg22	0.929	8E-14
dde2:_mock:dde2/pad4/sid2:flg22	0.452	1E-11
dde2:_mock:dde2/sid2:flg22	0.402	1E-18
dde2:_mock:ein2:flg22	0.434	1E-54
dde2:_mock:ein2/pad4:flg22	0.596	8E-09
dde2:_mock:ein2/pad4/sid2:flg22	0.572	0.002
dde2:_mock:ein2/sid2:flg22	0.447	1E-13
dde2:_mock:fls2:flg22	0.508	0.475
dde2:_mock:pad4:flg22	0.495	4E-49
dde2:_mock:pad4/sid2:flg22	0.484	6E-14
dde2:_mock:sid2:flg22	0.404	2E-35

dde2/ein2:_mock:dde2/ein2/pad4:_mock	0.653	7E-08
dde2/ein2:_mock:dde2/ein2/pad4/sid2:_mc	0.6	5E-12
dde2/ein2:_mock:dde2/ein2/sid2:_mock	0.606	1E-05
dde2/ein2:_mock:dde2/pad4:_mock	0.413	5E-05
dde2/ein2:_mock:dde2/pad4/sid2:_mock	0.278	7E-06
dde2/ein2:_mock:dde2/sid2:_mock	0.616	0.002
dde2/ein2:_mock:ein2:_mock	0.491	0.222
dde2/ein2:_mock:ein2/pad4:_mock	0.3	0.002
dde2/ein2:_mock:ein2/pad4/sid2:_mock	0.444	6E-08
dde2/ein2:_mock:ein2/sid2:_mock	0.141	0.009
dde2/ein2:_mock:fls2:_mock	0.193	0.055
dde2/ein2:_mock:pad4:_mock	0.3	0.035
dde2/ein2:_mock:pad4/sid2:_mock	0.42	6E-05
dde2/ein2:_mock:sid2:_mock	0.676	0.01
dde2/ein2:_mock:_Col:flg22	0.175	2E-99
dde2/ein2:_mock:dde2:flg22	0.55	6E-53
dde2/ein2:_mock:dde2/ein2:flg22	0.376	2E-32
dde2/ein2:_mock:dde2/ein2/pad4:flg22	0.388	0.43
dde2/ein2:_mock:dde2/ein2/pad4/sid2:flg2	0.237	0.159
dde2/ein2:_mock:dde2/ein2/sid2:flg22	0.418	0.014
dde2/ein2:_mock:dde2/pad4:flg22	0.755	1E-11
dde2/ein2:_mock:dde2/pad4/sid2:flg22	0.262	5E-08
dde2/ein2:_mock:dde2/sid2:flg22	0.178	2E-16
dde2/ein2:_mock:ein2:flg22	0.284	1E-38
dde2/ein2:_mock:ein2/pad4:flg22	0.316	9E-07
dde2/ein2:_mock:ein2/pad4/sid2:flg22	0.35	0.07
dde2/ein2:_mock:ein2/sid2:flg22	0.208	2E-11
dde2/ein2:_mock:fls2:flg22	0.335	0.054
dde2/ein2:_mock:pad4:flg22	0.326	4E-34
dde2/ein2:_mock:pad4/sid2:flg22	0.234	1E-11
dde2/ein2:_mock:sid2:flg22	0.263	7E-24
dde2/ein2/pad4:_mock:dde2/ein2/pad4/sid	0.955	0.943
dde2/ein2/pad4:_mock:dde2/ein2/sid2:_mc	0.941	0.245
dde2/ein2/pad4:_mock:dde2/pad4:_mock	0.775	0.087
dde2/ein2/pad4:_mock:dde2/pad4/sid2:_m	0.478	0.316
dde2/ein2/pad4:_mock:dde2/sid2:_mock	1	0.01
dde2/ein2/pad4:_mock:ein2:_mock	0.812	3E-05
dde2/ein2/pad4:_mock:ein2/pad4:_mock	0.631	0.009
dde2/ein2/pad4:_mock:ein2/pad4/sid2:_mc	0.726	0.977
dde2/ein2/pad4:_mock:ein2/sid2:_mock	0.383	0.003
dde2/ein2/pad4:_mock:fls2:_mock	0.394	5E-04
dde2/ein2/pad4:_mock:pad4:_mock	0.558	8E-04
dde2/ein2/pad4:_mock:pad4/sid2:_mock	0.784	0.082
dde2/ein2/pad4:_mock:sid2:_mock	0.974	0.004
dde2/ein2/pad4:_mock:_Col:flg22	0.438	7E-153
dde2/ein2/pad4:_mock:dde2:flg22	0.883	2E-88
dde2/ein2/pad4:_mock:dde2/ein2:flg22	0.73	4E-56
dde2/ein2/pad4:_mock:dde2/ein2/pad4:flg2	0.645	1E-11
dde2/ein2/pad4:_mock:dde2/ein2/pad4/sid	0.546	3E-08
dde2/ein2/pad4:_mock:dde2/ein2/sid2:flg2	0.688	7E-18
dde2/ein2/pad4:_mock:dde2/pad4:flg22	0.865	5E-30
dde2/ein2/pad4:_mock:dde2/pad4/sid2:flg2	0.454	3E-32
dde2/ein2/pad4:_mock:dde2/sid2:flg22	0.448	1E-36
dde2/ein2/pad4:_mock:ein2:flg22	0.534	5E-71
dde2/ein2/pad4:_mock:ein2/pad4:flg22	0.652	2E-22
dde2/ein2/pad4:_mock:ein2/pad4/sid2:flg2	0.589	2E-15
dde2/ein2/pad4:_mock:ein2/sid2:flg22	0.496	1E-29
dde2/ein2/pad4:_mock:fls2:flg22	0.607	5E-04
dde2/ein2/pad4:_mock:pad4:flg22	0.594	4E-65
dde2/ein2/pad4:_mock:pad4/sid2:flg22	0.536	3E-30
dde2/ein2/pad4:_mock:sid2:flg22	0.503	1E-51
dde2/ein2/pad4/sid2:_mock:dde2/ein2/sid2	0.888	0.158
dde2/ein2/pad4/sid2:_mock:dde2/pad4:_m	0.671	0.024
dde2/ein2/pad4/sid2:_mock:dde2/pad4/sid	0.381	0.212
dde2/ein2/pad4/sid2:_mock:dde2/sid2:_mc	0.956	8E-04
dde2/ein2/pad4/sid2:_mock:ein2:_mock	0.717	5E-08
dde2/ein2/pad4/sid2:_mock:ein2/pad4:_mc	0.5	7E-04

dde2/ein2/pad4/sid2:_mock:ein2/pad4/sid2	0.644	0.969
dde2/ein2/pad4/sid2:_mock:ein2/sid2:_moc	0.237	1E-04
dde2/ein2/pad4/sid2:_mock:fls2:_mock	0.248	5E-06
dde2/ein2/pad4/sid2:_mock:pad4:_mock	0.417	1E-05
dde2/ein2/pad4/sid2:_mock:pad4/sid2:_mc	0.682	0.022
dde2/ein2/pad4/sid2:_mock:sid2:_mock	0.989	2E-04
dde2/ein2/pad4/sid2:_mock:_Col:flg22	0.213	2E-264
dde2/ein2/pad4/sid2:_mock:dde2:flg22	0.806	3E-132
dde2/ein2/pad4/sid2:_mock:dde2/ein2:flg2	0.616	7E-86
dde2/ein2/pad4/sid2:_mock:dde2/ein2/pad	0.556	1E-15
dde2/ein2/pad4/sid2:_mock:dde2/ein2/pad	0.324	2E-16
dde2/ein2/pad4/sid2:_mock:dde2/ein2/sid2	0.603	8E-24
dde2/ein2/pad4/sid2:_mock:dde2/pad4:flg2	0.871	1E-46
dde2/ein2/pad4/sid2:_mock:dde2/pad4/sid	0.357	3E-42
dde2/ein2/pad4/sid2:_mock:dde2/sid2:flg2	0.301	8E-57
dde2/ein2/pad4/sid2:_mock:ein2:flg22	0.391	3E-108
dde2/ein2/pad4/sid2:_mock:ein2/pad4:flg2	0.524	8E-35
dde2/ein2/pad4/sid2:_mock:ein2/pad4/sid2	0.496	3E-20
dde2/ein2/pad4/sid2:_mock:ein2/sid2:flg22	0.351	4E-46
dde2/ein2/pad4/sid2:_mock:fls2:flg22	0.472	5E-06
dde2/ein2/pad4/sid2:_mock:pad4:flg22	0.458	2E-99
dde2/ein2/pad4/sid2:_mock:pad4/sid2:flg2	0.393	6E-47
dde2/ein2/pad4/sid2:_mock:sid2:flg22	0.358	6E-80
dde2/ein2/sid2:_mock:dde2/pad4:_mock	0.826	0.502
dde2/ein2/sid2:_mock:dde2/pad4/sid2:_mc	0.525	0.882
dde2/ein2/sid2:_mock:dde2/sid2:_mock	0.947	0.121
dde2/ein2/sid2:_mock:ein2:_mock	0.863	0.002
dde2/ein2/sid2:_mock:ein2/pad4:_mock	0.679	0.116
dde2/ein2/sid2:_mock:ein2/pad4/sid2:_moc	0.781	0.234
dde2/ein2/sid2:_mock:ein2/sid2:_mock	0.42	0.049
dde2/ein2/sid2:_mock:fls2:_mock	0.432	0.014
dde2/ein2/sid2:_mock:pad4:_mock	0.603	0.022
dde2/ein2/sid2:_mock:pad4/sid2:_mock	0.835	0.485
dde2/ein2/sid2:_mock:sid2:_mock	0.921	0.069
dde2/ein2/sid2:_mock:_Col:flg22	0.49	9E-143
dde2/ein2/sid2:_mock:dde2:flg22	0.935	2E-81
dde2/ein2/sid2:_mock:dde2/ein2:flg22	0.78	4E-50
dde2/ein2/sid2:_mock:dde2/ein2/pad4:flg2	0.699	1E-08
dde2/ein2/sid2:_mock:dde2/ein2/pad4/sid2	0.604	3E-05
dde2/ein2/sid2:_mock:dde2/ein2/sid2:flg22	0.743	6E-14
dde2/ein2/sid2:_mock:dde2/pad4:flg22	0.814	2E-25
dde2/ein2/sid2:_mock:dde2/pad4/sid2:flg2	0.499	6E-27
dde2/ein2/sid2:_mock:dde2/sid2:flg22	0.489	1E-31
dde2/ein2/sid2:_mock:ein2:flg22	0.578	2E-64
dde2/ein2/sid2:_mock:ein2/pad4:flg22	0.7	2E-18
dde2/ein2/sid2:_mock:ein2/pad4/sid2:flg22	0.64	9E-12
dde2/ein2/sid2:_mock:ein2/sid2:flg22	0.539	4E-25
dde2/ein2/sid2:_mock:fls2:flg22	0.654	0.014
dde2/ein2/sid2:_mock:pad4:flg22	0.641	1E-58
dde2/ein2/sid2:_mock:pad4/sid2:flg22	0.58	1E-25
dde2/ein2/sid2:_mock:sid2:flg22	0.546	1E-45
dde2/pad4:_mock:dde2/pad4/sid2:_mock	0.726	0.42
dde2/pad4:_mock:dde2/sid2:_mock	0.75	0.338
dde2/pad4:_mock:ein2:_mock	0.962	0.013
dde2/pad4:_mock:ein2/pad4:_mock	0.828	0.325
dde2/pad4:_mock:ein2/pad4/sid2:_mock	0.976	0.083
dde2/pad4:_mock:ein2/sid2:_mock	0.512	0.155
dde2/pad4:_mock:fls2:_mock	0.571	0.076
dde2/pad4:_mock:pad4:_mock	0.763	0.109
dde2/pad4:_mock:pad4/sid2:_mock	0.99	0.978
dde2/pad4:_mock:sid2:_mock	0.75	0.257
dde2/pad4:_mock:_Col:flg22	0.683	4E-133
dde2/pad4:_mock:dde2:flg22	0.89	1E-75
dde2/pad4:_mock:dde2/ein2:flg22	0.947	3E-54
dde2/pad4:_mock:dde2/ein2/pad4:flg22	0.898	8E-06
dde2/pad4:_mock:dde2/ein2/pad4/sid2:flg2	0.814	1E-03
dde2/pad4:_mock:dde2/ein2/sid2:flg22	0.941	9E-10

dde2/pad4:_mock:dde2/pad4:flg22	0.612	2E-26
dde2/pad4:_mock:dde2/pad4/sid2:flg22	0.699	2E-19
dde2/pad4:_mock:dde2/sid2:flg22	0.598	3E-33
dde2/pad4:_mock:ein2:flg22	0.737	2E-59
dde2/pad4:_mock:ein2/pad4:flg22	0.854	1E-18
dde2/pad4:_mock:ein2/pad4/sid2:flg22	0.842	4E-08
dde2/pad4:_mock:ein2/sid2:flg22	0.66	5E-26
dde2/pad4:_mock:fls2:flg22	0.819	0.077
dde2/pad4:_mock:pad4:flg22	0.805	8E-54
dde2/pad4:_mock:pad4/sid2:flg22	0.71	1E-26
dde2/pad4:_mock:sid2:flg22	0.701	2E-41
dde2/pad4/sid2:_mock:dde2/sid2:_mock	0.524	0.092
dde2/pad4/sid2:_mock:ein2:_mock	0.69	9E-04
dde2/pad4/sid2:_mock:ein2/pad4:_mock	0.876	0.088
dde2/pad4/sid2:_mock:ein2/pad4/sid2:_mo	0.72	0.303
dde2/pad4/sid2:_mock:ein2/sid2:_mock	0.813	0.036
dde2/pad4/sid2:_mock:fls2:_mock	0.829	0.009
dde2/pad4/sid2:_mock:pad4:_mock	0.96	0.015
dde2/pad4/sid2:_mock:pad4/sid2:_mock	0.717	0.405
dde2/pad4/sid2:_mock:sid2:_mock	0.503	0.05
dde2/pad4/sid2:_mock:_Col:flg22	0.964	9E-145
dde2/pad4/sid2:_mock:dde2:flg22	0.625	2E-82
dde2/pad4/sid2:_mock:dde2/ein2:flg22	0.771	6E-51
dde2/pad4/sid2:_mock:dde2/ein2/pad4:flg2	0.804	4E-09
dde2/pad4/sid2:_mock:dde2/ein2/pad4/sid	0.828	1E-05
dde2/pad4/sid2:_mock:dde2/ein2/sid2:flg2	0.758	2E-14
dde2/pad4/sid2:_mock:dde2/pad4:flg22	0.42	5E-26
dde2/pad4/sid2:_mock:dde2/pad4/sid2:flg2	0.968	2E-27
dde2/pad4/sid2:_mock:dde2/sid2:flg22	0.903	3E-32
dde2/pad4/sid2:_mock:ein2:flg22	0.988	2E-65
dde2/pad4/sid2:_mock:ein2/pad4:flg22	0.853	6E-19
dde2/pad4/sid2:_mock:ein2/pad4/sid2:flg2	0.866	4E-12
dde2/pad4/sid2:_mock:ein2/sid2:flg22	0.965	1E-25
dde2/pad4/sid2:_mock:fls2:flg22	0.903	0.009
dde2/pad4/sid2:_mock:pad4:flg22	0.917	1E-59
dde2/pad4/sid2:_mock:pad4/sid2:flg22	0.986	3E-26
dde2/pad4/sid2:_mock:sid2:flg22	0.974	1E-46
dde2/sid2:_mock:ein2:_mock	0.811	0.109
dde2/sid2:_mock:ein2/pad4:_mock	0.593	0.98
dde2/sid2:_mock:ein2/pad4/sid2:_mock	0.753	0.009
dde2/sid2:_mock:ein2/sid2:_mock	0.33	0.649
dde2/sid2:_mock:fls2:_mock	0.394	0.369
dde2/sid2:_mock:pad4:_mock	0.557	0.47
dde2/sid2:_mock:pad4/sid2:_mock	0.759	0.351
dde2/sid2:_mock:sid2:_mock	0.974	0.798
dde2/sid2:_mock:_Col:flg22	0.438	7E-125
dde2/sid2:_mock:dde2:flg22	0.883	5E-70
dde2/sid2:_mock:dde2/ein2:flg22	0.7	9E-49
dde2/sid2:_mock:dde2/ein2/pad4:flg22	0.679	3E-04
dde2/sid2:_mock:dde2/ein2/pad4/sid2:flg2	0.546	0.029
dde2/sid2:_mock:dde2/ein2/sid2:flg22	0.719	1E-07
dde2/sid2:_mock:dde2/pad4:flg22	0.85	2E-22
dde2/sid2:_mock:dde2/pad4/sid2:flg22	0.501	2E-16
dde2/sid2:_mock:dde2/sid2:flg22	0.398	9E-29
dde2/sid2:_mock:ein2:flg22	0.534	4E-54
dde2/sid2:_mock:ein2/pad4:flg22	0.615	3E-15
dde2/sid2:_mock:ein2/pad4/sid2:flg22	0.627	4E-06
dde2/sid2:_mock:ein2/sid2:flg22	0.448	3E-22
dde2/sid2:_mock:fls2:flg22	0.607	0.371
dde2/sid2:_mock:pad4:flg22	0.594	8E-49
dde2/sid2:_mock:pad4/sid2:flg22	0.49	8E-23
dde2/sid2:_mock:sid2:flg22	0.503	7E-37
ein2:_mock:ein2/pad4:_mock	0.809	0.114
ein2:_mock:ein2/pad4/sid2:_mock	0.939	2E-05
ein2:_mock:ein2/sid2:_mock	0.525	0.236
ein2:_mock:fls2:_mock	0.494	0.434
ein2:_mock:pad4:_mock	0.698	0.33

ein2:_mock:pad4/sid2:_mock	0.971	0.014
ein2:_mock:sid2:_mock	0.763	0.132
ein2:_mock:_Col:flg22	0.639	1E-112
ein2:_mock:dde2:flg22	0.919	5E-72
ein2:_mock:dde2/ein2:flg22	0.915	2E-33
ein2:_mock:dde2/ein2/pad4:flg22	0.861	0.043
ein2:_mock:dde2/ein2/pad4/sid2:flg22	0.767	0.881
ein2:_mock:dde2/ein2/sid2:flg22	0.903	2E-04
ein2:_mock:dde2/pad4:flg22	0.683	1E-13
ein2:_mock:dde2/pad4/sid2:flg22	0.664	2E-11
ein2:_mock:dde2/sid2:flg22	0.602	2E-18
ein2:_mock:ein2:flg22	0.669	2E-54
ein2:_mock:ein2/pad4:flg22	0.832	1E-08
ein2:_mock:ein2/pad4/sid2:flg22	0.805	0.002
ein2:_mock:ein2/sid2:flg22	0.658	2E-13
ein2:_mock:fls2:flg22	0.758	0.429
ein2:_mock:pad4:flg22	0.743	8E-49
ein2:_mock:pad4/sid2:flg22	0.703	9E-14
ein2:_mock:sid2:flg22	0.631	4E-35
ein2/pad4:_mock:ein2/pad4/sid2:_mock	0.869	0.008
ein2/pad4:_mock:ein2/sid2:_mock	0.661	0.666
ein2/pad4:_mock:fls2:_mock	0.709	0.38
ein2/pad4:_mock:pad4:_mock	0.915	0.483
ein2/pad4:_mock:pad4/sid2:_mock	0.819	0.34
ein2/pad4:_mock:sid2:_mock	0.608	0.816
ein2/pad4:_mock:_Col:flg22	0.875	4E-125
ein2/pad4:_mock:dde2:flg22	0.739	5E-70
ein2/pad4:_mock:dde2/ein2:flg22	0.88	2E-48
ein2/pad4:_mock:dde2/ein2/pad4:flg22	0.947	3E-04
ein2/pad4:_mock:dde2/ein2/pad4/sid2:flg2	0.988	0.031
ein2/pad4:_mock:dde2/ein2/sid2:flg22	0.905	2E-07
ein2/pad4:_mock:dde2/pad4:flg22	0.469	3E-22
ein2/pad4:_mock:dde2/pad4/sid2:flg22	0.848	3E-16
ein2/pad4:_mock:dde2/sid2:flg22	0.756	8E-29
ein2/pad4:_mock:ein2:flg22	0.887	4E-54
ein2/pad4:_mock:ein2/pad4:flg22	0.974	3E-15
ein2/pad4:_mock:ein2/pad4/sid2:flg22	0.996	4E-06
ein2/pad4:_mock:ein2/sid2:flg22	0.823	8E-22
ein2/pad4:_mock:fls2:flg22	0.973	0.383
ein2/pad4:_mock:pad4:flg22	0.958	9E-49
ein2/pad4:_mock:pad4/sid2:flg22	0.876	2E-22
ein2/pad4:_mock:sid2:flg22	0.85	8E-37
ein2/pad4/sid2:_mock:ein2/sid2:_mock	0.577	0.002
ein2/pad4/sid2:_mock:fls2:_mock	0.591	4E-04
ein2/pad4/sid2:_mock:pad4:_mock	0.786	8E-04
ein2/pad4/sid2:_mock:pad4/sid2:_mock	0.968	0.078
ein2/pad4/sid2:_mock:sid2:_mock	0.728	0.004
ein2/pad4/sid2:_mock:_Col:flg22	0.712	4E-153
ein2/pad4/sid2:_mock:dde2:flg22	0.867	1E-88
ein2/pad4/sid2:_mock:dde2/ein2:flg22	0.976	3E-56
ein2/pad4/sid2:_mock:dde2/ein2/pad4:flg2	0.913	9E-12
ein2/pad4/sid2:_mock:dde2/ein2/pad4/sid2	0.844	3E-08
ein2/pad4/sid2:_mock:dde2/ein2/sid2:flg22	0.96	6E-18
ein2/pad4/sid2:_mock:dde2/pad4:flg22	0.628	4E-30
ein2/pad4/sid2:_mock:dde2/pad4/sid2:flg2	0.691	2E-32
ein2/pad4/sid2:_mock:dde2/sid2:flg22	0.657	9E-37
ein2/pad4/sid2:_mock:ein2:flg22	0.759	3E-71
ein2/pad4/sid2:_mock:ein2/pad4:flg22	0.892	2E-22
ein2/pad4/sid2:_mock:ein2/pad4/sid2:flg22	0.849	2E-15
ein2/pad4/sid2:_mock:ein2/sid2:flg22	0.715	9E-30
ein2/pad4/sid2:_mock:fls2:flg22	0.842	4E-04
ein2/pad4/sid2:_mock:pad4:flg22	0.828	3E-65
ein2/pad4/sid2:_mock:pad4/sid2:flg22	0.761	3E-30
ein2/pad4/sid2:_mock:sid2:flg22	0.723	9E-52
ein2/sid2:_mock:fls2:_mock	0.983	0.63
ein2/sid2:_mock:pad4:_mock	0.774	0.76
ein2/sid2:_mock:pad4/sid2:_mock	0.504	0.168

ein2/sid2:_mock:sid2:_mock	0.365	0.871
ein2/sid2:_mock:_Col:flg22	0.726	5E-121
ein2/sid2:_mock:dde2:flg22	0.467	2E-67
ein2/sid2:_mock:dde2/ein2:flg22	0.556	3E-46
ein2/sid2:_mock:dde2/ein2/pad4:flg22	0.645	0.001
ein2/sid2:_mock:dde2/ein2/pad4/sid2:flg22	0.601	0.098
ein2/sid2:_mock:dde2/ein2/sid2:flg22	0.608	1E-06
ein2/sid2:_mock:dde2/pad4:flg22	0.245	1E-20
ein2/sid2:_mock:dde2/pad4/sid2:flg22	0.84	6E-15
ein2/sid2:_mock:dde2/sid2:flg22	0.898	1E-26
ein2/sid2:_mock:ein2:flg22	0.801	1E-51
ein2/sid2:_mock:ein2/pad4:flg22	0.637	9E-14
ein2/sid2:_mock:ein2/pad4/sid2:flg22	0.698	3E-05
ein2/sid2:_mock:ein2/sid2:flg22	0.83	3E-20
ein2/sid2:_mock:fls2:flg22	0.719	0.634
ein2/sid2:_mock:pad4:flg22	0.733	2E-46
ein2/sid2:_mock:pad4/sid2:flg22	0.777	9E-21
ein2/sid2:_mock:sid2:flg22	0.838	9E-35
fls2:_mock:pad4:_mock	0.767	0.843
fls2:_mock:pad4/sid2:_mock	0.563	0.08
fls2:_mock:sid2:_mock	0.324	0.472
fls2:_mock:_Col:flg22	0.747	9E-119
fls2:_mock:dde2:flg22	0.432	2E-77
fls2:_mock:dde2/ein2:flg22	0.612	7E-37
fls2:_mock:dde2/ein2/pad4:flg22	0.661	0.006
fls2:_mock:dde2/ein2/pad4/sid2:flg22	0.62	0.29
fls2:_mock:dde2/ein2/sid2:flg22	0.622	1E-05
fls2:_mock:dde2/pad4:flg22	0.307	6E-16
fls2:_mock:dde2/pad4/sid2:flg22	0.857	1E-13
fls2:_mock:dde2/sid2:flg22	0.925	6E-21
fls2:_mock:ein2:flg22	0.797	3E-59
fls2:_mock:ein2/pad4:flg22	0.688	2E-10
fls2:_mock:ein2/pad4/sid2:flg22	0.714	2E-04
fls2:_mock:ein2/sid2:flg22	0.863	1E-15
fls2:_mock:fls2:flg22	0.706	0.995
fls2:_mock:pad4:flg22	0.722	1E-53
fls2:_mock:pad4/sid2:flg22	0.816	5E-16
fls2:_mock:sid2:flg22	0.838	8E-39
pad4:_mock:pad4/sid2:_mock	0.755	0.115
pad4:_mock:sid2:_mock	0.49	0.605
pad4:_mock:_Col:flg22	0.984	2E-121
pad4:_mock:dde2:flg22	0.624	7E-79
pad4:_mock:dde2/ein2:flg22	0.809	6E-38
pad4:_mock:dde2/ein2/pad4:flg22	0.863	0.004
pad4:_mock:dde2/ein2/pad4/sid2:flg22	0.879	0.197
pad4:_mock:dde2/ein2/sid2:flg22	0.821	4E-06
pad4:_mock:dde2/pad4:flg22	0.45	1E-16
pad4:_mock:dde2/pad4/sid2:flg22	0.932	3E-14
pad4:_mock:dde2/sid2:flg22	0.863	9E-22
pad4:_mock:ein2:flg22	0.969	2E-60
pad4:_mock:ein2/pad4:flg22	0.892	5E-11
pad4:_mock:ein2/pad4/sid2:flg22	0.919	8E-05
pad4:_mock:ein2/sid2:flg22	0.925	2E-16
pad4:_mock:fls2:flg22	0.936	0.849
pad4:_mock:pad4:flg22	0.952	4E-54
pad4:_mock:pad4/sid2:flg22	0.974	1E-16
pad4:_mock:sid2:flg22	0.926	3E-40
pad4/sid2:_mock:sid2:_mock	0.758	0.268
pad4/sid2:_mock:_Col:flg22	0.673	2E-133
pad4/sid2:_mock:dde2:flg22	0.899	1E-75
pad4/sid2:_mock:dde2/ein2:flg22	0.937	8E-54
pad4/sid2:_mock:dde2/ein2/pad4:flg22	0.889	8E-06
pad4/sid2:_mock:dde2/ein2/pad4/sid2:flg2	0.803	0.001
pad4/sid2:_mock:dde2/ein2/sid2:flg22	0.932	1E-09
pad4/sid2:_mock:dde2/pad4:flg22	0.621	3E-26
pad4/sid2:_mock:dde2/pad4/sid2:flg22	0.691	2E-19
pad4/sid2:_mock:dde2/sid2:flg22	0.589	3E-33

pad4/sid2:_mock:ein2:flg22	0.728	3E-59
pad4/sid2:_mock:ein2/pad4:flg22	0.844	1E-18
pad4/sid2:_mock:ein2/pad4/sid2:flg22	0.833	5E-08
pad4/sid2:_mock:ein2/sid2:flg22	0.651	5E-26
pad4/sid2:_mock:fls2:flg22	0.81	0.081
pad4/sid2:_mock:pad4:flg22	0.796	9E-54
pad4/sid2:_mock:pad4/sid2:flg22	0.701	1E-26
pad4/sid2:_mock:sid2:flg22	0.693	2E-41
sid2:_mock:_Col:flg22	0.413	2E-125
sid2:_mock:dde2:flg22	0.841	8E-82
sid2:_mock:dde2/ein2:flg22	0.705	3E-40
sid2:_mock:dde2/ein2/pad4:flg22	0.655	7E-04
sid2:_mock:dde2/ein2/pad4/sid2:flg22	0.518	0.059
sid2:_mock:dde2/ein2/sid2:flg22	0.694	4E-07
sid2:_mock:dde2/pad4:flg22	0.891	3E-18
sid2:_mock:dde2/pad4/sid2:flg22	0.481	1E-15
sid2:_mock:dde2/sid2:flg22	0.428	1E-23
sid2:_mock:ein2:flg22	0.466	2E-63
sid2:_mock:ein2/pad4:flg22	0.628	2E-12
sid2:_mock:ein2/pad4/sid2:flg22	0.604	1E-05
sid2:_mock:ein2/sid2:flg22	0.475	6E-18
sid2:_mock:fls2:flg22	0.542	0.475
sid2:_mock:pad4:flg22	0.528	2E-57
sid2:_mock:pad4/sid2:flg22	0.514	2E-18
sid2:_mock:sid2:flg22	0.434	9E-43
_Col:flg22:dde2:flg22	0.558	0.005
_Col:flg22:dde2/ein2:flg22	0.741	2E-18
_Col:flg22:dde2/ein2/pad4:flg22	0.808	7E-95
_Col:flg22:dde2/ein2/pad4/sid2:flg22	0.796	3E-202
_Col:flg22:dde2/ein2/sid2:flg22	0.755	2E-80
_Col:flg22:dde2/pad4:flg22	0.32	4E-48
_Col:flg22:dde2/pad4/sid2:flg22	0.928	3E-55
_Col:flg22:dde2/sid2:flg22	0.84	1E-38
_Col:flg22:ein2:flg22	0.98	1E-09
_Col:flg22:ein2/pad4:flg22	0.845	2E-61
_Col:flg22:ein2/pad4/sid2:flg22	0.881	4E-85
_Col:flg22:ein2/sid2:flg22	0.919	2E-48
_Col:flg22:fls2:flg22	0.91	3E-119
_Col:flg22:pad4:flg22	0.929	5E-13
_Col:flg22:pad4/sid2:flg22	0.982	1E-47
_Col:flg22:sid2:flg22	0.931	4E-24
dde2:flg22:dde2/ein2:flg22	0.843	1E-06
dde2:flg22:dde2/ein2/pad4:flg22	0.79	3E-49
dde2:flg22:dde2/ein2/pad4/sid2:flg22	0.679	2E-92
dde2:flg22:dde2/ein2/sid2:flg22	0.831	4E-40
dde2:flg22:dde2/pad4:flg22	0.751	8E-22
dde2:flg22:dde2/pad4/sid2:flg22	0.6	2E-25
dde2:flg22:dde2/sid2:flg22	0.541	1E-16
dde2:flg22:ein2:flg22	0.597	0.004
dde2:flg22:ein2/pad4:flg22	0.761	2E-29
dde2:flg22:ein2/pad4/sid2:flg22	0.735	2E-43
dde2:flg22:ein2/sid2:flg22	0.594	4E-22
dde2:flg22:fls2:flg22	0.682	4E-77
dde2:flg22:pad4:flg22	0.667	1E-04
dde2:flg22:pad4/sid2:flg22	0.636	1E-21
dde2:flg22:sid2:flg22	0.561	2E-10
dde2/ein2:flg22:dde2/ein2/pad4:flg22	0.946	2E-24
dde2/ein2:flg22:dde2/ein2/pad4/sid2:flg22	0.874	2E-50
dde2/ein2:flg22:dde2/ein2/sid2:flg22	0.988	6E-18
dde2/ein2:flg22:dde2/pad4:flg22	0.566	9E-08
dde2/ein2:flg22:dde2/pad4/sid2:flg22	0.744	1E-08
dde2/ein2:flg22:dde2/sid2:flg22	0.645	1E-04
dde2/ein2:flg22:ein2:flg22	0.782	0.024
dde2/ein2:flg22:ein2/pad4:flg22	0.906	6E-13
dde2/ein2:flg22:ein2/pad4/sid2:flg22	0.888	3E-20
dde2/ein2:flg22:ein2/sid2:flg22	0.709	5E-08
dde2/ein2:flg22:fls2:flg22	0.865	6E-37

dde2/ein2:flg22:pad4:flg22	0.851	0.18
dde2/ein2:flg22:pad4/sid2:flg22	0.76	1E-07
dde2/ein2:flg22:sid2:flg22	0.746	0.343
dde2/ein2/pad4:flg22:dde2/ein2/pad4/sid2:	0.944	0.014
dde2/ein2/pad4:flg22:dde2/ein2/sid2:flg22	0.953	0.062
dde2/ein2/pad4:flg22:dde2/pad4:flg22	0.56	5E-08
dde2/ein2/pad4:flg22:dde2/pad4/sid2:flg22	0.773	2E-07
dde2/ein2/pad4:flg22:dde2/sid2:flg22	0.73	1E-11
dde2/ein2/pad4:flg22:ein2:flg22	0.835	2E-35
dde2/ein2/pad4:flg22:ein2/pad4:flg22	0.97	2E-04
dde2/ein2/pad4:flg22:ein2/pad4/sid2:flg22	0.936	0.257
dde2/ein2/pad4:flg22:ein2/sid2:flg22	0.789	7E-08
dde2/ein2/pad4:flg22:fls2:flg22	0.92	0.006
dde2/ein2/pad4:flg22:pad4:flg22	0.905	7E-31
dde2/ein2/pad4:flg22:pad4/sid2:flg22	0.837	4E-08
dde2/ein2/pad4:flg22:sid2:flg22	0.798	4E-21
dde2/ein2/pad4/sid2:flg22:dde2/ein2/sid2:fl	0.889	4E-06
dde2/ein2/pad4/sid2:flg22:dde2/pad4:flg22	0.411	2E-20
dde2/ein2/pad4/sid2:flg22:dde2/pad4/sid2:	0.792	5E-17
dde2/ein2/pad4/sid2:flg22:dde2/sid2:flg22	0.708	1E-27
dde2/ein2/pad4/sid2:flg22:ein2:flg22	0.843	1E-69
dde2/ein2/pad4/sid2:flg22:ein2/pad4:flg22	0.982	1E-12
dde2/ein2/pad4/sid2:flg22:ein2/pad4/sid2:fl	0.982	2E-04
dde2/ein2/pad4/sid2:flg22:ein2/sid2:flg22	0.784	6E-20
dde2/ein2/pad4/sid2:flg22:fls2:flg22	0.952	0.287
dde2/ein2/pad4/sid2:flg22:pad4:flg22	0.934	2E-62
dde2/ein2/pad4/sid2:flg22:pad4/sid2:flg22	0.845	2E-20
dde2/ein2/pad4/sid2:flg22:sid2:flg22	0.795	2E-45
dde2/ein2/sid2:flg22:dde2/pad4:flg22	0.597	1E-04
dde2/ein2/sid2:flg22:dde2/pad4/sid2:flg22	0.728	8E-04
dde2/ein2/sid2:flg22:dde2/sid2:flg22	0.69	2E-07
dde2/ein2/sid2:flg22:ein2:flg22	0.794	2E-27
dde2/ein2/sid2:flg22:ein2/pad4:flg22	0.928	0.04
dde2/ein2/sid2:flg22:ein2/pad4/sid2:flg22	0.889	0.47
dde2/ein2/sid2:flg22:ein2/sid2:flg22	0.749	2E-04
dde2/ein2/sid2:flg22:fls2:flg22	0.877	1E-05
dde2/ein2/sid2:flg22:pad4:flg22	0.863	2E-23
dde2/ein2/sid2:flg22:pad4/sid2:flg22	0.795	1E-04
dde2/ein2/sid2:flg22:sid2:flg22	0.757	6E-15
dde2/pad4:flg22:dde2/pad4/sid2:flg22	0.4	0.432
dde2/pad4:flg22:dde2/sid2:flg22	0.301	0.137
dde2/pad4:flg22:ein2:flg22	0.429	1E-12
dde2/pad4:flg22:ein2/pad4:flg22	0.49	0.053
dde2/pad4:flg22:ein2/pad4/sid2:flg22	0.513	9E-06
dde2/pad4:flg22:ein2/sid2:flg22	0.344	0.936
dde2/pad4:flg22:fls2:flg22	0.494	6E-16
dde2/pad4:flg22:pad4:flg22	0.482	5E-10
dde2/pad4:flg22:pad4/sid2:flg22	0.379	0.964
dde2/pad4:flg22:sid2:flg22	0.401	7E-05
dde2/pad4/sid2:flg22:dde2/sid2:flg22	0.931	0.031
dde2/pad4/sid2:flg22:ein2:flg22	0.96	2E-15
dde2/pad4/sid2:flg22:ein2/pad4:flg22	0.825	0.326
dde2/pad4/sid2:flg22:ein2/pad4/sid2:flg22	0.835	4E-05
dde2/pad4/sid2:flg22:ein2/sid2:flg22	0.993	0.477
dde2/pad4/sid2:flg22:fls2:flg22	0.875	1E-13
dde2/pad4/sid2:flg22:pad4:flg22	0.889	2E-12
dde2/pad4/sid2:flg22:pad4/sid2:flg22	0.958	0.409
dde2/pad4/sid2:flg22:sid2:flg22	0.998	2E-06
dde2/sid2:flg22:ein2:flg22	0.891	7E-09
dde2/sid2:flg22:ein2/pad4:flg22	0.731	6E-04
dde2/sid2:flg22:ein2/pad4/sid2:flg22	0.785	7E-09
dde2/sid2:flg22:ein2/sid2:flg22	0.931	0.116
dde2/sid2:flg22:fls2:flg22	0.807	5E-21
dde2/sid2:flg22:pad4:flg22	0.821	1E-06
dde2/sid2:flg22:pad4/sid2:flg22	0.877	0.148
dde2/sid2:flg22:sid2:flg22	0.929	0.009
ein2:flg22:ein2/pad4:flg22	0.864	1E-18

ein2:flg22:ein2/pad4/sid2:flg22	0.892	2E-30
ein2:flg22:ein2/sid2:flg22	0.953	7E-13
ein2:flg22:fls2:flg22	0.905	5E-59
ein2:flg22:pad4:flg22	0.921	0.31
ein2:flg22:pad4/sid2:flg22	0.998	2E-12
ein2:flg22:sid2:flg22	0.958	4E-04
ein2/pad4:flg22:ein2/pad4/sid2:flg22	0.972	0.007
ein2/pad4:flg22:ein2/sid2:flg22	0.798	0.065
ein2/pad4:flg22:fls2:flg22	0.949	2E-10
ein2/pad4:flg22:pad4:flg22	0.935	2E-15
ein2/pad4:flg22:pad4/sid2:flg22	0.851	0.049
ein2/pad4:flg22:sid2:flg22	0.827	1E-08
ein2/pad4/sid2:flg22:ein2/sid2:flg22	0.845	1E-05
ein2/pad4/sid2:flg22:fls2:flg22	0.977	2E-04
ein2/pad4/sid2:flg22:pad4:flg22	0.962	4E-26
ein2/pad4/sid2:flg22:pad4/sid2:flg22	0.893	7E-06
ein2/pad4/sid2:flg22:sid2:flg22	0.854	4E-17
ein2/sid2:flg22:fls2:flg22	0.868	1E-15
ein2/sid2:flg22:pad4:flg22	0.882	3E-10
ein2/sid2:flg22:pad4/sid2:flg22	0.946	0.9
ein2/sid2:flg22:sid2:flg22	0.991	5E-05
fls2:flg22:pad4:flg22	0.984	2E-53
fls2:flg22:pad4/sid2:flg22	0.916	4E-16
fls2:flg22:sid2:flg22	0.863	5E-39
pad4:flg22:pad4/sid2:flg22	0.931	7E-10
pad4:flg22:sid2:flg22	0.879	0.011
pad4/sid2:flg22:sid2:flg22	0.96	8E-05
