# Supplementary material for: Network Properties of Robust Immunity in Plants
Source: PLoS Genet. 2009 Dec 11;5(12):e1000772. doi: 10.1371/journal.pgen.1000772 (PMC2782137; doi:10.1371/journal.pgen.1000772)
Supplement: Table S7 — P-values for all comparisons in Figure S6B. (0.01 MB PDF) [file pgen.1000772.s015.pdf]

Table S7

Comparisons	0dpi	2dpi
_Col:dde2	0.86015	0.0178
_Col:dde2/ein2	0.46889	1E-15
_Col:dde2/ein2/pad4	0.72188	9.9E-37
_Col:dde2/ein2/pad4/sid2	0.52391	2.7E-83
_Col:dde2/ein2/sid2	0.80964	7.3E-34
_Col:dde2/pad4	0.62976	4.3E-20
_Col:dde2/pad4/sid2	0.99325	2.5E-20
_Col:dde2/sid2	0.49052	3.5E-18
_Col:ein2	0.74336	9.8E-07
_Col:ein2/pad4	0.94324	6.9E-31
_Col:ein2/pad4/sid2	0.9033	6.3E-32
_Col:ein2/sid2	0.81868	3.3E-25
_Col:fls2	0.71197	2.4E-71
_Col:pad4	0.92441	8.5E-07
_Col:pad4/sid2	0.77168	5.5E-20
_Col:sid2	0.52549	3.2E-11
dde2:dde2/ein2	0.65439	1.9E-06
dde2:dde2/ein2/pad4	0.88329	5.8E-18
dde2:dde2/ein2/pad4/sid2	0.78364	5.5E-34
dde2:dde2/ein2/sid2	0.95786	3.1E-16
dde2:dde2/pad4	0.59098	1.1E-08
dde2:dde2/pad4/sid2	0.88017	9E-09
dde2:dde2/sid2	0.67504	9.5E-08
dde2:ein2	0.90172	0.03617
dde2:ein2/pad4	0.83993	9.4E-15
dde2:ein2/pad4/sid2	0.96438	3.5E-15
dde2:ein2/sid2	0.74063	1.4E-11
dde2:fls2	0.65611	7.3E-40
dde2:pad4	0.82485	0.03412
dde2:pad4/sid2	0.92584	1.1E-08
dde2:sid2	0.70792	0.00039
dde2/ein2:dde2/ein2/pad4	0.76348	0.0001
dde2/ein2:dde2/ein2/pad4/sid2	0.78428	1.4E-10
dde2/ein2:dde2/ein2/sid2	0.69296	0.00068
dde2/ein2:dde2/pad4	0.32481	0.34216
dde2/ein2:dde2/pad4/sid2	0.5496	0.34631
dde2/ein2:dde2/sid2	0.9773	0.5692
dde2/ein2:ein2	0.74577	0.00692
dde2/ein2:ein2/pad4	0.51599	0.00266
dde2/ein2:ein2/pad4/sid2	0.6225	0.00204
dde2/ein2:ein2/sid2	0.4363	0.04455
dde2/ein2:fls2	0.37207	7.2E-18
dde2/ein2:pad4	0.50359	0.00746
dde2/ein2:pad4/sid2	0.72288	0.34676
dde2/ein2:sid2	0.94177	0.20706
dde2/ein2/pad4:dde2/ein2/pad4/sid2	0.92447	0.07902
dde2/ein2/pad4:dde2/ein2/sid2	0.92515	0.61664
dde2/ein2/pad4:dde2/pad4	0.4939	0.00332
dde2/ein2/pad4:dde2/pad4/sid2	0.76605	0.00312
dde2/ein2/pad4:dde2/sid2	0.78526	0.00093
dde2/ein2/pad4:ein2	0.9814	4.1E-11
dde2/ein2/pad4:ein2/pad4	0.72727	0.38703
dde2/ein2/pad4:ein2/pad4/sid2	0.84817	0.40932
dde2/ein2/pad4:ein2/sid2	0.63281	0.06129
dde2/ein2/pad4:fls2	0.55385	1.2E-06
dde2/ein2/pad4:pad4	0.71281	5E-11
dde2/ein2/pad4:pad4/sid2	0.95716	0.0032
dde2/ein2/pad4:sid2	0.81975	2.3E-07
dde2/ein2/pad4/sid2:dde2/ein2/sid2	0.83377	0.01795
dde2/ein2/pad4/sid2:dde2/pad4	0.35114	1.1E-07
dde2/ein2/pad4/sid2:dde2/pad4/sid2	0.64611	7.4E-08

dde2/ein2/pad4/sid2:dde2/sid2	0.81118	9.2E-09
dde2/ein2/pad4/sid2:ein2	0.90183	2.3E-22
dde2/ein2/pad4/sid2:ein2/pad4	0.60175	0.00514
dde2/ein2/pad4/sid2:ein2/pad4/sid2	0.74196	0.00555
dde2/ein2/pad4/sid2:ein2/sid2	0.49661	6.1E-05
dde2/ein2/pad4/sid2:fls2	0.41241	2.8E-05
dde2/ein2/pad4/sid2:pad4	0.58538	3.3E-22
dde2/ein2/pad4/sid2:pad4/sid2	0.87242	1.1E-07
dde2/ein2/pad4/sid2:sid2	0.85379	9.7E-16
dde2/ein2/sid2:dde2/pad4	0.55504	0.01434
dde2/ein2/sid2:dde2/pad4/sid2	0.83867	0.01335
dde2/ein2/sid2:dde2/sid2	0.71406	0.00474
dde2/ein2/sid2:ein2	0.94368	9.2E-10
dde2/ein2/sid2:ein2/pad4	0.79886	0.70966
dde2/ein2/sid2:ein2/pad4/sid2	0.92233	0.74512
dde2/ein2/sid2:ein2/sid2	0.70109	0.16709
dde2/ein2/sid2:fls2	0.61841	8.7E-08
dde2/ein2/sid2:pad4	0.78397	1.1E-09
dde2/ein2/sid2:pad4/sid2	0.9679	0.01388
dde2/ein2/sid2:sid2	0.74759	2.7E-06
dde2/pad4:dde2/pad4/sid2	0.69896	0.9876
dde2/pad4:dde2/sid2	0.33896	0.70608
dde2/pad4:ein2	0.50873	0.00025
dde2/pad4:ein2/pad4	0.73724	0.03869
dde2/pad4:ein2/pad4/sid2	0.62216	0.03303
dde2/pad4:ein2/sid2	0.83642	0.28898
dde2/pad4:fls2	0.92656	1.3E-14
dde2/pad4:pad4	0.75186	0.00028
dde2/pad4:pad4/sid2	0.5284	0.99059
dde2/pad4:sid2	0.36192	0.02637
dde2/pad4/sid2:dde2/sid2	0.56873	0.71461
dde2/pad4/sid2:ein2	0.7839	0.00024
dde2/pad4/sid2:ein2/pad4	0.95915	0.03636
dde2/pad4/sid2:ein2/pad4/sid2	0.9155	0.03093
dde2/pad4/sid2:ein2/sid2	0.85694	0.2797
dde2/pad4/sid2:fls2	0.76833	7.4E-15
dde2/pad4/sid2:pad4	0.94375	0.00027
dde2/pad4/sid2:pad4/sid2	0.80736	0.99708
dde2/pad4/sid2:sid2	0.59934	0.02623
dde2/sid2:ein2	0.7674	0.00106
dde2/sid2:ein2/pad4	0.53453	0.01517
dde2/sid2:ein2/pad4/sid2	0.64274	0.0121
dde2/sid2:ein2/sid2	0.45323	0.15307
dde2/sid2:fls2	0.38748	7.7E-16
dde2/sid2:pad4	0.52189	0.00116
dde2/sid2:pad4/sid2	0.74429	0.71482
dde2/sid2:sid2	0.96442	0.06618
ein2:ein2/pad4	0.74484	1.1E-08
ein2:ein2/pad4/sid2	0.86647	6E-09
ein2:ein2/sid2	0.64949	2.5E-06
ein2:fls2	0.56955	2.7E-29
ein2:pad4	0.73026	0.98047
ein2:pad4/sid2	0.97575	0.00027
ein2:sid2	0.80167	0.14445
ein2/pad4:ein2/pad4/sid2	0.87499	0.9597
ein2/pad4:ein2/sid2	0.89731	0.31472
ein2/pad4:fls2	0.80773	1.4E-08
ein2/pad4:pad4	0.98457	1.3E-08
ein2/pad4:pad4/sid2	0.76795	0.03816
ein2/pad4:sid2	0.56425	1.8E-05
ein2/pad4/sid2:ein2/sid2	0.77459	0.28801
ein2/pad4/sid2:fls2	0.68868	1.3E-08
ein2/pad4/sid2:pad4	0.85977	7.1E-09

ein2/pad4/sid2:pad4/sid2	0.89045	0.03207
ein2/pad4/sid2:sid2	0.67501	1.2E-05
ein2/sid2:fls2	0.909	2.8E-11
ein2/sid2:pad4	0.91263	2.8E-06
ein2/sid2:pad4/sid2	0.67151	0.28505
ein2/sid2:sid2	0.48051	0.00104
fls2:pad4	0.82274	2.6E-29
fls2:pad4/sid2	0.59035	1.2E-14
fls2:sid2	0.41241	4.1E-23
pad4:pad4/sid2	0.75323	0.00029
pad4:sid2	0.55127	0.15538
pad4/sid2:sid2	0.77827	0.02718
