# Supplementary material for: Network Properties of Robust Immunity in Plants
Source: PLoS Genet. 2009 Dec 11;5(12):e1000772. doi: 10.1371/journal.pgen.1000772 (PMC2782137; doi:10.1371/journal.pgen.1000772)
Supplement: Table S8 — P-values for all comparisons in Figure S7A. (0.02 MB PDF) [file pgen.1000772.s016.pdf]

Table S8

Comparisons	0dpi	2dpi
_Col:_mock:dde2:_mock	0.9578	0.8662
_Col:_mock:dde2/ein2:_mock	0.9506	0.7651
_Col:_mock:dde2/ein2/pad4:_mock	0.9261	2E-06
_Col:_mock:dde2/ein2/pad4/sid2:_mock	0.7249	1E-08
_Col:_mock:dde2/ein2/sid2:_mock	0.9659	0.0003
_Col:_mock:dde2/pad4:_mock	0.5114	2E-05
_Col:_mock:dde2/pad4/sid2:_mock	0.8384	2E-05
_Col:_mock:dde2/sid2:_mock	0.5647	0.0121
_Col:_mock:ein2:_mock	0.9454	0.2539
_Col:_mock:ein2/pad4:_mock	0.6475	0.8684
_Col:_mock:ein2/pad4/sid2:_mock	0.7217	0.0003
_Col:_mock:ein2/sid2:_mock	0.9353	9E-07
_Col:_mock:efr:_mock	0.7842	0.817
_Col:_mock:pad4:_mock	0.9566	0.0039
_Col:_mock:pad4/sid2:_mock	0.913	3E-05
_Col:_mock:sid2:_mock	0.9623	0.0385
_Col:_mock:_Col:elf18	0.8022	9E-22
_Col:_mock:dde2:elf18	0.7577	4E-32
_Col:_mock:dde2/ein2:elf18	0.8621	3E-43
_Col:_mock:dde2/ein2/pad4:elf18	0.9484	0.0013
_Col:_mock:dde2/ein2/pad4/sid2:elf18	0.8714	0.8922
_Col:_mock:dde2/ein2/sid2:elf18	0.9887	6E-05
_Col:_mock:dde2/pad4:elf18	0.5687	0.0071
_Col:_mock:dde2/pad4/sid2:elf18	0.6911	0.0009
_Col:_mock:dde2/sid2:elf18	0.8189	2E-06
_Col:_mock:ein2:elf18	0.7824	0.4046
_Col:_mock:ein2/pad4:elf18	0.7528	4E-47
_Col:_mock:ein2/pad4/sid2:elf18	0.6162	0.177
_Col:_mock:ein2/sid2:elf18	0.9217	0.5884
_Col:_mock:efr:elf18	0.7127	6E-13
_Col:_mock:pad4:elf18	0.5258	0.0199
_Col:_mock:pad4/sid2:elf18	0.6932	0.1438
_Col:_mock:sid2:elf18	0.6734	5E-06
dde2:_mock:dde2/ein2:_mock	0.9928	0.8963
dde2:_mock:dde2/ein2/pad4:_mock	0.9682	5E-06
dde2:_mock:dde2/ein2/pad4/sid2:_mock	0.6856	3E-08
dde2:_mock:dde2/ein2/sid2:_mock	0.9238	0.0006
dde2:_mock:dde2/pad4:_mock	0.478	5E-05
dde2:_mock:dde2/pad4/sid2:_mock	0.88	4E-05
dde2:_mock:dde2/sid2:_mock	0.601	0.0192
dde2:_mock:ein2:_mock	0.9034	0.3308
dde2:_mock:ein2/pad4:_mock	0.61	0.7382
dde2:_mock:ein2/pad4/sid2:_mock	0.7616	0.0005
dde2:_mock:ein2/sid2:_mock	0.9774	2E-06
dde2:_mock:efr:_mock	0.8252	0.9499
dde2:_mock:pad4:_mock	0.9988	0.0065
dde2:_mock:pad4/sid2:_mock	0.955	6E-05
dde2:_mock:sid2:_mock	0.9202	0.0573
dde2:_mock:_Col:elf18	0.8428	2E-22
dde2:_mock:dde2:elf18	0.7976	8E-33
dde2:_mock:dde2/ein2:elf18	0.9033	5E-44
dde2:_mock:dde2/ein2/pad4:elf18	0.99	0.0007
dde2:_mock:dde2/ein2/pad4/sid2:elf18	0.9126	0.7731
dde2:_mock:dde2/ein2/sid2:elf18	0.9697	3E-05
dde2:_mock:dde2/pad4:elf18	0.5339	0.0044
dde2:_mock:dde2/pad4/sid2:elf18	0.73	0.0005
dde2:_mock:dde2/sid2:elf18	0.8596	1E-06
dde2:_mock:ein2:elf18	0.7426	0.4961
dde2:_mock:ein2/pad4:elf18	0.7136	5E-48
dde2:_mock:ein2/pad4/sid2:elf18	0.6534	0.1329
dde2:_mock:ein2/sid2:elf18	0.9632	0.4877

dde2:_mock:efr:elf18	0.7519	2E-13
dde2:_mock:pad4:elf18	0.4924	0.0131
dde2:_mock:pad4/sid2:elf18	0.7321	0.1064
dde2:_mock:sid2:elf18	0.7119	2E-06
dde2/ein2:_mock:dde2/ein2/pad4:_mock	0.9754	9E-06
dde2/ein2:_mock:dde2/ein2/pad4/sid2:_m	0.679	5E-08
dde2/ein2:_mock:dde2/ein2/sid2:_mock	0.9167	0.0009
dde2/ein2:_mock:dde2/pad4:_mock	0.4724	8E-05
dde2/ein2:_mock:dde2/pad4/sid2:_mock	0.8871	7E-05
dde2/ein2:_mock:dde2/sid2:_mock	0.6073	0.0269
dde2/ein2:_mock:ein2:_mock	0.8963	0.3996
dde2/ein2:_mock:ein2/pad4:_mock	0.6037	0.6422
dde2/ein2:_mock:ein2/pad4/sid2:_mock	0.7685	0.0009
dde2/ein2:_mock:ein2/sid2:_mock	0.9846	4E-06
dde2/ein2:_mock:efr:_mock	0.8322	0.9462
dde2/ein2:_mock:pad4:_mock	0.994	0.0095
dde2/ein2:_mock:pad4/sid2:_mock	0.9622	0.0001
dde2/ein2:_mock:sid2:_mock	0.9131	0.0766
dde2/ein2:_mock:_Col:elf18	0.8497	8E-23
dde2/ein2:_mock:dde2:elf18	0.8045	2E-33
dde2/ein2:_mock:dde2/ein2:elf18	0.9103	1E-44
dde2/ein2:_mock:dde2/ein2/pad4:elf18	0.997	0.0005
dde2/ein2:_mock:dde2/ein2/pad4/sid2:elf	0.9197	0.6843
dde2/ein2:_mock:dde2/ein2/sid2:elf18	0.9627	2E-05
dde2/ein2:_mock:dde2/pad4:elf18	0.5281	0.0031
dde2/ein2:_mock:dde2/pad4/sid2:elf18	0.7366	0.0003
dde2/ein2:_mock:dde2/sid2:elf18	0.8666	6E-07
dde2/ein2:_mock:ein2:elf18	0.736	0.5738
dde2/ein2:_mock:ein2/pad4:elf18	0.707	1E-48
dde2/ein2:_mock:ein2/pad4/sid2:elf18	0.6598	0.1051
dde2/ein2:_mock:ein2/sid2:elf18	0.9702	0.4167
dde2/ein2:_mock:efr:elf18	0.7586	9E-14
dde2/ein2:_mock:pad4:elf18	0.4868	0.0093
dde2/ein2:_mock:pad4/sid2:elf18	0.7388	0.0832
dde2/ein2:_mock:sid2:elf18	0.7185	1E-06
dde2/ein2/pad4:_mock:dde2/ein2/pad4/si	0.6566	0.3053
dde2/ein2/pad4:_mock:dde2/ein2/sid2:_m	0.8922	0.2611
dde2/ein2/pad4:_mock:dde2/pad4:_mock	0.4536	0.6202
dde2/ein2/pad4:_mock:dde2/pad4/sid2:_	0.9115	0.6374
dde2/ein2/pad4:_mock:dde2/sid2:_mock	0.629	0.0254
dde2/ein2/pad4:_mock:ein2:_mock	0.8719	0.0003
dde2/ein2/pad4:_mock:ein2/pad4:_mock	0.5824	1E-06
dde2/ein2/pad4:_mock:ein2/pad4/sid2:_m	0.7922	0.265
dde2/ein2/pad4:_mock:ein2/sid2:_mock	0.9908	0.8532
dde2/ein2/pad4:_mock:efr:_mock	0.8563	7E-06
dde2/ein2/pad4:_mock:pad4:_mock	0.9694	0.0635
dde2/ein2/pad4:_mock:pad4/sid2:_mock	0.9868	0.5659
dde2/ein2/pad4:_mock:sid2:_mock	0.8886	0.0074
dde2/ein2/pad4:_mock:_Col:elf18	0.8736	2E-41
dde2/ein2/pad4:_mock:dde2:elf18	0.8281	2E-54
dde2/ein2/pad4:_mock:dde2/ein2:elf18	0.9344	1E-67
dde2/ein2/pad4:_mock:dde2/ein2/pad4:e	0.9787	1E-13
dde2/ein2/pad4:_mock:dde2/ein2/pad4/si	0.9438	1E-05
dde2/ein2/pad4:_mock:dde2/ein2/sid2:elf	0.9385	2E-16
dde2/ein2/pad4:_mock:dde2/pad4:elf18	0.5085	4E-12
dde2/ein2/pad4:_mock:dde2/pad4/sid2:el	0.7597	5E-14
dde2/ein2/pad4:_mock:dde2/sid2:elf18	0.8905	6E-19
dde2/ein2/pad4:_mock:ein2:elf18	0.7132	0.0005
dde2/ein2/pad4:_mock:ein2/pad4:elf18	0.6845	3E-72
dde2/ein2/pad4:_mock:ein2/pad4/sid2:elf	0.6819	2E-08
dde2/ein2/pad4:_mock:ein2/sid2:elf18	0.9945	1E-06
dde2/ein2/pad4:_mock:efr:elf18	0.7819	3E-29
dde2/ein2/pad4:_mock:pad4:elf18	0.468	5E-11

dde2/ein2/pad4:_mock:pad4/sid2:elf18	0.7618	1E-08
dde2/ein2/pad4:_mock:sid2:elf18	0.7413	3E-18
dde2/ein2/pad4/sid2:_mock:dde2/ein2/sic	0.7572	0.0318
dde2/ein2/pad4/sid2:_mock:dde2/pad4:_	0.7604	0.1285
dde2/ein2/pad4/sid2:_mock:dde2/pad4/si	0.5784	0.1347
dde2/ein2/pad4/sid2:_mock:dde2/sid2:_n	0.3538	0.0011
dde2/ein2/pad4/sid2:_mock:ein2:_mock	0.7769	4E-06
dde2/ein2/pad4/sid2:_mock:ein2/pad4:_n	0.9161	4E-09
dde2/ein2/pad4/sid2:_mock:ein2/pad4/sic	0.479	0.0325
dde2/ein2/pad4/sid2:_mock:ein2/sid2:_m	0.665	0.4008
dde2/ein2/pad4/sid2:_mock:efr:_mock	0.5316	4E-08
dde2/ein2/pad4/sid2:_mock:pad4:_mock	0.6845	0.004
dde2/ein2/pad4/sid2:_mock:pad4/sid2:_n	0.6447	0.1099
dde2/ein2/pad4/sid2:_mock:sid2:_mock	0.7606	0.0002
dde2/ein2/pad4/sid2:_mock:_Col:elf18	0.5505	2E-46
dde2/ein2/pad4/sid2:_mock:dde2:elf18	0.5124	7E-60
dde2/ein2/pad4/sid2:_mock:dde2/ein2:elf	0.6029	2E-73
dde2/ein2/pad4/sid2:_mock:dde2/ein2/pa	0.6808	8E-17
dde2/ein2/pad4/sid2:_mock:dde2/ein2/pa	0.6112	9E-08
dde2/ein2/pad4/sid2:_mock:dde2/ein2/sic	0.7182	1E-19
dde2/ein2/pad4/sid2:_mock:dde2/pad4:el	0.8232	5E-15
dde2/ein2/pad4/sid2:_mock:dde2/pad4/si	0.457	4E-17
dde2/ein2/pad4/sid2:_mock:dde2/sid2:elf	0.5649	2E-22
dde2/ein2/pad4/sid2:_mock:ein2:elf18	0.9438	1E-05
dde2/ein2/pad4/sid2:_mock:ein2/pad4:elf	0.9747	4E-78
dde2/ein2/pad4/sid2:_mock:ein2/pad4/sic	0.3967	7E-11
dde2/ein2/pad4/sid2:_mock:ein2/sid2:elf1	0.6564	1E-08
dde2/ein2/pad4/sid2:_mock:efr:elf18	0.4748	1E-33
dde2/ein2/pad4/sid2:_mock:pad4:elf18	0.7734	8E-14
dde2/ein2/pad4/sid2:_mock:pad4/sid2:elf	0.4587	3E-11
dde2/ein2/pad4/sid2:_mock:sid2:elf18	0.4426	8E-22
dde2/ein2/sid2:_mock:dde2/pad4:_mock	0.5392	0.5298
dde2/ein2/sid2:_mock:dde2/pad4/sid2:_n	0.8052	0.514
dde2/ein2/sid2:_mock:dde2/sid2:_mock	0.5362	0.2653
dde2/ein2/sid2:_mock:ein2:_mock	0.9795	0.013
dde2/ein2/sid2:_mock:ein2/pad4:_mock	0.6785	0.0002
dde2/ein2/sid2:_mock:ein2/pad4/sid2:_m	0.6899	0.9928
dde2/ein2/sid2:_mock:ein2/sid2:_mock	0.9014	0.1907
dde2/ein2/sid2:_mock:efr:_mock	0.7516	0.0007
dde2/ein2/sid2:_mock:pad4:_mock	0.9226	0.4634
dde2/ein2/sid2:_mock:pad4/sid2:_mock	0.8792	0.5824
dde2/ein2/sid2:_mock:sid2:_mock	0.9964	0.1197
dde2/ein2/sid2:_mock:_Col:elf18	0.7698	2E-36
dde2/ein2/sid2:_mock:dde2:elf18	0.7259	8E-49
dde2/ein2/sid2:_mock:dde2/ein2:elf18	0.8292	1E-61
dde2/ein2/sid2:_mock:dde2/ein2/pad4:elf	0.9149	1E-10
dde2/ein2/sid2:_mock:dde2/ein2/pad4/sic	0.8384	0.0006
dde2/ein2/sid2:_mock:dde2/ein2/sid2:elf1	0.9551	5E-13
dde2/ein2/sid2:_mock:dde2/pad4:elf18	0.5976	3E-09
dde2/ein2/sid2:_mock:dde2/pad4/sid2:elf	0.6604	6E-11
dde2/ein2/sid2:_mock:dde2/sid2:elf18	0.7863	2E-15
dde2/ein2/sid2:_mock:ein2:elf18	0.8149	0.0141
dde2/ein2/sid2:_mock:ein2/pad4:elf18	0.785	5E-66
dde2/ein2/sid2:_mock:ein2/pad4/sid2:elf1	0.5869	4E-06
dde2/ein2/sid2:_mock:ein2/sid2:elf18	0.8883	0.0001
dde2/ein2/sid2:_mock:efr:elf18	0.6816	7E-25
dde2/ein2/sid2:_mock:pad4:elf18	0.5536	2E-08
dde2/ein2/sid2:_mock:pad4/sid2:elf18	0.6624	2E-06
dde2/ein2/sid2:_mock:sid2:elf18	0.643	8E-15
dde2/pad4:_mock:dde2/pad4/sid2:_mock	0.3896	0.9807
dde2/pad4:_mock:dde2/sid2:_mock	0.2181	0.0816
dde2/pad4:_mock:ein2:_mock	0.5564	0.0019
dde2/pad4:_mock:ein2/pad4:_mock	0.8418	1E-05

dde2/pad4:_mock:ein2/pad4/sid2:_mock	0.3113	0.5358
dde2/pad4:_mock:ein2/sid2:_mock	0.4606	0.4961
dde2/pad4:_mock:efr:_mock	0.3523	6E-05
dde2/pad4:_mock:pad4:_mock	0.4771	0.1735
dde2/pad4:_mock:pad4/sid2:_mock	0.4438	0.9374
dde2/pad4:_mock:sid2:_mock	0.5422	0.029
dde2/pad4:_mock:_Col:elf18	0.3697	3E-39
dde2/pad4:_mock:dde2:elf18	0.3395	6E-52
dde2/pad4:_mock:dde2/ein2:elf18	0.412	5E-65
dde2/pad4:_mock:dde2/ein2/pad4:elf18	0.4767	2E-12
dde2/pad4:_mock:dde2/ein2/pad4/sid2:el	0.4188	7E-05
dde2/pad4:_mock:dde2/ein2/sid2:elf18	0.5086	7E-15
dde2/pad4:_mock:dde2/pad4:elf18	0.9386	8E-11
dde2/pad4:_mock:dde2/pad4/sid2:elf18	0.2966	1E-12
dde2/pad4:_mock:dde2/sid2:elf18	0.3812	3E-17
dde2/pad4:_mock:ein2:elf18	0.7108	0.0025
dde2/pad4:_mock:ein2/pad4:elf18	0.7398	2E-69
dde2/pad4:_mock:ein2/pad4/sid2:elf18	0.2512	2E-07
dde2/pad4:_mock:ein2/sid2:elf18	0.4562	1E-05
dde2/pad4:_mock:efr:elf18	0.3103	2E-27
dde2/pad4:_mock:pad4:elf18	0.9901	8E-10
dde2/pad4:_mock:pad4/sid2:elf18	0.298	1E-07
dde2/pad4:_mock:sid2:elf18	0.2856	1E-16
dde2/pad4/sid2:_mock:dde2/sid2:_mock	0.7099	0.0775
dde2/pad4/sid2:_mock:ein2:_mock	0.7853	0.0017
dde2/pad4/sid2:_mock:ein2/pad4:_mock	0.5086	1E-05
dde2/pad4/sid2:_mock:ein2/pad4/sid2:_n	0.8789	0.5199
dde2/pad4/sid2:_mock:ein2/sid2:_mock	0.9023	0.5116
dde2/pad4/sid2:_mock:efr:_mock	0.9443	5E-05
dde2/pad4/sid2:_mock:pad4:_mock	0.8812	0.166
dde2/pad4/sid2:_mock:pad4/sid2:_mock	0.9246	0.9182
dde2/pad4/sid2:_mock:sid2:_mock	0.8017	0.0273
dde2/pad4/sid2:_mock:_Col:elf18	0.9604	3E-39
dde2/pad4/sid2:_mock:dde2:elf18	0.9142	4E-52
dde2/pad4/sid2:_mock:dde2/ein2:elf18	0.9783	4E-65
dde2/pad4/sid2:_mock:dde2/ein2/pad4:el	0.8917	2E-12
dde2/pad4/sid2:_mock:dde2/ein2/pad4/si	0.9689	6E-05
dde2/pad4/sid2:_mock:dde2/ein2/sid2:elf	0.8519	6E-15
dde2/pad4/sid2:_mock:dde2/pad4:elf18	0.4409	7E-11
dde2/pad4/sid2:_mock:dde2/pad4/sid2:el	0.8443	1E-12
dde2/pad4/sid2:_mock:dde2/sid2:elf18	0.9776	2E-17
dde2/pad4/sid2:_mock:ein2:elf18	0.6333	0.0023
dde2/pad4/sid2:_mock:ein2/pad4:elf18	0.606	1E-69
dde2/pad4/sid2:_mock:ein2/pad4/sid2:elf	0.7639	2E-07
dde2/pad4/sid2:_mock:ein2/sid2:elf18	0.9183	1E-05
dde2/pad4/sid2:_mock:efr:elf18	0.8671	2E-27
dde2/pad4/sid2:_mock:pad4:elf18	0.4036	7E-10
dde2/pad4/sid2:_mock:pad4/sid2:elf18	0.8465	1E-07
dde2/pad4/sid2:_mock:sid2:elf18	0.8254	8E-17
dde2/sid2:_mock:ein2:_mock	0.5194	0.1701
dde2/sid2:_mock:ein2/pad4:_mock	0.3018	0.0075
dde2/sid2:_mock:ein2/pad4/sid2:_mock	0.8261	0.2614
dde2/sid2:_mock:ein2/sid2:_mock	0.6208	0.0155
dde2/sid2:_mock:efr:_mock	0.7626	0.0226
dde2/sid2:_mock:pad4:_mock	0.6021	0.7032
dde2/sid2:_mock:pad4/sid2:_mock	0.6408	0.0963
dde2/sid2:_mock:sid2:_mock	0.5333	0.658
dde2/sid2:_mock:_Col:elf18	0.7514	2E-31
dde2/sid2:_mock:dde2:elf18	0.7958	2E-43
dde2/sid2:_mock:dde2/ein2:elf18	0.6938	1E-55
dde2/sid2:_mock:dde2/ein2/pad4:elf18	0.6153	4E-08
dde2/sid2:_mock:dde2/ein2/pad4/sid2:elf	0.6851	0.0158
dde2/sid2:_mock:dde2/ein2/sid2:elf18	0.5802	4E-10

dde2/sid2:_mock:dde2/pad4:elf18	0.2558	7E-07
dde2/sid2:_mock:dde2/pad4/sid2:elf18	0.865	3E-08
dde2/sid2:_mock:dde2/sid2:elf18	0.7351	4E-12
dde2/sid2:_mock:ein2:elf18	0.3991	0.1482
dde2/sid2:_mock:ein2/pad4:elf18	0.3779	5E-60
dde2/sid2:_mock:ein2/pad4/sid2:elf18	0.9473	0.0003
dde2/sid2:_mock:ein2/sid2:elf18	0.6391	0.0049
dde2/sid2:_mock:efr:elf18	0.8422	8E-21
dde2/sid2:_mock:pad4:elf18	0.2298	4E-06
dde2/sid2:_mock:pad4/sid2:elf18	0.8628	0.0002
dde2/sid2:_mock:sid2:elf18	0.884	1E-11
ein2:_mock:ein2/pad4:_mock	0.6974	0.1914
ein2:_mock:ein2/pad4/sid2:_mock	0.6711	0.0127
ein2:_mock:ein2/sid2:_mock	0.881	0.0002
ein2:_mock:efr:_mock	0.7321	0.363
ein2:_mock:pad4:_mock	0.9022	0.0797
ein2:_mock:pad4/sid2:_mock	0.8589	0.0024
ein2:_mock:sid2:_mock	0.9831	0.3525
ein2:_mock:_Col:elf18	0.7505	5E-26
ein2:_mock:dde2:elf18	0.7069	5E-37
ein2:_mock:dde2/ein2:elf18	0.8095	1E-48
ein2:_mock:dde2/ein2/pad4:elf18	0.8949	2E-05
ein2:_mock:dde2/ein2/pad4/sid2:elf18	0.8186	0.2419
ein2:_mock:dde2/ein2/sid2:elf18	0.9349	5E-07
ein2:_mock:dde2/pad4:elf18	0.6153	0.0002
ein2:_mock:dde2/pad4/sid2:elf18	0.6421	1E-05
ein2:_mock:dde2/sid2:elf18	0.7669	1E-08
ein2:_mock:ein2:elf18	0.8346	0.8402
ein2:_mock:ein2/pad4:elf18	0.8045	7E-53
ein2:_mock:ein2/pad4/sid2:elf18	0.5696	0.0172
ein2:_mock:ein2/sid2:elf18	0.8683	0.1151
ein2:_mock:efr:elf18	0.6631	3E-16
ein2:_mock:pad4:elf18	0.5707	0.0008
ein2:_mock:pad4/sid2:elf18	0.6441	0.0126
ein2:_mock:sid2:elf18	0.6249	2E-08
ein2/pad4:_mock:ein2/pad4/sid2:_mock	0.4161	0.0002
ein2/pad4:_mock:ein2/sid2:_mock	0.5903	4E-07
ein2/pad4:_mock:efr:_mock	0.4649	0.6913
ein2/pad4:_mock:pad4:_mock	0.6089	0.0023
ein2/pad4:_mock:pad4/sid2:_mock	0.5711	2E-05
ein2/pad4:_mock:sid2:_mock	0.6818	0.0255
ein2/pad4:_mock:_Col:elf18	0.4835	4E-21
ein2/pad4:_mock:dde2:elf18	0.448	2E-31
ein2/pad4:_mock:dde2/ein2:elf18	0.5326	2E-42
ein2/pad4:_mock:dde2/ein2/pad4:elf18	0.6065	0.0021
ein2/pad4:_mock:dde2/ein2/pad4/sid2:elf	0.5404	0.9882
ein2/pad4:_mock:dde2/ein2/sid2:elf18	0.6422	0.0001
ein2/pad4:_mock:dde2/pad4:elf18	0.9048	0.011
ein2/pad4:_mock:dde2/pad4/sid2:elf18	0.3968	0.0016
ein2/pad4:_mock:dde2/sid2:elf18	0.497	5E-06
ein2/pad4:_mock:ein2:elf18	0.8617	0.3253
ein2/pad4:_mock:ein2/pad4:elf18	0.8922	2E-46
ein2/pad4:_mock:ein2/pad4/sid2:elf18	0.3415	0.2301
ein2/pad4:_mock:ein2/sid2:elf18	0.5832	0.6959
ein2/pad4:_mock:efr:elf18	0.4131	2E-12
ein2/pad4:_mock:pad4:elf18	0.8539	0.0293
ein2/pad4:_mock:pad4/sid2:elf18	0.3983	0.1896
ein2/pad4:_mock:sid2:elf18	0.3835	1E-05
ein2/pad4/sid2:_mock:ein2/sid2:_mock	0.7833	0.1938
ein2/pad4/sid2:_mock:efr:_mock	0.9343	0.0007
ein2/pad4/sid2:_mock:pad4:_mock	0.7628	0.4579
ein2/pad4/sid2:_mock:pad4/sid2:_mock	0.8049	0.5887
ein2/pad4/sid2:_mock:sid2:_mock	0.6866	0.1176

ein2/pad4/sid2:_mock:_Col:elf18	0.92	2E-36
ein2/pad4/sid2:_mock:dde2:elf18	0.9662	7E-49
ein2/pad4/sid2:_mock:dde2/ein2:elf18	0.8593	1E-61
ein2/pad4/sid2:_mock:dde2/ein2/pad4:elf	0.7747	1E-10
ein2/pad4/sid2:_mock:dde2/ein2/pad4/sid	0.85	0.0006
ein2/pad4/sid2:_mock:dde2/ein2/sid2:elf1	0.7363	4E-13
ein2/pad4/sid2:_mock:dde2/pad4:elf18	0.3573	3E-09
ein2/pad4/sid2:_mock:dde2/pad4/sid2:elf	0.963	6E-11
ein2/pad4/sid2:_mock:dde2/sid2:elf18	0.9029	2E-15
ein2/pad4/sid2:_mock:ein2:elf18	0.5306	0.0138
ein2/pad4/sid2:_mock:ein2/pad4:elf18	0.5056	4E-66
ein2/pad4/sid2:_mock:ein2/pad4/sid2:elf1	0.8805	4E-06
ein2/pad4/sid2:_mock:ein2/sid2:elf18	0.8006	0.0001
ein2/pad4/sid2:_mock:efr:elf18	0.9862	6E-25
ein2/pad4/sid2:_mock:pad4:elf18	0.3247	2E-08
ein2/pad4/sid2:_mock:pad4/sid2:elf18	0.9653	2E-06
ein2/pad4/sid2:_mock:sid2:elf18	0.9438	8E-15
ein2/sid2:_mock:efr:_mock	0.8473	3E-06
ein2/sid2:_mock:pad4:_mock	0.9786	0.0413
ein2/sid2:_mock:pad4/sid2:_mock	0.9776	0.4478
ein2/sid2:_mock:sid2:_mock	0.8978	0.0042
ein2/sid2:_mock:_Col:elf18	0.8646	2E-42
ein2/sid2:_mock:dde2:elf18	0.8192	2E-55
ein2/sid2:_mock:dde2/ein2:elf18	0.9254	1E-68
ein2/sid2:_mock:dde2/ein2/pad4:elf18	0.9878	3E-14
ein2/sid2:_mock:dde2/ein2/pad4/sid2:elf1	0.9348	4E-06
ein2/sid2:_mock:dde2/ein2/sid2:elf18	0.9475	6E-17
ein2/sid2:_mock:dde2/pad4:elf18	0.5158	1E-12
ein2/sid2:_mock:dde2/pad4/sid2:elf18	0.751	2E-14
ein2/sid2:_mock:dde2/sid2:elf18	0.8815	2E-19
ein2/sid2:_mock:ein2:elf18	0.7217	0.0003
ein2/sid2:_mock:ein2/pad4:elf18	0.6929	2E-73
ein2/sid2:_mock:ein2/pad4/sid2:elf18	0.6736	7E-09
ein2/sid2:_mock:ein2/sid2:elf18	0.9854	6E-07
ein2/sid2:_mock:efr:elf18	0.7731	5E-30
ein2/sid2:_mock:pad4:elf18	0.475	2E-11
ein2/sid2:_mock:pad4/sid2:elf18	0.7531	4E-09
ein2/sid2:_mock:sid2:elf18	0.7327	6E-19
efr:_mock:pad4:_mock	0.8263	0.0078
efr:_mock:pad4/sid2:_mock	0.8693	8E-05
efr:_mock:sid2:_mock	0.7482	0.066
efr:_mock:_Col:elf18	0.9846	1E-22
efr:_mock:dde2:elf18	0.969	4E-33
efr:_mock:dde2/ein2:elf18	0.9234	3E-44
efr:_mock:dde2/ein2/pad4:elf18	0.8375	0.0006
efr:_mock:dde2/ein2/pad4/sid2:elf18	0.9141	0.7298
efr:_mock:dde2/ein2/sid2:elf18	0.7983	2E-05
efr:_mock:dde2/pad4:elf18	0.4012	0.0037
efr:_mock:dde2/pad4/sid2:elf18	0.8985	0.0004
efr:_mock:dde2/sid2:elf18	0.9675	8E-07
efr:_mock:ein2:elf18	0.5851	0.5329
efr:_mock:ein2/pad4:elf18	0.5588	3E-48
efr:_mock:ein2/pad4/sid2:elf18	0.8169	0.1188
efr:_mock:ein2/sid2:elf18	0.8639	0.4527
efr:_mock:efr:elf18	0.9215	1E-13
efr:_mock:pad4:elf18	0.3661	0.0111
efr:_mock:pad4/sid2:elf18	0.9007	0.0947
efr:_mock:sid2:elf18	0.8794	2E-06
pad4:_mock:pad4/sid2:_mock	0.9562	0.1996
pad4:_mock:sid2:_mock	0.919	0.4102
pad4:_mock:_Col:elf18	0.8439	4E-33
pad4:_mock:dde2:elf18	0.7988	3E-45
pad4:_mock:dde2/ein2:elf18	0.9045	1E-57

pad4:_mock:dde2/ein2/pad4:elf18	0.9912	6E-09
pad4:_mock:dde2/ein2/pad4/sid2:elf18	0.9138	0.0058
pad4:_mock:dde2/ein2/sid2:elf18	0.9686	4E-11
pad4:_mock:dde2/pad4:elf18	0.533	1E-07
pad4:_mock:dde2/pad4/sid2:elf18	0.7311	4E-09
pad4:_mock:dde2/sid2:elf18	0.8608	3E-13
pad4:_mock:ein2:elf18	0.7415	0.0733
pad4:_mock:ein2/pad4:elf18	0.7125	4E-62
pad4:_mock:ein2/pad4/sid2:elf18	0.6544	7E-05
pad4:_mock:ein2/sid2:elf18	0.9643	0.0016
pad4:_mock:efr:elf18	0.753	3E-22
pad4:_mock:pad4:elf18	0.4914	8E-07
pad4:_mock:pad4/sid2:elf18	0.7332	5E-05
pad4:_mock:sid2:elf18	0.713	1E-12
pad4/sid2:_mock:sid2:_mock	0.8756	0.0353
pad4/sid2:_mock:_Col:elf18	0.8864	8E-39
pad4/sid2:_mock:dde2:elf18	0.8408	1E-51
pad4/sid2:_mock:dde2/ein2:elf18	0.9474	1E-64
pad4/sid2:_mock:dde2/ein2/pad4:elf18	0.9657	4E-12
pad4/sid2:_mock:dde2/ein2/pad4/sid2:elf	0.9568	9E-05
pad4/sid2:_mock:dde2/ein2/sid2:elf18	0.9255	1E-14
pad4/sid2:_mock:dde2/pad4:elf18	0.4981	1E-10
pad4/sid2:_mock:dde2/pad4/sid2:elf18	0.7721	2E-12
pad4/sid2:_mock:dde2/sid2:elf18	0.9034	5E-17
pad4/sid2:_mock:ein2:elf18	0.7011	0.0032
pad4/sid2:_mock:ein2/pad4:elf18	0.6726	4E-69
pad4/sid2:_mock:ein2/pad4/sid2:elf18	0.6939	3E-07
pad4/sid2:_mock:ein2/sid2:elf18	0.9925	2E-05
pad4/sid2:_mock:efr:elf18	0.7944	5E-27
pad4/sid2:_mock:pad4:elf18	0.4581	1E-09
pad4/sid2:_mock:pad4/sid2:elf18	0.7742	2E-07
pad4/sid2:_mock:sid2:elf18	0.7537	2E-16
sid2:_mock:_Col:elf18	0.7664	1E-29
sid2:_mock:dde2:elf18	0.7225	3E-41
sid2:_mock:dde2/ein2:elf18	0.8257	2E-53
sid2:_mock:dde2/ein2/pad4:elf18	0.9114	4E-07
sid2:_mock:dde2/ein2/pad4/sid2:elf18	0.8349	0.0442
sid2:_mock:dde2/ein2/sid2:elf18	0.9516	4E-09
sid2:_mock:dde2/pad4:elf18	0.6007	5E-06
sid2:_mock:dde2/pad4/sid2:elf18	0.6572	2E-07
sid2:_mock:dde2/sid2:elf18	0.7829	5E-11
sid2:_mock:ein2:elf18	0.8183	0.296
sid2:_mock:ein2/pad4:elf18	0.7884	1E-57
sid2:_mock:ein2/pad4/sid2:elf18	0.5839	0.0013
sid2:_mock:ein2/sid2:elf18	0.8848	0.0157
sid2:_mock:efr:elf18	0.6783	3E-19
sid2:_mock:pad4:elf18	0.5566	3E-05
sid2:_mock:pad4/sid2:elf18	0.6592	0.0009
sid2:_mock:sid2:elf18	0.6398	2E-10
_Col:elf18:dde2:elf18	0.953	0.0083
_Col:elf18:dde2/ein2:elf18	0.9378	4E-07
_Col:elf18:dde2/ein2/pad4:elf18	0.8504	8E-13
_Col:elf18:dde2/ein2/pad4/sid2:elf18	0.9283	3E-25
_Col:elf18:dde2/ein2/sid2:elf18	0.8104	3E-10
_Col:elf18:dde2/pad4:elf18	0.4051	1E-14
_Col:elf18:dde2/pad4/sid2:elf18	0.8815	2E-12
_Col:elf18:dde2/sid2:elf18	0.9826	3E-08
_Col:elf18:ein2:elf18	0.5929	7E-30
_Col:elf18:ein2/pad4:elf18	0.5661	4E-09
_Col:elf18:ein2/pad4/sid2:elf18	0.7991	7E-20
_Col:elf18:ein2/sid2:elf18	0.8772	2E-23
_Col:elf18:efr:elf18	0.9048	0.0056
_Col:elf18:pad4:elf18	0.3692	5E-16

_Col:elf18:pad4/sid2:elf18	0.8838	2E-19
_Col:elf18:sid2:elf18	0.8622	1E-08
dde2:elf18:dde2/ein2:elf18	0.8911	0.0137
dde2:elf18:dde2/ein2/pad4:elf18	0.8045	4E-22
dde2:elf18:dde2/ein2/pad4/sid2:elf18	0.8816	2E-37
dde2:elf18:dde2/ein2/sid2:elf18	0.7651	1E-18
dde2:elf18:dde2/pad4:elf18	0.3727	2E-24
dde2:elf18:dde2/pad4/sid2:elf18	0.9282	1E-21
dde2:elf18:dde2/sid2:elf18	0.9356	6E-16
dde2:elf18:ein2:elf18	0.5528	8E-43
dde2:elf18:ein2/pad4:elf18	0.5269	0.001
dde2:elf18:ein2/pad4/sid2:elf18	0.8449	5E-31
dde2:elf18:ein2/sid2:elf18	0.831	3E-35
dde2:elf18:efr:elf18	0.9517	7E-08
dde2:elf18:pad4:elf18	0.3386	3E-26
dde2:elf18:pad4/sid2:elf18	0.9305	2E-30
dde2:elf18:sid2:elf18	0.9087	1E-16
dde2/ein2:elf18:dde2/ein2/pad4:elf18	0.9119	8E-33
dde2/ein2:elf18:dde2/ein2/pad4/sid2:elf1ε	0.9904	2E-50
dde2/ein2:elf18:dde2/ein2/sid2:elf18	0.8714	9E-29
dde2/ein2:elf18:dde2/pad4:elf18	0.4505	1E-35
dde2/ein2:elf18:dde2/pad4/sid2:elf18	0.8204	2E-32
dde2/ein2:elf18:dde2/sid2:elf18	0.9552	2E-25
dde2/ein2:elf18:ein2:elf18	0.6479	2E-56
dde2/ein2:elf18:ein2/pad4:elf18	0.62	0.4095
dde2/ein2:elf18:ein2/pad4/sid2:elf18	0.7395	4E-43
dde2/ein2:elf18:ein2/sid2:elf18	0.939	7E-48
dde2/ein2:elf18:efr:elf18	0.8434	8E-15
dde2/ein2:elf18:pad4:elf18	0.4121	1E-37
dde2/ein2:elf18:pad4/sid2:elf18	0.8226	2E-42
dde2/ein2:elf18:sid2:elf18	0.8014	4E-26
dde2/ein2/pad4:elf18:dde2/ein2/pad4/sid2	0.9214	0.0007
dde2/ein2/pad4:elf18:dde2/ein2/sid2:elf1ε	0.9591	0.3753
dde2/ein2/pad4:elf18:dde2/pad4:elf18	0.5195	0.5541
dde2/ein2/pad4:elf18:dde2/pad4/sid2:elf1	0.7356	0.9244
dde2/ein2/pad4:elf18:dde2/sid2:elf18	0.8675	0.0952
dde2/ein2/pad4:elf18:ein2:elf18	0.7293	8E-06
dde2/ein2/pad4:elf18:ein2/pad4:elf18	0.7	9E-37
dde2/ein2/pad4:elf18:ein2/pad4/sid2:elf1ε	0.6577	0.038
dde2/ein2/pad4:elf18:ein2/sid2:elf18	0.9728	0.003
dde2/ein2/pad4:elf18:efr:elf18	0.758	9E-06
dde2/ein2/pad4:elf18:pad4:elf18	0.4779	0.3199
dde2/ein2/pad4:elf18:pad4/sid2:elf18	0.7378	0.051
dde2/ein2/pad4:elf18:sid2:elf18	0.7172	0.1368
dde2/ein2/pad4/sid2:elf18:dde2/ein2/sid2	0.8809	2E-05
dde2/ein2/pad4/sid2:elf18:dde2/pad4:elf1	0.4577	0.0048
dde2/ein2/pad4/sid2:elf18:dde2/pad4/sid2	0.8111	0.0005
dde2/ein2/pad4/sid2:elf18:dde2/sid2:elf1ε	0.9456	4E-07
dde2/ein2/pad4/sid2:elf18:ein2:elf18	0.6566	0.2855
dde2/ein2/pad4/sid2:elf18:ein2/pad4:elf1ε	0.6284	5E-55
dde2/ein2/pad4/sid2:elf18:ein2/pad4/sid2	0.7304	0.1805
dde2/ein2/pad4/sid2:elf18:ein2/sid2:elf18	0.9486	0.6547
dde2/ein2/pad4/sid2:elf18:efr:elf18	0.834	8E-15
dde2/ein2/pad4/sid2:elf18:pad4:elf18	0.419	0.0156
dde2/ein2/pad4/sid2:elf18:pad4/sid2:elf1ε	0.8133	0.1437
dde2/ein2/pad4/sid2:elf18:sid2:elf18	0.7921	1E-06
dde2/ein2/sid2:elf18:dde2/pad4:elf18	0.5532	0.1395
dde2/ein2/sid2:elf18:dde2/pad4/sid2:elf1ε	0.6974	0.4285
dde2/ein2/sid2:elf18:dde2/sid2:elf18	0.8274	0.4338
dde2/ein2/sid2:elf18:ein2:elf18	0.7681	1E-07
dde2/ein2/sid2:elf18:ein2/pad4:elf18	0.7383	2E-32
dde2/ein2/sid2:elf18:ein2/pad4/sid2:elf18	0.6211	0.0031
dde2/ein2/sid2:elf18:ein2/sid2:elf18	0.9319	0.0001

dde2/ein2/sid2:elf18:efr:elf18	0.7193	0.0004
dde2/ein2/sid2:elf18:pad4:elf18	0.5103	0.0601
dde2/ein2/sid2:elf18:pad4/sid2:elf18	0.6995	0.0046
dde2/ein2/sid2:elf18:sid2:elf18	0.6793	0.5473
dde2/pad4:elf18:dde2/pad4/sid2:elf18	0.3264	0.4924
dde2/pad4:elf18:dde2/sid2:elf18	0.4175	0.0239
dde2/pad4:elf18:ein2:elf18	0.7655	0.0001
dde2/pad4:elf18:ein2/pad4:elf18	0.7957	1E-39
dde2/pad4:elf18:ein2/pad4/sid2:elf18	0.2772	0.1377
dde2/pad4:elf18:ein2/sid2:elf18	0.4976	0.0176
dde2/pad4:elf18:efr:elf18	0.3411	5E-07
dde2/pad4:elf18:pad4:elf18	0.9477	0.6869
dde2/pad4:elf18:pad4/sid2:elf18	0.3278	0.1734
dde2/pad4:elf18:sid2:elf18	0.3145	0.0377
dde2/pad4/sid2:elf18:dde2/sid2:elf18	0.8643	0.1156
dde2/pad4/sid2:elf18:ein2:elf18	0.4943	5E-06
dde2/pad4/sid2:elf18:ein2/pad4:elf18	0.4698	3E-36
dde2/pad4/sid2:elf18:ein2/pad4/sid2:elf1[illegible]	0.916	0.0301
dde2/pad4/sid2:elf18:ein2/sid2:elf18	0.7615	0.0022
dde2/pad4/sid2:elf18:efr:elf18	0.9765	1E-05
dde2/pad4/sid2:elf18:pad4:elf18	0.2952	0.276
dde2/pad4/sid2:elf18:pad4/sid2:elf18	0.9977	0.0407
dde2/pad4/sid2:elf18:sid2:elf18	0.9804	0.1636
dde2/sid2:elf18:ein2:elf18	0.6081	1E-09
dde2/sid2:elf18:ein2/pad4:elf18	0.5809	6E-29
dde2/sid2:elf18:ein2/pad4/sid2:elf18	0.7823	0.0002
dde2/sid2:elf18:ein2/sid2:elf18	0.8945	4E-06
dde2/sid2:elf18:efr:elf18	0.8876	0.0052
dde2/sid2:elf18:pad4:elf18	0.3809	0.0078
dde2/sid2:elf18:pad4/sid2:elf18	0.8666	0.0003
dde2/sid2:elf18:sid2:elf18	0.8451	0.8564
ein2:elf18:ein2/pad4:elf18	0.9687	3E-61
ein2:elf18:ein2/pad4/sid2:elf18	0.4302	0.0162
ein2:elf18:ein2/sid2:elf18	0.7038	0.1298
ein2:elf18:efr:elf18	0.5131	2E-18
ein2:elf18:pad4:elf18	0.716	0.0005
ein2:elf18:pad4/sid2:elf18	0.4961	0.0115
ein2:elf18:sid2:elf18	0.4789	3E-09
ein2/pad4:elf18:ein2/pad4/sid2:elf18	0.4076	2E-47
ein2/pad4:elf18:ein2/sid2:elf18	0.6749	2E-52
ein2/pad4:elf18:efr:elf18	0.4881	1E-17
ein2/pad4:elf18:pad4:elf18	0.7456	8E-42
ein2/pad4:elf18:pad4/sid2:elf18	0.4716	7E-47
ein2/pad4:elf18:sid2:elf18	0.4549	9E-30
ein2/pad4/sid2:elf18:ein2/sid2:elf18	0.6825	0.3722
ein2/pad4/sid2:elf18:efr:elf18	0.8926	9E-11
ein2/pad4/sid2:elf18:pad4:elf18	0.2493	0.2794
ein2/pad4/sid2:elf18:pad4/sid2:elf18	0.9137	0.902
ein2/pad4/sid2:elf18:sid2:elf18	0.9355	0.0004
ein2/sid2:elf18:efr:elf18	0.7841	2E-13
ein2/sid2:elf18:pad4:elf18	0.457	0.0485
ein2/sid2:elf18:pad4/sid2:elf18	0.7637	0.3099
ein2/sid2:elf18:sid2:elf18	0.7429	9E-06
efr:elf18:pad4:elf18	0.309	6E-08
efr:elf18:pad4/sid2:elf18	0.9788	2E-10
efr:elf18:sid2:elf18	0.9569	0.003
pad4:elf18:pad4/sid2:elf18	0.2965	0.3378
pad4:elf18:sid2:elf18	0.2841	0.0132
pad4/sid2:elf18:sid2:elf18	0.9781	0.0006
