# Supplementary material for: Network Properties of Robust Immunity in Plants
Source: PLoS Genet. 2009 Dec 11;5(12):e1000772. doi: 10.1371/journal.pgen.1000772 (PMC2782137; doi:10.1371/journal.pgen.1000772)
Supplement: Table S9 — P-values for all comparisons in Figure S7B. (0.01 MB PDF) [file pgen.1000772.s017.pdf]

Table S9

Comparisons	0dpi	2dpi
_Col:dde2	0.99661	0.04695
_Col:dde2/ein2	0.92118	0.00014
_Col:dde2/ein2/pad4	0.84229	0.07822
_Col:dde2/ein2/pad4/sid2	0.85308	0.00057
_Col:dde2/ein2/sid2	0.88914	0.05379
_Col:dde2/pad4	0.90091	0.01149
_Col:dde2/pad4/sid2	0.96906	0.0427
_Col:dde2/sid2	0.67256	0.0306
_Col:efr	0.74166	1.50E-13
_Col:ein2	0.93423	4.80E-05
_Col:ein2/pad4	0.94267	6.24E-05
_Col:ein2/pad4/sid2	0.86764	0.0002
_Col:ein2/sid2	0.91313	0.07194
_Col:pad4	0.50055	0.00017
_Col:pad4/sid2	0.97919	0.0004
_Col:sid2	0.87591	0.00928
dde2:dde2/ein2	0.91781	0.06627
dde2:dde2/ein2/pad4	0.83896	0.00019
dde2:dde2/ein2/pad4/sid2	0.85641	6.55E-08
dde2:dde2/ein2/sid2	0.88578	9.45E-05
dde2:dde2/pad4	0.89754	6.87E-06
dde2:dde2/pad4/sid2	0.96566	6.29E-05
dde2:dde2/sid2	0.66946	3.53E-05
dde2:efr	0.73845	1.74E-20
dde2:ein2	0.93085	0.03656
dde2:ein2/pad4	0.93928	2.58E-09
dde2:ein2/pad4/sid2	0.86429	1.39E-08
dde2:ein2/sid2	0.90975	0.00016
dde2:pad4	0.49785	1.05E-08
dde2:pad4/sid2	0.98259	3.85E-08
dde2:sid2	0.87926	4.86E-06
dde2/ein2:dde2/ein2/pad4	0.92032	2.90E-08
dde2/ein2:dde2/ein2/pad4/sid2	0.77631	6.56E-13
dde2/ein2:dde2/ein2/sid2	0.96773	1.12E-08
dde2/ein2:dde2/pad4	0.9796	3.04E-10
dde2/ein2:dde2/pad4/sid2	0.95204	6.35E-09
dde2/ein2:dde2/sid2	0.74615	2.85E-09
dde2/ein2:efr	0.81753	4.67E-28
dde2/ein2:ein2	0.9869	0.79889
dde2/ein2:ein2/pad4	0.97844	1.06E-14
dde2/ein2:ein2/pad4/sid2	0.94601	8.95E-14
dde2/ein2:ein2/sid2	0.9919	2.34E-08
dde2/ein2:pad4	0.56545	6.30E-14
dde2/ein2:pad4/sid2	0.90051	3.31E-13
dde2/ein2:sid2	0.79865	1.91E-10
dde2/ein2/pad4:dde2/ein2/pad4/sid2	0.70088	0.0911
dde2/ein2/pad4:dde2/ein2/sid2	0.95249	0.86671
dde2/ein2/pad4:dde2/pad4	0.94065	0.44202
dde2/ein2/pad4:dde2/pad4/sid2	0.87274	0.79005
dde2/ein2/pad4:dde2/sid2	0.82299	0.68752
dde2/ein2/pad4:efr	0.89601	1.35E-08
dde2/ein2/pad4:ein2	0.9073	6.79E-09
dde2/ein2/pad4:ein2/pad4	0.8989	0.02419
dde2/ein2/pad4:ein2/pad4/sid2	0.97423	0.0492
dde2/ein2/pad4:ein2/sid2	0.92839	0.9693
dde2/ein2/pad4:pad4	0.63494	0.04393
dde2/ein2/pad4:pad4/sid2	0.82195	0.0741
dde2/ein2/pad4:sid2	0.72252	0.39917
dde2/ein2/pad4/sid2:dde2/ein2/sid2	0.7455	0.12798
dde2/ein2/pad4/sid2:dde2/pad4	0.75679	0.35667
dde2/ein2/pad4/sid2:dde2/pad4/sid2	0.82277	0.15449
dde2/ein2/pad4/sid2:dde2/sid2	0.54337	0.19772
dde2/ein2/pad4/sid2:efr	0.6067	5.87E-05
dde2/ein2/pad4/sid2:ein2	0.78892	1.06E-13
dde2/ein2/pad4/sid2:ein2/pad4	0.7971	0.57137

dde2/ein2/pad4/sid2:ein2/pad4/sid2	0.72496	0.7811
dde2/ein2/pad4/sid2:ein2/sid2	0.76855	0.09869
dde2/ein2/pad4/sid2:pad4	0.39056	0.74445
dde2/ein2/pad4/sid2:pad4/sid2	0.87358	0.92306
dde2/ein2/pad4/sid2:sid2	0.97683	0.39676
dde2/ein2/sid2:dde2/pad4	0.98813	0.54783
dde2/ein2/sid2:dde2/pad4/sid2	0.91987	0.9216
dde2/ein2/sid2:dde2/sid2	0.77697	0.81468
dde2/ein2/sid2:efr	0.84909	3.48E-08
dde2/ein2/sid2:ein2	0.95465	2.53E-09
dde2/ein2/sid2:ein2/pad4	0.9462	0.0369
dde2/ein2/sid2:ein2/pad4/sid2	0.97825	0.07195
dde2/ein2/sid2:ein2/sid2	0.97583	0.89708
dde2/ein2/sid2:pad4	0.59311	0.06469
dde2/ein2/sid2:pad4/sid2	0.86857	0.10555
dde2/ein2/sid2:sid2	0.76758	0.49946
dde2/pad4:dde2/pad4/sid2	0.93169	0.61523
dde2/pad4:dde2/sid2	0.76559	0.71388
dde2/pad4:efr	0.83745	8.38E-07
dde2/pad4:ein2	0.9665	6.00E-11
dde2/pad4:ein2/pad4	0.95805	0.13696
dde2/pad4:ein2/pad4/sid2	0.96638	0.23038
dde2/pad4:ein2/sid2	0.9877	0.46519
dde2/pad4:pad4	0.58287	0.21225
dde2/pad4:pad4/sid2	0.8803	0.30857
dde2/pad4:sid2	0.77897	0.94074
dde2/pad4/sid2:dde2/sid2	0.70109	0.89184
dde2/pad4/sid2:efr	0.77115	5.99E-08
dde2/pad4/sid2:ein2	0.96512	1.40E-09
dde2/pad4/sid2:ein2/pad4	0.97357	0.04673
dde2/pad4/sid2:ein2/pad4/sid2	0.89825	0.0889
dde2/pad4/sid2:ein2/sid2	0.94395	0.81983
dde2/pad4/sid2:pad4	0.52552	0.08025
dde2/pad4/sid2:pad4/sid2	0.94828	0.12844
dde2/pad4/sid2:sid2	0.84543	0.56398
dde2/sid2:efr	0.9259	1.25E-07
dde2/sid2:ein2	0.73376	6.10E-10
dde2/sid2:ein2/pad4	0.72579	0.06387
dde2/sid2:ein2/pad4/sid2	0.79794	0.11746
dde2/sid2:ein2/sid2	0.75385	0.71605
dde2/sid2:pad4	0.8017	0.10663
dde2/sid2:pad4/sid2	0.65365	0.1662
dde2/sid2:sid2	0.56279	0.65922
efr:ein2	0.8048	3.45E-29
efr:ein2/pad4	0.7966	0.00055
efr:ein2/pad4/sid2	0.87051	0.00018
efr:ein2/sid2	0.82542	1.68E-08
efr:pad4	0.73075	0.00022
efr:pad4/sid2	0.72205	8.77E-05
efr:sid2	0.62714	1.21E-06
ein2:ein2/pad4	0.99154	1.52E-15
ein2:ein2/pad4/sid2	0.93295	1.36E-14
ein2:ein2/sid2	0.9788	5.42E-09
ein2:pad4	0.55441	9.50E-15
ein2:pad4/sid2	0.91352	5.23E-14
ein2:sid2	0.81136	3.69E-11
ein2/pad4:ein2/pad4/sid2	0.92452	0.7732
ein2/pad4:ein2/sid2	0.97034	0.02671
ein2/pad4:pad4	0.54733	0.81025
ein2/pad4:pad4/sid2	0.92193	0.63873
ein2/pad4:sid2	0.81959	0.15767
ein2/pad4/sid2:ein2/sid2	0.95409	0.0538
ein2/pad4/sid2:pad4	0.6121	0.96165
ein2/pad4/sid2:pad4/sid2	0.84717	0.85612
ein2/pad4/sid2:sid2	0.74685	0.26054
ein2/sid2:pad4	0.57233	0.04811
ein2/sid2:pad4/sid2	0.89247	0.08053

ein2/sid2:sid2	0.79082	0.42103
pad4:pad4/sid2	0.48413	0.81857
pad4:sid2	0.40676	0.24074
pad4/sid2:sid2	0.8965	0.3452
