# Supplementary material for: Network Properties of Robust Immunity in Plants
Source: PLoS Genet. 2009 Dec 11;5(12):e1000772. doi: 10.1371/journal.pgen.1000772 (PMC2782137; doi:10.1371/journal.pgen.1000772)
Supplement: Table S10 — P-values for all comparisons in Figure S8. (0.02 MB PDF) [file pgen.1000772.s018.pdf]

Table S10

Comparisons	p-value
Col:0dpi:dde2:0dpi	0.98557518
Col:0dpi:dde2/ein2:0dpi	0.91176905
Col:0dpi:dde2/ein2/pad4:0dpi	0.8853687
Col:0dpi:quad:0dpi	0.8408841
Col:0dpi:dde2/ein2/sid2:0dpi	0.90339206
Col:0dpi:dde2/pad4:0dpi	0.91356139
Col:0dpi:dde2/pad4/sid2:0dpi	0.61480176
Col:0dpi:dde2/sid2:0dpi	0.99307246
Col:0dpi:ein2:0dpi	0.45334749
Col:0dpi:ein2/pad4:0dpi	0.86190028
Col:0dpi:ein2/pad4/sid2:0dpi	0.9445398
Col:0dpi:ein2/sid2:0dpi	0.58380333
Col:0dpi:pad3:0dpi	0.78920164
Col:0dpi:pad4:0dpi	0.73848246
Col:0dpi:pad4/sid2:0dpi	0.36019085
Col:0dpi:pmr4:0dpi	0.89202054
Col:0dpi:sid2:0dpi	0.64372895
Col:0dpi:Col:3dpi	1.18E-07
Col:0dpi:dde2:3dpi	5.78E-21
Col:0dpi:dde2/ein2:3dpi	1.4232E-33
Col:0dpi:dde2/ein2/pad4:3dpi	1.09E-18
Col:0dpi:quad:3dpi	1.2438E-36
Col:0dpi:dde2/ein2/sid2:3dpi	2.90E-16
Col:0dpi:dde2/pad4:3dpi	1.32E-19
Col:0dpi:dde2/pad4/sid2:3dpi	1.94E-18
Col:0dpi:dde2/sid2:3dpi	1.3992E-18
Col:0dpi:ein2:3dpi	1.4406E-10
Col:0dpi:ein2/pad4:3dpi	4.0343E-09
Col:0dpi:ein2/pad4/sid2:3dpi	5.79E-05
Col:0dpi:ein2/sid2:3dpi	3.9739E-08
Col:0dpi:pad3:3dpi	1.6783E-27
Col:0dpi:pad4:3dpi	2.1879E-06
Col:0dpi:pad4/sid2:3dpi	5.9618E-11
Col:0dpi:pmr4:3dpi	8.4543E-06
Col:0dpi:sid2:3dpi	8.7695E-07
dde2:0dpi:dde2/ein2:0dpi	0.89920964
dde2:0dpi:dde2/ein2/pad4:0dpi	0.89509325
dde2:0dpi:quad:0dpi	0.82677694
dde2:0dpi:dde2/ein2/sid2:0dpi	0.89365379
dde2:0dpi:dde2/pad4:0dpi	0.92332823
dde2:0dpi:dde2/pad4/sid2:0dpi	0.6061928
dde2:0dpi:dde2/sid2:0dpi	0.98325468
dde2:0dpi:ein2:0dpi	0.4438919
dde2:0dpi:ein2/pad4:0dpi	0.87158002
dde2:0dpi:ein2/pad4/sid2:0dpi	0.93474941
dde2:0dpi:ein2/sid2:0dpi	0.57539886
dde2:0dpi:pad3:0dpi	0.77532403
dde2:0dpi:pad4:0dpi	0.74778115
dde2:0dpi:pad4/sid2:0dpi	0.35380378
dde2:0dpi:pmr4:0dpi	9.02E-01
dde2:0dpi:sid2:0dpi	6.35E-01
dde2:0dpi:Col:3dpi	1.2471E-07
dde2:0dpi:dde2:3dpi	6.18E-21
dde2:0dpi:dde2/ein2:3dpi	1.5034E-33
dde2:0dpi:dde2/ein2/pad4:3dpi	1.15E-18
dde2:0dpi:quad:3dpi	1.32E-36
dde2:0dpi:dde2/ein2/sid2:3dpi	3.06E-16
dde2:0dpi:dde2/pad4:3dpi	1.3948E-19
dde2:0dpi:dde2/pad4/sid2:3dpi	2.0302E-18
dde2:0dpi:dde2/sid2:3dpi	1.476E-18
dde2:0dpi:ein2:3dpi	1.53E-10

dde2:0dpi:ein2/pad4:3dpi	4.2336E-09
dde2:0dpi:ein2/pad4/sid2:3dpi	5.947E-05
dde2:0dpi:ein2/sid2:3dpi	4.1429E-08
dde2:0dpi:pad3:3dpi	1.7877E-27
dde2:0dpi:pad4:3dpi	2.2833E-06
dde2:0dpi:pad4/sid2:3dpi	6.2707E-11
dde2:0dpi:pmr4:3dpi	8.8085E-06
dde2:0dpi:sid2:3dpi	9.1611E-07
dde2/ein2:0dpi:dde2/ein2/pad4:0dpi	0.82570701
dde2/ein2:0dpi:quad:0dpi	0.9479742
dde2/ein2:0dpi:dde2/ein2/sid2:0dpi	0.97300602
dde2/ein2:0dpi:dde2/pad4:0dpi	0.85233704
dde2/ein2:0dpi:dde2/pad4/sid2:0dpi	0.68966384
dde2/ein2:0dpi:dde2/sid2:0dpi	0.94102076
dde2/ein2:0dpi:ein2:0dpi	0.55154853
dde2/ein2:0dpi:ein2/pad4:0dpi	0.80358833
dde2/ein2:0dpi:ein2/pad4/sid2:0dpi	0.98744433
dde2/ein2:0dpi:ein2/sid2:0dpi	0.65847601
dde2/ein2:0dpi:pad3:0dpi	0.90158726
dde2/ein2:0dpi:pad4:0dpi	0.68793652
dde2/ein2:0dpi:pad4/sid2:0dpi	4.27E-01
dde2/ein2:0dpi:pmr4:0dpi	0.83198428
dde2/ein2:0dpi:sid2:0dpi	7.19E-01
dde2/ein2:0dpi:Col:3dpi	4.3173E-07
dde2/ein2:0dpi:dde2:3dpi	2.1662E-19
dde2/ein2:0dpi:dde2/ein2:3dpi	1.194E-31
dde2/ein2:0dpi:dde2/ein2/pad4:3dpi	5.8828E-18
dde2/ein2:0dpi:quad:3dpi	2.5124E-34
dde2/ein2:0dpi:dde2/ein2/sid2:3dpi	1.864E-15
dde2/ein2:0dpi:dde2/pad4:3dpi	1.1961E-18
dde2/ein2:0dpi:dde2/pad4/sid2:3dpi	1.0203E-17
dde2/ein2:0dpi:dde2/sid2:3dpi	1.1447E-17
dde2/ein2:0dpi:ein2:3dpi	7.4714E-10
dde2/ein2:0dpi:ein2/pad4:3dpi	1.1011E-08
dde2/ein2:0dpi:ein2/pad4/sid2:3dpi	6.8877E-05
dde2/ein2:0dpi:ein2/sid2:3dpi	7.8479E-08
dde2/ein2:0dpi:pad3:3dpi	1.4256E-25
dde2/ein2:0dpi:pad4:3dpi	4.1185E-06
dde2/ein2:0dpi:pad4/sid2:3dpi	2.0615E-10
dde2/ein2:0dpi:pmr4:3dpi	1.4665E-05
dde2/ein2:0dpi:sid2:3dpi	1.7442E-06
dde2/ein2/pad4:0dpi:quad:0dpi	0.77887378
dde2/ein2/pad4:0dpi:dde2/ein2/sid2:	0.82180547
dde2/ein2/pad4:0dpi:dde2/pad4:0dp	0.97589452
dde2/ein2/pad4:0dpi:dde2/pad4/sid2	0.58299771
dde2/ein2/pad4:0dpi:dde2/sid2:0dpi	0.89684268
dde2/ein2/pad4:0dpi:ein2:0dpi	0.48719765
dde2/ein2/pad4:0dpi:ein2/pad4:0dpi	0.97983812
dde2/ein2/pad4:0dpi:ein2/pad4/sid2:	0.85614405
dde2/ein2/pad4:0dpi:ein2/sid2:0dpi	0.55731419
dde2/ein2/pad4:0dpi:pad3:0dpi	7.44E-01
dde2/ein2/pad4:0dpi:pad4:0dpi	0.87212375
dde2/ein2/pad4:0dpi:pad4/sid2:0dpi	3.69E-01
dde2/ein2/pad4:0dpi:pmr4:0dpi	0.99430151
dde2/ein2/pad4:0dpi:sid2:0dpi	6.07E-01
dde2/ein2/pad4:0dpi:Col:3dpi	2.3882E-05
dde2/ein2/pad4:0dpi:dde2:3dpi	2.4892E-15
dde2/ein2/pad4:0dpi:dde2/ein2:3dpi	1.1644E-26
dde2/ein2/pad4:0dpi:dde2/ein2/pad4	1.4948E-15
dde2/ein2/pad4:0dpi:quad:3dpi	1.304E-28
dde2/ein2/pad4:0dpi:dde2/ein2/sid2:	6.47E-13
dde2/ein2/pad4:0dpi:dde2/pad4:3dp	9.6079E-16
dde2/ein2/pad4:0dpi:dde2/pad4/sid2	2.4649E-15

dde2/ein2/pad4:0dpi:dde2/sid2:3dpi	7.1897E-15
dde2/ein2/pad4:0dpi:ein2:3dpi	1.1059E-07
dde2/ein2/pad4:0dpi:ein2/pad4:3dpi	4.248E-07
dde2/ein2/pad4:0dpi:ein2/pad4/sid2:	0.00026224
dde2/ein2/pad4:0dpi:ein2/sid2:3dpi	1.3777E-06
dde2/ein2/pad4:0dpi:pad3:3dpi	1.0526E-20
dde2/ein2/pad4:0dpi:pad4:3dpi	5.7753E-05
dde2/ein2/pad4:0dpi:pad4/sid2:3dpi	1.4793E-08
dde2/ein2/pad4:0dpi:pmr4:3dpi	0.0001631
dde2/ein2/pad4:0dpi:sid2:3dpi	2.8522E-05
quad:0dpi:dde2/ein2/sid2:0dpi	0.98780701
quad:0dpi:dde2/pad4:0dpi	0.80631659
quad:0dpi:dde2/pad4/sid2:0dpi	0.71380873
quad:0dpi:dde2/sid2:0dpi	0.89816755
quad:0dpi:ein2:0dpi	0.56575514
quad:0dpi:ein2/pad4:0dpi	7.56E-01
quad:0dpi:ein2/pad4/sid2:0dpi	9.47E-01
quad:0dpi:ein2/sid2:0dpi	0.68078633
quad:0dpi:pad3:0dpi	9.47E-01
quad:0dpi:pad4:0dpi	0.63808724
quad:0dpi:pad4/sid2:0dpi	4.36E-01
quad:0dpi:pmr4:0dpi	7.85E-01
quad:0dpi:sid2:0dpi	7.44E-01
quad:0dpi:Col:3dpi	6.2254E-08
quad:0dpi:dde2:3dpi	2.7682E-21
quad:0dpi:dde2/ein2:3dpi	7.7532E-34
quad:0dpi:dde2/ein2/pad4:3dpi	6.43E-19
quad:0dpi:quad:3dpi	6.6729E-37
quad:0dpi:dde2/ein2/sid2:3dpi	1.6008E-16
quad:0dpi:dde2/pad4:3dpi	7.3122E-20
quad:0dpi:dde2/pad4/sid2:3dpi	1.1372E-18
quad:0dpi:dde2/sid2:3dpi	7.7272E-19
quad:0dpi:ein2:3dpi	7.5058E-11
quad:0dpi:ein2/pad4:3dpi	2.3571E-09
quad:0dpi:ein2/pad4/sid2:3dpi	4.2714E-05
quad:0dpi:ein2/sid2:3dpi	2.497E-08
quad:0dpi:pad3:3dpi	8.3346E-28
quad:0dpi:pad4:3dpi	1.3576E-06
quad:0dpi:pad4/sid2:3dpi	3.3976E-11
quad:0dpi:pmr4:3dpi	5.3408E-06
quad:0dpi:sid2:3dpi	5.3823E-07
dde2/ein2/sid2:0dpi:dde2/pad4:0dpi	0.84538028
dde2/ein2/sid2:0dpi:dde2/pad4/sid2:	0.7458912
dde2/ein2/sid2:0dpi:dde2/sid2:0dpi	0.92384486
dde2/ein2/sid2:0dpi:ein2:0dpi	6.59E-01
dde2/ein2/sid2:0dpi:ein2/pad4:0dpi	0.8022119
dde2/ein2/sid2:0dpi:ein2/pad4/sid2:(	9.65E-01
dde2/ein2/sid2:0dpi:ein2/sid2:0dpi	0.7173975
dde2/ein2/sid2:0dpi:pad3:0dpi	0.95166149
dde2/ein2/sid2:0dpi:pad4:0dpi	0.69943066
dde2/ein2/sid2:0dpi:pad4/sid2:0dpi	0.50034263
dde2/ein2/sid2:0dpi:pmr4:0dpi	0.82736384
dde2/ein2/sid2:0dpi:sid2:0dpi	0.7722197
dde2/ein2/sid2:0dpi:Col:3dpi	1.0017E-05
dde2/ein2/sid2:0dpi:dde2:3dpi	7.8907E-16
dde2/ein2/sid2:0dpi:dde2/ein2:3dpi	4.0822E-27
dde2/ein2/sid2:0dpi:dde2/ein2/pad4:	6.0379E-16
dde2/ein2/sid2:0dpi:quad:3dpi	4.4568E-29
dde2/ein2/sid2:0dpi:dde2/ein2/sid2:3	2.4609E-13
dde2/ein2/sid2:0dpi:dde2/pad4:3dpi	3.5829E-16
dde2/ein2/sid2:0dpi:dde2/pad4/sid2:	9.9649E-16
dde2/ein2/sid2:0dpi:dde2/sid2:3dpi	2.6919E-15
dde2/ein2/sid2:0dpi:ein2:3dpi	4.2713E-08

dde2/ein2/sid2:0dpi:ein2/pad4:3dpi	1.8532E-07
dde2/ein2/sid2:0dpi:ein2/pad4/sid2:3	0.00015733
dde2/ein2/sid2:0dpi:ein2/sid2:3dpi	6.5593E-07
dde2/ein2/sid2:0dpi:pad3:3dpi	3.3468E-21
dde2/ein2/sid2:0dpi:pad4:3dpi	2.8119E-05
dde2/ein2/sid2:0dpi:pad4/sid2:3dpi	6.1291E-09
dde2/ein2/sid2:0dpi:pmr4:3dpi	8.1868E-05
dde2/ein2/sid2:0dpi:sid2:3dpi	1.3626E-05
dde2/pad4:0dpi:dde2/pad4/sid2:0dp	6.04E-01
dde2/pad4:0dpi:dde2/sid2:0dpi	0.92079117
dde2/pad4:0dpi:ein2:0dpi	5.09E-01
dde2/pad4:0dpi:ein2/pad4:0dpi	0.95574977
dde2/pad4:0dpi:ein2/pad4/sid2:0dpi	0.87991726
dde2/pad4:0dpi:ein2/sid2:0dpi	0.57774154
dde2/pad4:0dpi:pad3:0dpi	0.77143604
dde2/pad4:0dpi:pad4:0dpi	0.84839128
dde2/pad4:0dpi:pad4/sid2:0dpi	0.38529156
dde2/pad4:0dpi:pmr4:0dpi	0.981591
dde2/pad4:0dpi:sid2:0dpi	0.62817167
dde2/pad4:0dpi:Col:3dpi	2.1286E-05
dde2/pad4:0dpi:dde2:3dpi	2.134E-15
dde2/pad4:0dpi:dde2/ein2:3dpi	1.0114E-26
dde2/pad4:0dpi:dde2/ein2/pad4:3dp	1.3238E-15
dde2/pad4:0dpi:quad:3dpi	1.1287E-28
dde2/pad4:0dpi:dde2/ein2/sid2:3dpi	5.6836E-13
dde2/pad4:0dpi:dde2/pad4:3dpi	8.4179E-16
dde2/pad4:0dpi:dde2/pad4/sid2:3dp	2.1832E-15
dde2/pad4:0dpi:dde2/sid2:3dpi	6.3029E-15
dde2/pad4:0dpi:ein2:3dpi	9.7431E-08
dde2/pad4:0dpi:ein2/pad4:3dpi	3.8039E-07
dde2/pad4:0dpi:ein2/pad4/sid2:3dpi	0.00024505
dde2/pad4:0dpi:ein2/sid2:3dpi	1.2482E-06
dde2/pad4:0dpi:pad3:3dpi	9.0249E-21
dde2/pad4:0dpi:pad4:3dpi	5.2498E-05
dde2/pad4:0dpi:pad4/sid2:3dpi	1.32E-08
dde2/pad4:0dpi:pmr4:3dpi	0.00014888
dde2/pad4:0dpi:sid2:3dpi	2.59E-05
dde2/pad4/sid2:0dpi:dde2/sid2:0dpi	0.67480352
dde2/pad4/sid2:0dpi:ein2:0dpi	9.40E-01
dde2/pad4/sid2:0dpi:ein2/pad4:0dpi	5.66E-01
dde2/pad4/sid2:0dpi:ein2/pad4/sid2:	0.71289044
dde2/pad4/sid2:0dpi:ein2/sid2:0dpi	0.96976126
dde2/pad4/sid2:0dpi:pad3:0dpi	0.74787413
dde2/pad4/sid2:0dpi:pad4:0dpi	0.4779868
dde2/pad4/sid2:0dpi:pad4/sid2:0dpi	7.26E-01
dde2/pad4/sid2:0dpi:pmr4:0dpi	0.5878986
dde2/pad4/sid2:0dpi:sid2:0dpi	0.9723896
dde2/pad4/sid2:0dpi:Col:3dpi	2.7476E-06
dde2/pad4/sid2:0dpi:dde2:3dpi	1.5048E-16
dde2/pad4/sid2:0dpi:dde2/ein2:3dpi	9.11E-28
dde2/pad4/sid2:0dpi:dde2/ein2/pad4	1.6344E-16
dde2/pad4/sid2:0dpi:quad:3dpi	9.6026E-30
dde2/pad4/sid2:0dpi:dde2/ein2/sid2:	6.0794E-14
dde2/pad4/sid2:0dpi:dde2/pad4:3dp	8.6455E-17
dde2/pad4/sid2:0dpi:dde2/pad4/sid2	2.7E-16
dde2/pad4/sid2:0dpi:dde2/sid2:3dpi	6.5236E-16
dde2/pad4/sid2:0dpi:ein2:3dpi	1.0568E-08
dde2/pad4/sid2:0dpi:ein2/pad4:3dpi	5.4799E-08
dde2/pad4/sid2:0dpi:ein2/pad4/sid2:	7.3811E-05
dde2/pad4/sid2:0dpi:ein2/sid2:3dpi	2.20E-07
dde2/pad4/sid2:0dpi:pad3:3dpi	6.466E-22
dde2/pad4/sid2:0dpi:pad4:3dpi	9.65E-06
dde2/pad4/sid2:0dpi:pad4/sid2:3dpi	1.6916E-09

dde2/pad4/sid2:0dpi:pmr4:3dpi	2.9293E-05
dde2/pad4/sid2:0dpi:sid2:3dpi	4.5565E-06
dde2/sid2:0dpi:ein2:0dpi	0.58306453
dde2/sid2:0dpi:ein2/pad4:0dpi	0.876885
dde2/sid2:0dpi:ein2/pad4/sid2:0dpi	0.95881375
dde2/sid2:0dpi:ein2/sid2:0dpi	0.64736732
dde2/sid2:0dpi:pad3:0dpi	0.86240923
dde2/sid2:0dpi:pad4:0dpi	0.77137959
dde2/sid2:0dpi:pad4/sid2:0dpi	0.44177226
dde2/sid2:0dpi:pmr4:0dpi	0.90249548
dde2/sid2:0dpi:sid2:0dpi	0.70024373
dde2/sid2:0dpi:Col:3dpi	1.4528E-05
dde2/sid2:0dpi:dde2:3dpi	1.2853E-15
dde2/sid2:0dpi:dde2/ein2:3dpi	6.3653E-27
dde2/sid2:0dpi:dde2/ein2/pad4:3dpi	8.8726E-16
dde2/sid2:0dpi:quad:3dpi	7.0242E-29
dde2/sid2:0dpi:dde2/ein2/sid2:3dpi	3.7107E-13
dde2/sid2:0dpi:dde2/pad4:3dpi	5.4466E-16
dde2/sid2:0dpi:dde2/pad4/sid2:3dpi	1.4638E-15
dde2/sid2:0dpi:dde2/sid2:3dpi	4.0856E-15
dde2/sid2:0dpi:ein2:3dpi	6.41E-08
dde2/sid2:0dpi:ein2/pad4:3dpi	2.6399E-07
dde2/sid2:0dpi:ein2/pad4/sid2:3dpi	1.96E-04
dde2/sid2:0dpi:ein2/sid2:3dpi	9.0023E-07
dde2/sid2:0dpi:pad3:3dpi	5.44E-21
dde2/sid2:0dpi:pad4:3dpi	3.83E-05
dde2/sid2:0dpi:pad4/sid2:3dpi	8.9208E-09
dde2/sid2:0dpi:pmr4:3dpi	0.00010997
dde2/sid2:0dpi:sid2:3dpi	1.8686E-05
ein2:0dpi:ein2/pad4:0dpi	0.46956881
ein2:0dpi:ein2/pad4/sid2:0dpi	6.24E-01
ein2:0dpi:ein2/sid2:0dpi	0.9736043
ein2:0dpi:pad3:0dpi	0.60583775
ein2:0dpi:pad4:0dpi	0.38126123
ein2:0dpi:pad4/sid2:0dpi	0.74939137
ein2:0dpi:pmr4:0dpi	0.4922446
ein2:0dpi:sid2:0dpi	0.90853701
ein2:0dpi:Col:3dpi	5.0718E-08
ein2:0dpi:dde2:3dpi	1.754E-20
ein2:0dpi:dde2/ein2:3dpi	1.4281E-32
ein2:0dpi:dde2/ein2/pad4:3dpi	9.1193E-19
ein2:0dpi:quad:3dpi	2.855E-35
ein2:0dpi:dde2/ein2/sid2:3dpi	2.3808E-16
ein2:0dpi:dde2/pad4:3dpi	1.52E-19
ein2:0dpi:dde2/pad4/sid2:3dpi	1.5812E-18
ein2:0dpi:dde2/sid2:3dpi	1.45E-18
ein2:0dpi:ein2:3dpi	8.1758E-11
ein2:0dpi:ein2/pad4:3dpi	1.72E-09
ein2:0dpi:ein2/pad4/sid2:3dpi	2.3205E-05
ein2:0dpi:ein2/sid2:3dpi	1.5437E-08
ein2:0dpi:pad3:3dpi	1.2759E-26
ein2:0dpi:pad4:3dpi	7.8594E-07
ein2:0dpi:pad4/sid2:3dpi	2.9478E-11
ein2:0dpi:pmr4:3dpi	2.97E-06
ein2:0dpi:sid2:3dpi	3.2091E-07
ein2/pad4:0dpi:ein2/pad4/sid2:0dpi	0.83636119
ein2/pad4:0dpi:ein2/sid2:0dpi	0.54050777
ein2/pad4:0dpi:pad3:0dpi	0.72193989
ein2/pad4:0dpi:pad4:0dpi	0.89206368
ein2/pad4:0dpi:pad4/sid2:0dpi	0.35581332
ein2/pad4:0dpi:pmr4:0dpi	0.974142
ein2/pad4:0dpi:sid2:0dpi	0.58941044
ein2/pad4:0dpi:Col:3dpi	2.6285E-05

ein2/pad4:0dpi:dde2:3dpi	2.8311E-15
ein2/pad4:0dpi:dde2/ein2:3dpi	1.3101E-26
ein2/pad4:0dpi:dde2/ein2/pad4:3dpi	1.65E-15
ein2/pad4:0dpi:quad:3dpi	1.4714E-28
ein2/pad4:0dpi:dde2/ein2/sid2:3dpi	7.21E-13
ein2/pad4:0dpi:dde2/pad4:3dpi	1.0731E-15
ein2/pad4:0dpi:dde2/pad4/sid2:3dpi	2.73E-15
ein2/pad4:0dpi:dde2/sid2:3dpi	8.03E-15
ein2/pad4:0dpi:ein2:3dpi	1.2291E-07
ein2/pad4:0dpi:ein2/pad4:3dpi	4.658E-07
ein2/pad4:0dpi:ein2/pad4/sid2:3dpi	0.00027749
ein2/pad4:0dpi:ein2/sid2:3dpi	1.4961E-06
ein2/pad4:0dpi:pad3:3dpi	1.20E-20
ein2/pad4:0dpi:pad4:3dpi	6.2532E-05
ein2/pad4:0dpi:pad4/sid2:3dpi	1.6318E-08
ein2/pad4:0dpi:pmr4:3dpi	0.00017598
ein2/pad4:0dpi:sid2:3dpi	3.095E-05
ein2/pad4/sid2:0dpi:ein2/sid2:0dpi	0.68485581
ein2/pad4/sid2:0dpi:pad3:0dpi	0.9104798
ein2/pad4/sid2:0dpi:pad4:0dpi	0.73221699
ein2/pad4/sid2:0dpi:pad4/sid2:0dpi	0.47290749
ein2/pad4/sid2:0dpi:pmr4:0dpi	0.86175209
ein2/pad4/sid2:0dpi:sid2:0dpi	7.39E-01
ein2/pad4/sid2:0dpi:Col:3dpi	1.19E-05
ein2/pad4/sid2:0dpi:dde2:3dpi	9.88E-16
ein2/pad4/sid2:0dpi:dde2/ein2:3dpi	5.01E-27
ein2/pad4/sid2:0dpi:dde2/ein2/pad4:	7.207E-16
ein2/pad4/sid2:0dpi:quad:3dpi	5.49E-29
ein2/pad4/sid2:0dpi:dde2/ein2/sid2:3	2.97E-13
ein2/pad4/sid2:0dpi:dde2/pad4:3dpi	4.34E-16
ein2/pad4/sid2:0dpi:dde2/pad4/sid2:	1.1893E-15
ein2/pad4/sid2:0dpi:dde2/sid2:3dpi	3.2613E-15
ein2/pad4/sid2:0dpi:ein2:3dpi	5.1491E-08
ein2/pad4/sid2:0dpi:ein2/pad4:3dpi	2.18E-07
ein2/pad4/sid2:0dpi:ein2/pad4/sid2:3	0.00017399
ein2/pad4/sid2:0dpi:ein2/sid2:3dpi	7.5893E-07
ein2/pad4/sid2:0dpi:pad3:3dpi	4.1845E-21
ein2/pad4/sid2:0dpi:pad4:3dpi	3.2409E-05
ein2/pad4/sid2:0dpi:pad4/sid2:3dpi	7.2854E-09
ein2/pad4/sid2:0dpi:pmr4:3dpi	9.3807E-05
ein2/pad4/sid2:0dpi:sid2:3dpi	1.5762E-05
ein2/sid2:0dpi:pad3:0dpi	0.71429152
ein2/sid2:0dpi:pad4:0dpi	0.45487364
ein2/sid2:0dpi:pad4/sid2:0dpi	7.55E-01
ein2/sid2:0dpi:pmr4:0dpi	0.56211016
ein2/sid2:0dpi:sid2:0dpi	9.42E-01
ein2/sid2:0dpi:Col:3dpi	2.3544E-06
ein2/sid2:0dpi:dde2:3dpi	1.2394E-16
ein2/sid2:0dpi:dde2/ein2:3dpi	7.6494E-28
ein2/sid2:0dpi:dde2/ein2/pad4:3dpi	1.4026E-16
ein2/sid2:0dpi:quad:3dpi	8.0309E-30
ein2/sid2:0dpi:dde2/ein2/sid2:3dpi	5.1597E-14
ein2/sid2:0dpi:dde2/pad4:3dpi	7.3202E-17
ein2/sid2:0dpi:dde2/pad4/sid2:3dpi	2.3172E-16
ein2/sid2:0dpi:dde2/sid2:3dpi	5.5258E-16
ein2/sid2:0dpi:ein2:3dpi	8.957E-09
ein2/sid2:0dpi:ein2/pad4:3dpi	4.7435E-08
ein2/sid2:0dpi:ein2/pad4/sid2:3dpi	6.745E-05
ein2/sid2:0dpi:ein2/sid2:3dpi	1.9366E-07
ein2/sid2:0dpi:pad3:3dpi	5.3372E-22
ein2/sid2:0dpi:pad4:3dpi	8.4943E-06
ein2/sid2:0dpi:pad4/sid2:3dpi	1.45E-09
ein2/sid2:0dpi:pmr4:3dpi	2.59E-05

ein2/sid2:0dpi:sid2:3dpi	3.999E-06
pad3:0dpi:pad4:0dpi	6.06E-01
pad3:0dpi:pad4/sid2:0dpi	0.46304205
pad3:0dpi:pmr4:0dpi	7.51E-01
pad3:0dpi:sid2:0dpi	7.79E-01
pad3:0dpi:Col:3dpi	5.0299E-08
pad3:0dpi:dde2:3dpi	2.1684E-21
pad3:0dpi:dde2/ein2:3dpi	6.3413E-34
pad3:0dpi:dde2/ein2/pad4:3dpi	5.3892E-19
pad3:0dpi:quad:3dpi	5.43E-37
pad3:0dpi:dde2/ein2/sid2:3dpi	1.3148E-16
pad3:0dpi:dde2/pad4:3dpi	6.0082E-20
pad3:0dpi:dde2/pad4/sid2:3dpi	9.5339E-19
pad3:0dpi:dde2/sid2:3dpi	6.3461E-19
pad3:0dpi:ein2:3dpi	6.0407E-11
pad3:0dpi:ein2/pad4:3dpi	1.9706E-09
pad3:0dpi:ein2/pad4/sid2:3dpi	3.8589E-05
pad3:0dpi:ein2/sid2:3dpi	2.14E-08
pad3:0dpi:pad3:3dpi	6.6107E-28
pad3:0dpi:pad4:3dpi	1.16E-06
pad3:0dpi:pad4/sid2:3dpi	2.8177E-11
pad3:0dpi:pmr4:3dpi	4.5795E-06
pad3:0dpi:sid2:3dpi	4.572E-07
pad4:0dpi:pad4/sid2:0dpi	0.28998186
pad4:0dpi:pmr4:0dpi	0.86650288
pad4:0dpi:sid2:0dpi	0.49964647
pad4:0dpi:Col:3dpi	4.3733E-05
pad4:0dpi:dde2:3dpi	5.6475E-15
pad4:0dpi:dde2/ein2:3dpi	2.4697E-26
pad4:0dpi:dde2/ein2/pad4:3dpi	2.8544E-15
pad4:0dpi:quad:3dpi	2.8181E-28
pad4:0dpi:dde2/ein2/sid2:3dpi	1.2867E-12
pad4:0dpi:dde2/pad4:3dpi	1.9423E-15
pad4:0dpi:dde2/pad4/sid2:3dpi	4.70E-15
pad4:0dpi:dde2/sid2:3dpi	1.45E-14
pad4:0dpi:ein2:3dpi	2.1599E-07
pad4:0dpi:ein2/pad4:3dpi	7.61E-07
pad4:0dpi:ein2/pad4/sid2:3dpi	0.0003749
pad4:0dpi:ein2/sid2:3dpi	2.32E-06
pad4:0dpi:pad3:3dpi	2.39E-20
pad4:0dpi:pad4:3dpi	9.5411E-05
pad4:0dpi:pad4/sid2:3dpi	2.7565E-08
pad4:0dpi:pmr4:3dpi	0.00026347
pad4:0dpi:sid2:3dpi	4.7788E-05
pad4/sid2:0dpi:pmr4:0dpi	3.73E-01
pad4/sid2:0dpi:sid2:0dpi	0.70024373
pad4/sid2:0dpi:Col:3dpi	6.4311E-07
pad4/sid2:0dpi:dde2:3dpi	2.4998E-17
pad4/sid2:0dpi:dde2/ein2:3dpi	1.8203E-28
pad4/sid2:0dpi:dde2/ein2/pad4:3dpi	3.97E-17
pad4/sid2:0dpi:quad:3dpi	1.85E-30
pad4/sid2:0dpi:dde2/ein2/sid2:3dpi	1.3292E-14
pad4/sid2:0dpi:dde2/pad4:3dpi	1.85E-17
pad4/sid2:0dpi:dde2/pad4/sid2:3dpi	6.5624E-17
pad4/sid2:0dpi:dde2/sid2:3dpi	1.4036E-16
pad4/sid2:0dpi:ein2:3dpi	2.2565E-09
pad4/sid2:0dpi:ein2/pad4:3dpi	1.4235E-08
pad4/sid2:0dpi:ein2/pad4/sid2:3dpi	3.1678E-05
pad4/sid2:0dpi:ein2/sid2:3dpi	6.5868E-08
pad4/sid2:0dpi:pad3:3dpi	1.101E-22
pad4/sid2:0dpi:pad4:3dpi	2.91E-06
pad4/sid2:0dpi:pad4/sid2:3dpi	4.1068E-10
pad4/sid2:0dpi:pmr4:3dpi	9.2148E-06

pad4/sid2:0dpi:sid2:3dpi	1.3385E-06
pmr4:0dpi:sid2:0dpi	0.61191811
pmr4:0dpi:Col:3dpi	2.3242E-05
pmr4:0dpi:dde2:3dpi	2.4002E-15
pmr4:0dpi:dde2/ein2:3dpi	1.1262E-26
pmr4:0dpi:dde2/ein2/pad4:3dpi	1.4525E-15
pmr4:0dpi:quad:3dpi	1.2602E-28
pmr4:0dpi:dde2/ein2/sid2:3dpi	6.2738E-13
pmr4:0dpi:dde2/pad4:3dpi	9.3123E-16
pmr4:0dpi:dde2/pad4/sid2:3dpi	2.3952E-15
pmr4:0dpi:dde2/sid2:3dpi	6.9695E-15
pmr4:0dpi:ein2:3dpi	1.0733E-07
pmr4:0dpi:ein2/pad4:3dpi	4.1386E-07
pmr4:0dpi:ein2/pad4/sid2:3dpi	0.00025808
pmr4:0dpi:ein2/sid2:3dpi	1.346E-06
pmr4:0dpi:pad3:3dpi	1.015E-20
pmr4:0dpi:pad4:3dpi	5.6467E-05
pmr4:0dpi:pad4/sid2:3dpi	1.4388E-08
pmr4:0dpi:pmr4:3dpi	0.00015963
pmr4:0dpi:sid2:3dpi	2.787E-05
sid2:0dpi:Col:3dpi	3.162E-06
sid2:0dpi:dde2:3dpi	1.7964E-16
sid2:0dpi:dde2/ein2:3dpi	1.0687E-27
sid2:0dpi:dde2/ein2/pad4:3dpi	1.8794E-16
sid2:0dpi:quad:3dpi	1.1306E-29
sid2:0dpi:dde2/ein2/sid2:3dpi	7.061E-14
sid2:0dpi:dde2/pad4:3dpi	1.0064E-16
sid2:0dpi:dde2/pad4/sid2:3dpi	3.10E-16
sid2:0dpi:dde2/sid2:3dpi	7.5909E-16
sid2:0dpi:ein2:3dpi	1.23E-08
sid2:0dpi:ein2/pad4:3dpi	6.25E-08
sid2:0dpi:ein2/pad4/sid2:3dpi	8.0119E-05
sid2:0dpi:ein2/sid2:3dpi	2.4791E-07
sid2:0dpi:pad3:3dpi	7.7045E-22
sid2:0dpi:pad4:3dpi	1.0838E-05
sid2:0dpi:pad4/sid2:3dpi	1.9429E-09
sid2:0dpi:pmr4:3dpi	3.2755E-05
sid2:0dpi:sid2:3dpi	5.1309E-06
Col:3dpi:dde2:3dpi	6.1113E-14
Col:3dpi:dde2/ein2:3dpi	2.2663E-31
Col:3dpi:dde2/ein2/pad4:3dpi	6.0607E-12
Col:3dpi:quad:3dpi	7.0577E-37
Col:3dpi:dde2/ein2/sid2:3dpi	9.7272E-09
Col:3dpi:dde2/pad4:3dpi	1.4981E-12
Col:3dpi:dde2/pad4/sid2:3dpi	1.1468E-11
Col:3dpi:dde2/sid2:3dpi	2.3889E-11
Col:3dpi:ein2:3dpi	0.01799896
Col:3dpi:ein2/pad4:3dpi	0.02506275
Col:3dpi:ein2/pad4/sid2:3dpi	2.90E-01
Col:3dpi:ein2/sid2:3dpi	3.35E-02
Col:3dpi:pad3:3dpi	1.5866E-23
Col:3dpi:pad4:3dpi	0.56938801
Col:3dpi:pad4/sid2:3dpi	1.21E-03
Col:3dpi:pmr4:3dpi	0.86318258
Col:3dpi:sid2:3dpi	4.07E-01
dde2:3dpi:dde2/ein2:3dpi	1.41E-14
dde2:3dpi:dde2/ein2/pad4:3dpi	0.00792288
dde2:3dpi:quad:3dpi	1.8215E-18
dde2:3dpi:dde2/ein2/sid2:3dpi	0.72445482
dde2:3dpi:dde2/pad4:3dpi	0.03554948
dde2:3dpi:dde2/pad4/sid2:3dpi	0.01122632
dde2:3dpi:dde2/sid2:3dpi	0.11615806
dde2:3dpi:ein2:3dpi	1.1986E-06

dde2:3dpi:ein2/pad4:3dpi	0.00050727
dde2:3dpi:ein2/pad4/sid2:3dpi	0.0090256
dde2:3dpi:ein2/sid2:3dpi	0.00520558
dde2:3dpi:pad3:3dpi	5.4439E-05
dde2:3dpi:pad4:3dpi	6.4566E-07
dde2:3dpi:pad4/sid2:3dpi	0.0127705
dde2:3dpi:pmr4:3dpi	1.104E-07
dde2:3dpi:sid2:3dpi	1.9805E-06
dde2/ein2:3dpi:dde2/ein2/pad4:3dpi	0.00256374
dde2/ein2:3dpi:quad:3dpi	0.69400023
dde2/ein2:3dpi:dde2/ein2/sid2:3dpi	7.66E-09
dde2/ein2:3dpi:dde2/pad4:3dpi	1.332E-05
dde2/ein2:3dpi:dde2/pad4/sid2:3dpi	1.75E-03
dde2/ein2:3dpi:dde2/sid2:3dpi	1.5441E-06
dde2/ein2:3dpi:ein2:3dpi	4.2278E-24
dde2/ein2:3dpi:ein2/pad4:3dpi	3.4232E-17
dde2/ein2:3dpi:ein2/pad4/sid2:3dpi	3.2533E-10
dde2/ein2:3dpi:ein2/sid2:3dpi	1.2519E-13
dde2/ein2:3dpi:pad3:3dpi	9.3058E-07
dde2/ein2:3dpi:pad4:3dpi	6.3548E-21
dde2/ein2:3dpi:pad4/sid2:3dpi	6.8935E-15
dde2/ein2:3dpi:pmr4:3dpi	8.6383E-22
dde2/ein2:3dpi:sid2:3dpi	2.3623E-20
dde2/ein2/pad4:3dpi:quad:3dpi	0.00079425
dde2/ein2/pad4:3dpi:dde2/ein2/sid2:	0.03628544
dde2/ein2/pad4:3dpi:dde2/pad4:3dp	0.43634014
dde2/ein2/pad4:3dpi:dde2/pad4/sid2	0.92026934
dde2/ein2/pad4:3dpi:dde2/sid2:3dpi	0.23584114
dde2/ein2/pad4:3dpi:ein2:3dpi	3.7377E-08
dde2/ein2/pad4:3dpi:ein2/pad4:3dpi	1.5012E-06
dde2/ein2/pad4:3dpi:ein2/pad4/sid2:	7.5775E-05
dde2/ein2/pad4:3dpi:ein2/sid2:3dpi	2.1964E-05
dde2/ein2/pad4:3dpi:pad3:3dpi	0.79447034
dde2/ein2/pad4:3dpi:pad4:3dpi	4.5007E-09
dde2/ein2/pad4:3dpi:pad4/sid2:3dpi	3.78E-05
dde2/ein2/pad4:3dpi:pmr4:3dpi	1.0637E-09
dde2/ein2/pad4:3dpi:sid2:3dpi	1.1417E-08
quad:3dpi:dde2/ein2/sid2:3dpi	4.4952E-10
quad:3dpi:dde2/pad4:3dpi	1.6032E-06
quad:3dpi:dde2/pad4/sid2:3dpi	0.00051927
quad:3dpi:dde2/sid2:3dpi	1.4764E-07
quad:3dpi:ein2:3dpi	4.1983E-27
quad:3dpi:ein2/pad4:3dpi	6.1708E-19
quad:3dpi:ein2/pad4/sid2:3dpi	6.581E-11
quad:3dpi:ein2/sid2:3dpi	7.3551E-15
quad:3dpi:pad3:3dpi	4.3174E-09
quad:3dpi:pad4:3dpi	8.1816E-23
quad:3dpi:pad4/sid2:3dpi	1.6061E-16
quad:3dpi:pmr4:3dpi	1.0407E-23
quad:3dpi:sid2:3dpi	3.18E-22
dde2/ein2/sid2:3dpi:dde2/pad4:3dpi	0.13507534
dde2/ein2/sid2:3dpi:dde2/pad4/sid2:	4.70E-02
dde2/ein2/sid2:3dpi:dde2/sid2:3dpi	0.29902264
dde2/ein2/sid2:3dpi:ein2:3dpi	6.4292E-05
dde2/ein2/sid2:3dpi:ein2/pad4:3dpi	0.00114807
dde2/ein2/sid2:3dpi:ein2/pad4/sid2:3	0.00850485
dde2/ein2/sid2:3dpi:ein2/sid2:3dpi	0.00636499
dde2/ein2/sid2:3dpi:pad3:3dpi	0.01369921
dde2/ein2/sid2:3dpi:pad4:3dpi	5.4245E-06
dde2/ein2/sid2:3dpi:pad4/sid2:3dpi	0.01598631
dde2/ein2/sid2:3dpi:pmr4:3dpi	1.3145E-06
dde2/ein2/sid2:3dpi:sid2:3dpi	1.3326E-05
dde2/pad4:3dpi:dde2/pad4/sid2:3dp	0.50403757

dde2/pad4:3dpi:dde2/sid2:3dpi	0.64500367
dde2/pad4:3dpi:ein2:3dpi	5.0404E-08
dde2/pad4:3dpi:ein2/pad4:3dpi	4.185E-06
dde2/pad4:3dpi:ein2/pad4/sid2:3dpi	0.00030648
dde2/pad4:3dpi:ein2/sid2:3dpi	7.3625E-05
dde2/pad4:3dpi:pad3:3dpi	0.46664798
dde2/pad4:3dpi:pad4:3dpi	6.694E-09
dde2/pad4:3dpi:pad4/sid2:3dpi	0.00013705
dde2/pad4:3dpi:pmr4:3dpi	1.3302E-09
dde2/pad4:3dpi:sid2:3dpi	1.894E-08
dde2/pad4/sid2:3dpi:dde2/sid2:3dpi	0.28202736
dde2/pad4/sid2:3dpi:ein2:3dpi	6.5302E-08
dde2/pad4/sid2:3dpi:ein2/pad4:3dpi	2.402E-06
dde2/pad4/sid2:3dpi:ein2/pad4/sid2:	0.00013712
dde2/pad4/sid2:3dpi:ein2/sid2:3dpi	3.2505E-05
dde2/pad4/sid2:3dpi:pad3:3dpi	0.89250273
dde2/pad4/sid2:3dpi:pad4:3dpi	7.6162E-09
dde2/pad4/sid2:3dpi:pad4/sid2:3dpi	5.7777E-05
dde2/pad4/sid2:3dpi:pmr4:3dpi	1.8183E-09
dde2/pad4/sid2:3dpi:sid2:3dpi	1.9182E-08
dde2/sid2:3dpi:ein2:3dpi	5.212E-07
dde2/sid2:3dpi:ein2/pad4:3dpi	2.6926E-05
dde2/sid2:3dpi:ein2/pad4/sid2:3dpi	0.00092005
dde2/sid2:3dpi:ein2/sid2:3dpi	0.0003275
dde2/sid2:3dpi:pad3:3dpi	0.20494769
dde2/sid2:3dpi:pad4:3dpi	5.7545E-08
dde2/sid2:3dpi:pad4/sid2:3dpi	0.00069486
dde2/sid2:3dpi:pmr4:3dpi	1.2036E-08
dde2/sid2:3dpi:sid2:3dpi	1.5692E-07
ein2:3dpi:ein2/pad4:3dpi	0.69752081
ein2:3dpi:ein2/pad4/sid2:3dpi	0.90529771
ein2:3dpi:ein2/sid2:3dpi	0.58891024
ein2:3dpi:pad3:3dpi	1.9152E-14
ein2:3dpi:pad4:3dpi	0.21850016
ein2:3dpi:pad4/sid2:3dpi	0.16556388
ein2:3dpi:pmr4:3dpi	0.10845456
ein2:3dpi:sid2:3dpi	0.32536203
ein2/pad4:3dpi:ein2/pad4/sid2:3dpi	0.72435699
ein2/pad4:3dpi:ein2/sid2:3dpi	0.85208617
ein2/pad4:3dpi:pad3:3dpi	3.206E-09
ein2/pad4:3dpi:pad4:3dpi	0.15195374
ein2/pad4:3dpi:pad4/sid2:3dpi	0.37432126
ein2/pad4:3dpi:pmr4:3dpi	0.07780156
ein2/pad4:3dpi:sid2:3dpi	0.22457705
ein2/pad4/sid2:3dpi:ein2/sid2:3dpi	0.63663434
ein2/pad4/sid2:3dpi:pad3:3dpi	1.8674E-05
ein2/pad4/sid2:3dpi:pad4:3dpi	0.51613343
ein2/pad4/sid2:3dpi:pad4/sid2:3dpi	0.33173149
ein2/pad4/sid2:3dpi:pmr4:3dpi	0.3772542
ein2/pad4/sid2:3dpi:sid2:3dpi	0.62002352
ein2/sid2:3dpi:pad3:3dpi	6.1637E-07
ein2/sid2:3dpi:pad4:3dpi	0.1445543
ein2/sid2:3dpi:pad4/sid2:3dpi	0.54565897
ein2/sid2:3dpi:pmr4:3dpi	0.07956861
ein2/sid2:3dpi:sid2:3dpi	0.2058098
pad3:3dpi:pad4:3dpi	6.5863E-13
pad3:3dpi:pad4/sid2:3dpi	4.0555E-07
pad3:3dpi:pmr4:3dpi	8.3858E-14
pad3:3dpi:sid2:3dpi	2.5152E-12
pad4:3dpi:pad4/sid2:3dpi	0.0213734
pad4:3dpi:pmr4:3dpi	0.73635183
pad4:3dpi:sid2:3dpi	0.82522495
pad4/sid2:3dpi:pmr4:3dpi	0.00868008

pad4/sid2:3dpi:sid2:3dpi	0.03683159
pmr4:3dpi:sid2:3dpi	0.57736697
