# Supplementary material for: Network Properties of Robust Immunity in Plants
Source: PLoS Genet. 2009 Dec 11;5(12):e1000772. doi: 10.1371/journal.pgen.1000772 (PMC2782137; doi:10.1371/journal.pgen.1000772)
Supplement: Text S1 — Information for Table 1. (0.04 MB DOC) [file pgen.1000772.s019.doc]

**Supporting Information (For Table 1)**

*A. Gene interaction variable values.*

When the effects and interactions of four genes, D, E, P, and S are modeled:

(i) Two wild-type genes. The immunity contribution in the genotype DE (i.e., p/s double mutant) can be explained by the sum of the D effect, E effect, and the interaction between D and E; D + E + D:E. Therefore, the variable value corresponding to the 2-gene interaction is 1.

(ii) Three wild-type genes. The immunity contribution in the genotype DEP (i.e., s mutant) is denoted in three ways using the immunity contributions of the gene effects and the 2-gene interactions.

As the combination of DE and P,

DE + P + DE:P = (D + E + D:E) + P + DE:P … (a)

As the combination of EP and D,

EP + D + EP:D = (E + P + E:P) + D + EP:D … (b)

As the combination of DP and E,

DP + E + DP:E = (P + D + D:P) + E + DP:E … (c)

The notations (a)-(c) are equivalent and inclusive. Therefore, the immunity contribution in DEP can be considered as the equal mix of the three states (i.e., average of the three).

DEP = D + E + P + (D:E + E:P + D:P) / 3 + (DE:P + EP:D + DP:E) / 3 … (d)

Since we cannot assume the distributivity for the operators “:” and “+” (see below “*B. Non-distributivity*” for the proof), DE:P + EP:D + DP:E cannot be generally written by a linear combination of the gene effects and the 2-gene interactions. Therefore, it is reasonable to define D:E:P = (DE:P + EP:D + DP:E) / 3 as the term that cannot be explained by the gene effects and the 2-gene interactions. So,

DEP = D + E + P + (D:E + E:P + D:P) / 3 + D:E:P … (e)

Therefore, the variable values corresponding to the 2-gene interactions are 1/3 in a single mutant genotype.

(iii) Four wild-type genes. Similarly, the immunity contribution in the genotype DEPS (i.e., wild type) is denoted in four ways using the immunity contributions of the gene effects and the 2- and 3-gene interactions.

As the combination of DEP and S,

DEP + S + DEP:S = (D + E + P + (D:E + E:P + D:P) / 3 + D:E:P) + S + DEP:S … (f)

As the combination of DES and P,

DES + P + DES:P = (D + E + S + (D:E + E:S + D:S) / 3 + D:E:S) + P + DES:P … (g)

As the combination of DPS and E,

DPS + E + DPS:E = (D + P + S + (D:P + P:S + D:S) / 3 + D:P:S) + E + DPS:E … (h)

As the combination of EPS and D,

EPS + D + EPS:D = (E + P + S + (E:P + P:S + E:S) / 3 + E:P:S) + D + EPS:D … (i)

The notations (f)-(i) are equivalent and inclusive. The immunity contribution in DEPS can be considered as the equal mix of the four states (i.e., average of the four).

DEPS = D + E + P + S + (D:E + D:P + D:S + E:P + E:S + P:S) / 6 +

(D:E:P + D:E:S + D:P:S + E:P:S) / 4 + D:E:P:S … (j)

where D:E:P:S = (DEP:S + DES:P + DPS:E + EPS:D) / 4.

Therefore the variables corresponding to the 2- and 3-gene interactions are 1/6 and 1/4, respectively, in the wild type genotype.

*B. Non-distributivity*

In section *A* (ii), if we assume the distributivity regarding “:” and “+”,

DE:P = (D + E + D:E):P = D:P + E:P + (D:E):P

So, (a) becomes

DE + P + DE:P = D + E + P + D:E + D:P + E:P + DE:P … (k)

Similarly, (b) becomes

EP + D + EP:D = D + E + P + D:E + D:P + E:P + EP:D … (l)

Similarly, (c) becomes,

DP + E + DP:E = D + E + P + D:E + D:P + E:P + DP:E … (m)

Since (k), (l), and (m) are identical,

DE:P = EP:D = DP:E … (n)

However, we can easily imagine the following situation: signaling sectors D and P directly interact, signaling sectors E and P directly interact, but signaling sectors D and E do not directly interact; i.e., D:P ≠0, E:P ≠0, D:E = 0. So, (D:E):P = 0. However, it is possible that D and E can interact through P, i.e., (E:P):D ≠0 or (D:P):E≠0. This contradicts with (n). Therefore, the initial assumption of the distributivity is false.

Similarly, the non-distributivity regarding “:” and “+” can be proven in the case of *A* (iii).
