# Supplementary material for: Network Properties of Robust Immunity in Plants
Source: PLoS Genet. 2009 Dec 11;5(12):e1000772. doi: 10.1371/journal.pgen.1000772 (PMC2782137; doi:10.1371/journal.pgen.1000772)
Supplement: Text S2 — Supporting materials and methods. (0.04 MB DOC) [file pgen.1000772.s020.doc]

**Supporting Materials and Methods**

**Macroscopic HR assay.**

Plants were inoculated with *Pto* DC3000 carrying pLAFR or expressing AvrRpt2, AvrRpm1 or AvrPphB (OD_600_ = 0.05, 0.5 x 10^8^ CFU ml^-1^). Leaves were photographed 24 hpi.

**Electrolyte leakage assay.**

The use of an electrolyte leakage assay as a measure of HR has been described previously [1]. In short, per sample, two leaves per plant were inoculated with *Pto* DC3000 carrying an empty vector (pLAFR) or expressing AvrRpt2 or AvrRpm1 (OD_600_ = 0.1, 1x 10^8^ CFU ml^-1^). One hour after inoculation, two leaf discs per leaf from four leaves per plant were transferred to a Petri dish containing 25 ml of water and placed on a shaker for 1 h. After this washing step, the leaf discs were transferred to glass tubes containing 6 ml of water. The conductivity of the samples was determined using a portable conductivity meter (VWR Scientific, Batavia, IL, USA) at the indicated time points. This experiment was repeated twice, and data from two independent experiments were combined.

**References**

1. Van Poecke RM, Sato M, Lenarz-Wyatt L, Weisberg S, Katagiri F. (2007) Natural variation in RPS2-mediated resistance among arabidopsis accessions: Correlation between gene expression profiles and phenotypic responses. Plant Cell 19(12): 4046-4060.
